# Aplysinopsin-type and Bromotyrosine-derived Alkaloids from the South China Sea Sponge *Fascaplysinopsis reticulata*

**DOI:** 10.1038/s41598-019-38696-3

**Published:** 2019-02-19

**Authors:** Qi Wang, Xu-Li Tang, Xiang-Chao Luo, Nicole J. de Voog, Ping-Lin Li, Guo-Qiang Li

**Affiliations:** 1 Key Laboratory of Marine Drugs, Chinese Ministry of Education, School of Medicine and Pharmacy, Ocean University of China, Qingdao, 266003 Republic of China; 2 Laboratory of Marine Drugs and Biological Products, National Laboratory for Marine Science and Technology, Qingdao, 266235 Republic of China; 3 College of Chemistry and Chemical Engineering, Ocean University of China, Qingdao, 266100 Republic of China; 4 Institute of Chronic Diseases, Qingdao University, Qingdao, 266071 Republic of China; 5 National Museum of Natural History, 2300 RA Leiden, The Netherlands

## Abstract

Seven pairs of new oxygenated aplysinopsin-type enantiomers, (+)- and (−)-oxoaplysinopsins A‒G (**1**‒**7**), two new bromotyrosine-derived alkaloids, subereamollines C and D (**18** and **19**), together with ten known compounds (**8**‒**17**) were isolated from the Xisha Islands sponge *Fascaplysinopsis reticulata*. The planar structures were determined by extensive NMR and MS spectroscopic data. Each of the optically pure enantiomers was achieved by chiral HPLC separation. The absolute configurations were assigned by the quantum chemical calculation methods. Compound **19** showed cytotoxicity against Jurkat cell lines with IC_50_ value of 0.88 μM. Compounds **2**, **16** and **17** showed tyrosine phosphatase 1B (PTP1B) inhibition activity with IC_50_ value ranging from 7.67 to 26.5 μM, stronger than the positive control of acarbose and 1-deoxynojirimycin. A structural activity relationship for the aplysinopsin-type enantiomers were observed in PTP1B inhibition activity of **2** and cytotoxicity of **3** that the dextrorotary (+)**-2** and (+)**-3** showed stronger activity than the levorotary (−)**-2** and (−)**-3**.

## Introduction

Sponge of the genus *Fascaplysinopsis* is special in taxonomy that it is a rare monotypic sponge genus containing only one species *F. reticulata* which was originally identified as *Aplysinopsis reticulata* in 1912 and revised to *F. reticulata* in 1980^1^. *Fascaplysinopsis* is important resource of marine natural products^2–6^ that there were more than 60 compounds been isolated since the typical aplysinopsin firstly found in 1977^7–12^. Aplysinopsins are a class of indole alkaloids structurally architected by an indole and an imidazole moieties which showed rich structural diversity characterized by N^3^′-methylaplysinopsin^13^, brominated derivatives^14^, oxoforms^15^, and dimmeric forms^16^. Up to date, there are totally 30 aplysinopsins isolated, showing a diverse origin including sponge genera of *Dercitus*^17^, *Smenospongia*^18^, *Verongula*^19^ *et al*. as well as corals of *Tubastrea*^20^, *Dendrophyllia*^21^ and mollusc of *Phestilla*^22^. Aplysinopsins have shown pharmaceutical significance with neuromodulation, antineoplastic, antiplasmodial, and antimicrobial activities^11^. The geographic locations of these aplysinopsin-origin organisms are mostly focusing on Caribbean, Mediterranean Sea, as well as Indo-Pacific region.

**Figure 1:**
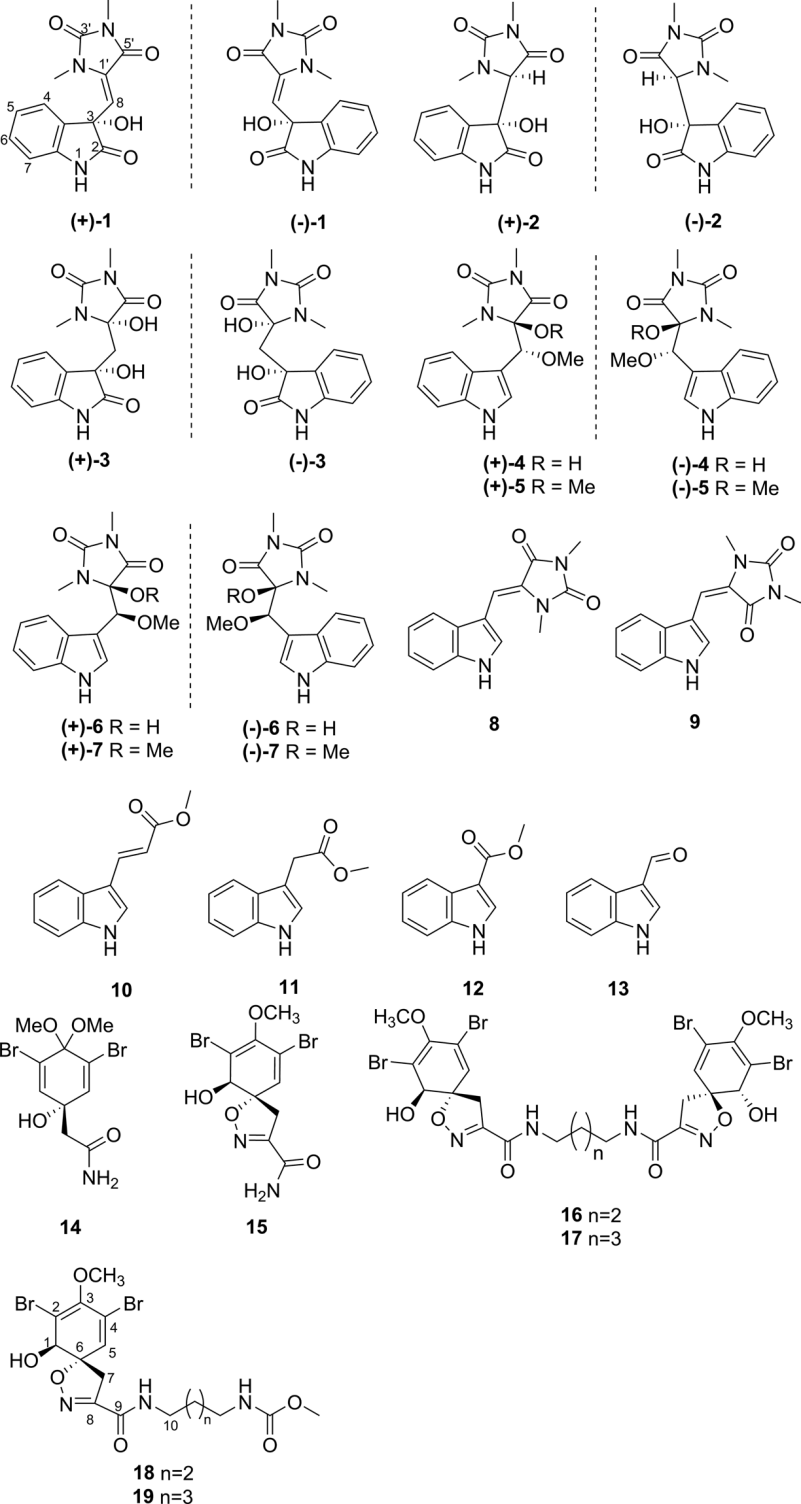
Structures of **1**–**19** from sponge *Fascaplysinopsis reticulata*.

Our first investigation on XiSha Islands (Paracel Islands) *F. reticulata* has yielded (+)- and (−)-spiroreticulatine, a pair of unusual spiro bisheterocyclic quinoline-imidazole alkaloids in previous study^23^. A further study on this species yielded eighteen compounds, including seven pairs of new oxygenated aplysinopsin-type enantiomers, (+)- and (−)-oxoaplysinopsins A‒G (**1**‒**7**), two new bromotyrosine-derived alkaloids, subereamollines C and D (**18** and **19**), together with ten known related compounds (**8**‒**17**) (Fig. 1). The enantiomers were purified by chiral HPLC method. And all the absolute configurations were determined by comparing experimental and calculated ECD using quantum chemical calculation method. The cytotoxicity against selected tumor cell lines and tyrosine phosphatase 1B (PTP1B) inhibition activity of the isolates were assayed. Herein we report the isolation, structural elucidation, and biological activities of these compounds.

## Results and Discussion

Oxoaplysinopsin A (**1**) was obtained as yellow, amorphous powder, possessing a molecular formula of C_14_H_13_N_3_O_4_ with 10 degrees of unsaturation as informed by its HRESIMS data. The IR spectrum suggested the presence of carbonyl (1702 cm^−1^) group and aromatic moiety (1603, 1497, 1449 cm^−1^). The ^1^H NMR spectrum of **1** (Table 1) displayed indole signals characterized by an imino proton (*δ*_H_ 10.30, br s) and four aromatic protons in an ABCD coupling system (*δ*_H_ 6.80, d, *J* = 7.6 Hz; 6.88, dd, *J* = 7.5, 7.5 Hz; 7.16, d, *J* = 6.7 Hz; and 7.18, dd, *J* = 7.7, 6.2 Hz)^18^. Besieds, an olefinic proton (*δ*_H_ 5.80, s), and two nitrogen-bearing methyl protons (*δ*_H_ 2.83, 3.04), as well as a hydroxyl proton (*δ*_H_ 6.85, s) were observed. The ^13^C NMR and DEPT spectra of **1** (Table 1) exhibited totally 14 carbon resonances which were divided into two methyls (*δ*_C_ 26.1, 24.4), five olefinic methines (*δ*_C_ 109.4, 117.5, 121.4, 123.9, and 129.2), and seven quaternary carbons including one oxygen-bearing (*δ*_C_ 74.9), three olefinic (*δ*_C_ 130.4, 132.1, 143.0), and three amide carbonyls (*δ*_C_ 153.3, 161.3, and 176.2). The 1D NMR data suggested compound **1** as aplysinopsin analogue of 3′-deimino-3′-oxoaplysinopsin^13^.

**Table 1:** ^1^H (500 MHz) and ^13^C NMR (125 MHz) Data for 1–3 and 6 in DMSO-d_6_.

No	1		2		3	
	*δ*_*C*_, type	*δ*_H_ (*J* in Hz)	*δ*_*C*_, type	*δ*_H_ (*J* in Hz)	*δ*_*C*_, type	*δ*_H_ (*J* in Hz)
1		10.30, br s		10.43, br s		10.30, br s
2	176.2, C		175.3, C		177.9, C	
3	74.9, C		76.2, C		72.3, C	
3a	132.1, C		126.9, C		128.6, C	
4	123.9, CH	7.16, d, 1 H (6.7)	124.1, CH	7.08, d, 1 H (7.5)	126.3, CH	7.04, d, 1 H (7.4)
5	121.4, CH	6.88, dd, 1 H (7.5, 7.5)	121.5, CH	6.90, dd, 1 H (7.5, 7.5)	120.9, CH	6.88, dd, 1 H (7.5, 7.5)
6	129.2, CH	7.18, dd, 1 H (7.7, 6.2)	130.1, CH	7.22, dd, 1 H (7.7, 7.7)	129.4, CH	7.13, dd, 1 H (7.5, 7.6)
7	109.4, CH	6.80, d, 1 H (7.6)	109.9, CH	6.78, d, 1 H (7.7)	109.2, CH	6.70, d, 1 H (7.5)
7a	143.0, C		142.6, C		142.3, C	
8	117.5, CH	5.80, s, 1 H			41.7, CH_2_	2.57, s, 2 H
1′	130.4, C		66.1, CH	4.40, s, 1 H	83.4, C	
3′	153.3, C		157.2, C		155.6, C	
5′	161.3, C		168.6, C		172.3, C	
2′-NCH_3_	26.1, CH_3_	3.04, s, 3 H	31.3, CH_3_	3.14, s, 3 H	24.4, CH_3_	2.55, s, 3 H
4′-NCH_3_	24.4, CH_3_	2.83, s, 3 H	24.2, CH_3_	2.50, s, 3 H	23.7, CH_3_	2.19, s, 3 H
3-OH		6.85, s		6.62, s		6.70, s
1′-OH						6.00, s

**Figure 2:**
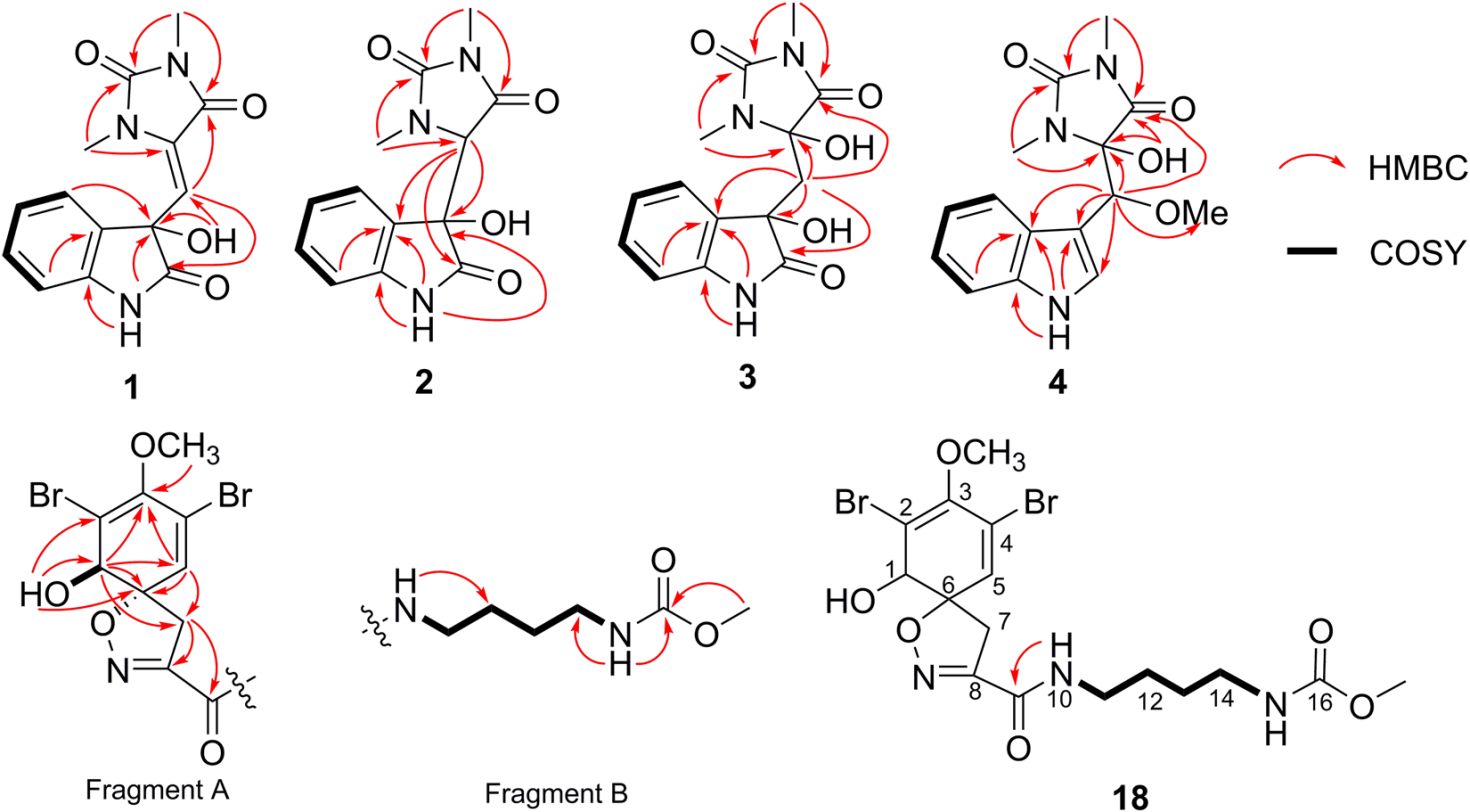
Key COSY and HMBC correlations in compounds **1**–**4**, and **18**.

A consecutive ^1^H-^1^H COSY correlation from H-4 to H-7, together with the HMBC correlations from H-4 to C-3 (*δ*_C_ 74.9), and from 1-NH to C-3, C-3a (*δ*_C_ 132.1) and C-7a (*δ*_C_ 143.0) confirmed the indole moiety (Fig. 2). The HMBC correlations from NMe (*δ*_H_ 3.04) to C-1′ (*δ*_C_ 130.4) and C-3′ (*δ*_C_ 153.3), from the other NMe (*δ*_H_ 2.83) to C-3′ and C-5′ (*δ*_C_ 161.3) constructed the 1, 3-dimethyl-imidazolidin-2, 4-dione moiety. The residual hydroxyl group was located at C-3 evident from HMBC correlations from the hydroxyl proton to C-2 (*δ*_C_ 176.2), C-3, C-3a and C-8. The additional HMBC correlations from H-8 (*δ*_H_ 5.80) to C-2, C-3, C-1′ and C-5′ indicated that the imidazolidin and indole moieties were connected through sp2 methine C-8. Thus, the planar structure of **1** was elucidated as shown.

Oxoaplysinopsin B (**2**) was isolated as a yellow, amorphous powder. HRESIMS implied its molecular formula of C_13_H_13_N_3_O_4_, 12 atomic mass less than that of compound **1**. The spectroscopic data of **2** were similar to those of compound **1** except for the disappeared methine signal of CH-8 and extra N-bearing CH-1′ (*δ*_H_ 4.40 and *δ*_C_ 66.1). HMBC correlations from H-1′ (*δ*_H_ 4.40) to C-2 (*δ*_C_ 175.3), C-3 (*δ*_C_ 76.2), C-3a (*δ*_C_ 126.9), C-3′ (*δ*_C_ 157.2), and C-5′ (*δ*_C_ 121.5), and from NMe (*δ*_H_ 3.14) to C-1′ (Fig. 2), suggested a direct connection of 2-oxoindole and 1, 3-dimethylimidazolidin-2, 4-dione moieties in compound **2**.

Oxoaplysinopsin C (**3**) had molecular formula of C_14_H_15_N_3_O_5_ by HRESIMS data. The main difference of 1D NMR between compounds **3** and **1** was that a methylene signals (*δ*_H_ 2.57 and *δ*_C_ 41.7) was observed in **3** intend of olefinic methine in **1**. HMBC correlations from the methylene H_2_-8 (*δ*_H_ 2.57) to C-2 (*δ*_C_ 177.9), C-3 (*δ*_C_ 72.3), C-3a (*δ*_C_ 128.6), C-1′ (*δ*_C_ 83.4), and C-5′ (*δ*_C_ 172.3) (Fig. 2), suggested that compound **3** was a hydroxylated product of **1**.

Oxoaplysinopsins D (**4**) had molecular formula of C_15_H_17_N_3_O_4_ from HRESIMS data, 14 atomic mass more than that of **3**. Analysis of its ^1^H and ^13^C NMR spectra (Table 2) disclosed that **4** was very similar to **3** except for an extra methoxyl group (*δ*_H_/*δ*_C_ 3.20/57.3) and olefinic CH (*δ*_H_/*δ*_C_ 7.15/124.6) instead of the carbonyl group of C-2 in **3**. HMBC correlations (Fig. 2) from OMe group to C-8 (*δ*_C_ 78.7), and from H-8 (*δ*_H_ 4.88) to C-2 (*δ*_C_ 124.6), C-3 (*δ*_C_ 108.5), C-3a (*δ*_C_ 127.0), C-1′ (*δ*_C_ 87.4), C-5′ (*δ*_C_ 171.8), and OMe indicated that the structure of compound **4** was shown as depicted. Oxoaplysinopsins E (**6**) had the same molecular formula with that of **4**. And their 1D NMR data were very similar except for slight differences around the chiral center of C-8 and C-1′. COSY and HMBC data of **6** indicated that **6** had the same planar structure with **4**, indicating that they were epimers.

**Table 2:** ^1^H (500 MHz) and ^13^C NMR (125 MHz) Data for 4–5 in DMSO-d_6_.

No	4		5		6		7	
	*δ*_*C*_, type	*δ*_H_ (*J* in Hz)	*δ*_*C*_, type	*δ*_H_ (*J* in Hz)	*δ*_*C*_, type	*δ*_H_ (*J* in Hz)	*δ*_*C*_, type	*δ*_H_ (*J* in Hz)
1		11.13, br s		11.18, br s		11.17, br s		11.20, br s
2	124.6, CH	7.15, d, 1 H (2.3)	124.7, CH	7.17, d, 1 H (2.2)	125.3, CH	7.28, s, 1 H	125.8, C	7.29, s
3	108.5, C		107.9, C		108.3, C		108.4, C	
3a	127.0, C		127.0, C		126.5, C		126.8, C	
4	119.0, CH	7.54, d, 1 H (8.0)	118.9, CH	7.53, d, 1 H (8.0)	120.2, CH	7.62, d, 1 H (8.0)	120.4, CH	7.58, d, 1 H (8.0)
5	118.7, CH	6.97, dd, 1 H (8.0, 8.0)	118.8, CH	6.97, dd, 1 H (7.8, 7.2)	118.9, CH	6.98, dd, 1 H (7.5, 7.5)	119.5, CH	7.00, dd, 1 H (7.3, 7.7)
6	120.9, CH	7.05, dd, 1 H (7.2, 7.8)	120.9, CH	7.06, dd, 1 H (7.2, 7.9)	121.0, CH	7.07, dd, 1 H (7.3, 7.7)	121.5, CH	7.08, dd, 1 H (7.2, 7.9)
7	111.4, CH	7.34, d, 1 H (8.1)	111.4, CH	7.35, d, 1 H (8.1)	111.5, CH	7.36, d, 1 H (8.1)	112.1, CH	7.37, d, 1 H (8.1)
7a	135.7, C		135.7, C		136.3, C		136.8, C	
8	78.7, CH	4.88, s, 1 H	77.9, CH	4.94, s, 1 H	79.2, CH	4.78, s, 1 H	79.4, CH	4.85, s, 1 H
1′	87.4, C		92.4, C		86.8, C		92.3, C	
3′	155.6, C		155.6, C		156.6, C		156.6, C	
5′	171.8, C		169.2, C		173.7, C		171.4, C	
2′-NCH_3_	25.5, CH_3_	3.00, s, 3 H	25.6, CH_3_	3.02, s, 3 H	25.6, CH_3_	2.22, s, 3 H	26.1, CH_3_	2.33, s, 3 H
4′-NCH_3_	23.9, CH_3_	2.54, s, 3 H	23.9, CH_3_	2.60, s, 3 H	24.2, CH_3_	2.86, s, 3 H	24.7, CH_3_	2.90, s, 3 H
8-OCH_3_	57.3, CH_3_	3.20, s, 3 H	57.1, CH_3_	3.19, s, 3 H	57.0, CH_3_	3.18, s, 3 H	57.6, CH_3_	3.19, s, 3 H
1′-OH		6.96, s				6.94, s		
1′-OCH_3_			51.3, CH_3_	3.03, s, 3 H			51.8, CH_3_	3.01, s, 3 H

Oxoaplysinopsin F and G (**5** and **7**) had the same molecular of C_16_H_19_N_3_O_4_, 14 atomic mass more than those of compounds **4** and **6**. An extra methoxyl group at *δ*_H_/*δ*_C_ 3.03/51.3 in **5** and *δ*_H_/*δ*_C_ 3.01/51.8 in **7** suggested compounds **5** and **7** as 1′-methylated product of **4** and **6**, which was confirmed by HMBC correlations from OMe to C-1′. And the empimeric relationship between **6** and **7** were also shown by the slight differences around chiral centers of C-8 and C-1′.

**Figure 3:**
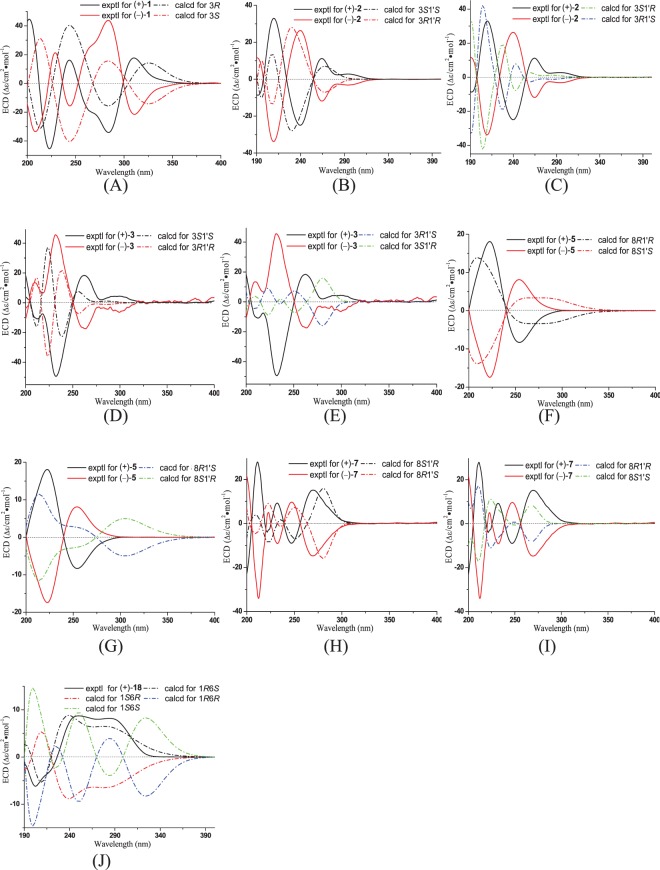
(**A**) Experimental ECD spectra of (+)- and (−)**-1** in MeOH and calculated ECD spectra of (3*R*)**-1** and (3*S*)-1 (half width 0.3; UV-shift 5 nm). (**B**) Experimental ECD spectra of (+)- and (−)**-2** in MeOH and calculated ECD spectra of (3*R*, 1′*R*)**-2** and (3*S*, 1′*S*)**-2** (half width 0.24; UV-shift -18 nm).(**C**) Experimental ECD spectra of (+)- and (−**)-2** in MeOH and calculated ECD spectra of (3*R*, 1′*S*)**-2** and (3*S*, 1′*R*)**-2** (half width 0.24; UV-shift 0 nm). (**D**) Experimental ECD spectra of (+)- and (−)**-3** in MeOH and calculated ECD spectra of (3*R*, 1′*R*)**-3** and (3*S*, 1′*S*)**-3** (half width 0.2; UV-shift 10 nm). (**E**) Experimental ECD spectra of (+)- and (−)**-3** in MeOH and calculated ECD spectra of (3*R*, 1′*S*)**-3** and (3*S*, 1′*R*)**-3** (half width 0.2; UV-shift 10 nm). (**F**) Experimental ECD spectra of (+)- and (−)**-5** in MeOH and calculated ECD spectra of (8*R*, 1′*R*)**-5** and (8*S*, 1′*S*)**-5** (half width 0.44; UV-shift 0 nm). (**G**) Experimental ECD spectra of (+)- and (−)**-5** in MeOH and calculated ECD spectra of (8*R*, 1′*S*)**-5** and (8*S*, 1′*R*)**-5** (half width 0.44; UV-shift 0 nm).(**H**) Experimental ECD spectra of (+)- and (−)**-7** in MeOH and calculated ECD spectra of (8*S*, 1′*R*)**-7** and (8*R*, 1′*S*)**-7** (half width 0.16; UV-shift -20 nm). (**I**) Experimental ECD spectra of (+)- and (−)**-7** in MeOH and calculated ECD spectra of (8*R*, 1′*R*)**-7** and (8*S*, 1′*S*)**-7** (half width 0.16; UV-shift -20 nm). (**J**) Experimental ECD spectra of **18** in MeOH and calculated ECD spectra of (1*R*, 6′*S*)**-18a**,(1*S*, 6′*R*)-**18a**,(1*R*, 6′*R*)-**18a**, and (1*S*, 6′*S*)-**18a** (half width 0.5; UV-shift: -13 nm).

Compounds **1**–**7** were initially obtained as optical inactivity compounds, indicating that they were enantiomers. Chiral HPLC purification afforded seven pairs of enantiomers, (+)- and (−)**-1**‒(+)- and (−)**-7**, in a ratio of almost 1:1 (Supporting Information). The opposite optical rotation values and mirror ECD spectra for the dextrorotary and levorotary enantiomers were observed (Fig. 3). To determine their absolute configurations, ECD calculation for respective (+)- and (−)-isomers of compounds **1**–**7** were performed by the TDDFT/ECD method at RB3LYP/DGDZVP level (Supporting Information)^23,24^. The experimental ECD of (+**)-1** exhibited two strong positive Cotton effect (CEs) at 242.5 and 309.0 nm and two strong negative CEs at 222.5 and 284.5 nm, in agreement with the calculated ECD spectrum for 3*R* configuration (Fig. 3), and showed mirror-like relationship with calculated and experimental ECD spectra for 3*S* configuration. Therefore, 3*R* and 3*S* were finally assigned for (+)**-1** and (−)**-1**, respectively. Similarly, the absolute configurations of 3*S*, 1′*S* for (+)**-2**, 3*R*, 1′*R* for (−)**-2**, 3*R*, 1′*R* for (+)**-3**, 3*S*, 1′*S* for (−)**-3**, 8*R*, 1′*R* for (+)**-5**, 8*S*, 1′*S* for (−)**-5**, 8*S*, 1′*R* for (+)**-7**, and 8*R*, 1′*S* for (−)**-7** were assigned (Fig. 3). And compounds (+)**-4** and(−)**-4**, and (+)**-6** and (−)**-6** showed similar Cotton effects as respective (+)**-5** and(−)**-5**, and (+)**-7** and (−)**-7** (Supporting Information), indicating that they possessed the same absolute configuration.

Subereamolline C (**18**) was isolated as a white, amorphous powder. The HRESIMS spectrum showed three quasi-molecular ion peaks (*m*/*z* 509.9869, 511.9846, 513.9825) in a ratio of 1:2:1, indicating that compound **18** was a dibrominated product possessing molecular formula of C_16_H_21_Br_2_N_3_O_6_ with 7 degrees of unsaturation. ^13^C NMR and DEPT spectra of **18** (Table 3) exhibited a total of 16 carbon resonances which were divided into two methoxys (*δ*_C_ 59.6, 51.1), five methylenes (*δ*_C_ 39.9, 39.4, 38.5, 26.9 and 26.2), two methines (*δ*_C_ 131.3, 73.6) and seven quaternary carbons (*δ*_C_ 158.8, 156.7, 154.5, 147.1, 120.8, 113.1, 90.1), which was similar with those of brominated phenolic compound subereaphenol A isolated from a methanol extract of Red Sea Sponge *Suberea mollis*^25^, except for the absence of oxygenated methylene in **18**. HMBC correlation from 16-OCH_3_ (*δ*_H_ 3.50) to C-16 (*δ*_C_ 156.7) suggested the terminal methyl ester in **18** rather than the ethyl ester in subereaphenol A. Detailed analysis of COSY and HMBC data (Fig. 2) allowed the planar structure of **18** determined. The absolute configuration of **18** was suggested to be the same as subereaphenol A by comparing their optical rotation values, which was confirmed by theoretical ECD calculation of all the four candidates of 1*R*, 6*S* and 1*R*, 6*R* and their enantiomers of **18a** (Supporting Information)^26^. The calculated CEs of (1*R*, 6*S*)-**18a** matched well with the experimental CEs of **18** and were image symmetrical to (1*S*, 6*R*)-**18a**, while they were totally different from those of (1*R*, 6*R*)-**18a** and (1*S*, 6*S*)-**18a** (Fig. 3). That allowed the absolute configuration of **18** determined as 1*R*, 6*S*.

**Table 3:** ^1^H (500 MHz) and ^13^C NMR (125 MHz) Data for 18 and 19 in DMSO-*d*_6_.

No	18		19	
	*δ*_*C*_, type	*δ*_*H*_ (*J* in Hz)	*δ*_*C*_, type	*δ*_*H*_ (*J* in Hz)
1	73.6, CH	3.91, d, 1 H (5.6)	73.6, CH	3.91, d, 1 H (5.6)
2	113.1, C		113.1, C	
3	147.1, C		147.1, C	
4	120.8, C		120.8, C	
5	131.3, CH	6.58, s, 1 H	131.3, CH	6.59, s, 1 H
6	90.1, C		90.1, C	
7	39.4, CH_2_	3.61, d, 1 H (18.2); 3.20 d, 1 H (18.2)	39.4, CH_2_	3.61, d, 1 H (18.3); 3.21, d, 1 H (18.3)
8	154.5, C		154.1, C	
9	158.8, C		158.8, C	
10		8.49, t (5.7, 5.8)		8.52, t (5.5, 5.6)
11	38.5, CH_2_	3.13, m, 2 H	38.7, CH_2_	3.11, m, 2 H
12	26.2, CH_2_	1.43, m, 2 H	29.1, CH_2_	1.44, m, 2 H
13	26.9, CH_2_	1.38, m, 2 H	23.6, CH_2_	1.23, m, 2 H
14	39.9, CH_2_	2.96, m, 2 H	28.5, CH_2_	1.38, m, 2 H
15		7.08, t (4.9, 4.9)	40.1, CH_2_	2.94, m, 2 H
16	156.7, C			7.11, t (5.5, 5.5)
17			156.7, C	
1-OH		6.36, d (7.1)		6.39, d (7.1)
3-OCH_3_	59.6, CH_3_	3.65, s, 3 H	59.6, CH_3_	3.64, s, 3 H
16-OCH_3_	51.1, CH_3_	3.50, s, 3 H		
17-OCH_3_			51.5, CH_3_	3.50, s, 3 H

Subereamolline D (**19**) possessed the molecular formula of C_17_H_23_Br_2_N_3_O_6_ by HRESIMS, 14 atomic mass more than that of compound **18**. The 1D NMR data of compound **19** were very similar with those of **18** except for an extra CH_2_ group (*δ*_H_ 1.23, *δ*_C_ 23.6) in **19**. ^1^H-^1^H COSY correlations of NH-10/H_2_-11/H_2_-12/H_2_-13/H_2_-14/NH-15, together with HMBC correlations from OMe, NH-15 and H_2_-14 to C-16 suggested methyl (5-aminopentyl)carbamate moiety in **19** rather than the methyl (4-aminobutyl)carbamate moiety in **18**. And the absolute configuration of **19** was determined the same as **18** by comparing their optical rotation value and NMR data.

On comparison of the physical and spectroscopic data with published values, the known compounds were identified as (*Z*)-3′-deimino-3′-oxoaplysinopsin (**8**)^15^, (*E*)-3′-deimino-3′-oxoaplysinopsin (**9**)^15^, (*E*)-3-indolylpropenoate (**10**)^27^, indolyl-3-acetic acid methyl ester (**11**)^28^, 3-methoxycarbonylindole (**12**)^29^, 3-formylindole (**13**)^30^, 3, 5-dibromoverongiaquinol dimethyl ketal (**14**)^31^, purealidin R (**15**)^31^, aerothionin (**16**)^32^, and homoaerothionin (**17**)^25^.

The cytotoxicity against human lung carcinoma (A549), human cervical cancer (HeLa), human leukemia (K562), and human T-cell leukemia (Jurkat) cell lines, as well as tyrosine phosphatase 1B (PTP1B) inhibition activity of the isolates were assayed. Compound **19** showed cytotoxicity against Jurkat cell lines with IC_50_ value of 0.88 μM, comparable to the positive control of Doxorubicin (IC_50_ = 0.442 μM). Compounds **16** and **17** showed tyrosine phosphatase 1B (PTP1B) inhibition activity with IC_50_ value of 7.67 and 11.25 μM, respectively, stronger than the positive control of acarbose (457 μg/mL) and 1-deoxynojirimycin (31.29 μg/mL). The racemate (±)**-2** (20.8 μM) and optically pure (+)**-2** (18.3 μM) and (−)**-2** (26.5 μM) showed a little high IC_50_ value in inhibition of PTP1B but were still stronger than the positive controls. The previous study showed a preliminary structure-activity relationship of the significant role of the substations in the benzene and imidazole moieties^11^. The present study suggested oxygen pattern rather than methylation as another contribution for bioactivity of aplysinopsins. In addition, the dextrorotary (+)**-2** showed stronger activity than the levorotary enantiomers (−)**-2**. This firstly encountered structural activity relationship for the aplysinopsin-type enantiomers were also observed in cytotoxicity assay of compound **3** against HeLa cell lines that the dextrorotary (+)**-3** had IC_50_ value of 27.0 μM, two fold of the levorotary enantiomers (−)**-3** with IC_50_ value of 61.6 μM.

In summary, nine aplysinopsin-type alkaloids, nine indole analogues, and six bromotyrosine-derived alkaloids were isolated from the Xisha Islands sponge *Fascaplysinopsis reticulata*. Seven new aplysinopsin-type alkaloids (**1**‒**7**) and two new bromotyrosine-derived alkaloids (**18** and **19**) were identified by comprehensive using of NMR, MS, and quantum chemical calculation methods. Although the first and the only one aplysinopsin type indole alkaloid was isolated from *F.reticulata*, in previous study the dimmeric indole alkaloids fascaplysin were suggested as the main metabolites in *Fascaplysinopsis* sponge^7–10^. Furthermore, the konwn oxygenated aplysinopsin mainly focused on 3′-oxoaplysinopsin including 3′-deimino-3′-oxoaplysinopsin, and 3′-deimino-2′, 4′-bis(demethyl)-3′-oxoaplysinopsin, as well as their brominated analogues^7–10^. In the present study, series of 3, 8-oxoaplysinopsins (**1**‒**7**) were firstly encountered in *F. reticulata*. Besides, bromotyrosine-derived alkaloids were reported previously as a kind of characteristic structures solely isolated from the sponge of Verongida order^8,33–35^, and were recently obtained through culturing sponge *Arenosclera brasiliensis* derived bacterium *Pseudovibrio denitrificans*^26^. And there were totally no more than 30 bromotyrosine-derived alkaloids found in nature^8^. We obtained six bromotyrosine-derived alkaloids (**14**‒**19**) from the sponge *F. reticulata*. The result indicated a significant chemical diversity in *F. reticulata* which is possibly attributed to the special geography of XiSha Islands.

The series of aplysinopsin enantiomers inspired again the biosynthetic enantiodivergence evidence in natural^36,37^. Sponges were suggested to be potentially biosynthetic enantiodivergence, since more and more enantiomers such as purealidin R from *Psammaplysilla*^38^, plakortolides H and I from *Plakortis*^39^, and strongylodiols A–C from *Petrosia* (*Strongylophora*)^40^, as well as corynechromones from Sponge-Derived Strain of the Fungus *Corynespora cassiicola*^41^, and DD- and LL-diketopiperazines from respective *Calyx* sponge derived *Pecten maximus* and *Isodictya* sponge derived *Pseudomonas aeruginosa*^42,43^, were isolated. In present study, the firstly encountered versatile enantiomeric 3-oxoaplysinopsin (**1**–**3**), 1′-oxoaplysinopsin (**3**–**7**), or 8-oxoaplysinopsin (**4**–**7**) showed remarkable stereochemistry diversity in oxoaplysinopsins which are possibly originated from (*Z*)- or (*E*)-3′-deimino-3′-oxoaplysinopsin (**8**/**9**). That indicated a potential enantiodivergence in *F. reticulata* which could be unveiled through the biosynthesis and symbiot study in future.

## Experimental Section

### General Experimental Procedures

Optical rotations were measured on a JASCO P-1020 digital polarimeter. UV spectra were recorded on a Beckman DU640 spectrophotometer. CD spectra were obtained on a JASCO J-810 spectropolarimeter. IR spectra were taken on a Nicolet NEXUS 470 spectrophotometer in KBr discs. NMR spectra were measured by Bruker AVANCE III 600 spectrometers. The 2.5000 ppm and 39.50 ppm resonances of DMSO were used as internal references for ^1^H and ^13^C NMR spectra, respectively. HRESIMS spectra were measured on a Micromass Q-Tof Ultima GLOBAL GAA076LC and Thermo Scientific LTQ orbitrap XL mass spectrometers. Semi-preparative HPLC utilized an ODS column [YYMC-Pack ODS-A, 100 × 250 mm, 5 µm, 1.5 mL/min]. Chiral HPLC utilized chiral analytical columns [CHIRALPAK IC column (4.6 × 250 mm, 5 µm)]. Silica gel (200–300 mesh, Qingdao, China) was used for column chromatography, and precoated Silica gel plates (GF254, Qingdao, China) were used for TLC, and spots visualized by heating SiO_2_ plates sprayed with 5% H_2_SO_4_ in EtOH.

### Animal Material

The marine sponge *Fascaplysinopsis reticulata* was collected from Xisha Island of South China Sea in December 2009, and was frozen immediately after collection. The specimen was identified by Nicole J. de Voogd, National Museum of Natural History, Leiden, The Netherlands. The voucher specimen (No. XS-2009-29) was deposited at State Key Laboratory of Marine Drugs, Ocean University of China, P. R. China.

### Extraction and isolation

A frozen specimen of *Fascaplysinopsis reticulata* (1.6 kg, wet weight) was homogenized and then extracted with MeOH four times (3 days each time) at RT. The combined solutions were concentrated in vacuo and was then subsequently desalted by redissolving with MeOH to yield a residue (69 g). The crude extract was subjected to silica gel vacuum liquid chromatography (VLC), eluting with a gradient of petroleum/acetone (from 10:0 to 1:1, v:v) and subsequently CH_2_Cl_2_/MeOH (from 20:1 to 0:1, v-v) to obtain eight fractions (Fr.1–Fr.10). Fr.3 (6.3 g) was then subjected to a silica gel CC (petroleum/ethyl acetate, from 50:1 to 1:1, v:v) to give twelve subfractions Fr.3-1–Fr.3-12. Fr.3-12 (449.0 mg) was also subjected to a silica gel CC (petroleum/acetone, from 10:1 to 1:1, v:v) to give eight subfractions Fr.3-12-1–Fr.3-12-8. Fr.3-12-7 (53 mg) was purified by semi-preparative HPLC (ODS, 5 µm, 250 × 10 mm; MeOH/H_2_O, 40:60, v/v; 1.5 mL/min) to afford **6** (6.1 mg) and **7** (6.0 mg). Fr.3-12-8 (37 mg) was also purified by semi-preparative HPLC (ODS, 5 µm, 250 × 10 mm; MeOH/H_2_O, 40:60, v/v; 1.5 mL/min) to afford **1** (1.9 mg) and **2** (1.8 mg). Fr.4 (7.0 g) was subjected to a silica gel CC (petroleum/ethyl acetate, from 20:1 to 1:1, v:v) to give twelve fractions Fr.4-1–Fr.4-12. Fr.4-10 (296.8 mg) was purified by semi-preparative HPLC (ODS, 5 µm, 250 × 10 mm; MeOH/H_2_O, 55:45, v/v; 1.5 mL/min) to afford **4** (8.8 mg), **5** (12.6 mg), **11** (5.5 mg), **12** (6.0 mg) and **13** (2.8 mg). Fr.5 (5.5 g) was subjected to a silica gel CC (petroleum/ethyl acetate, from 10:1 to 1:1, v:v) to give twelve fractions Fr.5-1–Fr.5-10. Fr.5-8 (693.7 mg) was also subjected to a silica gel CC (petroleum/acetone, from 20:1 to 1:1, v:v) to give seven fractions Fr.5-8-1–Fr.5-8-7. Fr.5-8-5 (77 mg) was purified by semi-preparative HPLC (ODS, 5 µm, 250 × 10 mm; MeOH/H_2_O, 30:70, v/v; 1.5 mL/min) to afford **3** (4.0 mg). Fr.6 (20 g) was subjected to a silica gel CC (petroleum/ethyl acetate, from 10:1 to 1:1, v:v) to give six fractions Fr.6-1–Fr.6-6. Fr.6-2 (5.2 g) was also subjected to a silica gel CC (petroleum/acetone, from 20:1 to 1:1, v:v) to give five fractions Fr.6-2-1–Fr.6-2-5. Fr.6-2-4 (456 mg) was purified by semi-preparative HPLC (ODS, 5 µm, 250 × 10 mm; MeOH/H_2_O, 40:70, v/v; 1.5 mL/min) to afford **8** (4.0 mg), **9** (3.0 mg) and **10** (6.3 mg). Fr.6-2-5 (875 mg) was purified by semi-preparative HPLC (ODS, 5 µm, 250 × 10 mm; MeOH/H_2_O, 30:70, v/v; 1.5 mL/min) to afford **14** (19.0 mg), **15** (38.0 mg), **16** (20.0 mg), **17** (36.3 mg), **18** (11.2 mg) and **19** (10.0 mg).

### Cytotoxicity assay

The cytotoxicity assay of the isolated compounds were evaluated against human lung carcinoma (A549), human cervical cancer (HeLa), human leukemia (K562), and human T-cell leukemia (Jurkat) cell lines using the MTT method with Doxorubicin as the positive control^44^.

### Tyrosine phosphatase 1B (PTP1B) inhibition activity assay

The antidiabetic activities were evaluated on the inhibition of tyrosine phosphatase 1B (PTP1B) protein which is recently of substantial interest for the treatment of type-2 diabetes mellitus^45,46^. 1-Deoxynojirimycin and Acarbose were used as positive controls.

#### Oxoaplysinopsin A (1)

yellow, amorphous powder; ^1^H and ^13^C NMR data, see Table 1; UV (MeOH) *λ*_max_ (log *ε*): 213 (3.74), 237 (3.87), 295 (3.90) nm; IR (KBr) *ν*_max_: 3360, 1702, 1603, 1497, 1449, 1163 cm^−1^; HRESIMS *m/z* 310.0795 [M + Na]^+^ (calcd for C_14_H_13_N_3_O_4_Na: 310.0798). (+)**-1**: [α]^20^_D_ = +28.3 (*c* 0.2, MeOH); (−)**-1**: [α]^20^_D_ = −27.9 (*c* 0.2, MeOH).

#### Oxoaplysinopsin B (2)

Yellow, amorphous powder; ^1^H and ^13^C NMR data, see Table 1; UV (MeOH) *λ*_max_ (log *ε*): 210 (4.02), 245 (4.01), 290 (3.99) nm; IR (KBr) *ν*_max_: 3619, 1754, 1680, 1497, 1449, 1007 cm^−1^; HRESIMS *m/z* 298.0790 [M + Na]^+^ (calcd for C_13_H_13_N_3_O_4_Na: 298.0798). (+)**-2**: [α]^20^_D_ = +4.5 (*c* 0.2, MeOH); (−)**-2**: [α]^20^_D_ = −3.3 (*c* 0.2, MeOH).

#### Oxoaplysinopsin C (3)

Yellow, amorphous powder; ^1^H and ^13^C NMR data, see Table 1; UV (MeOH) *λ*_max_ (log *ε*): 211 (3.89), 250 (3.85), 295 (3.92) nm; IR (KBr) *ν*_max_: 3357, 2980, 1708, 1653, 1601, 1524, 1019 cm^−1^; HRESIMS *m/z* 328.0906 [M + Na]^+^ (calcd for C_14_H_15_N_3_O_5_Na: 328.0904). (+)**-3**: [α]^20^_D_ = +5.6 (*c* 0.2, MeOH); (−)**-3**: [α]^20^_D_ = −5.1 (*c* 0.2, MeOH).

#### Oxoaplysinopsin D (4)

White, amorphous powder; ^1^H and ^13^C NMR data, see Table 2; UV (MeOH) *λ*_max_ (log *ε*): 212 (3.95), 278 (4.00) nm; IR (KBr) *ν*_max_: 3213, 1697, 1643, 1597, 1502, 1148 cm^−1^; HRESIMS *m/z* 326.1110 [M + Na]^+^ (calcd for C_15_H_17_N_3_O_4_Na: 326.1111). (+)*-*4: [α]^20^_D_ = +7.4 (*c* 0.2, MeOH); (−)***-*4**: [α]^20^_D_ = −6.8 (*c* 0.2, MeOH).

#### Oxoaplysinopsin E (5)

White, amorphous powder; ^1^H and ^13^C NMR data, see Table 2; UV (MeOH) *λ*_max_ (log *ε*): 215 (4.01), 270 (3.84) nm; IR (KBr) *ν*_max_: 1705, 1655, 1603, 1502, 1068 cm^−1^; HRESIMS *m/z* 340.1266 [M + Na]^+^ (calcd for C_16_H_19_N_3_O_4_Na: 340.1268). (+)**-5**: [α]^20^_D_ = +6.4 (*c* 0.2, MeOH); (−)**-5**: [α]^20^_D_ = −6.1 (*c* 0.2, MeOH).

#### Oxoaplysinopsin F (6)

White, amorphous powder; ^1^H and ^13^C NMR data, see Table 2; UV (MeOH) *λ*_max_ (log *ε*): 216 (3.95), 270 (3.85) nm; IR (KBr) *ν*_max_: 3314, 1698, 1650, 1591, 1502, 1106 cm^−1^; HRESIMS *m/z* 326.1110 [M + Na]^+^ (calcd for C_15_H_17_N_3_O_4_Na: 326.1111). (+)***-*6**: +10 (*c* 0.1, MeOH); (+)***-*6**: −10.4 (*c* 0.1, MeOH).

#### Oxoaplysinopsin G (7)

White, amorphous powder; ^1^H and ^13^C NMR data, see Table 2; UV (MeOH) *λ*_max_ (log *ε*): 215 (3.88), 270 (3.90) nm; IR (KBr) *ν*_max_: 1709, 1664, 1616, 1493, 1103 cm^−1^; HRESIMS *m/z* 340.1266 [M + Na]^+^ (calcd for C_16_H_19_N_3_O_4_Na: 340.1268). (+)**-7**: [α]^20^_D_ = +19.2 (*c* 0.2, MeOH); (+)**-7**: [α]^20^_D_ = −18.8 (*c* 0.2, MeOH).

#### Subereamolline C (18)

White, amorphous powder; [α]^20^_D_ = +189.1 (*c* 0.2, MeOH); ^1^H and ^13^C NMR data, see Table 3; UV (MeOH) *λ*_max_ (log *ε*): 280 (3.77), 230 (3.91), 205 (3.96) nm; HRESIMS *m/z* 511.9846 [M + H]^+^ (calcd for C_16_H_22_N_3_O_6_Br^81^Br: 511.9849).

#### Subereamolline D (19)

White, amorphous powder; [α]^20^_D_ = +145.0 (*c* 0.2, MeOH); ^1^H and ^13^C NMR data, see Table 3; UV (MeOH) *λ*_max_ (log *ε*): 281 (3.55), 236 (3.89), 211 (3.94) nm; HRESIMS *m/z* 526.0000 [M + H]^+^ (calcd for C_17_H_24_N_3_O_6_Br^81^Br: 526.0006).

## Supplementary information

Aplysinopsin-type and Bromotyrosine-derived Alkaloids from the South China Sea Sponge Fascaplysinopsis reticulata
