# Supplementary material for: Aplysinopsin-type and Bromotyrosine-derived Alkaloids from the South China Sea Sponge Fascaplysinopsis reticulata
Source: Sci Rep. 2019 Feb 19;9:2248. doi: 10.1038/s41598-019-38696-3 (PMC6381124; doi:10.1038/s41598-019-38696-3)
Supplement: Supplementary file 1 — Aplysinopsin-type and Bromotyrosine-derived Alkaloids from the South China Sea Sponge Fascaplysinopsis reticulata [file 41598_2019_38696_MOESM1_ESM.pdf]

Supporting Information

Aplysinopsin-type and Bromotyrosine-derived Alkaloids from the South China Sea Sponge *Fascaplysinopsis reticulata*

Qi Wang, Xu-Li Tang, Xiang-Chao Luo, Nicole J. de Voog, Ping-Lin Li, and Guo-Qiang Li.

Key Laboratory of Marine Drugs, Chinese Ministry of Education, School of Medicine and Pharmacy, Ocean University of China, Qingdao 266003, People's Republic of China.
Laboratory of Marine Drugs and Biological Products, National Laboratory for Marine Science and Technology, Qingdao 266235, Peoples' Republic of China.
College of Chemistry and Chemical Engineering, Ocean University of China, Qingdao 266100, People's Republic of China.
Institute of Chronic Diseases, Qingdao University, Qingdao 266071, People's Republic of China.
National Museum of Natural History, 2300 RA Leiden, The Netherlands.

Table of contents

Page

1. **Computational details** 4

2. **1D and 2D NMR, MS, and chiral HPLC spectra of the new compounds** 31

Figure S1. (+)-HRESIMS spectrum of **1**. 31
Figure S2. ^{1}H NMR (500 MHz, DMSO-d_6) spectrum of **1**. 31
Figure S3. ^{1}H NMR (500 MHz, DMSO-d_6) spectrum of **1**. 32
Figure S4. ^{13}C NMR (125 MHz, DMSO-d_6) spectrum of **1**. 32
Figure S5. HMQC spectrum of Compound **1**. 33
Figure S6. ^{1}H-^{1}H COSY spectrum of Compound **1**. 33
Figure S7. HMBC spectrum of Compound **1**. 34
Figure S8. (+)-HRESIMS spectrum of **2**. 34
Figure S9. ^{1}H NMR (500 MHz, DMSO-d_6) spectrum of **2**. 35
Figure S10. ^{1}H NMR (500 MHz, DMSO-d_6) spectrum of **2**. 35
Figure S11. ^{13}C NMR (125 MHz, DMSO-d_6) spectrum of **2**. 36
Figure S12. HMQC spectrum of Compound **2**. 36
Figure S13. ^{1}H-^{1}H COSY spectrum of Compound **2**. 37
Figure S14. HMBC spectrum of Compound **2**. 37
Figure S15. (+)-HRESIMS spectrum of **3**. 38
Figure S16. ^{1}H NMR (500 MHz, DMSO-d_6) spectrum of **3**. 38
Figure S17. ^{1}H NMR (500 MHz, DMSO-d_6) spectrum of **3**. 39
Figure S18. ^{13}C NMR (125 MHz, DMSO-d_6) spectrum of **3**. 39
Figure S19. HSQC spectrum of Compound **3**. 40
Figure S20. ^{1}H-^{1}H COSY spectrum of Compound **3**. 40
Figure S21. HMBC spectrum of Compound **3**. 41
Figure S22. (+)-HRESIMS spectrum of **4**. 41
Figure S23. ^{1}H NMR (500 MHz, DMSO-d_6) spectrum of **4**. 42
Figure S24. ^{1}H NMR (500 MHz, DMSO-d_6) spectrum of **4**. 42
Figure S25. ^{13}C NMR (125 MHz, DMSO-d_6) spectrum of **4**. 43
Figure S26. HSQC spectrum of Compound **4**. 43
Figure S27. HMBC spectrum of Compound **4**. 44
Figure S28. (+)-HRESIMS spectrum of **5**. 44
Figure S29. ^{1}H NMR (500 MHz, DMSO-d_6) spectrum of **5**. 45
Figure S30. ^{1}H NMR (500 MHz, DMSO-d_6) spectrum of **5**. 45
Figure S31. ^{13}C NMR (125 MHz, DMSO-d_6) spectrum of **5**. 46
Figure S32. HSQC spectrum of Compound **5**. 46
Figure S33. HMBC spectrum of Compound **5**. 47
Figure S34. (+)-HRESIMS spectrum of **6**. 47
Figure S35. ^{1}H NMR (500 MHz, DMSO-d_6) spectrum of **6**. 48
Figure S36. ^{1}H NMR (500 MHz, DMSO-d_6) spectrum of **6**. 48
Figure S37. ^{13}C NMR (125 MHz, DMSO-d_6) spectrum of **6**. 49
Figure S38. HSQC spectrum of Compound **6**. 49
Figure S39. HMBC spectrum of Compound **6**. 50
Figure S40. (+)-HRESIMS spectrum of **7**. 50
Figure S41. ^{1}H NMR (500 MHz, DMSO-d_6) spectrum of **7**. 51

Figure S42. ^{1}H NMR (500 MHz, DMSO-d_6) spectrum of **7**.......... 51
Figure S43. ^{13}C NMR (125 MHz, DMSO-d_6) spectrum of **7**.......... 52
Figure S44. HSQC spectrum of Compound **7**.......... 52
Figure S45. ^{1}H-^{1}H COSY spectrum of Compound **7**.......... 53
Figure S46. HMBC spectrum of Compound **7**.......... 53
Figure S47. (+)-HRESIMS spectrum of **18**.......... 54
Figure S48. ^{1}H NMR (500 MHz, DMSO-d_6) spectrum of **18**.......... 54
Figure S49. ^{1}H NMR (500 MHz, DMSO-d_6) spectrum of **18**.......... 55
Figure S50. ^{13}C NMR (125 MHz, DMSO-d_6) spectrum of **18**.......... 55
Figure S51. HSQC spectrum of Compound **18**.......... 56
Figure S52. ^{1}H-^{1}H COSY spectrum of Compound **18**.......... 56
Figure S53. HMBC spectrum of Compound **18**.......... 57
Figure S54. (+)-HRESIMS spectrum of **19**.......... 57
Figure S55. ^{1}H NMR (500 MHz, DMSO-d_6) spectrum of **19**.......... 58
Figure S56. ^{1}H NMR (500 MHz, DMSO-d_6) spectrum of **19**.......... 58
Figure S57. ^{13}C NMR (125 MHz, DMSO-d_6) spectrum of **19**.......... 59
Figure S58. HSQC spectrum of Compound **19**.......... 59
Figure S59. ^{1}H-^{1}H COSY spectrum of Compound **19**.......... 60
Figure S60. HMBC spectrum of Compound **19**.......... 60
Figure S61. Chiral HPLC of Compound **1**.......... 61
Figure S62. Chiral HPLC of Compound **2**.......... 61
Figure S63. Chiral HPLC of Compound **3**.......... 62
Figure S64. Chiral HPLC of Compound **4**.......... 62
Figure S65. Chiral HPLC of Compound **5**.......... 63
Figure S66. Chiral HPLC of Compound **6**.......... 63
Figure S67. Chiral HPLC of Compound **7**.......... 64
Figure S68. (a) Experimental ECD spectra of (+)- and (-)-**4** in MeOH and experimental ECD spectra of (+)- and (-)-**5** in MeOH. (b) Experimental ECD spectra of (+)- and (-)-**6** in MeOH and experimental ECD spectra of (+)- and (-)-**7** in MeOH.......... 64
Figure S69. Calculated fragments of (1R,6R)-18, (1R,6S)-18, (1S,6R)-18 and (1S,6S)-18.......... 65

1. Computational details

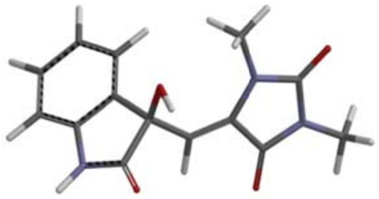

1a1 (92.4%)

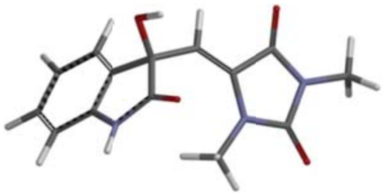

1a2 (7.4%)

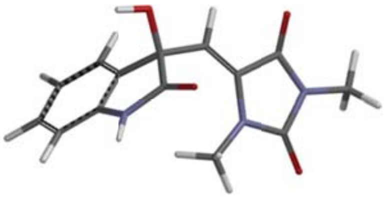

1a3 (0.2%)

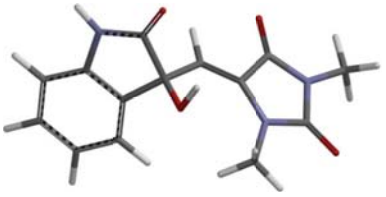

1b1 (92.4%)

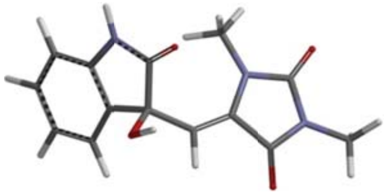

1b2 (7.4%)

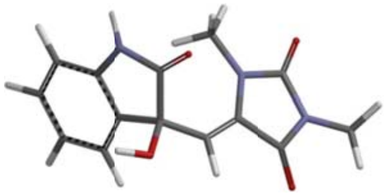

1b3 (0.2%)

Figure S1. Stable conformers of compound **1** with 3*R* (**1a**) and 3*S* (**1b**) configurations, respectively.

Table S1. Important thermodynamic parameters (a.u.) of the optimized compound **1** at B3LYP/6-31G(d,p) level in the gas phase

conformations	E+ZPE	G	conformations	E+ZPE	G
**1a1**	-1006.262388	-1006.311019	**1b1**	-1006.262388	-1006.311019
**1a2**	-1006.260031	-1006.308636	**1b2**	-1006.260031	-1006.308636
**1a3**	-1006.256404	-1006.305003	**1b3**	-1006.256404	-1006.305003

Table S2. Optimized Z-Matrixes of compound **1** in the Gas Phase (Å) at B3LYP/6-31G(d,p) level.

**1a1**				**1a2**				**1a3**			
C	-1.041891	-1.284179	-3.480248	C	-1.112152	-3.157413	-1.917825	C	-1.160179	-2.947684	-1.914091
C	-0.295814	-0.750991	-4.546659	C	-0.600020	-2.776552	-3.171941	C	-0.563933	-2.625953	-3.147645
C	0.908456	-0.084206	-4.312093	C	0.496009	-1.915748	-3.265698	C	0.605525	-1.863808	-3.200702
C	1.326356	0.028309	-3.002338	C	1.043549	-1.457774	-2.085878	C	1.141070	-1.443817	-2.001983
C	0.596932	-0.493962	-1.930622	C	0.541892	-1.821145	-0.832360	C	0.556322	-1.748992	-0.769832
C	-0.597206	-1.158674	-2.160234	C	-0.538322	-2.682968	-0.734374	C	-0.597919	-2.512702	-0.710510
N	2.485766	0.640375	-2.550652	N	2.117889	-0.589075	-1.946736	N	2.275436	-0.666423	-1.820515
C	2.598517	0.541408	-1.169817	C	2.386420	-0.331555	-0.604332	C	2.494353	-0.386147	-0.469590
C	1.311889	-0.201300	-0.634293	C	1.370805	-1.174744	0.244884	C	1.400854	-1.178797	0.341603
O	3.541194	0.934519	-0.498296	O	3.280228	0.384976	-0.180065	O	3.390999	0.323865	-0.043709
C	0.542948	0.717877	0.270522	C	0.611951	-0.447652	1.307852	C	0.696035	-0.387725	1.393906
C	-0.175016	0.546366	1.389075	C	-0.070578	0.704220	1.360024	C	-0.039023	0.732435	1.399186
C	-0.790796	1.743175	2.066768	C	-0.687069	1.139172	2.665835	C	-0.601686	1.244134	2.701612
N	-1.428057	1.244383	3.167172	N	-1.281445	2.339725	2.400397	N	-1.265362	2.393845	2.378676

C	-1.319318	-0.127440	3.229494	C	-1.149456	2.680125	1.072790	C	-1.224160	2.631135	1.023330
N	-0.546254	-0.546360	2.135918	N	-0.394086	1.676285	0.444659	N	-0.456981	1.614613	0.431806
C	-0.266384	-1.935939	1.903538	C	-0.115102	1.725439	-0.960557	C	-0.226037	1.583732	-0.982342
C	-2.183953	2.030176	4.107022	C	-2.028787	3.115310	3.355538	C	-1.996475	3.208091	3.313822
O	-1.818172	-0.847055	4.082377	O	-1.620455	3.673131	0.537748	O	-1.770849	3.556457	0.440739
O	-0.726168	2.897950	1.683626	O	-0.656233	0.523332	3.716634	O	-0.496075	0.713194	3.793003
O	1.710394	-1.399614	-0.000625	O	2.117621	-2.211564	0.891687	O	2.031578	-2.281265	1.003288
H	-1.976135	-1.802749	-3.686404	H	-1.960323	-3.837542	-1.869118	H	-2.068709	-3.547136	-1.898292
H	-0.662068	-0.863013	-5.564903	H	-1.061767	-3.164059	-4.077682	H	-1.021340	-2.978328	-4.070143
H	1.485328	0.323965	-5.133966	H	0.892465	-1.626481	-4.232077	H	1.063443	-1.616599	-4.151813
H	-1.175938	-1.574916	-1.342531	H	-0.922653	-2.993163	0.232614	H	-1.052528	-2.772658	0.241243
H	3.193973	1.062995	-3.132008	H	2.658334	-0.194363	-2.702040	H	2.855885	-0.285058	-2.553495
H	0.606185	1.754182	-0.076787	H	0.639174	-0.999185	2.252128	H	0.839613	-0.840302	2.379547
H	-0.495161	-2.198427	0.869112	H	0.958463	1.843425	-1.113373	H	0.845801	1.622443	-1.178773
H	0.780003	-2.136726	2.146151	H	-0.494001	0.822173	-1.439972	H	-0.681567	0.687324	-1.404884
H	-0.883471	-2.566205	2.551463	H	-0.611609	2.581816	-1.427049	H	-0.683307	2.449400	-1.471255
H	-1.951456	1.703664	5.124695	H	-3.095243	2.965537	3.162679	H	-3.060271	2.970819	3.218983
H	-1.959961	3.093814	3.992906	H	-1.796708	2.800623	4.376202	H	-1.670723	3.007399	4.337717
H	-3.248793	1.859758	3.922590	H	-1.797501	4.176484	3.226615	H	-1.851998	4.265001	3.072616
H	2.454069	-1.151160	0.580044	H	2.878708	-1.770789	1.312793	H	2.292209	-2.920492	0.318496
**1b1**				**1b2**				**1b3**			
C	-2.974629	1.012083	-2.230258	C	-3.850668	0.103289	-0.214200	C	-3.684542	0.061851	-0.344858
C	-2.784048	2.204051	-2.952091	C	-3.937549	1.215889	-1.071354	C	-3.788271	1.233792	-1.117173
C	-1.750150	3.081967	-2.620113	C	-2.935006	2.188395	-1.085157	C	-2.830381	2.245461	-1.015770
C	-0.932846	2.729595	-1.566258	C	-1.865987	2.007165	-0.233178	C	-1.788781	2.043630	-0.135734
C	-1.106776	1.548630	-0.839298	C	-1.761525	0.903716	0.619169	C	-1.668326	0.882134	0.632169
C	-2.133580	0.676566	-1.164425	C	-2.758540	-0.057822	0.643841	C	-2.620420	-0.120177	0.543098
N	0.140137	3.454146	-1.069660	N	-0.759561	2.834824	-0.095316	N	-0.723901	2.898313	0.108410
C	0.719177	2.810055	0.016190	C	0.131678	2.330624	0.849115	C	0.176112	2.347728	1.024001
C	-0.053715	1.450783	0.237165	C	-0.497631	1.014777	1.428877	C	-0.449608	0.987352	1.513816
O	1.642358	3.237783	0.693613	O	1.183851	2.851409	1.186796	O	1.224301	2.869856	1.367731
C	0.889937	0.299788	0.038217	C	0.375021	-0.198654	1.450948	C	0.487943	-0.174918	1.519567
C	1.034150	-0.903033	0.611342	C	1.181633	-0.797445	0.564267	C	1.231830	-0.792390	0.591960
C	2.153851	-1.807993	0.166233	C	1.954589	-2.024074	0.979367	C	2.083657	-1.971977	0.989083
N	2.040881	-2.927031	0.941330	N	2.686755	-2.376058	-0.118407	N	2.727405	-2.350961	-0.154835
C	0.955278	-2.843753	1.785213	C	2.424326	-1.542579	-1.182493	C	2.339368	-1.578686	-1.226275
N	0.345533	-1.597096	1.577637	N	1.508615	-0.569123	-0.750424	N	1.432573	-0.615170	-0.756019
C	-0.836241	-1.210977	2.297066	C	1.006341	0.429324	-1.648034	C	0.844848	0.347781	-1.639993
C	2.892899	-4.083392	0.846131	C	3.556025	-3.521086	-0.193396	C	3.625148	-3.471522	-0.256924
O	0.587692	-3.709177	2.566258	O	2.895779	-1.644825	-2.305635	O	2.710720	-1.718706	-2.382560
O	2.965433	-1.568898	-0.710493	O	1.906706	-2.570931	2.067045	O	2.147075	-2.478059	2.095448
O	-0.645737	1.468743	1.519929	O	-0.877925	1.287611	2.782398	O	-0.912336	1.154517	2.858811
H	-3.788770	0.344020	-2.504288	H	-4.648961	-0.636353	-0.213120	H	-4.446237	-0.710282	-0.437858

H	-3.452674	2.443547	-3.776160	H	-4.800565	1.320201	-1.725618	H	-4.627556	1.351073	-1.799988
H	-1.604995	4.002239	-3.174087	H	-3.005009	3.047250	-1.742584	H	-2.911602	3.147548	-1.611636
H	-2.287949	-0.242891	-0.609813	H	-2.702578	-0.906874	1.318359	H	-2.548795	-1.019861	1.147680
H	0.446940	4.355881	-1.402656	H	-0.605196	3.699932	-0.591423	H	-0.559382	3.784829	-0.346170
H	1.618288	0.520639	-0.748726	H	0.333446	-0.690479	2.427148	H	0.581848	-0.591465	2.526841
H	-0.572731	-0.454768	3.040581	H	-0.081867	0.370251	-1.688116	H	-0.234191	0.193618	-1.678783
H	-1.595852	-0.844079	1.604496	H	1.345322	1.413586	-1.322251	H	1.096730	1.354102	-1.304146
H	-1.258496	-2.067782	2.831318	H	1.384884	0.268113	-2.662051	H	1.232634	0.231720	-2.656686
H	2.339702	-4.879628	0.339255	H	4.466194	-3.254719	-0.738232	H	4.457170	-3.217011	-0.919553
H	3.799380	-3.852566	0.280857	H	3.808031	-3.882062	0.807021	H	4.002023	-3.755518	0.729010
H	3.153446	-4.427432	1.851155	H	3.038650	-4.311451	-0.745301	H	3.077662	-4.313051	-0.691717
H	0.054105	1.779982	2.124339	H	-0.0992803	1.6981774	3.2019393	H	-1.684717	1.744452	2.829055

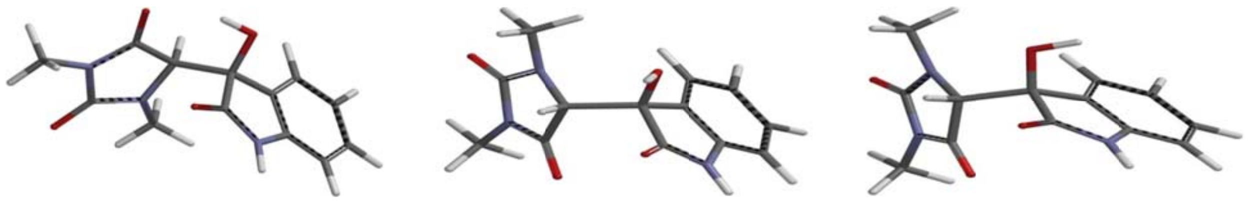

2a1 (90.3%) **2a2** (8.8%) **2a3** (0.8%)

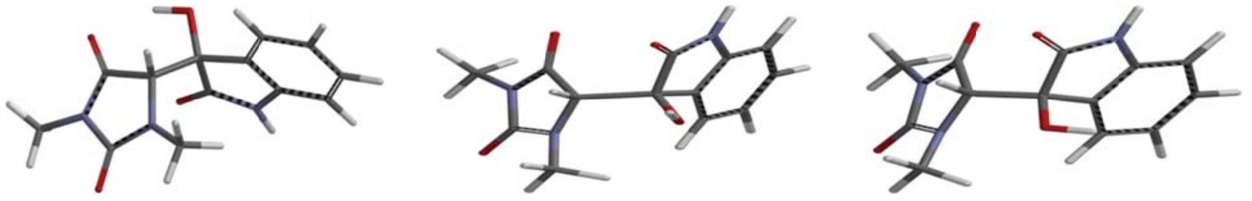

2b1 (90.3%) **2b2** (8.9%) **2b3** (0.8%)

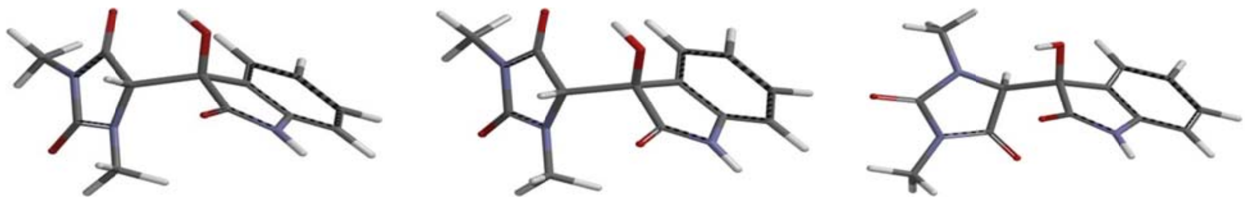

2c1 (50.4%) **2c2** (49.2%) **2c3** (0.4%)

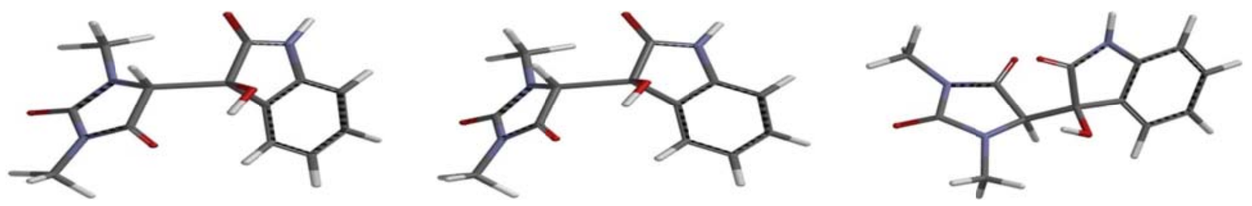

2d1 (50.5%) **2d2** (49.1%) **2d3** (0.4%)

Figure S2. Stable conformers of compound **2** with 3*R*,1′*R* (**2a**), 3*S*,1′*S* (**2b**), 3*R*,1′*S* (**2c**) and 3*S*,1′*R* (**2d**) configurations, respectively.

Table S3. Important thermodynamic parameters (a.u.) of the optimized compound **2** at B3LYP/6-31G(d,p) level in the gas phase

conformations	E+ZPE	G	conformations	E+ZPE	G
**2a1**	-968.182561	-968.228144	**2b1**	-968.182561	-968.228144
**2a2**	-968.180026	-968.225951	**2b2**	-968.180027	-968.225953
**2a3**	-968.177590	-968.223721	**2b3**	-968.177590	-968.223721
**2c1**	-968.180340	-968.226320	**2d1**	-968.180337	-968.226298
**2c2**	-968.180337	-968.226298	**2d2**	-968.180332	-968.226270
**2c3**	-968.175139	-968.221672	**2d3**	-968.175139	-968.221672

Table S4. Optimized Z-Matrixes of compound **2** in the Gas Phase (Å) at B3LYP/6-31G(d,p) level.

**2a1**				**2a2**				**2a3**			
C	-3.886499	0.489830	-1.251021	C	-1.661419	2.494565	-1.362748	C	-1.781872	2.498339	-1.265038
C	-4.357017	0.686372	0.048390	C	-2.742536	2.562060	-0.483006	C	-2.877494	2.481831	-0.399989
C	-3.572565	0.364993	1.164436	C	-3.085927	1.476397	0.333001	C	-3.182742	1.351734	0.369313
C	-2.303621	-0.150040	0.931210	C	-2.309719	0.329666	0.235993	C	-2.351011	0.245468	0.247906
C	-1.809797	-0.333818	-0.369663	C	-1.212044	0.248464	-0.638449	C	-1.236323	0.251179	-0.609059
C	-2.602061	-0.030659	-1.465099	C	-0.885290	1.329533	-1.443813	C	-0.952153	1.373711	-1.375021
N	-1.337863	-0.564063	1.862088	N	-2.463534	-0.888801	0.919141	N	-2.460867	-1.003116	0.874453
C	-0.195709	-1.037618	1.253478	C	-1.513819	-1.803088	0.551320	C	-1.439141	-1.859916	0.520546
C	-0.418892	-0.924180	-0.293016	C	-0.574687	-1.111807	-0.477449	C	-0.544769	-1.092790	-0.515319
C	0.706841	-0.035864	-0.909857	C	0.877836	-1.088870	0.080588	C	0.907104	-1.066415	0.019501
C	2.070388	-0.725316	-0.748605	C	1.010884	-0.225742	1.342107	C	1.021997	-0.249086	1.313951
N	2.904526	0.179095	-0.160657	N	2.014146	0.675575	1.088625	N	2.025636	0.662789	1.105097
C	2.248295	1.401221	0.113967	C	2.570114	0.504491	-0.199648	C	2.584305	0.552863	-0.188030
N	0.934934	1.253724	-0.280032	N	1.855449	-0.496841	-0.817625	N	1.873160	-0.419913	-0.855473
C	0.083799	2.419716	-0.461720	C	2.366012	-1.149363	-2.015060	C	2.416511	-1.034489	-2.060296
C	4.268740	-0.104503	0.251384	C	2.458199	1.698689	2.017920	C	2.456752	1.650464	2.077642
O	2.775687	2.384208	0.595625	O	3.507893	1.137894	-0.645834	O	3.522151	1.208615	-0.600685
O	2.349236	-1.868221	-1.082647	O	0.350908	-0.310606	2.360630	O	0.350285	-0.367571	2.321067
O	-0.405545	-2.184698	-0.922607	O	-0.611338	-1.870582	-1.680255	O	-0.474473	-1.808205	-1.743681
O	0.799924	-1.458531	1.808881	O	-1.427940	-2.975042	0.871201	O	-1.279622	-3.001404	0.895175
H	0.483077	0.066021	-1.981555	H	1.136106	-2.129390	0.330967	H	1.178558	-2.113551	0.218322
H	-4.519281	0.734428	-2.097957	H	-1.419705	3.347242	-1.988872	H	-1.571337	3.384616	-1.854731
H	-5.353313	1.089480	0.203436	H	-3.334589	3.470712	-0.428047	H	-3.512347	3.359399	-0.321384
H	-3.949810	0.507183	2.172288	H	-3.930757	1.532247	1.012110	H	-4.040298	1.343373	1.034676
H	-2.236467	-0.204141	-2.472871	H	-0.045988	1.272516	-2.128488	H	-0.099775	1.377917	-2.046563
H	-1.453805	-0.565601	2.865123	H	-3.186758	-1.096677	1.591836	H	-3.131857	-1.239650	1.590418
H	-0.118948	2.596085	-1.525564	H	2.768554	-2.143179	-1.778005	H	2.798953	-2.040509	-1.846578
H	-0.868219	2.303346	0.059908	H	1.575583	-1.259694	-2.757432	H	1.649043	-1.111095	-2.829885
H	0.618016	3.277087	-0.050892	H	3.169382	-0.525833	-2.408417	H	3.236290	-0.403796	-2.405310
H	4.700334	0.828189	0.613989	H	3.334843	2.177057	1.581181	H	3.336893	2.146982	1.669142

H	4.272249	-0.854391	1.046782	H	1.670731	2.440344	2.178589	H	1.665779	2.383815	2.258485
H	4.842499	-0.481723	-0.597646	H	2.714441	1.244592	2.977843	H	2.703600	1.160031	3.021926
H	0.526664	-2.480130	-0.962157	H	-0.636073	-2.802760	-1.404734	H	-1.352729	-1.794680	-2.148709
**2b1**				**2b2**				**2b3**			
C	3.886500	0.489835	-1.251018	C	1.661407	2.494633	-1.362635	C	1.781872	2.498339	-1.265038
C	4.357017	0.686372	0.048395	C	2.742550	2.562083	-0.482919	C	2.877493	2.481831	-0.399989
C	3.572564	0.364990	1.164439	C	3.085955	1.476385	0.333032	C	3.182741	1.351734	0.369313
C	2.303620	-0.150043	0.931211	C	2.309736	0.329662	0.235991	C	2.351011	0.245468	0.247906
C	1.809797	-0.333817	-0.369663	C	1.212040	0.248503	-0.638421	C	1.236323	0.251179	-0.609059
C	2.602062	-0.030654	-1.465098	C	0.885263	1.329615	-1.443723	C	0.952152	1.373711	-1.375021
N	1.337862	-0.564069	1.862087	N	2.463536	-0.888822	0.919118	N	2.460867	-1.003116	0.874452
C	0.195708	-1.037623	1.253474	C	1.513818	-1.803094	0.551275	C	1.439141	-1.859916	0.520546
C	0.418893	-0.924179	-0.293019	C	0.574688	-1.111775	-0.477475	C	0.544769	-1.092791	-0.515319
C	-0.706841	-0.035860	-0.909857	C	-0.877837	-1.088882	0.080514	C	-0.907104	-1.066416	0.019502
C	-2.070387	-0.725314	-0.748606	C	-1.010891	-0.225840	1.342080	C	-1.021997	-0.249086	1.313951
N	-2.904525	0.179093	-0.160653	N	-2.014296	0.675371	1.088724	N	-2.025636	0.662789	1.105097
C	-2.248295	1.401220	0.113973	C	-2.570183	0.504455	-0.199582	C	-2.584305	0.552863	-0.188030
N	-0.934934	1.253726	-0.280030	N	-1.855459	-0.496789	-0.817661	N	-1.873160	-0.419913	-0.855473
C	-0.083802	2.419720	-0.461719	C	-2.365997	-1.149171	-2.015179	C	-2.416511	-1.034490	-2.060295
C	-4.268739	-0.104505	0.251386	C	-2.458167	1.698575	2.018001	C	-2.456752	1.650464	2.077642
O	-2.775689	2.384207	0.595628	O	-3.507923	1.137915	-0.645770	O	-3.522151	1.208615	-0.600685
O	-2.349232	-1.868219	-1.082650	O	-0.350862	-0.310715	2.360564	O	-0.350285	-0.367571	2.321067
O	0.405545	-2.184694	-0.922616	O	0.611400	-1.870497	-1.680314	O	0.474473	-1.808206	-1.743681
O	-0.799924	-1.458538	1.808875	O	1.427962	-2.975066	0.871098	O	1.279623	-3.001404	0.895175
H	-0.483078	0.066027	-1.981556	H	-1.136098	-2.129419	0.330847	H	-1.178558	-2.113551	0.218322
H	4.519282	0.734435	-2.097953	H	1.419688	3.347341	-1.988715	H	1.571336	3.384616	-1.854732
H	5.353312	1.089480	0.203442	H	3.334608	3.470731	-0.427942	H	3.512346	3.359399	-0.321385
H	3.949808	0.507177	2.172292	H	3.930797	1.532196	1.012130	H	4.040298	1.343373	1.034675
H	2.236469	-0.204133	-2.472871	H	0.045940	1.272634	-2.128375	H	0.099775	1.377916	-2.046564
H	1.453804	-0.565610	2.865122	H	3.186864	-1.096778	1.591678	H	3.131857	-1.239649	1.590418
H	0.118936	2.596095	-1.525564	H	-2.768314	-2.143110	-1.778285	H	-2.798953	-2.040509	-1.846577
H	-0.618018	3.277088	-0.050883	H	-3.169508	-0.525703	-2.408340	H	-3.236290	-0.403797	-2.405310
H	0.868219	2.303350	0.059901	H	-1.575626	-1.259192	-2.757666	H	-1.649043	-1.111096	-2.829885
H	-4.272248	-0.854371	1.046803	H	-1.670511	2.440009	2.178760	H	-1.665779	2.383817	2.258482
H	-4.700343	0.828193	0.613964	H	-3.334666	2.177162	1.581217	H	-3.336893	2.146981	1.669143
H	-4.842487	-0.481752	-0.597639	H	-2.714636	1.244494	2.977871	H	-2.703596	1.160032	3.021926
H	-0.526665	-2.480121	-0.962171	H	0.636078	-2.802683	-1.404824	H	1.352729	-1.794680	-2.148709
**2c1**				**2c2**				**2c3**			
C	1.768299	2.847859	-0.079782	C	1.768762	2.847766	-0.079914	C	3.802932	0.841052	-1.387659
C	2.854877	2.458038	-0.863299	C	2.855415	2.457771	-0.863241	C	4.386445	-0.282782	-0.801369
C	3.172072	1.105278	-1.041425	C	3.172485	1.104972	-1.041212	C	3.709280	-1.032808	0.169110
C	2.360240	0.168115	-0.416803	C	2.360477	0.167903	-0.416637	C	2.433761	-0.618201	0.524999
C	1.251554	0.540161	0.359467	C	1.251711	0.540151	0.359448	C	1.829706	0.504133	-0.061550

C	0.961410	1.884902	0.543025	C	0.961677	1.884927	0.542836	C	2.510973	1.240213	-1.017352
N	2.478600	-1.232948	-0.426778	N	2.478693	-1.233085	-0.426430	N	1.563045	-1.175086	1.476397
C	1.504854	-1.848598	0.328805	C	1.504711	-1.848645	0.328893	C	0.384569	-0.479876	1.566390
C	0.607109	-0.716772	0.923782	C	0.607076	-0.716699	0.923807	C	0.451785	0.682163	0.529234
C	-0.874184	-0.974233	0.507661	C	-0.874235	-0.974057	0.507532	C	-0.689189	0.578792	-0.545681
C	-1.775374	0.115620	1.088794	C	-1.775549	0.115706	1.088774	C	-1.032336	-0.881799	-0.873936
N	-2.548054	0.580956	0.068284	N	-2.548351	0.580897	0.068204	N	-2.376968	-1.023902	-0.634197
C	-2.235265	-0.059356	-1.162610	C	-2.235506	-0.059286	-1.162590	C	-2.975613	0.185464	-0.225998
N	-1.206158	-0.929187	-0.906757	N	-1.206118	-0.928880	-0.906825	N	-1.965835	1.123319	-0.105516
C	-0.811411	-1.935042	-1.881697	C	-0.811118	-1.934626	-1.881763	C	-2.296841	2.537823	-0.244650
C	-3.532662	1.643165	0.181965	C	-3.532874	1.643205	0.181901	C	-3.106712	-2.273196	-0.754102
O	-2.805410	0.150223	-2.214792	O	-2.805797	0.150022	-2.214754	O	-4.165354	0.352994	-0.046108
O	-1.766582	0.527021	2.244455	O	-1.766885	0.527119	2.244401	O	-0.265164	-1.753196	-1.236042
O	0.684413	-0.834449	2.329195	O	0.684221	-0.834412	2.329223	O	0.347725	1.907437	1.239836
O	1.347131	-3.041943	0.481020	O	1.346774	-3.041998	0.480894	O	-0.535992	-0.675759	2.339357
H	-1.126990	-1.948754	0.949241	H	-1.127063	-1.948609	0.949075	H	-0.340075	1.086960	-1.454642
H	1.549410	3.902318	0.054013	H	1.549965	3.902254	0.053790	H	4.351197	1.408753	-2.132364
H	3.472904	3.212822	-1.340110	H	3.473605	3.212443	-1.340018	H	5.386157	-0.585141	-1.098364
H	4.025506	0.805142	-1.641530	H	4.025978	0.804702	-1.641174	H	4.169333	-1.904343	0.623779
H	0.131451	2.190363	1.172790	H	0.131611	2.190534	1.172385	H	2.057490	2.119438	-1.465853
H	3.210867	-1.754705	-0.885730	H	3.210639	-1.755081	-0.885605	H	1.763600	-1.976745	2.056219
H	-0.924440	-2.940353	-1.464192	H	-0.922896	-2.939886	-1.463821	H	-2.313720	2.844535	-1.300317
H	-1.458564	-1.819355	-2.751763	H	-1.458944	-1.819882	-2.751455	H	-3.288215	2.693745	0.181025
H	0.227997	-1.800520	-2.193307	H	0.227978	-1.799174	-2.194109	H	-1.561894	3.142012	0.285531
H	-3.045270	2.602697	0.376885	H	-3.045007	2.604579	0.366482	H	-2.838267	-2.955094	0.057876
H	-4.071273	1.688905	-0.764469	H	-4.078688	1.682326	-0.760697	H	-4.169796	-2.038362	-0.701178
H	-4.221290	1.424107	1.000471	H	-4.214737	1.428875	1.007223	H	-2.868354	-2.747275	-1.708356
H	-0.026552	-0.276051	2.698125	H	-0.026714	-0.275988	2.698169	H	-0.391426	1.783527	1.858898
**2d1**				**2d2**				**2d3**			
C	-1.768763	2.847766	-0.079915	C	-1.768376	2.847877	-0.079864	C	-3.802931	0.841049	-1.387662
C	-2.855416	2.457771	-0.863241	C	-2.855162	2.458086	-0.863098	C	-4.386444	-0.282783	-0.801370
C	-3.172485	1.104972	-1.041212	C	-3.172479	1.105337	-1.041052	C	-3.709280	-1.032808	0.169111
C	-2.360477	0.167902	-0.416637	C	-2.360595	0.168134	-0.416524	C	-2.433761	-0.618200	0.525000
C	-1.251711	0.540151	0.359448	C	-1.251734	0.540177	0.359525	C	-1.829706	0.504132	-0.061551
C	-0.961677	1.884927	0.542836	C	-0.961406	1.884900	0.542836	C	-2.510972	1.240211	-1.017355
N	-2.478693	-1.233085	-0.426430	N	-2.478964	-1.232851	-0.426466	N	-1.563045	-1.175083	1.476399
C	-1.504711	-1.848645	0.328893	C	-1.504989	-1.848606	0.328726	C	-0.384570	-0.479872	1.566392
C	-0.607076	-0.716698	0.923808	C	-0.607201	-0.716818	0.923690	C	-0.451785	0.682164	0.529234
C	0.874235	-0.974057	0.507532	C	0.874076	-0.974012	0.507349	C	0.689189	0.578791	-0.545682
C	1.775549	0.115707	1.088774	C	1.775511	0.115539	1.088791	C	1.032335	-0.881801	-0.873934
N	2.548351	0.580897	0.068204	N	2.548476	0.580677	0.068308	N	2.376968	-1.023903	-0.634197
C	2.235506	-0.059286	-1.162590	C	2.235662	-0.059346	-1.162559	C	2.975613	0.185463	-0.225998
N	1.206118	-0.928880	-0.906825	N	1.205861	-0.928542	-0.907054	N	1.965835	1.123318	-0.105518

C	0.811118	-1.934626	-1.881763	C	0.810857	-1.934313	-1.881967	C	2.296842	2.537822	-0.244654
C	3.532874	1.643205	0.181901	C	3.533215	1.642757	0.182183	C	3.106712	-2.273197	-0.754102
O	2.805797	0.150022	-2.214754	O	2.806282	0.149822	-2.214574	O	4.165354	0.352993	-0.046107
O	1.766885	0.527119	2.244401	O	1.766841	0.526801	2.244468	O	0.265163	-1.753198	-1.236037
O	-0.684221	-0.834412	2.329223	O	-0.684298	-0.834730	2.329125	O	-0.347725	1.907439	1.239834
O	-1.346774	-3.041998	0.480894	O	-1.347228	-3.041979	0.480671	O	0.535990	-0.675753	2.339361
H	1.127063	-1.948609	0.949075	H	1.126982	-1.948671	0.948634	H	0.340075	1.086958	-1.454643
H	-1.549965	3.902254	0.053790	H	-1.549370	3.902324	0.053826	H	-4.351196	1.408750	-2.132367
H	-3.473605	3.212443	-1.340018	H	-3.473253	3.212866	-1.339832	H	-5.386157	-0.585143	-1.098364
H	-4.025978	0.804702	-1.641174	H	-4.026045	0.805233	-1.640991	H	-4.169333	-1.904342	0.623781
H	-0.131611	2.190535	1.172385	H	-0.131246	2.190391	1.172310	H	-2.057488	2.119435	-1.465857
H	-3.210639	-1.755082	-0.885605	H	-3.211118	-1.754680	-0.885502	H	-1.763602	-1.976739	2.056226
H	-0.227978	-1.799174	-2.194108	H	-0.228615	-1.799534	-2.193310	H	1.561894	3.142012	0.285524
H	1.458945	-1.819882	-2.751454	H	1.457860	-1.818720	-2.752158	H	3.288215	2.693744	0.181023
H	0.922896	-2.939886	-1.463820	H	0.923811	-2.939673	-1.464538	H	2.313724	2.844532	-1.300322
H	3.045007	2.604579	0.366481	H	3.045296	2.605663	0.358651	H	2.838257	-2.955101	0.057869
H	4.078688	1.682325	-0.760698	H	4.084767	1.676604	-0.757277	H	4.169795	-2.038364	-0.701165
H	4.214737	1.428875	1.007222	H	4.209599	1.431960	1.012856	H	2.868363	-2.747271	-1.708362
H	0.026714	-0.275988	2.698169	H	0.026665	-0.276345	2.698084	H	0.391425	1.783530	1.858897

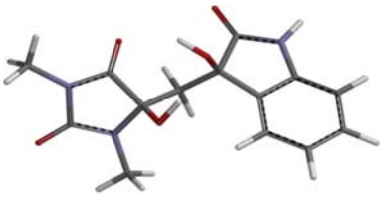

3a1 (52.6%)

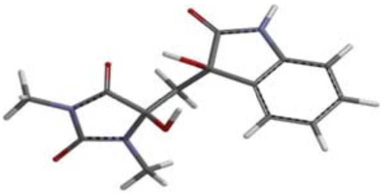

3a2 (38.0%)

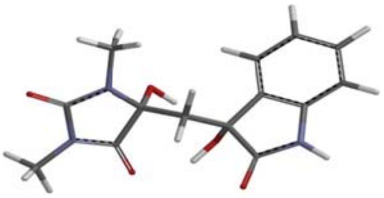

3a3 (9.4%)

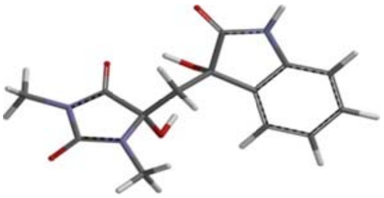

3b1 (52.6%)

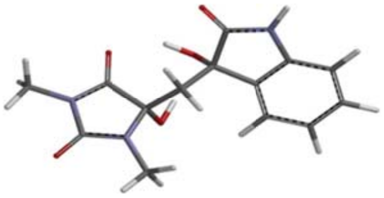

3b2 (38.0%)

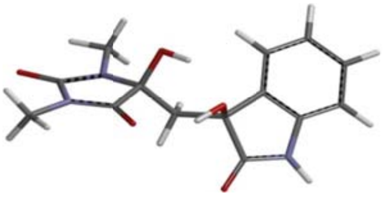

3b3 (9.4%)

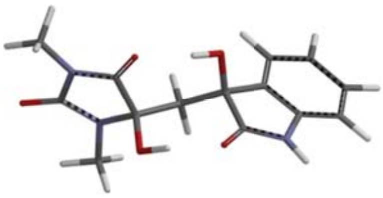

3c (99.988%)

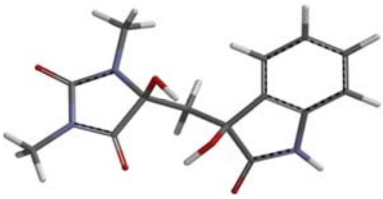

3d (99.98%)

Figure S3. Stable conformers of compound **3** with 3*R*,1′*R* (**3a**), 3*S*,1′*S* (**3b**), 3*R*,1′*S* (**3c**) and 3*S*,1′*R* (**3d**) configurations, respectively.

Table S5. Important thermodynamic parameters (a.u.) of the optimized compound **3** at B3LYP/6-31G(d,p) level in the gas phase

conformations	E+ZPE	G	conformations	E+ZPE	G
**2a1**	-1082.689287	-1082.737802	**2b1**	-1082.689287	-1082.737802
**2a2**	-1082.689429	-1082.737497	**2b2**	-1082.689429	-1082.737497
**2a3**	-1082.689375	-1082.736176	**2b3**	-1082.689375	-1082.736176
**2c1**	-1082.691764	-1082.739958	**2d1**	-1082.689287	-1082.737802

Table S6. Optimized Z-Matrixes of compound **3** in the Gas Phase (Å) at B3LYP/6-31G(d,p) level.

**3a1**				**3a2**				**3a3**			
C	-3.635299	2.158798	0.707180	C	3.641858	-2.154654	0.706535	C	3.634463	-2.159532	0.707139
C	-4.754643	1.518507	0.173804	C	4.759569	-1.510817	0.173998	C	4.754166	-1.519369	0.174335
C	-4.663687	0.231063	-0.370698	C	4.665077	-0.223512	-0.370248	C	4.663760	-0.231765	-0.369845
C	-3.420241	-0.387307	-0.354083	C	3.419766	0.391101	-0.354233	C	3.420466	0.386922	-0.353494
C	-2.284962	0.241552	0.180847	C	2.286141	-0.241341	0.179951	C	2.284862	-0.241776	0.180897
C	-2.387501	1.519189	0.710908	C	2.392141	-1.518778	0.709747	C	2.386846	-1.519596	0.710635
N	-3.068545	-1.655134	-0.844870	N	3.064368	1.657868	-0.845084	N	3.069268	1.655004	-0.844003
C	-1.728361	-1.921329	-0.684787	C	1.723109	1.919579	-0.686272	C	1.729100	1.921461	-0.684257
C	-1.100857	-0.699411	0.062727	C	1.099287	0.696119	0.061751	C	1.101057	0.699498	0.062743
C	0.112728	-0.156021	-0.741670	C	-0.112836	0.148280	-0.742225	C	-0.112327	0.156376	-0.742396
C	1.281293	0.366802	0.129943	C	-1.281019	-0.371505	0.131319	C	-1.281280	-0.366216	0.128692
C	2.234367	-0.787905	0.544558	C	-2.231666	0.784379	0.545576	C	-2.234149	0.788712	0.543205
N	3.491427	-0.421537	0.173956	N	-3.489210	0.423261	0.177555	N	-3.491648	0.421190	0.175318
C	3.493202	0.827227	-0.497744	C	-3.495057	-0.826798	-0.496968	C	-3.493538	-0.827493	-0.496599
N	2.182034	1.227028	-0.610363	N	-2.185798	-1.229676	-0.607293	N	-2.182213	-1.226044	-0.611851
O	4.490906	1.387279	-0.902347	O	-4.491527	-1.387281	-0.904762	O	-4.491564	-1.389388	-0.897841
O	1.908533	-1.821993	1.114418	O	-1.909139	1.820204	1.114292	O	-1.908160	1.823065	1.112432
O	-1.135451	-2.921990	-1.035407	O	1.127123	2.917979	-1.038098	O	1.136491	2.922343	-1.034739
O	-0.732607	-1.136706	1.378025	O	0.730244	1.132614	1.376958	O	0.732552	1.136524	1.377930
O	0.844889	1.056159	1.287376	O	-0.843849	-1.062912	1.287313	O	-0.845294	-1.056033	1.286054
C	4.703037	-1.188851	0.412988	C	-4.691369	1.204808	0.417275	C	-4.702908	1.189660	0.412208
C	1.835069	2.579913	-1.014461	C	-1.841079	-2.582316	-1.014704	C	-1.835260	-2.578983	-1.015868
H	-3.730025	3.157620	1.120920	H	3.739374	-3.153310	1.120032	H	3.728803	-3.158504	1.120608
H	-5.715579	2.024323	0.174969	H	5.722027	-2.013732	0.175599	H	5.714968	-2.025445	0.175690
H	-5.535562	-0.262454	-0.788713	H	5.535731	0.272682	-0.787641	H	5.535893	0.261647	-0.787442
H	-1.509522	2.012588	1.115315	H	1.515363	-2.014903	1.113455	H	1.508520	-2.012848	1.114474
H	-3.703288	-2.318292	-1.264817	H	3.697157	2.322788	-1.265188	H	3.704277	2.318015	-1.263787
H	0.496688	-0.957454	-1.381793	H	-0.497292	0.946788	-1.385704	H	0.227207	-0.651463	-1.395740
H	-0.226627	0.651759	-1.395195	H	0.228049	-0.661741	-1.392151	H	-0.495825	0.957939	-1.382618
H	0.049775	-1.727133	1.295256	H	-0.053533	1.721764	1.294697	H	-0.049528	1.727326	1.294963
H	0.244655	0.429507	1.743215	H	-0.243663	-0.436455	1.743441	H	-0.245126	-0.429561	1.742205
H	5.200512	-1.410809	-0.533656	H	-4.616411	2.176344	-0.077538	H	-5.438978	0.570741	0.928961

H	5.389133	-0.623479	1.047823	H	-5.530799	0.640940	0.010944	H	-5.130511	1.531075	-0.533846
H	4.411376	-2.114418	0.909209	H	-4.830063	1.363121	1.489363	H	-4.431846	2.048040	1.026745
H	1.079736	2.569855	-1.806181	H	-2.747772	-3.046834	-1.402509	H	-1.459503	-3.152932	-0.164094
H	1.457132	3.153004	-0.163093	H	-1.083116	-2.571100	-1.803820	H	-1.078172	-2.568732	-1.805871
H	2.742152	3.047521	-1.397908	H	-1.466912	-3.158629	-0.163875	H	-2.741779	-3.045829	-1.401558
**3b1**				**3b2**				**3b3**			
C	-3.635299	-2.158798	0.707180	C	-3.641858	-2.154654	0.706535	C	3.634463	2.159532	0.707139
C	-4.754643	-1.518507	0.173804	C	-4.759569	-1.510817	0.173998	C	4.754166	1.519369	0.174335
C	-4.663687	-0.231063	-0.370698	C	-4.665077	-0.223512	-0.370248	C	4.663760	0.231765	-0.369845
C	-3.420241	0.387307	-0.354083	C	-3.419766	0.391101	-0.354233	C	3.420466	-0.386922	-0.353494
C	-2.284962	-0.241552	0.180847	C	-2.286141	-0.241341	0.179951	C	2.284862	0.241776	0.180897
C	-2.387501	-1.519189	0.710908	C	-2.392141	-1.518778	0.709747	C	2.386846	1.519596	0.710635
N	-3.068545	1.655134	-0.844870	N	-3.064368	1.657868	-0.845084	N	3.069268	-1.655004	-0.844003
C	-1.728361	1.921329	-0.684787	C	-1.723109	1.919579	-0.686272	C	1.729100	-2.605718	
C	-1.100857	0.699411	0.062727	C	-1.099287	0.696119	0.061751	C	1.101057	-0.699498	0.062743
C	0.112728	0.156021	-0.741670	C	0.112836	0.148280	-0.742225	C	-0.112327	-0.156376	-0.742396
C	1.281293	-0.366802	0.129943	C	1.281019	-0.371505	0.131319	C	-1.281280	0.366216	0.128692
C	2.234367	0.787905	0.544558	C	2.231666	0.784379	0.545576	C	-2.234149	-0.788712	0.543205
N	3.491427	0.421537	0.173956	N	3.489210	0.423261	0.177555	N	-3.491648	-0.421190	0.175318
C	3.493202	-0.827227	-0.497744	C	3.495057	-0.826798	-0.496968	C	-3.493538	0.827493	-0.496599
N	2.182034	-1.227028	-0.610363	N	2.185798	-1.229676	-0.607293	N	-2.182213	1.226044	-0.611851
O	4.490906	-1.387279	-0.902347	O	4.491527	-1.387281	-0.904762	O	-4.491564	1.389388	-0.897841
O	1.908533	1.821993	1.114418	O	1.909139	1.820204	1.114292	O	-1.908160	-1.823065	1.112432
O	-1.135451	2.921990	-1.035407	O	-1.127123	2.917979	-1.038098	O	1.136491	-2.922343	-1.034739
O	-0.732607	1.136706	1.378025	O	-0.730244	1.132614	1.376958	O	0.732552	-1.136524	1.377930
O	0.844889	-1.056159	1.287376	O	0.843849	-1.062912	1.287313	O	-0.845294	1.056033	1.286054
C	4.703037	1.188851	0.412988	C	4.691369	1.204808	0.417275	C	-4.702908	-1.189660	0.412208
C	1.835069	-2.579913	-1.014461	C	1.841079	-2.582316	-1.014704	C	-1.835260	2.578983	-1.015868
H	-3.730025	-3.157620	1.120920	H	-3.739374	-3.153310	1.120032	H	3.728803	3.158504	1.120608
H	-5.715579	-2.024323	0.174969	H	-5.722027	-2.013732	0.175599	H	5.714968	2.025445	0.175690
H	-5.535562	0.262454	-0.788713	H	-5.535731	0.272682	-0.787641	H	5.535893	-0.261647	-0.787442
H	-1.509522	-2.012588	1.115315	H	-1.515363	-2.014903	1.113455	H	1.508520	2.012848	1.114474
H	-3.703288	2.318292	-1.264817	H	-3.697157	2.322788	-1.265188	H	3.704277	-2.318015	-1.263787
H	0.496688	0.957454	-1.381793	H	-0.228049	-0.661741	-1.392151	H	0.227207	0.651463	-1.395740
H	-0.226627	-0.651759	-1.395195	H	0.497292	0.946788	-1.385704	H	-0.495825	-0.957939	-1.382618
H	0.049775	1.727133	1.295256	H	0.053533	1.721764	1.294697	H	-0.049528	-1.727326	1.294963
H	0.244655	-0.429507	1.743215	H	0.243663	-0.436455	1.743441	H	-0.245126	0.429561	1.742205
H	5.200512	1.410809	-0.533656	H	4.830063	1.363121	1.489363	H	-5.438978	-0.570741	0.928961
H	5.389133	0.623479	1.047823	H	5.530799	0.640940	0.010944	H	-5.130511	-1.531075	-0.533846
H	4.411376	2.114418	0.909209	H	4.616411	2.176344	-0.077538	H	-4.431846	-2.048040	1.026745
H	1.079736	-2.569855	-1.806181	H	1.083116	-2.571100	-1.803820	H	-1.459503	3.152932	-0.164094
H	1.457132	-3.153004	-0.163093	H	2.747772	-3.046834	-1.402509	H	-1.078172	2.568732	-1.805871
H	2.742152	-3.047521	-1.397908	H	1.466912	-3.158629	-0.163875	H	-2.741779	3.045829	-1.401558

3c				3d			
C	4.074600	-1.965600	-1.114400	C	-3.635300	-2.158800	0.707200
C	5.027900	-1.347300	-0.304200	C	-4.754600	-1.518500	0.173800
C	4.710900	-0.210300	0.451000	C	-4.663700	-0.231100	-0.370700
C	3.412500	0.271800	0.363400	C	-3.420200	0.387300	-0.354100
C	2.439700	-0.343200	-0.434200	C	-2.285000	-0.241600	0.180800
C	2.768400	-1.459900	-1.185200	C	-2.387500	-1.519200	0.710900
N	2.843900	1.389200	1.004100	N	-3.068500	1.655100	-0.844900
C	1.527600	1.557300	0.681100	C	-1.728400	1.921300	-0.684800
C	1.131800	0.428000	-0.330100	C	-1.100900	0.699400	0.062700
C	-0.006100	-0.492100	0.187600	C	0.112700	0.156000	-0.741700
C	-1.414300	0.081700	0.490400	C	1.281300	-0.366800	0.129900
C	-2.171300	0.654400	-0.738500	C	2.234400	0.787900	0.544600
N	-3.377600	0.031200	-0.797600	N	3.491400	0.421500	0.174000
C	-3.506800	-0.956700	0.214700	C	3.493200	-0.827200	-0.497700
N	-2.312200	-0.998600	0.887200	N	2.182000	-1.227000	-0.610400
O	-4.501400	-1.629000	0.399300	O	4.490900	-1.387300	-0.902300
O	-1.791300	1.552300	-1.481000	O	1.908500	1.822000	1.114400
O	0.811800	2.466900	1.088500	O	-1.135500	2.922000	-1.035400
O	0.860200	1.006600	-1.586200	O	-0.732600	1.136700	1.378000
O	-1.427100	1.043700	1.513300	O	0.844900	-1.056200	1.287400
C	-4.435200	0.344100	-1.744000	C	4.703000	1.188900	0.413000
C	-2.184500	-1.698000	2.156300	C	1.835100	-2.579900	-1.014500
H	4.347000	-2.839400	-1.697400	H	-3.730000	-3.157600	1.120900
H	6.036100	-1.747600	-0.258200	H	-5.715600	-2.024300	0.175000
H	5.456000	0.274100	1.074300	H	-5.535600	0.262500	-0.788700
H	2.029500	-1.929000	-1.827800	H	-1.509500	-2.012600	1.115300
H	3.338300	2.042500	1.595000	H	-3.703300	2.318300	-1.264800
H	0.335000	-0.969600	1.112900	H	0.496700	0.957500	-1.381800
H	-0.125100	-1.286500	-0.557400	H	-0.226600	-0.651800	-1.395200
H	-0.016100	1.448100	-1.549900	H	0.049800	1.727100	1.295300
H	-0.750200	1.731700	1.318500	H	0.244700	-0.429500	1.743200
H	-4.737100	1.389100	-1.640200	H	5.200500	1.410800	-0.533700
H	-5.275400	-0.312400	-1.518500	H	5.389100	0.623500	1.047800
H	-4.090600	0.175800	-2.767400	H	4.411400	2.114400	0.909200
H	-3.121600	-2.225400	2.336200	H	1.079700	-2.569900	-1.806200
H	-2.002100	-0.983100	2.963200	H	1.457100	-3.153000	-0.163100
H	-1.370000	-2.428700	2.125300	H	2.742200	-3.047500	-1.397900

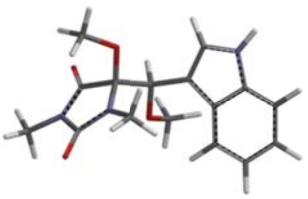

5a1 (69.1%)

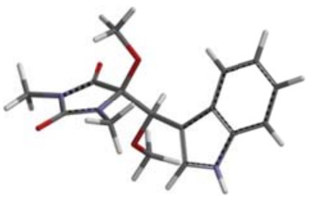

5a2 (30.3%)

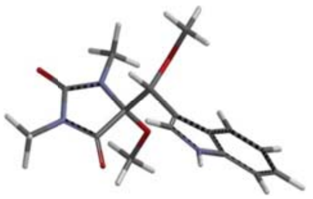

5a3 (0.3%)

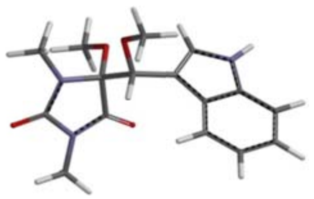

5a4 (0.3%)

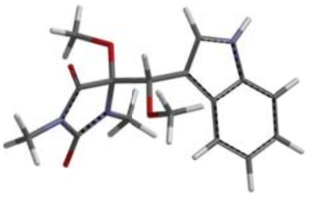

5b1 (69.1%)

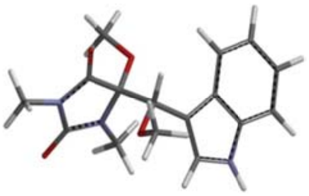

5b2 (30.3%)

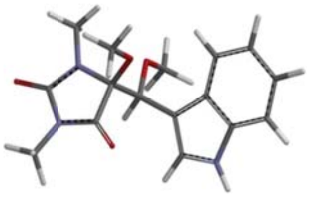

5b3 (0.3%)

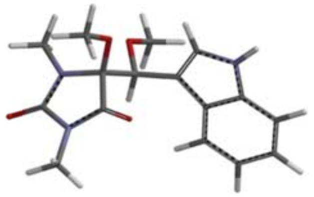

5b4 (0.3%)

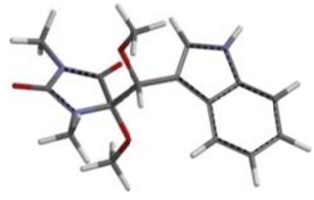

5c1 (38.9%)

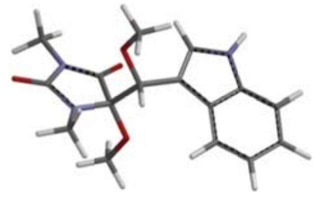

5c2 (27.7%)

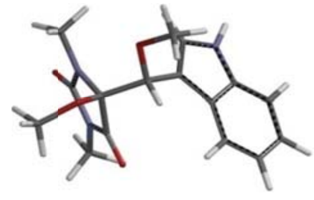

5c3 (17.4%)

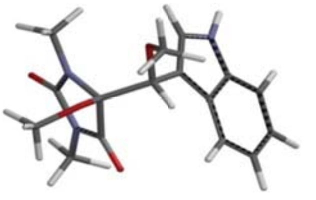

5c4 (6.9%)

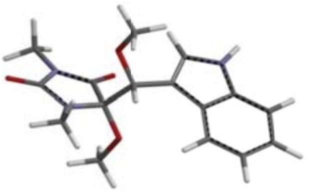

5c5 (5.6%)

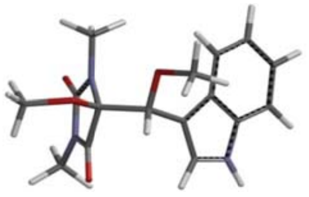

5c6 (3.4%)

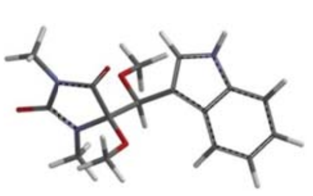

5d1 (38.9%)

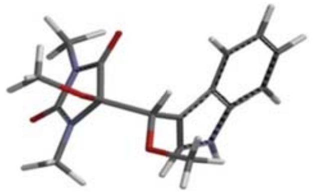

5d2 (27.7%)

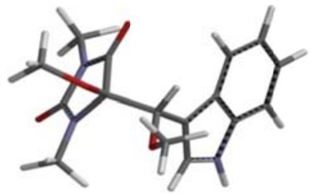

5d3 (17.4%)

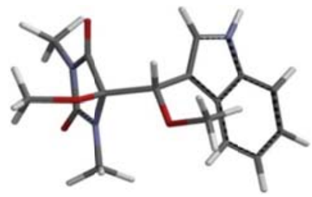

5d4 (6.9%)

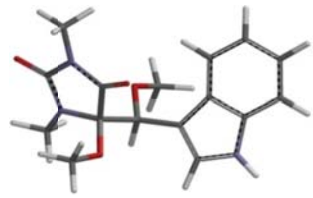

5d5 (5.6%)

Figure S4. Stable conformers of compound **5** (or **7**) with 8*R*,1′*R* (**5a**), 8*S*,1′*S* (**5b**), 8*R*,1′*S* (**5c**) and 8*S*,1′*R* (**5d**) configurations, respectively.

Table S7. Important thermodynamic parameters (a.u.) of the optimized compound **5** (or **7**) at B3LYP/6-31G(d,p) level in the gas phase

conformations	E+ZPE	G	conformations	E+ZPE	G
**5a1**	-1085.999006	-1086.052164	**5b1**	-1085.999006	-1086.052164
**5a2**	-1085.999203	-1086.051387	**5b2**	-1085.999203	-1086.051387
**5a3**	-1085.994904	-1086.046996	**5b3**	-1085.994907	-1086.046999

**5a4**	-1085.994219	-1086.046879	**5b4**	-1085.994219	-1086.046879
**5c1**	-1085.998553	-1086.051618	**5d1**	-1085.998553	-1086.051618
**5c2**	-1085.998361	-1086.051299	**5d2**	-1085.998228	-1086.050885
**5c3**	-1085.998223	-1086.050860	**5d3**	-1085.997328	-1086.049998
**5c4**	-1085.997324	-1086.049993	**5d4**	-1085.997049	-1086.049305
**5c5**	-1085.998456	-1086.049796	**5d5**	-1085.996289	-1086.048277
**5c6**	-1086.049315	-1086.049310			

Table S8. Optimized Z-Matrixes of compound **5** (or **7**) in the Gas Phase (Å) at B3LYP/6-31G(d,p) level.

5a1				5a2				5a3			
C	3.563264	2.139234	-0.035372	C	4.041400	1.757400	-0.733300	C	3.921600	-1.144800	1.328400
C	4.691178	1.405044	-0.455170	C	5.035700	1.053500	-0.023200	C	4.870300	-0.130500	1.084100
C	4.651100	0.019196	-0.544382	C	4.758800	-0.166300	0.581300	C	4.574800	0.943500	0.254000
C	3.452115	-0.615701	-0.204517	C	3.459400	-0.668700	0.453600	C	3.302100	0.978000	-0.325800
C	2.299170	0.101589	0.221796	C	2.439000	0.023500	-0.256000	C	2.327600	-0.028800	-0.089100
C	2.374836	1.506052	0.305176	C	2.750700	1.259400	-0.851400	C	2.658900	-1.105700	0.752500
N	3.126790	-1.957380	-0.198242	N	2.905600	-1.840900	0.929100	N	2.741500	1.897900	-1.191400
C	1.823440	-2.107875	0.223727	C	1.585800	-1.914900	0.535000	C	1.460900	1.502500	-1.506700
C	1.269307	-0.878501	0.487011	C	1.249900	-0.794700	-0.185800	C	1.155600	0.334200	-0.853700
C	-0.138681	-0.652036	0.968202	C	-0.093100	-0.564300	-0.823200	C	-0.146300	-0.407600	-0.966000
C	-1.248625	-0.659420	-0.122192	C	-1.024300	0.471900	-0.135700	C	-1.114200	-0.221600	0.244200
C	-2.605107	-0.443607	0.608790	C	-2.360700	0.487200	-0.932900	C	-1.641500	1.248100	0.275400
N	-3.164637	0.688338	0.079394	N	-3.353200	0.148600	-0.054200	N	-2.985600	1.190400	0.006400
C	-2.355889	1.246349	-0.938060	C	-2.843700	-0.089000	1.245900	C	-3.454600	-0.145200	-0.074500
N	-1.245114	0.450256	-1.058889	N	-1.490000	0.117100	1.195100	N	-2.367500	-0.961500	0.071400
O	-2.642874	2.236571	-1.583383	O	-3.515800	-0.395300	2.212600	O	-4.618400	-0.463400	-0.234700
C	-0.257522	0.670994	-2.104051	C	-0.663300	0.060400	2.390100	C	-2.504300	-2.410200	0.120600
C	-4.421099	1.277836	0.502683	C	-4.755200	0.003800	-0.398400	C	-3.867500	2.342800	-0.030700
O	-3.067450	-1.163684	1.473165	O	-2.484800	0.756300	-2.112500	O	-0.997200	2.254500	0.495900
O	-0.294489	0.582730	1.655136	O	-0.331600	1.694600	-0.174300	O	-0.418000	-0.596000	1.398400
C	0.163588	0.547250	2.999233	C	-1.084400	2.859100	0.159400	C	-1.045100	-0.297700	2.643400
O	-1.143995	-1.916863	-0.746782	O	-0.852200	-1.770000	-0.871800	O	0.039600	-1.812200	-1.105400
C	-2.234069	-2.295026	-1.584757	C	-0.648900	-2.521200	-2.060500	C	0.627700	-2.190100	-2.339600
H	3.627607	3.221720	0.023555	H	4.292000	2.709800	-1.191000	H	4.186800	-1.970800	1.982100
H	5.606003	1.930239	-0.712809	H	6.034600	1.472300	0.054400	H	5.848800	-0.189500	1.551700
H	5.518679	-0.549747	-0.866775	H	5.522800	-0.709700	1.130200	H	5.304400	1.725400	0.062100
H	1.511085	2.074702	0.630055	H	1.984800	1.817200	-1.378600	H	1.926600	-1.880100	0.946600
H	3.747779	-2.710181	-0.445295	H	3.398400	-2.553300	1.441900	H	3.184700	2.741700	-1.514900
H	1.372801	-3.087152	0.292624	H	0.973500	-2.768500	0.783900	H	0.847300	2.100700	-2.165300
H	-0.435308	-1.483041	1.624875	H	0.040800	-0.159400	-1.836700	H	-0.697500	-0.024400	-1.842600

H	0.589734	1.264426	-1.751190	H	-1.270800	0.363200	3.245400	H	-3.486800	-2.646600	0.534100
H	0.109815	-0.295839	-2.454172	H	0.175800	0.747300	2.268000	H	-1.719600	-2.812500	0.761400
H	-0.745609	1.201431	-2.923670	H	-0.273100	-0.944700	2.572000	H	-2.411200	-2.863500	-0.869400
H	-4.247233	2.179614	1.097217	H	-5.285300	-0.283600	0.509600	H	-3.883000	2.848600	0.939000
H	-5.016702	1.543819	-0.372390	H	-4.880400	-0.766300	-1.164400	H	-4.866000	1.980800	-0.275500
H	-4.943666	0.537689	1.109547	H	-5.154000	0.947000	-0.781000	H	-3.527600	3.052800	-0.788600
H	-0.394147	-0.195365	3.586824	H	-0.371100	3.684300	0.178200	H	-2.041100	-0.752500	2.715500
H	1.235606	0.319779	3.056732	H	-1.553600	2.768700	1.146300	H	-1.122800	0.784200	2.800200
H	-0.013194	1.540166	3.418115	H	-1.853600	3.064800	-0.594500	H	-0.400600	-0.724200	3.413200
H	-3.148432	-2.455398	-1.001450	H	-1.241100	-3.434000	-1.962400	H	1.631400	-1.765500	-2.461300
H	-2.424107	-1.548658	-2.365534	H	-0.990800	-1.963400	-2.942600	H	0.005800	-1.876700	-3.192300
H	-1.941548	-3.234996	-2.055887	H	0.407100	-2.792000	-2.192200	H	0.696800	-3.279900	-2.332300
**5a4**				**5b1**				**5b2**			
C	3.801800	1.593400	-1.481000	C	3.563300	-2.139200	-0.035400	C	-4.041300	1.757300	-0.733800
C	4.824900	1.360900	-0.539700	C	4.691200	-1.405000	-0.455200	C	-5.035600	1.053800	-0.023300
C	4.629500	0.500400	0.533900	C	4.651100	-0.019200	-0.544400	C	-4.758800	-0.165800	0.581600
C	3.382700	-0.122500	0.643100	C	3.452100	0.615700	-0.204500	C	-3.459400	-0.668400	0.453900
C	2.333700	0.095600	-0.292100	C	2.299200	-0.101600	0.221800	C	-2.439100	0.023500	-0.256100
C	2.564800	0.972500	-1.367000	C	2.374800	-1.506100	0.305200	C	-2.750800	1.259200	-0.851900
N	2.908700	-1.018200	1.581200	N	3.126800	1.957400	-0.198200	N	-2.905800	-1.840600	0.929500
C	1.611200	-1.361600	1.276600	C	1.823400	2.107900	0.223700	C	-1.585900	-1.914600	0.535800
C	1.211700	-0.704700	0.137600	C	1.269300	0.878500	0.487000	C	-1.250000	-0.794600	-0.185600
C	-0.116900	-0.833400	-0.553100	C	-0.138700	0.652000	0.968200	C	0.093000	-0.564600	-0.823100
C	-1.340500	-0.406900	0.308200	C	-1.248600	0.659400	-0.122200	C	1.024400	0.471600	-0.135800
C	-1.226000	1.099300	0.701000	C	-2.605100	0.443600	0.608800	C	2.360700	0.486700	-0.933000
N	-2.223900	1.757400	0.026100	N	-3.164600	-0.688300	0.079400	N	3.353200	0.148300	-0.054300
C	-3.085700	0.860000	-0.652300	C	-2.355900	-1.246300	-0.938100	C	2.843800	-0.088800	1.245900
N	-2.585400	-0.399600	-0.461800	N	-1.245100	-0.450300	-1.058900	N	1.490000	0.116900	1.195000
O	-4.083000	1.186000	-1.269100	O	-2.642900	-2.236600	-1.583400	O	3.516000	-0.394400	2.212800
C	-3.246600	-1.575300	-1.009200	C	-0.257500	-0.671000	-2.104100	C	0.663600	0.061300	2.390100
C	-2.487800	3.181000	0.133700	C	-4.421100	-1.277800	0.502700	C	4.755300	0.004000	-0.398500
O	-0.425800	1.595500	1.467300	O	-3.067500	1.163700	1.473200	O	2.484800	0.755600	-2.112700
O	-1.392400	-1.268300	1.414900	O	-0.294500	-0.582700	1.655100	O	0.852000	-1.770400	-0.871600
C	-2.332900	-0.928900	2.432000	C	0.163600	-0.547300	2.999200	C	0.648100	-2.522100	-2.059900
O	-0.400200	-2.163000	-0.987400	O	-1.144000	1.916900	-0.746800	O	0.331700	1.694400	-0.174600
C	0.469700	-2.625200	-2.006200	C	-2.234100	2.295000	-1.584800	C	1.084800	2.858900	0.158700
H	3.986500	2.272300	-2.308200	H	3.627600	-3.221700	0.023600	H	-4.291900	2.709600	-1.191900
H	5.780900	1.862800	-0.654600	H	5.606000	-1.930200	-0.712800	H	-6.034500	1.472800	0.054300
H	5.416300	0.320000	1.261000	H	5.518700	0.549700	-0.866800	H	-5.522800	-0.708900	1.130700
H	1.787800	1.168100	-2.101100	H	1.511100	-2.074700	0.630100	H	-1.984800	1.816600	-1.379500
H	3.421000	-1.347800	2.382700	H	3.747800	2.710200	-0.445300	H	-3.398000	-2.551900	1.444400

H	1.051700	-2.042800	1.898300	H	1.372800	3.087200	0.292600	H	-0.973600	-2.768100	0.784900
H	-0.120700	-0.160900	-1.427100	H	-0.435300	1.483000	1.624900	H	-0.040900	-0.159900	-1.836700
H	-3.057800	-2.421500	-0.348400	H	0.589700	-1.264400	-1.751200	H	0.272400	-0.943400	2.572300
H	-2.878800	-1.826000	-2.007800	H	0.109800	0.295800	-2.454200	H	-0.175000	0.748900	2.268200
H	-4.317100	-1.368000	-1.062100	H	-0.745600	-1.201400	-2.923700	H	1.271600	0.363600	3.245300
H	-2.761700	3.448100	1.158600	H	-4.247200	-2.179600	1.097200	H	4.881000	-0.766700	-1.163800
H	-3.312200	3.411800	-0.540900	H	-5.016700	-1.543800	-0.372400	H	5.285600	-0.282300	0.509800
H	-1.597600	3.747800	-0.148400	H	-4.943700	-0.537700	1.109500	H	5.153500	0.947100	-0.782000
H	-3.349300	-0.841200	2.029300	H	-0.394100	0.195400	3.586800	H	0.989600	-1.964700	-2.942400
H	-2.056200	0.003800	2.936900	H	1.235600	-0.319800	3.056700	H	-0.408000	-2.792900	-2.191000
H	-2.306500	-1.748000	3.152500	H	-0.013200	-1.540200	3.418100	H	1.240300	-3.434800	-1.961800
H	1.511900	-2.665400	-1.665800	H	-3.148400	2.455400	-1.001500	H	1.853800	3.064400	-0.595500
H	0.419200	-1.987300	-2.902400	H	-2.424100	1.548700	-2.365500	H	1.554300	2.768500	1.145500
H	0.137900	-3.632100	-2.269000	H	-1.941500	3.235000	-2.055900	H	0.371500	3.684100	0.177700
**5b3**				**5b4**				**5c1**			
C	-3.921600	-1.145300	1.328100	C	-3.802000	1.593200	-1.481100	C	4.236454	-1.750303	0.061720
C	-4.870400	-0.131100	1.083900	C	-4.825100	1.360800	-0.539700	C	5.141345	-0.755892	-0.362167
C	-4.575000	0.943200	0.254100	C	-4.629600	0.500500	0.533900	C	4.736788	0.564956	-0.515337
C	-3.302300	0.978100	-0.325500	C	-3.382800	-0.122400	0.643100	C	3.402174	0.868275	-0.228918
C	-2.327600	-0.028700	-0.089000	C	-2.333800	0.095700	-0.292100	C	2.472488	-0.115747	0.205596
C	-2.658800	-1.105800	0.752300	C	-2.564900	0.972400	-1.367100	C	2.910796	-1.444282	0.340899
N	-2.741600	1.898400	-1.190600	N	-2.908800	-1.018000	1.581300	N	2.724259	2.070530	-0.284178
C	-1.461100	1.503100	-1.506200	C	-1.611100	-1.361300	1.276800	C	1.414028	1.877061	0.094011
C	-1.155700	0.334700	-0.853500	C	-1.211700	-0.704600	0.137700	C	1.211233	0.555095	0.407640
C	0.146200	-0.407100	-0.965900	C	0.116800	-0.833400	-0.553000	C	-0.069985	-0.055988	0.897056
C	1.114200	-0.221500	0.244200	C	1.340500	-0.406800	0.308100	C	-1.050163	-0.512324	-0.228298
C	1.641900	1.248100	0.275600	C	1.226100	1.099500	0.700700	C	-1.670378	0.704364	-0.975804
N	2.986300	1.190000	0.008100	N	2.224300	1.757300	0.025900	N	-3.005073	0.721157	-0.660462
C	3.454700	-0.145600	-0.074200	C	3.086000	0.859800	-0.652200	C	-3.370685	-0.370352	0.157889
N	2.367200	-0.961700	0.071400	N	2.585300	-0.399800	-0.461800	N	-2.248586	-1.144153	0.316104
O	4.618300	-0.464200	-0.235000	O	4.083400	1.185500	-1.268900	O	-4.485938	-0.572393	0.599795
C	2.503800	-2.410500	0.119600	C	3.246500	-1.575500	-1.009100	C	-2.221938	-2.248405	1.256308
C	3.868100	2.342300	-0.030800	C	2.488300	3.180900	0.133500	C	-3.948893	1.720727	-1.123899
O	0.997800	2.254800	0.495100	O	0.425900	1.596000	1.466800	O	-1.096975	1.486213	-1.709631
O	-0.040100	-1.811600	-1.105500	O	0.400100	-2.163100	-0.987200	O	-0.301481	-1.377460	-1.036117
C	-0.627800	-2.189200	-2.340000	C	-0.469800	-2.625300	-2.005900	C	-0.917685	-1.797945	-2.251574
O	0.417800	-0.595700	1.398400	O	1.392300	-1.268100	1.415000	O	-0.838289	0.819281	1.714233
C	1.045300	-0.298000	2.643400	C	2.332900	-0.928800	2.431900	C	-0.283975	1.051656	2.999067
H	-4.186700	-1.971400	1.981600	H	-3.986700	2.272000	-2.308400	H	4.583656	-2.774346	0.163221
H	-5.849000	-0.190300	1.551400	H	-5.781100	1.862700	-0.654700	H	6.170678	-1.026832	-0.577368
H	-5.304700	1.725000	0.062400	H	-5.416400	0.320100	1.261000	H	5.431357	1.331336	-0.847800

H	-1.926500	-1.880200	0.946300	H	-1.788000	1.168000	-2.101200	H	2.218329	-2.224357	0.642177
H	-3.185400	2.741500	-1.515100	H	-3.420700	-1.346700	2.383500	H	3.116380	2.949694	-0.578352
H	-0.847400	2.101700	-2.164400	H	-1.051600	-2.042500	1.898500	H	0.710927	2.695482	0.099405
H	0.697400	-0.023900	-1.842500	H	0.120500	-0.160900	-1.427200	H	0.179184	-0.971421	1.458333
H	2.411100	-2.863000	-0.870800	H	2.878600	-1.826300	-2.007600	H	-1.515395	-3.003736	0.904647
H	1.718700	-2.813000	0.759800	H	3.057700	-2.421700	-0.348300	H	-1.937428	-1.925359	2.265293
H	3.486000	-2.647300	0.533300	H	4.316900	-1.368300	-1.062100	H	-3.222959	-2.678992	1.301227
H	4.866600	1.979900	-0.275400	H	3.313000	3.411600	-0.540700	H	-4.012488	1.711526	-2.215547
H	3.884100	2.849400	0.938200	H	2.761700	3.448100	1.158600	H	-4.919593	1.477817	-0.691760
H	3.528000	3.051400	-0.789500	H	1.598300	3.747800	-0.149100	H	-3.633922	2.715909	-0.799777
H	-0.005600	-1.875600	-3.192400	H	-0.419200	-1.987800	-2.902300	H	-1.889731	-2.271602	-2.067552
H	-1.631400	-1.764300	-2.461900	H	-1.512100	-2.665200	-1.665600	H	-1.042513	-0.956714	-2.942632
H	-0.697200	-3.279000	-2.333000	H	-0.138300	-3.632500	-2.268300	H	-0.239538	-2.528397	-2.695177
H	1.123200	0.783800	2.800700	H	2.057000	0.004300	2.936300	H	-0.176076	0.113433	3.563742
H	2.041100	-0.753100	2.715100	H	3.349400	-0.842100	2.029200	H	0.697304	1.537878	2.938176
H	0.400900	-0.724600	3.413200	H	2.305900	-1.747700	3.152800	H	-0.980522	1.705455	3.528131
**5c2**				**5c3**				**5c4**			
C	-4.229700	1.749700	0.088100	C	-3.747800	-0.723000	-1.580800	C	-3.644300	-0.652600	-1.605300
C	-5.136300	0.763100	-0.350300	C	-4.600100	-0.795100	-0.459900	C	-4.576600	-0.520400	-0.556300
C	-4.734000	-0.556100	-0.522600	C	-4.194600	-0.322800	0.782000	C	-4.233000	0.113400	0.631100
C	-3.400000	-0.865800	-0.240300	C	-2.913500	0.231200	0.872900	C	-2.931400	0.612600	0.742900
C	-2.468700	0.110200	0.208600	C	-2.036800	0.318400	-0.243700	C	-1.968700	0.485400	-0.298000
C	-2.904700	1.437400	0.363000	C	-2.474700	-0.177100	-1.486000	C	-2.350600	-0.160300	-1.490000
N	-2.724400	-2.068500	-0.312800	N	-2.253800	0.780100	1.954800	N	-2.327700	1.295100	1.779100
C	-1.414000	-1.882900	0.068900	C	-1.005900	1.209800	1.560500	C	-1.032200	1.605500	1.428900
C	-1.208800	-0.566000	0.401700	C	-0.821800	0.948900	0.223000	C	-0.758600	1.125500	0.171200
C	0.073400	0.033500	0.902300	C	0.407200	1.312100	-0.570700	C	0.550300	1.309000	-0.549600
C	1.049200	0.523500	-0.213500	C	1.534200	0.241400	-0.521900	C	1.536200	0.102400	-0.405000
C	1.658700	-0.671300	-1.004200	C	0.952400	-1.143500	-0.915300	C	0.874500	-1.202100	-0.917100
N	2.996500	-0.704000	-0.693800	N	1.108700	-1.962400	0.169800	N	0.763600	-2.040100	0.157600
C	3.368800	0.355600	0.158700	C	1.785900	-1.309100	1.231200	C	1.359500	-1.486000	1.319900
N	2.252100	1.133600	0.344000	N	2.047800	-0.030800	0.814600	N	1.848300	-0.254500	0.971200
O	4.487600	0.532600	0.601400	O	2.075700	-1.826900	2.293400	O	1.423000	-2.040500	2.400600
C	2.234600	2.202500	1.324000	C	2.814300	0.907000	1.618600	C	2.552900	0.594200	1.916900
C	3.939600	-1.692100	-1.183300	C	0.675400	-3.346400	0.233600	C	0.149700	-3.355400	0.124300
O	1.072200	-1.420100	-1.761500	O	0.468600	-1.437200	-1.992300	O	0.534300	-1.428900	-2.064200
O	0.297600	1.415400	-0.988500	O	2.524200	0.690800	-1.413600	O	2.673600	0.464900	-1.152300
C	0.907400	1.873800	-2.193500	C	3.566000	-0.233900	-1.715200	C	3.653000	-0.553400	-1.346300
O	0.845300	-0.863500	1.691900	O	0.994500	2.523600	-0.112700	O	1.200500	2.472200	-0.048000
C	0.299700	-1.126200	2.974700	C	0.323000	3.686100	-0.567300	C	1.651100	3.382000	-1.041400
H	-4.575200	2.772700	0.204200	H	-4.093500	-1.109400	-2.535000	H	-3.944500	-1.152000	-2.521700

H	-6.165100	1.038900	-0.561700	H	-5.589000	-1.230800	-0.567700	H	-5.579300	-0.919000	-0.678000
H	-5.429800	-1.316400	-0.866400	H	-4.847700	-0.380900	1.648200	H	-4.948900	0.220200	1.441100
H	-2.210700	2.211700	0.675200	H	-1.817100	-0.158600	-2.348300	H	-1.640200	-0.290200	-2.298900
H	-3.118300	-2.942600	-0.619200	H	-2.629400	0.867300	2.884800	H	-2.770600	1.550700	2.646200
H	-0.712700	-2.702900	0.064400	H	-0.342100	1.694800	2.260300	H	-0.401300	2.180200	2.089100
H	-0.174300	0.932800	1.489700	H	0.147000	1.392900	-1.636300	H	0.369800	1.397300	-1.629000
H	1.944800	1.845500	2.320000	H	2.169200	1.601700	2.163900	H	1.890400	1.330000	2.381100
H	1.537000	2.978200	0.999600	H	3.464500	1.487900	0.962500	H	3.348700	1.126700	1.393700
H	3.240200	2.620000	1.386100	H	3.410100	0.333000	2.330100	H	2.975600	-0.046700	2.692300
H	4.254500	-2.358900	-0.375600	H	-0.395100	-3.411800	0.025300	H	-0.884400	-3.273100	-0.218900
H	4.822200	-1.196000	-1.592100	H	0.887300	-3.707700	1.239800	H	0.179800	-3.754800	1.137900
H	3.434900	-2.268000	-1.959500	H	1.214600	-3.953100	-0.499400	H	0.696300	-4.017700	-0.552800
H	1.022600	1.055700	-2.913400	H	3.187900	-1.095200	-2.278800	H	3.267800	-1.362900	-1.977100
H	1.883300	2.335700	-2.000400	H	4.074200	-0.585300	-0.808600	H	4.002400	-0.966300	-0.392200
H	0.230100	2.622400	-2.607200	H	4.281000	0.310300	-2.334400	H	4.490800	-0.073300	-1.854700
H	0.999200	-1.793000	3.483100	H	-0.719700	3.716800	-0.224000	H	0.813500	3.763800	-1.642800
H	-0.682500	-1.609900	2.909000	H	0.336100	3.749900	-1.664900	H	2.392100	2.921800	-1.703600
H	0.196800	-0.201600	3.562400	H	0.861000	4.543300	-0.155900	H	2.111000	4.220000	-0.511300
**5c5**				**5c6**				**5d1**			
C	-4.232200	1.749800	0.082600	C	-3.609400	0.390500	1.658600	C	4.236500	1.750300	0.061700
C	-5.138300	0.761300	-0.352300	C	-4.418300	-0.605800	1.075400	C	5.141300	0.755900	-0.362200
C	-4.735300	-0.558200	-0.520500	C	-4.051000	-1.219600	-0.115200	C	4.736800	-0.565000	-0.515300
C	-3.401000	-0.866300	-0.237700	C	-2.847600	-0.818100	-0.704400	C	3.402200	-0.868300	-0.228900
C	-2.470100	0.111700	0.207800	C	-2.004300	0.172700	-0.128200	C	2.472500	0.115700	0.205600
C	-2.906800	1.439200	0.358100	C	-2.415600	0.786600	1.070500	C	2.910800	1.444300	0.340900
N	-2.724600	-2.068700	-0.306600	N	-2.250600	-1.234400	-1.877600	N	2.724300	-2.070500	-0.284200
C	-1.414200	-1.881100	0.074100	C	-1.075800	-0.539200	-2.063600	C	1.414000	-1.877100	0.094000
C	-1.209700	-0.563000	0.402500	C	-0.871800	0.326800	-1.014900	C	1.211200	-0.555100	0.407600
C	0.072200	0.039700	0.900200	C	0.318200	1.243700	-0.866300	C	-0.070000	0.056000	0.897100
C	1.049600	0.520300	-0.218100	C	1.547200	0.592400	-0.171100	C	-1.050200	0.512300	-0.228300
C	1.662600	-0.681400	-0.995600	C	1.891300	-0.743100	-0.890800	C	-1.670400	-0.704400	-0.975800
N	2.999700	-0.708700	-0.683400	N	1.842500	-1.728400	0.054700	N	-3.005100	-0.721200	-0.660500
C	3.369400	0.360500	0.158700	C	1.523300	-1.211400	1.339400	C	-3.370700	0.370400	0.157900
N	2.250700	1.136900	0.336300	N	1.334300	0.135900	1.195300	N	-2.248600	1.144200	0.316100
O	4.487900	0.546300	0.598800	O	1.459000	-1.873300	2.357100	O	-4.485900	0.572400	0.599800
C	2.230200	2.217000	1.303900	C	1.119600	1.003900	2.344500	C	-2.221900	2.248400	1.256300
C	3.943200	-1.702600	-1.160400	C	2.098500	-3.134700	-0.193000	C	-3.948900	-1.720700	-1.123900
O	1.079500	-1.439100	-1.746900	O	2.166800	-0.871600	-2.070100	O	-1.097000	-1.486200	-1.709600
O	0.299000	1.404000	-1.003400	O	2.579200	1.545700	-0.278500	O	-0.301500	1.377500	-1.036100
C	0.911800	1.852200	-2.210700	C	3.883600	1.108000	0.089000	C	-0.917700	1.797900	-2.251600
O	0.843100	-0.851500	1.697500	O	0.006700	2.421500	-0.138500	O	-0.838300	-0.819300	1.714200

C	0.294800	-1.106100	2.980800	C	-0.711500	3.381300	-0.896800	C	-0.284000	-1.051700	2.999100
H	-4.578200	2.773000	0.195600	H	-3.930400	0.858500	2.584500	H	4.583700	2.774300	0.163200
H	-6.167300	1.035800	-0.564300	H	-5.345900	-0.894100	1.560600	H	6.170700	1.026800	-0.577400
H	-5.430700	-1.320000	-0.861600	H	-4.676300	-1.980900	-0.573100	H	5.431400	-1.331300	-0.847800
H	-2.213300	2.215000	0.667700	H	-1.809600	1.568300	1.512600	H	2.218300	2.224400	0.642200
H	-3.118100	-2.944200	-0.609600	H	-2.620400	-1.927300	-2.507500	H	3.116400	-2.949700	-0.578400
H	-0.712300	-2.700600	0.071500	H	-0.448800	-0.726400	-2.923500	H	0.710900	-2.695500	0.099400
H	-0.175900	0.943500	1.480400	H	0.696800	1.506400	-1.865200	H	0.179200	0.971400	1.458300
H	1.941200	1.870800	2.304000	H	0.073200	0.995700	2.661200	H	-1.515400	3.003700	0.904600
H	1.530500	2.987000	0.970500	H	1.400600	2.017800	2.062600	H	-1.937400	1.925400	2.265300
H	3.234600	2.638100	1.361400	H	1.737800	0.658500	3.176700	H	-3.223000	2.679000	1.301200
H	4.150300	-2.447300	-0.385900	H	1.374200	-3.532400	-0.909000	H	-4.012500	-1.711500	-2.215500
H	4.878700	-1.213600	-1.436500	H	2.003900	-3.654800	0.760300	H	-4.919600	-1.477800	-0.691800
H	3.497500	-2.194500	-2.025700	H	3.104600	-3.274200	-0.597400	H	-3.633900	-2.715900	-0.799800
H	1.032300	1.027400	-2.922000	H	4.264000	0.347400	-0.604200	H	-1.889700	2.271600	-2.067600
H	1.885500	2.319300	-2.018800	H	3.909700	0.713600	1.112400	H	-1.042500	0.956700	-2.942600
H	0.233500	2.594400	-2.634200	H	4.523900	1.989900	0.033100	H	-0.239500	2.528400	-2.695200
H	-0.687200	-1.590400	2.916100	H	-1.682200	2.994200	-1.232800	H	-0.176100	-0.113400	3.563700
H	0.190400	-0.177900	3.562400	H	-0.133800	3.705200	-1.774600	H	0.697300	-1.537900	2.938200
H	0.993300	-1.769600	3.494900	H	-0.876500	4.240400	-0.243000	H	-0.980500	-1.705500	3.528100
**5d2**				**5d3**				**5d4**			
C	3.747900	-0.722500	-1.581000	C	3.643500	-0.654400	-1.604700	C	3.608800	0.390200	1.659100
C	4.600200	-0.795000	-0.460000	C	4.576000	-0.520600	-0.556100	C	4.417900	-0.605800	1.075700
C	4.194600	-0.323100	0.782000	C	4.232700	0.114900	0.630400	C	4.051000	-1.219300	-0.115200
C	2.913400	0.230900	0.873000	C	2.931200	0.614300	0.741800	C	2.847700	-0.817900	-0.704400
C	2.036800	0.318500	-0.243600	C	1.968200	0.485400	-0.298600	C	2.004100	0.172600	-0.128100
C	2.474800	-0.176600	-1.486100	C	2.349800	-0.161900	-1.489800	C	2.415100	0.786300	1.070900
N	2.253600	0.779300	1.955100	N	2.327800	1.298300	1.777200	N	2.251100	-1.233700	-1.878100
C	1.005800	1.209200	1.560700	C	1.032200	1.608100	1.426900	C	1.076000	-0.539000	-2.063800
C	0.821700	0.948800	0.223200	C	0.758200	1.126200	0.170000	C	0.871700	0.326800	-1.014800
C	-0.407100	1.312200	-0.570500	C	-0.551000	1.308600	-0.550500	C	-0.318200	1.243700	-0.866200
C	-1.534200	0.241500	-0.521900	C	-1.536400	0.101800	-0.404700	C	-1.547200	0.592500	-0.170900
C	-0.952300	-1.143400	-0.915400	C	-0.874700	-1.202700	-0.916700	C	-1.891500	-0.742800	-0.890900
N	-1.108600	-1.962300	0.169600	N	-0.762500	-2.040100	0.158300	N	-1.843000	-1.728300	0.054400
C	-1.785900	-1.309100	1.231100	C	-1.357800	-1.485700	1.320800	C	-1.523200	-1.211700	1.339100
N	-2.047900	-0.030800	0.814600	N	-1.847500	-0.254700	0.971800	N	-1.334100	0.135500	1.195300
O	-2.075700	-1.827000	2.293300	O	-1.420200	-2.039600	2.401900	O	-1.458800	-1.873900	2.356600
C	-2.814400	0.906900	1.618600	C	-2.551400	0.594300	1.917800	C	-1.119400	1.003100	2.344900
C	-0.675600	-3.346400	0.233300	C	-0.147900	-3.355100	0.125200	C	-2.098300	-3.134600	-0.193900
O	-0.468600	-1.437000	-1.992500	O	-0.535500	-1.430000	-2.064000	O	-2.166800	-0.871000	-2.070300
O	-2.524200	0.691000	-1.413700	O	-2.674400	0.463600	-1.151500	O	-2.579100	1.545900	-0.277900

C	-3.565900	-0.233700	-1.715400	C	-3.653800	-0.554900	-1.344000	C	-3.883500	1.108300	0.089800
O	-0.994500	2.523700	-0.112500	O	-1.201600	2.471900	-0.049400	O	-0.006600	2.421600	-0.138500
C	-0.322600	3.686200	-0.566700	C	-1.652600	3.381000	-1.043200	C	0.711900	3.381200	-0.896900
H	4.093600	-1.108600	-2.535200	H	3.943500	-1.155100	-2.520400	H	3.929600	0.857900	2.585100
H	5.589000	-1.230600	-0.567900	H	5.578800	-0.919400	-0.677500	H	5.345500	-0.894100	1.561000
H	4.847600	-0.381500	1.648200	H	4.948900	0.223000	1.440100	H	4.676500	-1.980300	-0.573200
H	1.817300	-0.157800	-2.348500	H	1.639200	-0.292900	-2.298300	H	1.808800	1.567700	1.513000
H	2.629200	0.866400	2.885000	H	2.771000	1.555400	2.643600	H	2.619700	-1.928300	-2.506700
H	0.341900	1.693900	2.260800	H	0.401400	2.183800	2.086500	H	0.449100	-0.726100	-2.923800
H	-0.146900	1.393000	-1.636100	H	-0.371000	1.396400	-1.630000	H	-0.696900	1.506400	-1.865100
H	-3.465400	1.487100	0.962600	H	-3.347300	1.127000	1.394900	H	-1.738700	0.658300	3.176500
H	-2.169400	1.602300	2.163000	H	-1.888500	1.329900	2.381700	H	-1.399200	2.017300	2.062700
H	-3.409300	0.332900	2.330700	H	-2.973900	-0.046300	2.693500	H	-0.073300	0.994000	2.662400
H	-1.215200	-3.953000	-0.499500	H	-0.694600	-4.018000	-0.551100	H	-3.104600	-3.274700	-0.597500
H	-0.887200	-3.707700	1.239600	H	-0.177000	-3.754000	1.139100	H	-2.002600	-3.655100	0.759100
H	0.394900	-3.412100	0.024600	H	0.885900	-3.272400	-0.218700	H	-1.374400	-3.531500	-0.910700
H	-4.074300	-0.585100	-0.808900	H	-4.002500	-0.967200	-0.389400	H	-3.909400	0.713400	1.112900
H	-3.187800	-1.095100	-2.278900	H	-3.268900	-1.364900	-1.974500	H	-4.264200	0.348100	-0.603800
H	-4.280800	0.310500	-2.334800	H	-4.492000	-0.075400	-1.852400	H	-4.523600	1.990400	0.034500
H	-0.335100	3.750100	-1.664300	H	-2.393700	2.920300	-1.704800	H	0.134500	3.704800	-1.775000
H	0.719900	3.716700	-0.222700	H	-0.815200	3.762500	-1.645000	H	1.682800	2.993900	-1.232400
H	-0.860800	4.543300	-0.155500	H	-2.112400	4.219200	-0.513500	H	0.876600	4.240500	-0.243300
**5c2**											
C	-3.355800	2.145900	0.308700								
C	-4.517300	1.539800	-0.211900								
C	-4.540800	0.186400	-0.523100								
C	-3.370800	-0.547100	-0.301000								
C	-2.185900	0.040800	0.220700								
C	-2.196500	1.414300	0.527100								
N	-3.110300	-1.888000	-0.503900								
C	-1.815600	-2.162500	-0.121300								
C	-1.205100	-1.016700	0.325400								
C	0.210100	-0.938400	0.828500								
C	1.294700	-0.695100	-0.271900								
C	1.106600	0.654700	-1.016700								
N	2.140200	1.467300	-0.619300								
C	3.058500	0.785400	0.208400								
N	2.622300	-0.514200	0.305600								
O	4.055900	1.275700	0.702600								
C	3.212000	-1.430700	1.263200								
C	2.335500	2.832100	-1.071700								

O	0.234900	0.933600	-1.814500
O	1.211300	-1.826000	-1.098600
C	1.976900	-1.784300	-2.301900
O	0.406400	0.081300	1.797200
C	-0.220800	-0.179200	3.044700
H	-3.370000	3.207100	0.539000
H	-5.407900	2.140100	-0.372700
H	-5.434000	-0.284500	-0.924200
H	-1.305600	1.888200	0.923000
H	-3.759300	-2.558300	-0.881300
H	-1.411100	-3.160100	-0.213000
H	0.477900	-1.918200	1.258200
H	4.270100	-1.184600	1.359600
H	3.108400	-2.453200	0.892900
H	2.742600	-1.348800	2.250800
H	2.553300	2.859200	-2.143600
H	3.176000	3.244400	-0.513500
H	1.432900	3.417700	-0.883600
H	3.035800	-1.585500	-2.096800
H	1.586400	-1.029700	-2.993100
H	1.879500	-2.772200	-2.755300
H	0.137700	-1.124700	3.478900
H	-1.312100	-0.223400	2.951800
H	0.052200	0.643100	3.709100

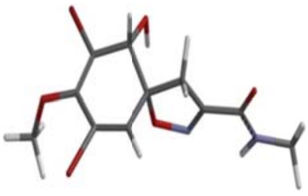

18a1 (64.8%)

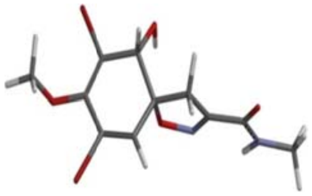

18a2 (29.7%)

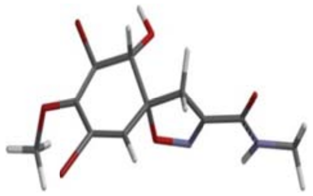

18a3 (2.9%)

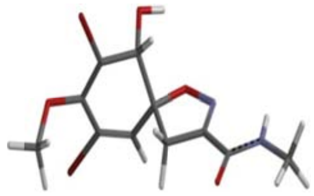

18a4 (2.6%)

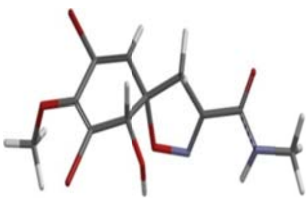

18b1 (64.8%)

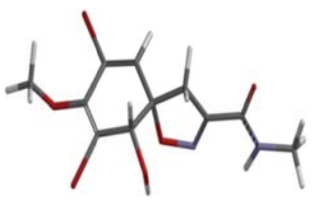

18b2 (29.7%)

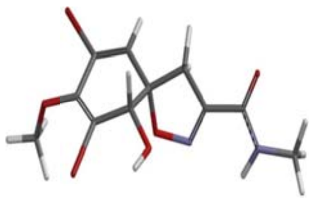

18b3 (2.9%)

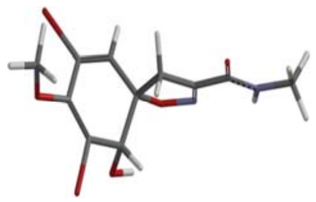

18b4 (2.6%)

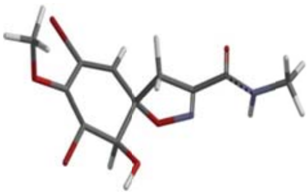

18c1 (23.8%)

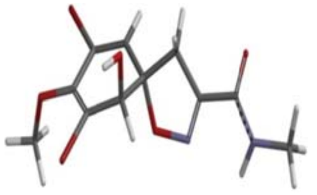

18c2 (21.6%)

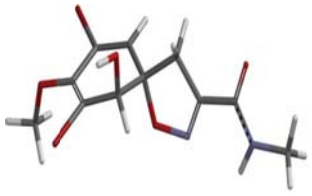

18c3 (21.5%)

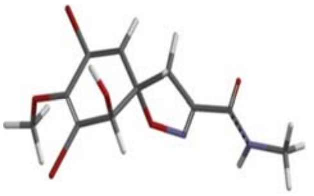

18c4 (16.9%)

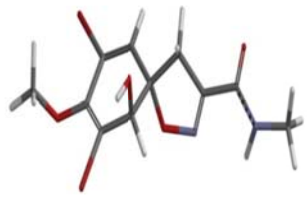

18c5 (6.5%)

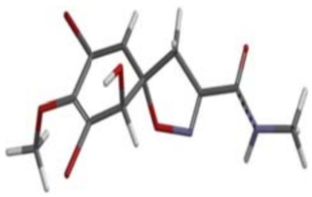

18c6 (5.3%)

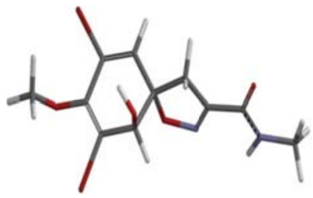

18c7 (4.4%)

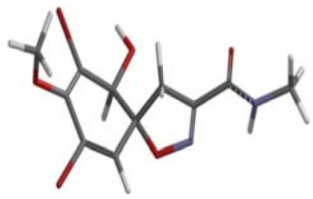

18d1 (23.8%)

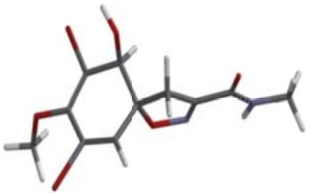

18d2 (21.6%)

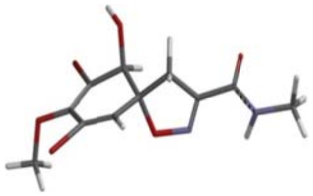

18d3 (21.5%)

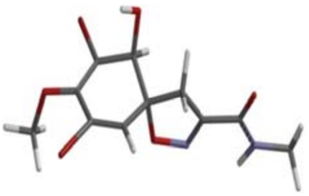

18d4 (16.9%)

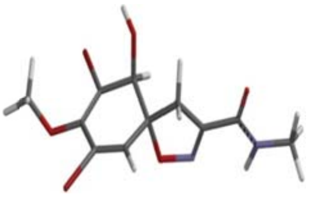

18d5 (6.5%)

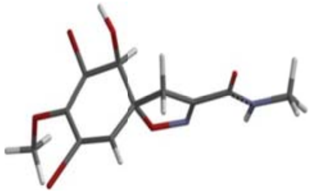

18d6 (5.3%)

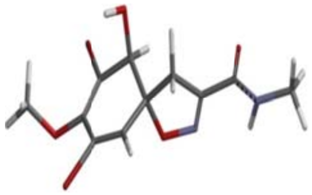

18d7 (4.4%)

Figure S5. Stable conformers of compound **18** with 1*R*,6*R* (**18a**), 1*S*,6*S* (**18b**), 1*R*,6*S* (**18c**) and 1*S*,6*R* (**18d**) configurations, respectively

Table S9. Important thermodynamic parameters (a.u.) of the optimized compound **18** at B3LYP/6-31G(d,p) level in the gas phase

conformations	E+ZPE	G	conformations	E+ZPE	G
**18a1**	-5979.895889	-5979.947626	**18b1**	-5979.895889	-5979.947626
**18a2**	-5979.895384	-5979.946889	**18b2**	-5979.895384	-5979.946889
**18a3**	-5979.892986	-5979.944711	**18b3**	-5979.892986	-5979.944711
**18a4**	-5979.892906	-5979.944605	**18b4**	-5979.892906	-5979.944605
**18c1**	-5979.894427	-5979.946581	**18d1**	-5979.894427	-5979.946581
**18c2**	-5979.894316	-5979.94649	**18d2**	-5979.894316	-5979.94649
**18c3**	-5979.894316	-5979.946488	**18d3**	-5979.894316	-5979.946488
**18c4**	-5979.894308	-5979.946262	**18d4**	-5979.894308	-5979.946262
**18c5**	-5979.893675	-5979.945352	**18d5**	-5979.893675	-5979.945352

**18c6**	-5979.894119	-5979.945161	**18d6**	-5979.894119	-5979.945161
**18c7**	-5979.893197	-5979.944995	**18d7**	-5979.893197	-5979.944995

Table S10. Optimized Z-Matrixes of compound **18** in the Gas Phase (Å) at B3LYP/6-31G(d,p) level.

**18a1**				**18a2**				**18a3**			
C	1.404032	1.104435	-0.291	C	-1.37796	1.09341	0.121389	C	-1.273155	-1.125098	-0.29188
C	-0.013078	1.137808	-0.843737	C	0.018466	1.124456	0.724857	C	0.1108	-0.98128	-0.899587
C	-0.826974	-0.105704	-0.415407	C	0.847213	-0.122348	0.333987	C	0.804601	0.307441	-0.415329
C	-0.012126	-1.368786	-0.424166	C	0.028632	-1.382064	0.302901	C	-0.14755	1.475318	-0.443548
C	1.313158	-1.317432	-0.233393	C	-1.286932	-1.326729	0.055564	C	-1.455217	1.282077	-0.222511
C	2.05495	-0.051279	-0.059469	C	-2.019832	-0.059494	-0.147922	C	-2.045982	-0.058014	-0.024221
O	-1.235057	0.139449	1.007166	O	1.324476	0.120911	-1.06616	O	1.193363	0.12681	1.008024
N	-2.617882	0.08676	1.120132	N	2.711322	0.079122	-1.109475	N	2.568948	0.110541	1.125419
C	-3.149156	-0.114953	-0.028024	C	3.186135	-0.122896	0.063009	C	3.11807	0.327835	-0.011552
C	-2.1714	-0.206162	-1.162039	C	2.153347	-0.226587	1.145847	C	2.148652	0.535626	-1.138217
Br	2.335795	-2.923223	-0.156545	Br	-2.304621	-2.932487	-0.077344	Br	-2.652787	2.762968	-0.148087
Br	2.285714	2.765141	-0.079807	Br	-2.244469	2.761764	-0.107041	Br	-1.960433	-2.885808	-0.102709
C	-4.630599	-0.231798	-0.212974	C	4.65705	-0.229831	0.321715	C	4.601423	0.389136	-0.183618
O	-5.08874	-0.445002	-1.332471	O	5.059789	-0.447412	1.46163	O	5.079721	0.655631	-1.28403
N	-5.365604	-0.089255	0.920294	N	5.447043	-0.073466	-0.77194	N	5.321609	0.138625	0.941322
O	-0.72488	2.311369	-0.539065	O	0.742865	2.295393	0.44077	O	0.986323	-2.051946	-0.652455
C	-6.813318	-0.178885	0.928433	C	6.894017	-0.151777	-0.707702	C	6.770901	0.164013	0.962477
O	3.373596	-0.099175	0.276304	O	-3.279841	-0.117357	-0.662582	O	-3.347203	-0.171262	0.357633
C	3.634909	-0.188372	1.686182	C	-4.344497	-0.061246	0.301327	C	-3.57186	-0.050676	1.772795
H	0.065937	1.115484	-1.940564	H	-0.099125	1.108099	1.818419	H	-0.044571	-0.846163	-1.989032
H	-0.530957	-2.317209	-0.503807	H	0.541678	-2.332452	0.395985	H	0.26303	2.473575	-0.542759
H	-2.322484	0.624783	-1.857868	H	2.267115	0.597453	1.85676	H	2.321826	-0.181452	-1.944364
H	-2.287744	-1.135819	-1.725098	H	2.243932	-1.161638	1.704888	H	2.245296	1.539517	-1.563511
H	-4.86262	0.079392	1.780519	H	4.986807	0.097832	-1.655312	H	4.803701	-0.070699	1.783598
H	-0.876628	2.297919	0.41908	H	0.933202	2.27436	-0.510491	H	0.485463	-2.877727	-0.727203
H	-7.155231	-0.997348	1.571341	H	7.274553	-0.960191	-1.341507	H	7.143925	0.917517	1.665081
H	-7.138054	-0.370372	-0.094546	H	7.168166	-0.352263	0.328262	H	7.110944	0.415559	-0.042483
H	-7.265563	0.755655	1.277899	H	7.35567	0.7901	-1.023631	H	7.181021	-0.812189	1.244201
H	4.720306	-0.220169	1.788007	H	-5.269415	-0.0989	-0.275432	H	-4.647481	-0.160862	1.914941
H	3.201886	-1.101154	2.109306	H	-4.304124	0.873437	0.869174	H	-3.253214	0.930507	2.139557
H	3.240987	0.692809	2.203152	H	-4.301006	-0.921767	0.97715	H	-3.042875	-0.844475	2.310434
**18a4**				**18b1**				**18b2**			
C	-1.090059	-1.183009	0.259052	C	1.404032	-1.104435	-0.291	C	-1.37796	-1.09341	0.121389
C	0.175024	-0.919167	1.037005	C	-0.013078	-1.137808	-0.843737	C	0.018466	-1.124456	0.724857
C	0.768271	0.459859	0.642159	C	-0.826974	0.105704	-0.415407	C	0.847213	0.122348	0.333987
C	-0.283369	1.532342	0.565544	C	-0.012126	1.368786	-0.424166	C	0.028632	1.382064	0.302901

C	-1.516986	1.207609	0.164011	C	1.313158	1.317432	-0.233393	C	-1.286932	1.326729	0.055564
C	-1.919309	-0.189929	-0.11524	C	2.05495	0.051279	-0.059469	C	-2.019832	0.059494	-0.147922
O	1.719303	0.815049	1.715638	O	-1.235057	-0.139449	1.007166	O	1.324476	-0.120911	-1.06616
N	3.027173	0.675021	1.254708	N	-2.617882	-0.08676	1.120132	N	2.711322	-0.079122	-1.109475
C	3.022672	0.428492	-0.002555	C	-3.149156	0.114953	-0.028024	C	3.186135	0.122896	0.063009
C	1.656385	0.373431	-0.621522	C	-2.1714	0.206162	-1.162039	C	2.153347	0.226587	1.145847
Br	-2.853525	2.549092	-0.052364	Br	2.335795	2.923223	-0.156545	Br	-2.304621	2.932487	-0.077344
Br	-1.547048	-2.996664	-0.038427	Br	2.285714	-2.765141	-0.079807	Br	-2.244469	-2.761764	-0.107041
C	4.287877	0.258151	-0.788283	C	-4.630599	0.231798	-0.212974	C	4.65705	0.229831	0.321715
O	4.226502	0.039556	-1.995075	O	-5.08874	0.445002	-1.332471	O	5.059789	0.447412	1.46163
N	5.432757	0.365483	-0.066652	N	-5.365604	0.089255	0.920294	N	5.447043	0.073466	-0.77194
O	-0.148495	-0.970562	2.412932	O	-0.72488	-2.311369	-0.539065	O	0.742865	-2.295393	0.44077
C	6.748344	0.23536	-0.664765	C	-6.813318	0.178885	0.928433	C	6.894017	0.151777	-0.707702
O	-3.133107	-0.431883	-0.677667	O	3.373596	0.099175	0.276304	O	-3.279841	0.117357	-0.662582
C	-3.184326	-0.288492	-2.105483	C	3.634909	0.188372	1.686182	C	-4.344497	0.061246	0.301327
H	0.921947	-1.685955	0.781602	H	0.065937	-1.115484	-1.940564	H	-0.099125	-1.108099	1.818419
H	0.008175	2.55297	0.784009	H	-0.530957	2.317209	-0.503807	H	0.541678	2.332452	0.395985
H	1.497246	-0.544139	-1.194279	H	-2.322484	-0.624783	-1.857868	H	2.267115	-0.597453	1.85676
H	1.509596	1.213415	-1.308296	H	-2.287744	1.135819	-1.725098	H	2.243932	1.161638	1.704888
H	5.342289	0.556698	0.921689	H	-4.86262	-0.079392	1.780519	H	4.986807	-0.097832	-1.655312
H	0.629642	-0.636004	2.885118	H	-0.876628	-2.297919	0.41908	H	0.933202	-2.27436	-0.510491
H	7.326477	1.159075	-0.553077	H	-7.155231	0.997348	1.571341	H	7.274553	0.960191	-1.341507
H	6.60999	0.02836	-1.726098	H	-7.138054	0.370372	-0.094546	H	7.168166	0.352263	0.328262
H	7.308562	-0.588096	-0.209067	H	-7.265563	-0.755655	1.277899	H	7.35567	-0.7901	-1.023631
H	-4.214074	-0.505338	-2.391378	H	4.720306	0.220169	1.788007	H	-5.269415	0.0989	-0.275432
H	-2.510371	-1.006852	-2.584537	H	3.201886	1.101154	2.109306	H	-4.304124	-0.873437	0.869174
H	-2.929714	0.732191	-2.410542	H	3.240987	-0.692809	2.203152	H	-4.301006	0.921767	0.97715
**18b3**				**18b4**				**18c1**			
C	-1.273155	1.125098	-0.29188	C	-1.090059	1.183009	0.259052	C	-1.270207	-1.135383	-0.271849
C	0.1108	0.98128	-0.899587	C	0.175024	0.919167	1.037005	C	0.06121	-1.042117	-1.004871
C	0.804601	-0.307441	-0.415329	C	0.768271	-0.459859	0.642159	C	0.792101	0.274671	-0.666352
C	-0.14755	-1.475318	-0.443548	C	-0.283369	-1.532342	0.565544	C	-0.158827	1.444693	-0.614022
C	-1.455217	-1.282077	-0.222511	C	-1.516986	-1.207609	0.164011	C	-1.434489	1.277208	-0.248658
C	-2.045982	0.058014	-0.024221	C	-1.919309	0.189929	-0.11524	C	-2.009075	-0.055624	0.038454
O	1.193363	-0.12681	1.008024	O	1.719303	-0.815049	1.715638	O	1.732189	0.512937	-1.778619
N	2.568948	-0.110541	1.125419	N	3.027173	-0.675021	1.254708	N	3.038064	0.485862	-1.316008
C	3.11807	-0.327835	-0.011552	C	3.022672	-0.428492	-0.002555	C	3.05663	0.307923	-0.04748
C	2.148652	-0.535626	-1.138217	C	1.656385	-0.373431	-0.621522	C	1.702824	0.187845	0.585504
Br	-2.652787	-2.762968	-0.148087	Br	-2.853525	-2.549092	-0.052364	Br	-2.607673	2.771752	-0.08819
Br	-1.960433	2.885808	-0.102709	Br	-1.547048	2.996664	-0.038427	Br	-1.969754	-2.884084	-0.005695
C	4.601423	-0.389136	-0.183618	C	4.287877	-0.258151	-0.788283	C	4.335425	0.251657	0.72757

O	5.079721	-0.655631	-1.28403	O	4.226502	-0.039556	-1.995075	N	5.465863	0.320398	-0.0236
N	5.321609	-0.138625	0.941322	N	5.432757	-0.365483	-0.066652	C	6.793325	0.290576	0.55855
O	0.986323	2.051946	-0.652455	O	-0.148495	0.970562	2.412932	O	4.305333	0.153493	1.951861
C	6.770901	-0.164013	0.962477	C	6.748344	-0.23536	-0.664765	O	0.944794	-2.102362	-0.719448
O	-3.347203	0.171262	0.357633	O	-3.133107	0.431883	-0.677667	O	-3.274416	-0.15063	0.529432
C	-3.57186	0.050676	1.772795	C	-3.184326	0.288492	-2.105483	C	-3.385048	0.010499	1.953137
H	-0.044571	0.846163	-1.989032	H	0.921947	1.685955	0.781602	H	-0.160667	-1.003719	-2.086183
H	0.26303	-2.473575	-0.542759	H	0.008175	-2.55297	0.784009	H	0.256964	2.425806	-0.81257
H	2.321826	0.181452	-1.944364	H	1.497246	0.544139	-1.194279	H	1.524682	0.999161	1.297307
H	2.245296	-1.539517	-1.563511	H	1.509596	-1.213415	-1.308296	H	1.590948	-0.7617	1.111782
H	4.803701	0.070699	1.783598	H	5.342289	-0.556698	0.921689	H	5.351153	0.399008	-1.024644
H	0.485463	2.877727	-0.727203	H	0.629642	0.636004	2.885118	H	7.369632	-0.563845	0.186893
H	7.143925	-0.917517	1.665081	H	7.326477	-1.159075	-0.553077	H	6.677722	0.198763	1.638814
H	7.110944	-0.415559	-0.042483	H	6.60999	-0.02836	-1.726098	H	7.345569	1.210313	0.335302
H	7.181021	0.812189	1.244201	H	7.308562	0.588096	-0.209067	H	0.4607	-2.93394	-0.828619
H	-4.647481	0.160862	1.914941	H	-4.214074	0.505338	-2.391378	H	-4.447057	-0.081131	2.183108
H	-3.253214	-0.930507	2.139557	H	-2.510371	1.006852	-2.584537	H	-3.028062	0.996905	2.267108
H	-3.042875	0.844475	2.310434	H	-2.929714	-0.732191	-2.410542	H	-2.825535	-0.776996	2.469253
**18c2**				**18c3**				**18c4**			
C	1.216822	1.153028	0.141569	C	-1.217044	-1.152948	0.141452	C	1.199725	1.161138	0.178463
C	-0.207949	1.053313	0.618461	C	0.207778	-1.053607	0.61817	C	-0.211383	1.021251	0.684603
C	-0.842603	-0.32062	0.283068	C	0.842691	0.320263	0.283146	C	-0.838445	-0.350815	0.292575
C	0.128977	-1.463147	0.350884	C	-0.12864	1.462996	0.35107	C	0.141008	-1.487039	0.340388
C	1.437124	-1.256494	0.154925	C	-1.436848	1.256652	0.155205	C	1.446824	-1.255572	0.149192
C	2.017148	0.088032	-0.040925	C	-2.017179	-0.087728	-0.040619	C	2.005705	0.101637	-0.031422
O	-1.264933	-0.223215	-1.146258	O	1.265098	0.223159	-1.146241	O	-1.249376	-0.209119	-1.135684
N	-2.645712	-0.144874	-1.2193	N	2.645868	0.144819	-1.219266	N	-2.629003	-0.122008	-1.213935
C	-3.163128	-0.308403	-0.057719	C	3.163251	0.308025	-0.057641	C	-3.154725	-0.324058	-0.061911
C	-2.166054	-0.548156	1.03915	C	2.166148	0.547398	1.039304	C	-2.164561	-0.603052	1.031826
Br	2.654824	-2.722694	0.151067	Br	-2.654205	2.723114	0.15101	Br	2.688967	-2.700673	0.118373
Br	1.900148	2.923316	-0.009971	Br	-1.900926	-2.922998	-0.010289	Br	1.860633	2.929188	0.002282
C	-4.643857	-0.30349	0.161375	C	4.643975	0.30284	0.161501	C	-4.636472	-0.327612	0.14905
N	-5.388855	-0.079055	-0.953014	N	5.388994	0.079661	-0.95313	N	-5.375375	-0.048593	-0.956745
C	-6.838576	-0.054408	-0.930717	C	6.838713	0.054617	-0.930799	C	-6.825312	-0.02612	-0.941462
O	-5.096231	-0.495345	1.287076	O	5.096314	0.493449	1.287426	O	-5.094134	-0.574796	1.261814
O	-0.269776	1.234997	2.031506	O	0.269691	-1.235542	2.031239	O	-0.26592	1.266598	2.086289
O	3.341858	0.211624	-0.322084	O	-3.342021	-0.210817	-0.321458	O	3.324232	0.236604	-0.335859
C	3.682125	0.077342	-1.712342	C	-3.682267	-0.077721	-1.711859	C	3.630419	0.164938	-1.73901
H	-0.811066	1.8117	0.101438	H	0.810747	-1.812031	0.101045	H	-0.841075	1.790296	0.229814
H	-0.265576	-2.463207	0.488442	H	0.2662	2.46297	0.488431	H	-0.238064	-2.494981	0.46489
H	-2.29102	0.135994	1.879534	H	2.261532	1.564514	1.433999	H	-2.287956	0.057743	1.892226

H	-2.261444	-1.56539	1.433532	H	2.291177	-0.137007	1.879461	H	-2.267706	-1.631761	1.393856
H	-4.891536	0.052627	-1.822954	H	4.891688	-0.051307	-1.823184	H	-4.87365	0.125249	-1.816693
H	-7.258945	-0.844689	-1.562654	H	7.222976	-0.911805	-1.275076	H	-7.24124	-0.785929	-1.61252
H	-7.15554	-0.217542	0.099655	H	7.259254	0.845078	-1.56238	H	-7.208471	0.955319	-1.241353
H	-7.223014	0.912078	-1.274587	H	7.155666	0.217194	0.099657	H	-7.147943	-0.238107	0.078184
H	0.207387	2.051826	2.237506	H	-0.208296	-2.051892	2.237215	H	0.445488	0.761692	2.506039
H	4.764722	0.196212	-1.767262	H	-3.193557	-0.86171	-2.29982	H	3.34214	-0.806361	-2.154524
H	3.403309	-0.910966	-2.092057	H	-4.764903	-0.19627	-1.766672	H	4.711274	0.287511	-1.81513
H	3.193158	0.860629	-2.301035	H	-3.403075	0.910151	-2.092446	H	3.12672	0.973003	-2.279178
**18c5**				**18c6**				**18c7**			
C	1.207883	1.125773	-0.047522	C	1.213753	1.153091	0.141959	C	1.182387	1.137551	0.004337
C	-0.20281	1.05619	0.474611	C	-0.211887	1.04836	0.615025	C	-0.206443	1.011533	0.574053
C	-0.865554	-0.319781	0.206081	C	-0.841096	-0.327712	0.277587	C	-0.861929	-0.35743	0.222146
C	0.09619	-1.471325	0.245624	C	0.134177	-1.46697	0.347258	C	0.111779	-1.49957	0.235667
C	1.397328	-1.284401	-0.007952	C	1.442143	-1.255733	0.154985	C	1.409384	-1.279002	-0.014865
C	1.989258	0.048909	-0.241663	C	2.018132	0.09081	-0.038501	C	1.970816	0.071414	-0.237546
O	-1.361653	-0.250332	-1.200924	O	-1.260667	-0.231392	-1.15227	O	-1.338491	-0.224755	-1.185467
N	-2.743125	-0.158305	-1.203459	N	-2.641748	-0.150684	-1.22752	N	-2.719015	-0.124616	-1.199306
C	-3.200811	-0.291335	-0.013327	C	-3.16077	-0.315791	-0.066794	C	-3.191984	-0.313283	-0.022482
C	-2.14981	-0.518873	1.034723	C	-2.165411	-0.559541	1.030825	C	-2.153644	-0.594732	1.024987
Br	2.588124	-2.771383	-0.072707	Br	2.66483	-2.717797	0.15263	Br	2.636112	-2.735836	-0.094756
Br	1.904483	2.889546	-0.235417	Br	1.891756	2.925621	-0.007309	Br	1.836826	2.904524	-0.215471
C	-4.667387	-0.263333	0.283861	C	-4.64157	-0.306428	0.14909	C	-4.661425	-0.298257	0.260046
N	-5.468484	-0.054154	-0.793907	N	-5.391729	-0.083405	-0.958825	N	-5.450585	-0.022653	-0.811207
C	-6.91452	-0.010069	-0.69485	C	-6.84203	-0.055216	-0.892431	C	-6.897588	0.019112	-0.724559
O	-5.060784	-0.423698	1.436459	O	-5.106842	-0.494217	1.270275	O	-5.06657	-0.527765	1.396852
O	-0.211686	1.288867	1.882553	O	-0.277806	1.229469	2.02808	O	-0.193758	1.26631	1.97592
O	3.274109	0.128048	-0.68303	O	3.343262	0.218659	-0.315941	O	3.235718	0.170625	-0.728713
C	4.267468	0.297452	0.343138	C	3.687102	0.089213	-1.70578	C	4.267844	0.382138	0.249211
H	-0.814422	1.804853	-0.046261	H	-0.816308	1.80491	0.09686	H	-0.849014	1.784754	0.145374
H	-0.30618	-2.465716	0.400709	H	-0.257355	-2.468478	0.482817	H	-0.268562	-2.504286	0.380705
H	-2.225743	0.18298	1.866169	H	-2.2921	0.120594	1.874173	H	-2.233235	0.071003	1.886709
H	-2.232772	-1.52741	1.45354	H	-2.260467	-1.578857	1.420087	H	-2.247429	-1.621076	1.396348
H	-5.017412	0.050021	-1.692365	H	-4.912658	0.052284	-1.837306	H	-4.990079	0.135386	-1.696934
H	-7.377748	-0.820358	-1.268998	H	-7.237368	0.108127	-1.896129	H	-7.356175	-0.753544	-1.35182
H	-7.177067	-0.125719	0.357021	H	-7.23408	-0.999699	-0.502275	H	-7.282829	0.996856	-1.033412
H	-7.305743	0.94624	-1.058637	H	-7.190277	0.748795	-0.235988	H	-7.171385	-0.159698	0.315525
H	0.244888	2.129266	2.033635	H	0.196987	2.047283	2.235607	H	0.503032	0.722095	2.370187
H	5.224666	0.350647	-0.176645	H	3.196669	0.872254	-2.293569	H	4.094459	1.311751	0.799774
H	4.096102	1.226647	0.895271	H	3.412566	-0.899116	-2.088592	H	5.198735	0.458509	-0.313583
H	4.268169	-0.558263	1.025644	H	4.76939	0.212038	-1.758018	H	4.32648	-0.467126	0.937957

18d1				18d2				18d3			
C	-1.270207	1.135383	-0.271849	C	1.216822	-1.153028	0.141569	C	-1.217044	1.152948	0.141452
C	0.06121	1.042117	-1.004871	C	-0.207949	-1.053313	0.618461	C	0.207778	1.053607	0.61817
C	0.792101	-0.274671	-0.666352	C	-0.842603	0.32062	0.283068	C	0.842691	-0.320263	0.283146
C	-0.158827	-1.444693	-0.614022	C	0.128977	1.463147	0.350884	C	-0.12864	-1.462996	0.35107
C	-1.434489	-1.277208	-0.248658	C	1.437124	1.256494	0.154925	C	-1.436848	-1.256652	0.155205
C	-2.009075	0.055624	0.038454	C	2.017148	-0.088032	-0.040925	C	-2.017179	0.087728	-0.040619
O	1.732189	-0.512937	-1.778619	O	-1.264933	0.223215	-1.146258	O	1.265098	-0.223159	-1.146241
N	3.038064	-0.485862	-1.316008	N	-2.645712	0.144874	-1.2193	N	2.645868	-0.144819	-1.219266
C	3.05663	-0.307923	-0.04748	C	-3.163128	0.308403	-0.057719	C	3.163251	-0.308025	-0.057641
C	1.702824	-0.187845	0.585504	C	-2.166054	0.548156	1.03915	C	2.166148	-0.547398	1.039304
Br	-2.607673	-2.771752	-0.08819	Br	2.654824	2.722694	0.151067	Br	-2.654205	-2.723114	0.15101
Br	-1.969754	2.884084	-0.005695	Br	1.900148	-2.923316	-0.009971	Br	-1.900926	2.922998	-0.010289
C	4.335425	-0.251657	0.72757	C	-4.643857	0.30349	0.161375	C	4.643975	-0.30284	0.161501
N	5.465863	-0.320398	-0.0236	N	-5.388855	0.079055	-0.953014	N	5.388994	-0.079661	-0.95313
C	6.793325	-0.290576	0.55855	C	-6.838576	0.054408	-0.930717	C	6.838713	-0.054617	-0.930799
O	4.305333	-0.153493	1.951861	O	-5.096231	0.495345	1.287076	O	5.096314	-0.493449	1.287426
O	0.944794	2.102362	-0.719448	O	-0.269776	-1.234997	2.031506	O	0.269691	1.235542	2.031239
O	-3.274416	0.15063	0.529432	O	3.341858	-0.211624	-0.322084	O	-3.342021	0.210817	-0.321458
C	-3.385048	-0.010499	1.953137	C	3.682125	-0.077342	-1.712342	C	-3.682267	0.077721	-1.711859
H	-0.160667	1.003719	-2.086183	H	-0.811066	-1.8117	0.101438	H	0.810747	1.812031	0.101045
H	0.256964	-2.425806	-0.81257	H	-0.265576	2.463207	0.488442	H	0.2662	-2.46297	0.488431
H	1.524682	-0.999161	1.297307	H	-2.29102	-0.135994	1.879534	H	2.261532	-1.564514	1.433999
H	1.590948	0.7617	1.111782	H	-2.261444	1.56539	1.433532	H	2.291177	0.137007	1.879461
H	5.351153	-0.399008	-1.024644	H	-4.891536	-0.052627	-1.822954	H	4.891688	0.051307	-1.823184
H	7.369632	0.563845	0.186893	H	-7.258945	0.844689	-1.562654	H	7.222976	0.911805	-1.275076
H	6.677722	-0.198763	1.638814	H	-7.15554	0.217542	0.099655	H	7.259254	-0.845078	-1.56238
H	7.345569	-1.210313	0.335302	H	-7.223014	-0.912078	-1.274587	H	7.155666	-0.217194	0.099657
H	0.4607	2.93394	-0.828619	H	0.207387	-2.051826	2.237506	H	-0.208296	2.051892	2.237215
H	-4.447057	0.081131	2.183108	H	4.764722	-0.196212	-1.767262	H	-3.193557	0.86171	-2.29982
H	-3.028062	-0.996905	2.267108	H	3.403309	0.910966	-2.092057	H	-4.764903	0.19627	-1.766672
H	-2.825535	0.776996	2.469253	H	3.193158	-0.860629	-2.301035	H	-3.403075	-0.910151	-2.092446
18d4				18d5				18d6			
C	1.199725	-1.161138	0.178463	C	1.207883	-1.125773	-0.047522	C	1.213753	-1.153091	0.141959
C	-0.211383	-1.021251	0.684603	C	-0.20281	-1.05619	0.474611	C	-0.211887	-1.04836	0.615025
C	-0.838445	0.350815	0.292575	C	-0.865554	0.319781	0.206081	C	-0.841096	0.327712	0.277587
C	0.141008	1.487039	0.340388	C	0.09619	1.471325	0.245624	C	0.134177	1.46697	0.347258
C	1.446824	1.255572	0.149192	C	1.397328	1.284401	-0.007952	C	1.442143	1.255733	0.154985
C	2.005705	-0.101637	-0.031422	C	1.989258	-0.048909	-0.241663	C	2.018132	-0.09081	-0.038501
O	-1.249376	0.209119	-1.135684	O	-1.361653	0.250332	-1.200924	O	-1.260667	0.231392	-1.15227
N	-2.629003	0.122008	-1.213935	N	-2.743125	0.158305	-1.203459	N	-2.641748	0.150684	-1.22752

C	-3.154725	0.324058	-0.061911	C	-3.200811	0.291335	-0.013327	C	-3.16077	0.315791	-0.066794
C	-2.164561	0.603052	1.031826	C	-2.14981	0.518873	1.034723	C	-2.165411	0.559541	1.030825
Br	2.688967	2.700673	0.118373	Br	2.588124	2.771383	-0.072707	Br	2.66483	2.717797	0.15263
Br	1.860633	-2.929188	0.002282	Br	1.904483	-2.889546	-0.235417	Br	1.891756	-2.925621	-0.007309
C	-4.636472	0.327612	0.14905	C	-4.667387	0.263333	0.283861	C	-4.64157	0.306428	0.14909
N	-5.375375	0.048593	-0.956745	N	-5.468484	0.054154	-0.793907	N	-5.391729	0.083405	-0.958825
C	-6.825312	0.02612	-0.941462	C	-6.91452	0.010069	-0.69485	C	-6.84203	0.055216	-0.892431
O	-5.094134	0.574796	1.261814	O	-5.060784	0.423698	1.436459	O	-5.106842	0.494217	1.270275
O	-0.26592	-1.266598	2.086289	O	-0.211686	-1.288867	1.882553	O	-0.277806	-1.229469	2.02808
O	3.324232	-0.236604	-0.335859	O	3.274109	-0.128048	-0.68303	O	3.343262	-0.218659	-0.315941
C	3.630419	-0.164938	-1.73901	C	4.267468	-0.297452	0.343138	C	3.687102	-0.089213	-1.70578
H	-0.841075	-1.790296	0.229814	H	-0.814422	-1.804853	-0.046261	H	-0.816308	-1.80491	0.09686
H	-0.238064	2.494981	0.46489	H	-0.30618	2.465716	0.400709	H	-0.257355	2.468478	0.482817
H	-2.287956	-0.057743	1.892226	H	-2.225743	-0.18298	1.866169	H	-2.2921	-0.120594	1.874173
H	-2.267706	1.631761	1.393856	H	-2.232772	1.52741	1.45354	H	-2.260467	1.578857	1.420087
H	-4.87365	-0.125249	-1.816693	H	-5.017412	-0.050021	-1.692365	H	-4.912658	-0.052284	-1.837306
H	-7.24124	0.785929	-1.61252	H	-7.377748	0.820358	-1.268998	H	-7.237368	-0.108127	-1.896129
H	-7.208471	-0.955319	-1.241353	H	-7.177067	0.125719	0.357021	H	-7.23408	0.999699	-0.502275
H	-7.147943	0.238107	0.078184	H	-7.305743	-0.94624	-1.058637	H	-7.190277	-0.748795	-0.235988
H	0.445488	-0.761692	2.506039	H	0.244888	-2.129266	2.033635	H	0.196987	-2.047283	2.235607
H	3.34214	0.806361	-2.154524	H	5.224666	-0.350647	-0.176645	H	3.196669	-0.872254	-2.293569
H	4.711274	-0.287511	-1.81513	H	4.096102	-1.226647	0.895271	H	3.412566	0.899116	-2.088592
H	3.12672	-0.973003	-2.279178	H	4.268169	0.558263	1.025644	H	4.76939	-0.212038	-1.758018

5c2			
C	1.182387	-1.137551	0.004337
C	-0.206443	-1.011533	0.574053
C	-0.861929	0.35743	0.222146
C	0.111779	1.49957	0.235667
C	1.409384	1.279002	-0.014865
C	1.970816	-0.071414	-0.237546
O	-1.338491	0.224755	-1.185467
N	-2.719015	0.124616	-1.199306
C	-3.191984	0.313283	-0.022482
C	-2.153644	0.594732	1.024987
Br	2.636112	2.735836	-0.094756
Br	1.836826	-2.904524	-0.215471
C	-4.661425	0.298257	0.260046
N	-5.450585	0.022653	-0.811207
C	-6.897588	-0.019112	-0.724559
O	-5.06657	0.527765	1.396852
O	-0.193758	-1.26631	1.97592

O	3.235718	-0.170625	-0.728713
C	4.267844	-0.382138	0.249211
H	-0.849014	-1.784754	0.145374
H	-0.268562	2.504286	0.380705
H	-2.233235	-0.071003	1.886709
H	-2.247429	1.621076	1.396348
H	-4.990079	-0.135386	-1.696934
H	-7.356175	0.753544	-1.35182
H	-7.282829	-0.996856	-1.033412
H	-7.171385	0.159698	0.315525
H	0.503032	-0.722095	2.370187
H	4.094459	-1.311751	0.799774
H	5.198735	-0.458509	-0.313583
H	4.32648	0.467126	0.937957

2. 1D and 2D NMR, MS, and chiral HPLC spectra of the new compounds.

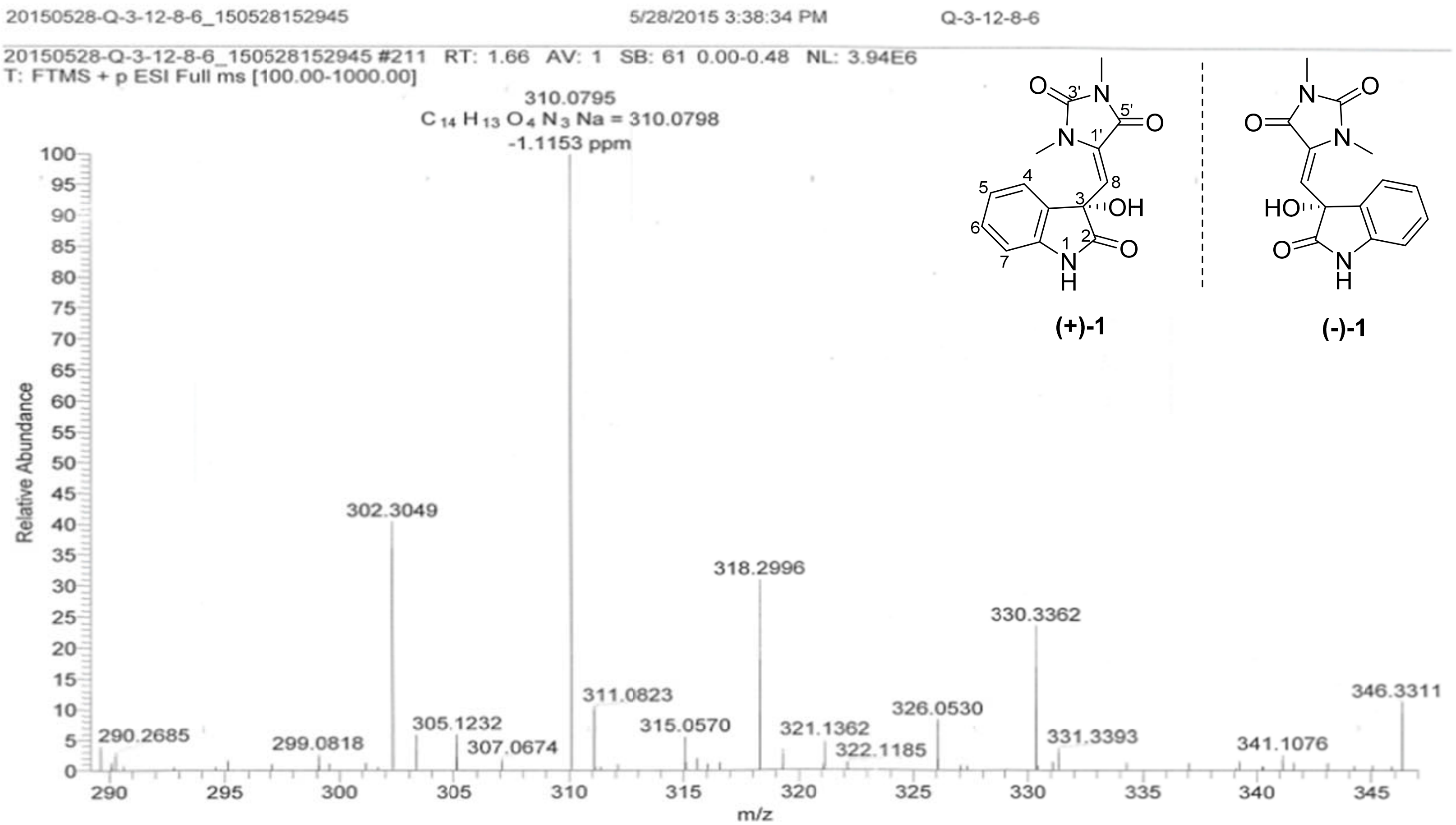

Figure S1. (+)-HRESIMS spectrum of **1**.

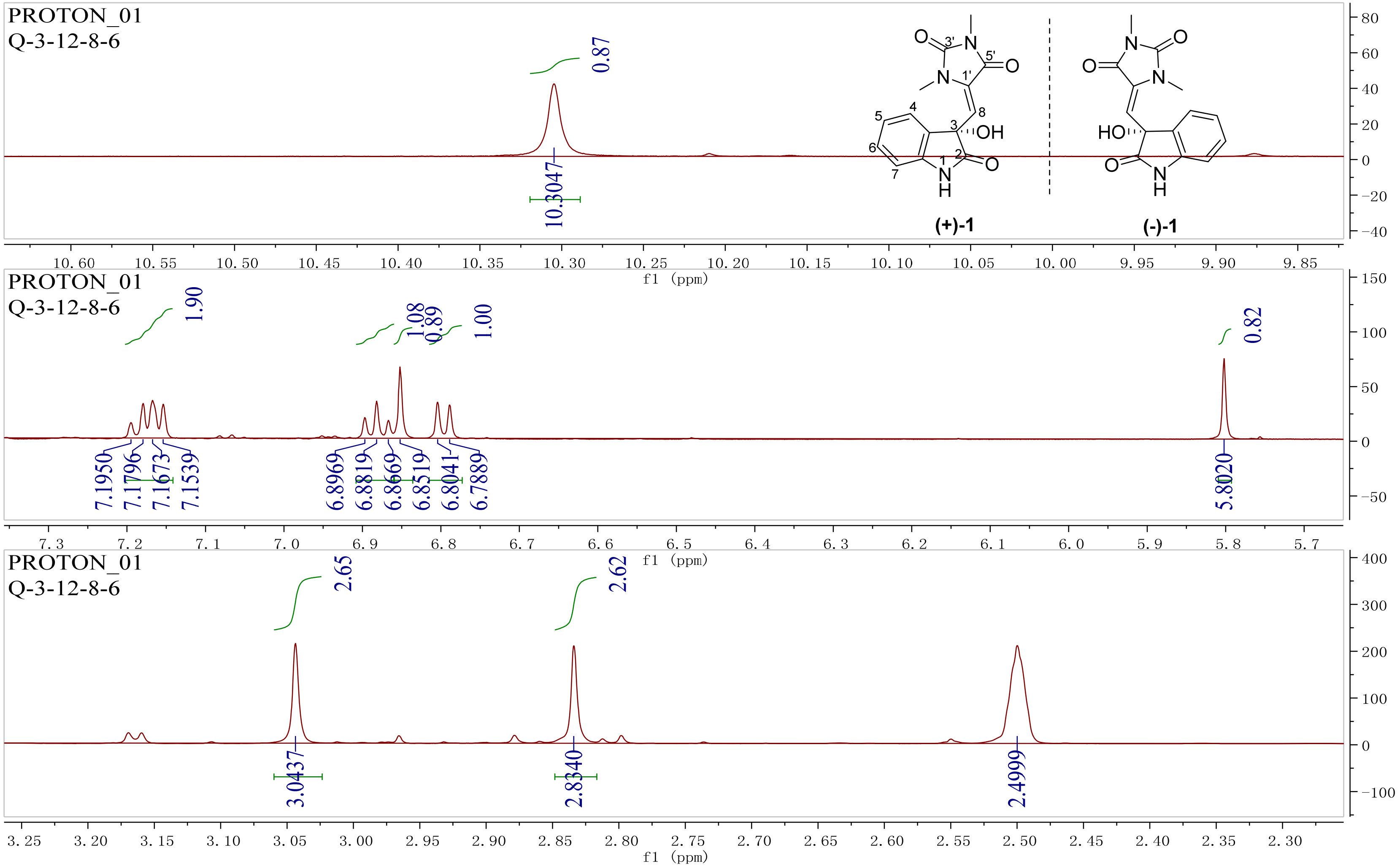

Figure S2. ^{1}H NMR (500 MHz, DMSO-d_6) spectrum of **1**.

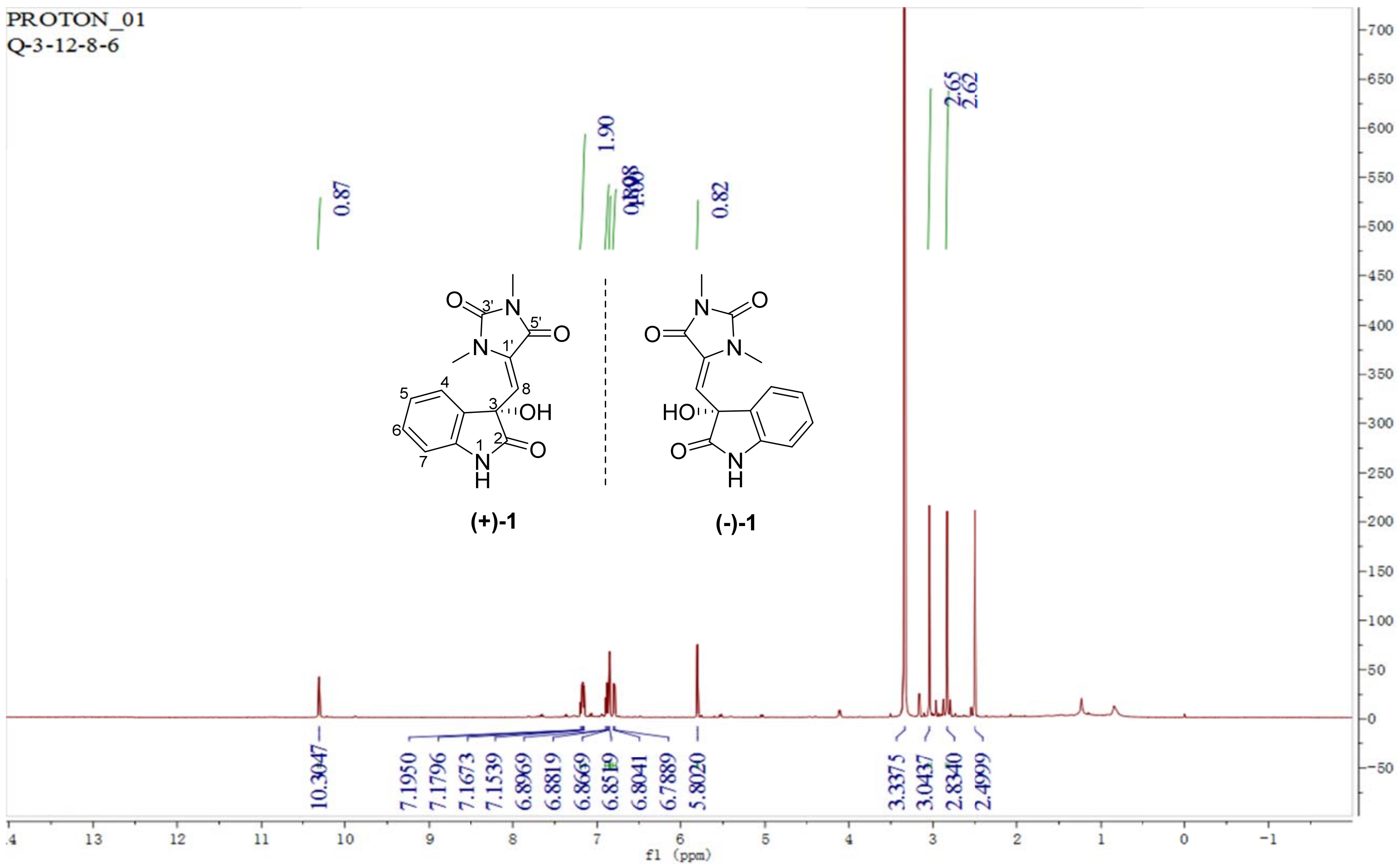

Figure S3. ^{1}H NMR (500 MHz, DMSO-d_6) spectrum of **1**.

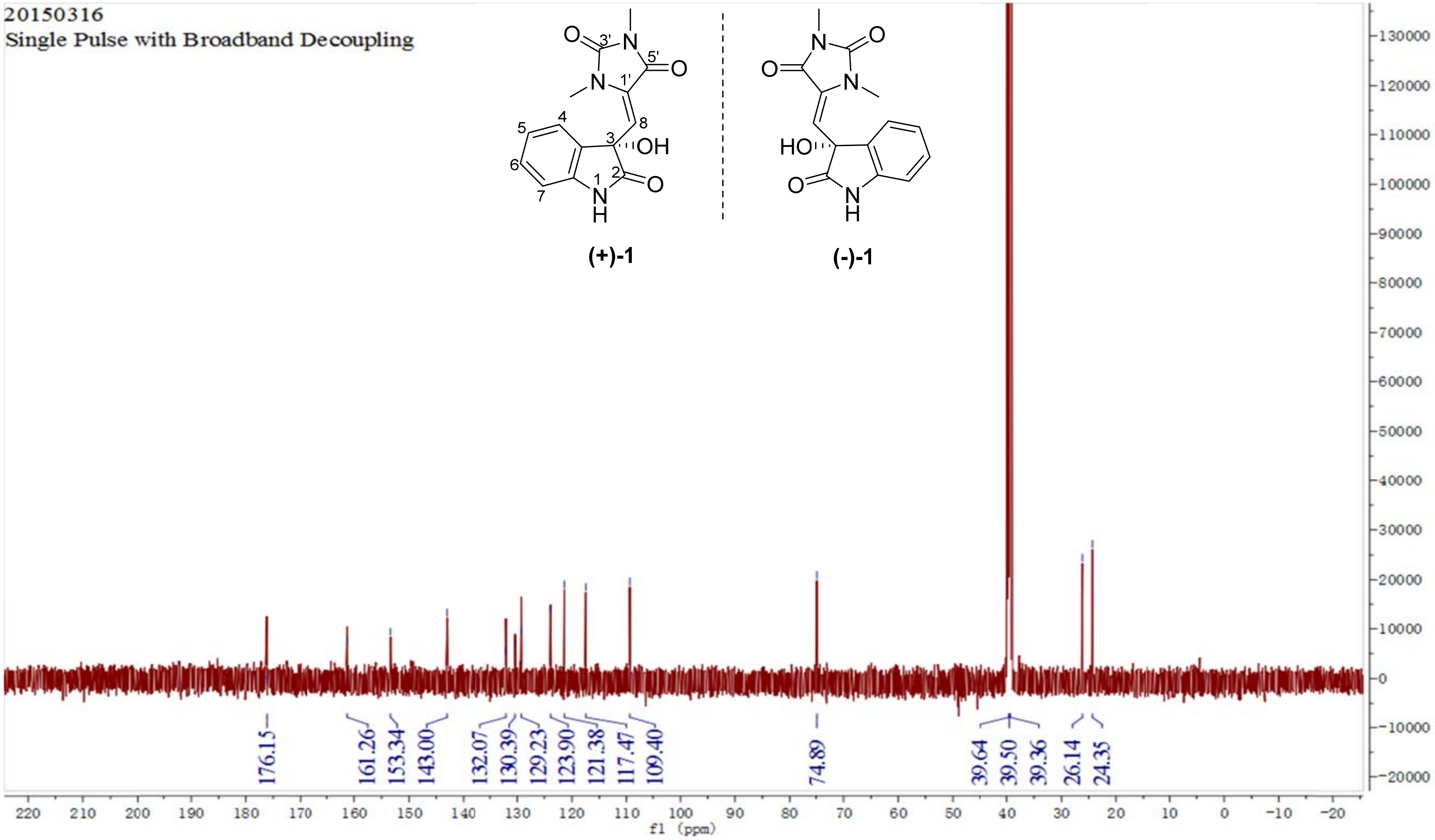

Figure S4. ^{13}C NMR (125 MHz, DMSO-d_6) spectrum of **1**.

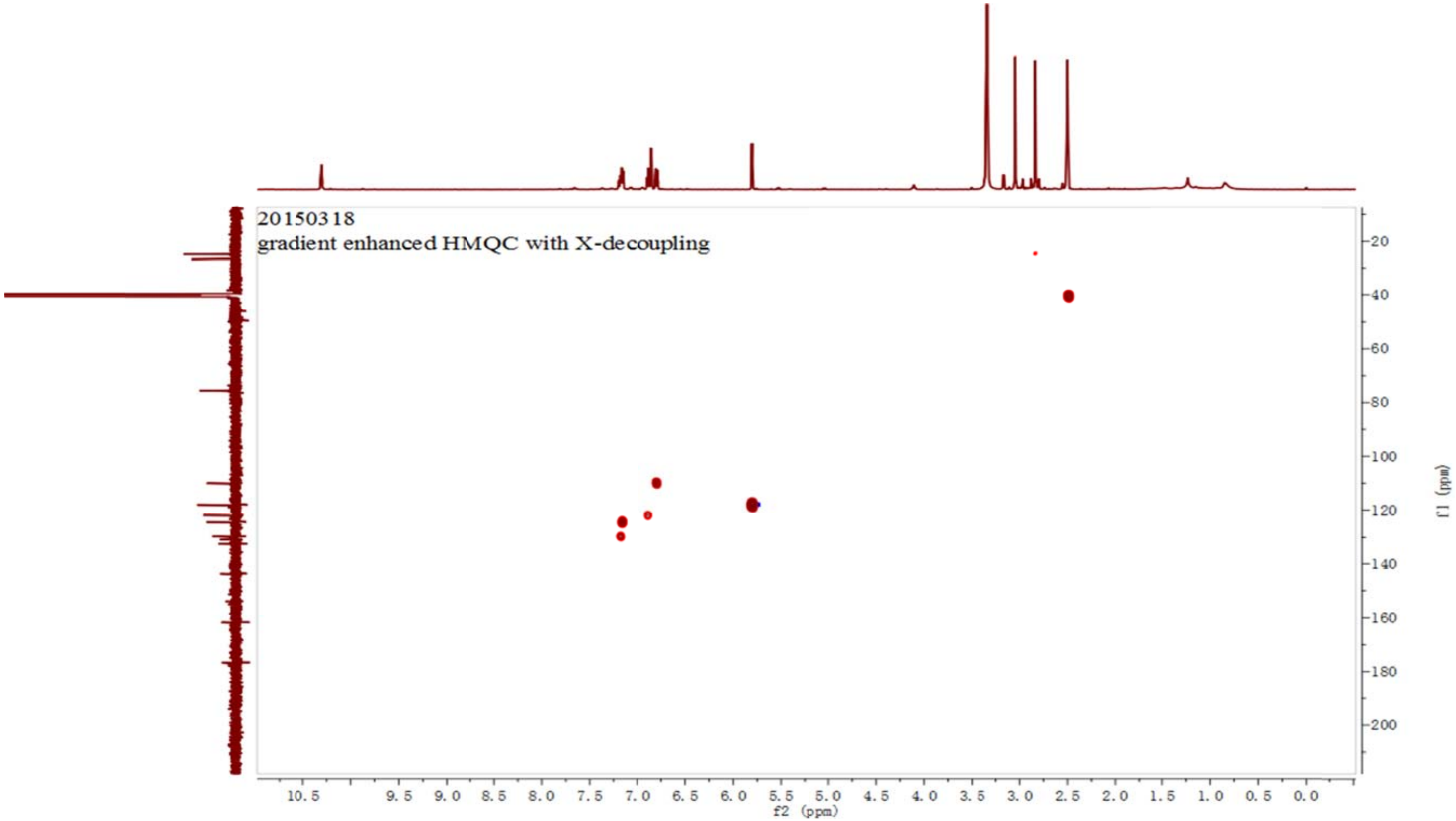

Figure S5. HMQC spectrum of Compound **1**.

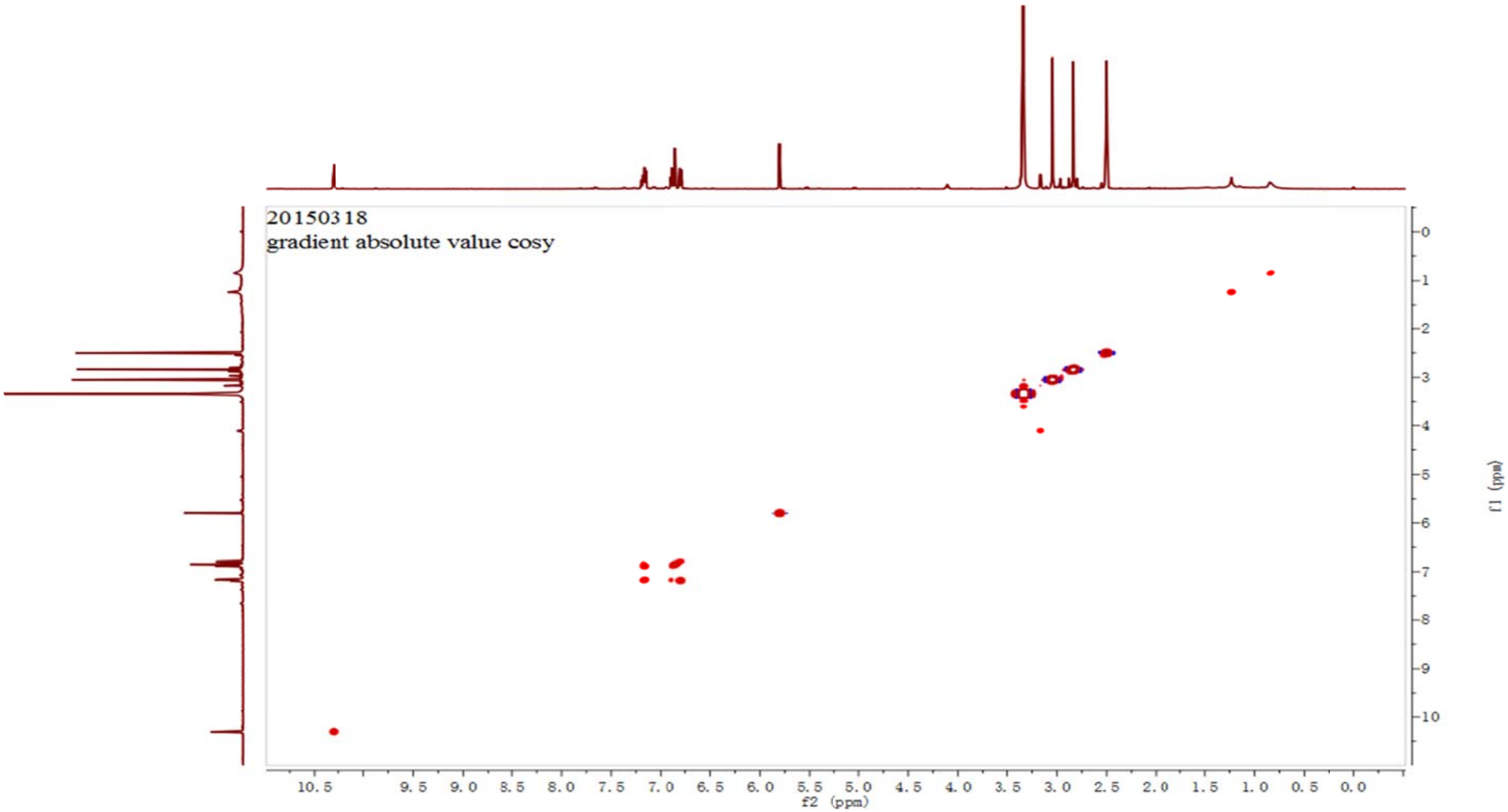

Figure S6. ^{1}H-^{1}H COSY spectrum of Compound **1**.

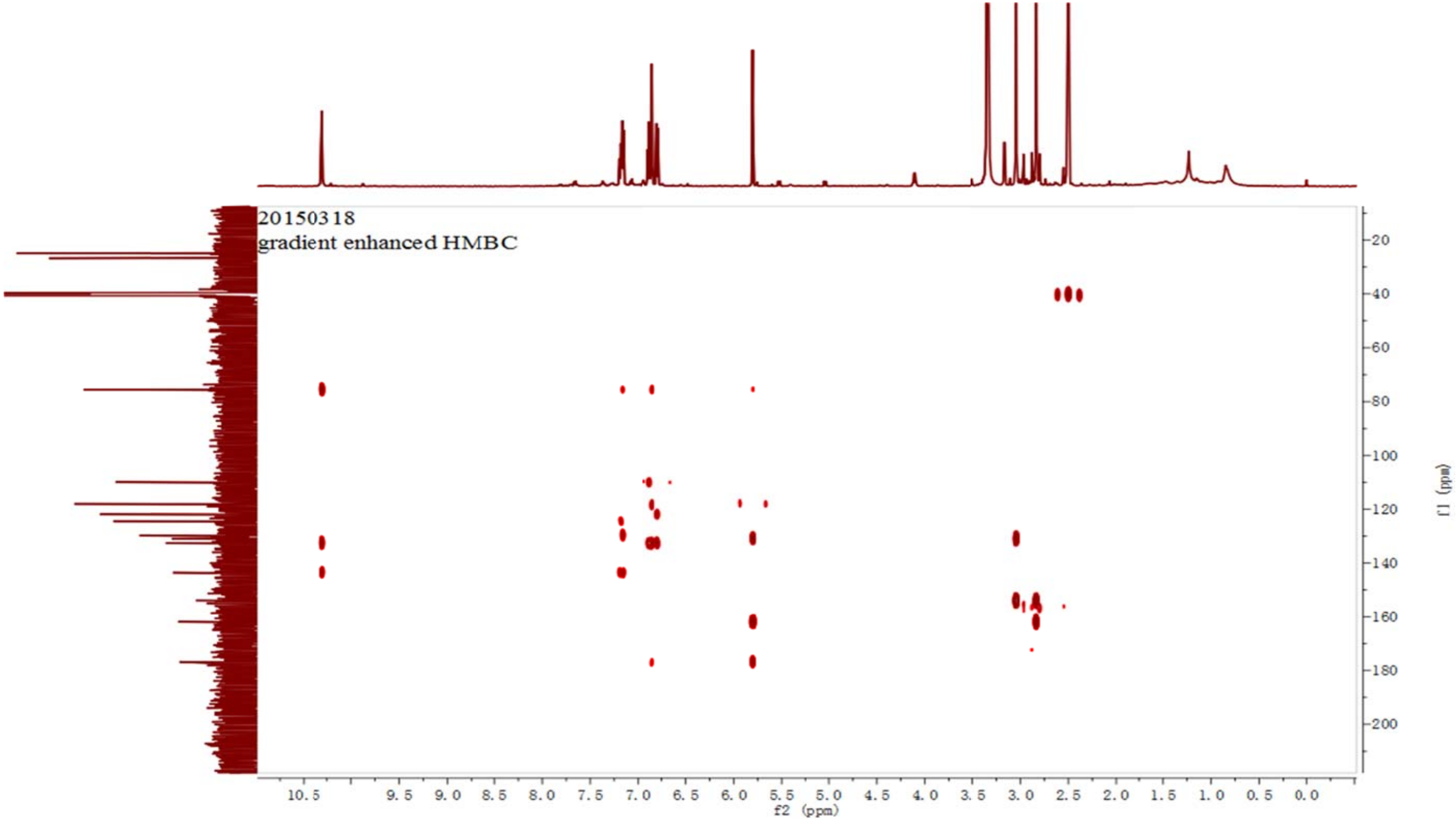

Figure S7. HMBC spectrum of Compound **1**.

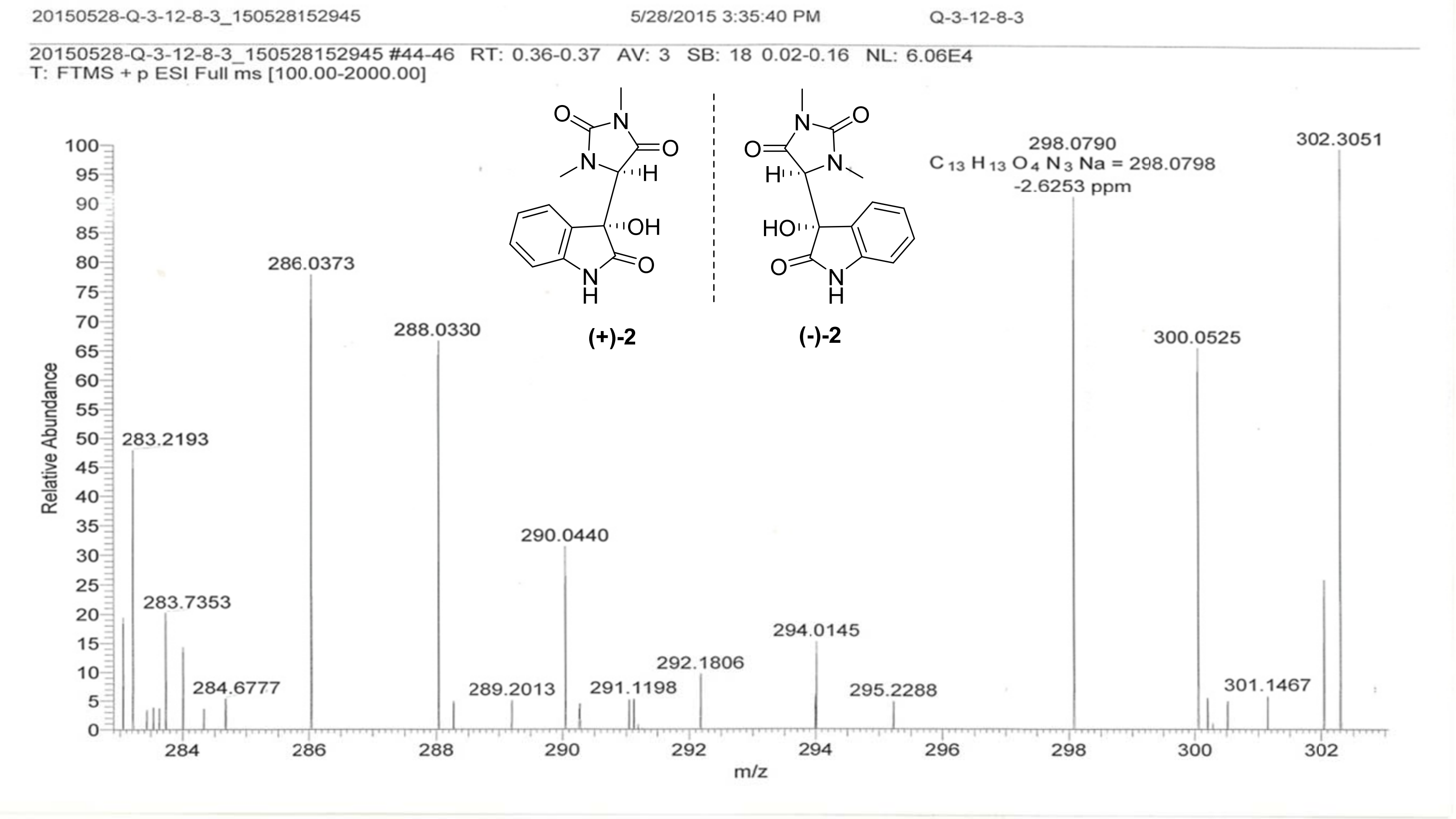

Figure S8. (+)-HRESIMS spectrum of **2**.

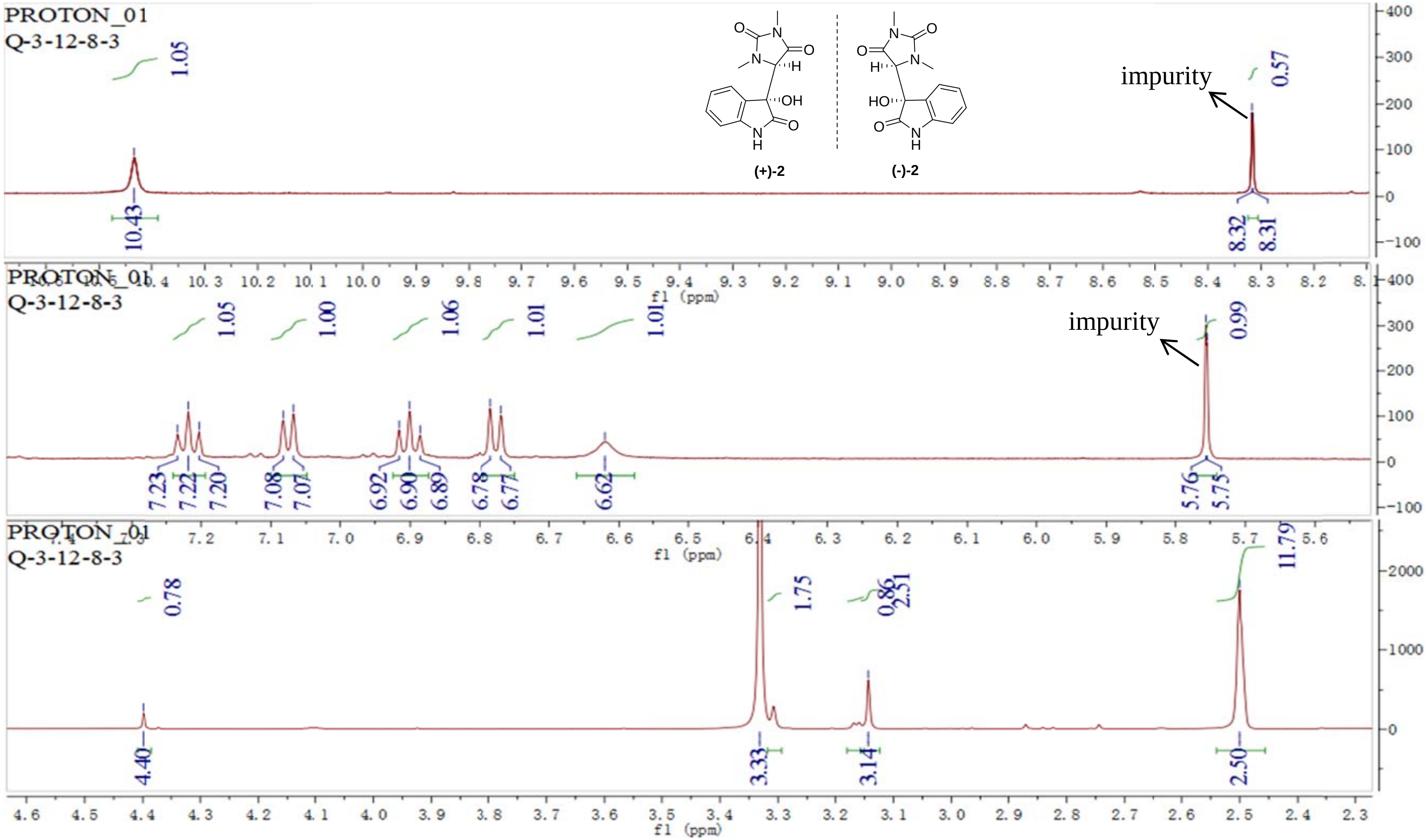

Figure S9. ^{1}H NMR (500 MHz, DMSO-d_6) spectrum of **2**.

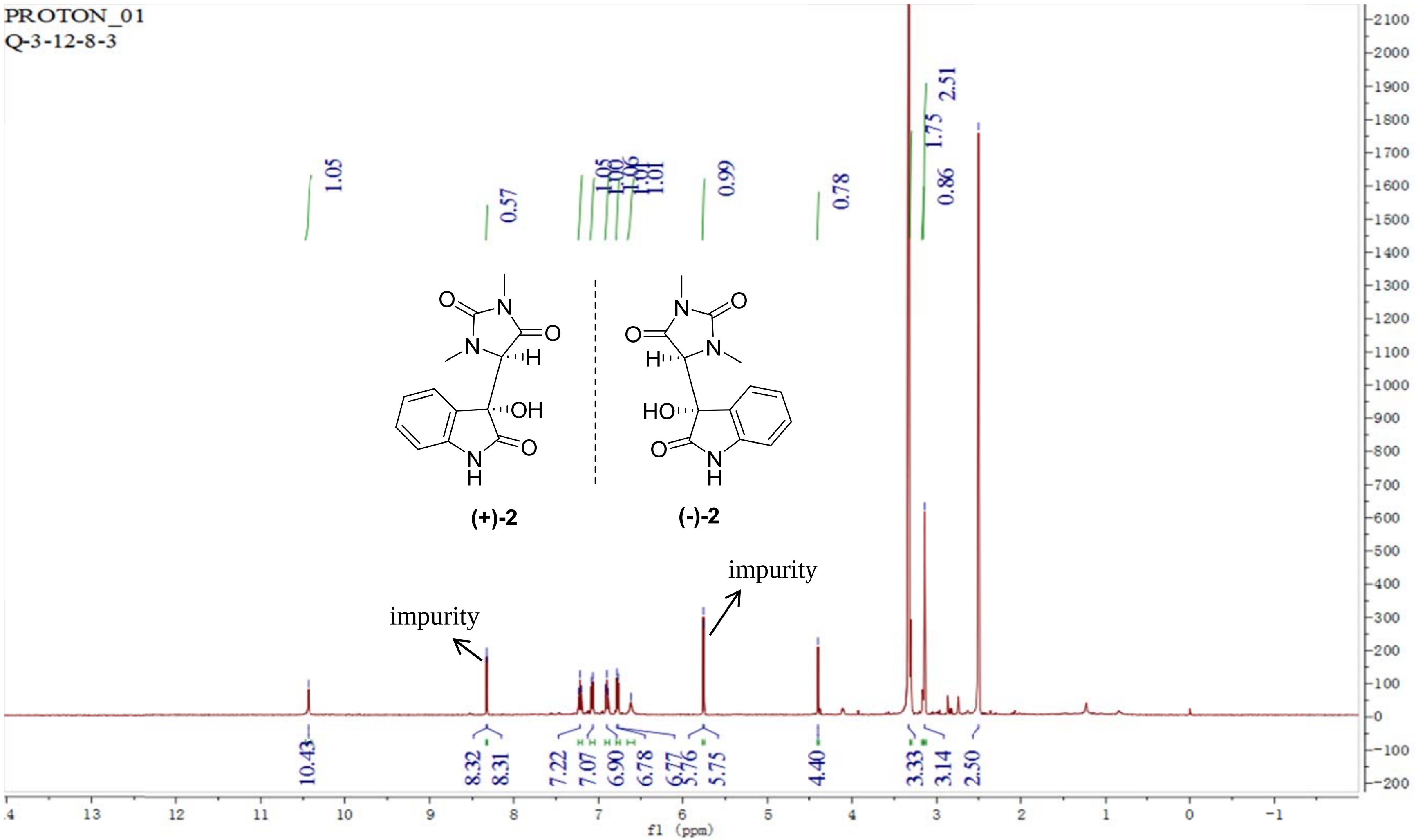

Figure S10. ^{1}H NMR (500 MHz, DMSO-d_6) spectrum of **2**.

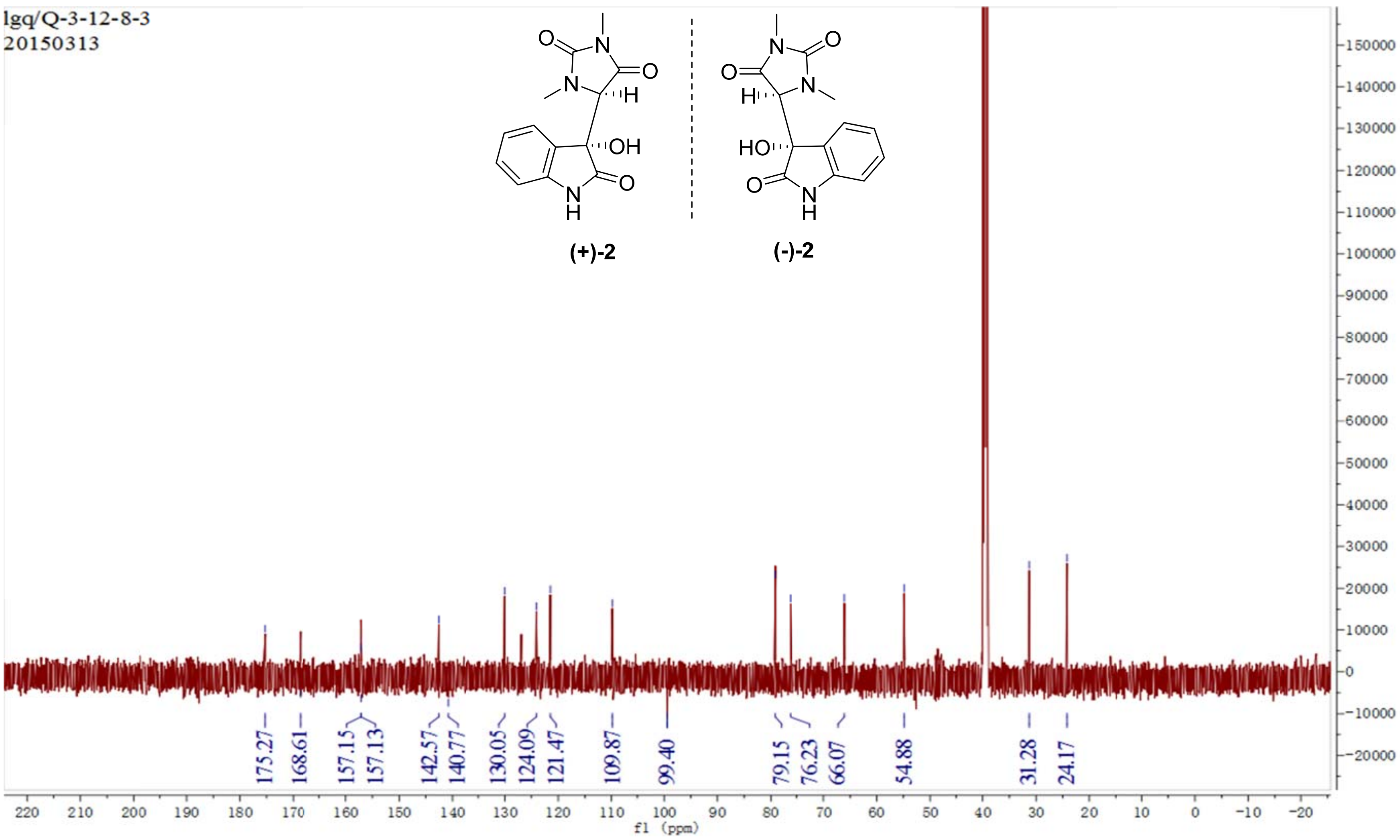

Figure S11. ^{13}C NMR (125 MHz, DMSO-d_6) spectrum of **2**.

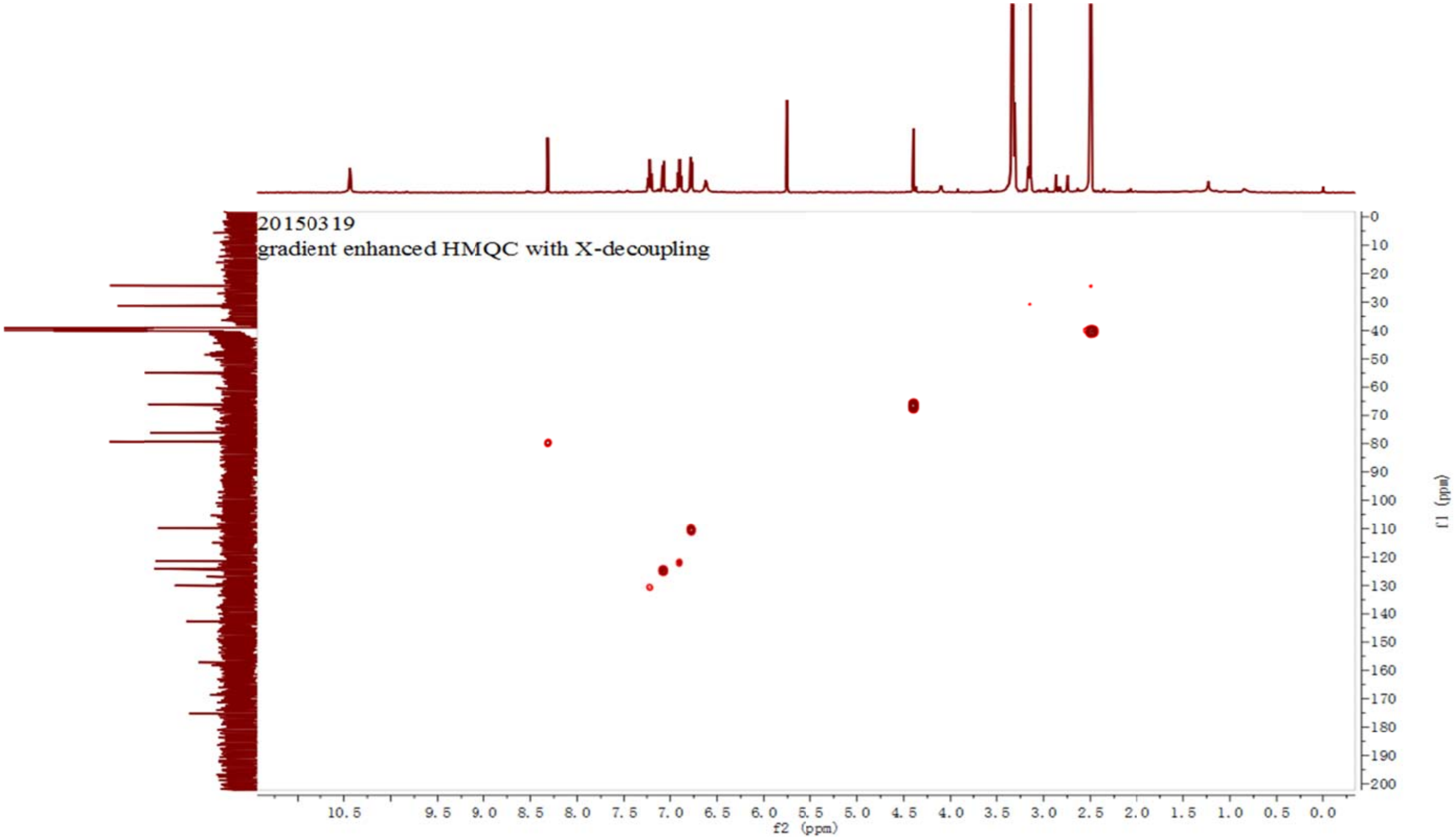

Figure S12. HMQC spectrum of Compound **2**.

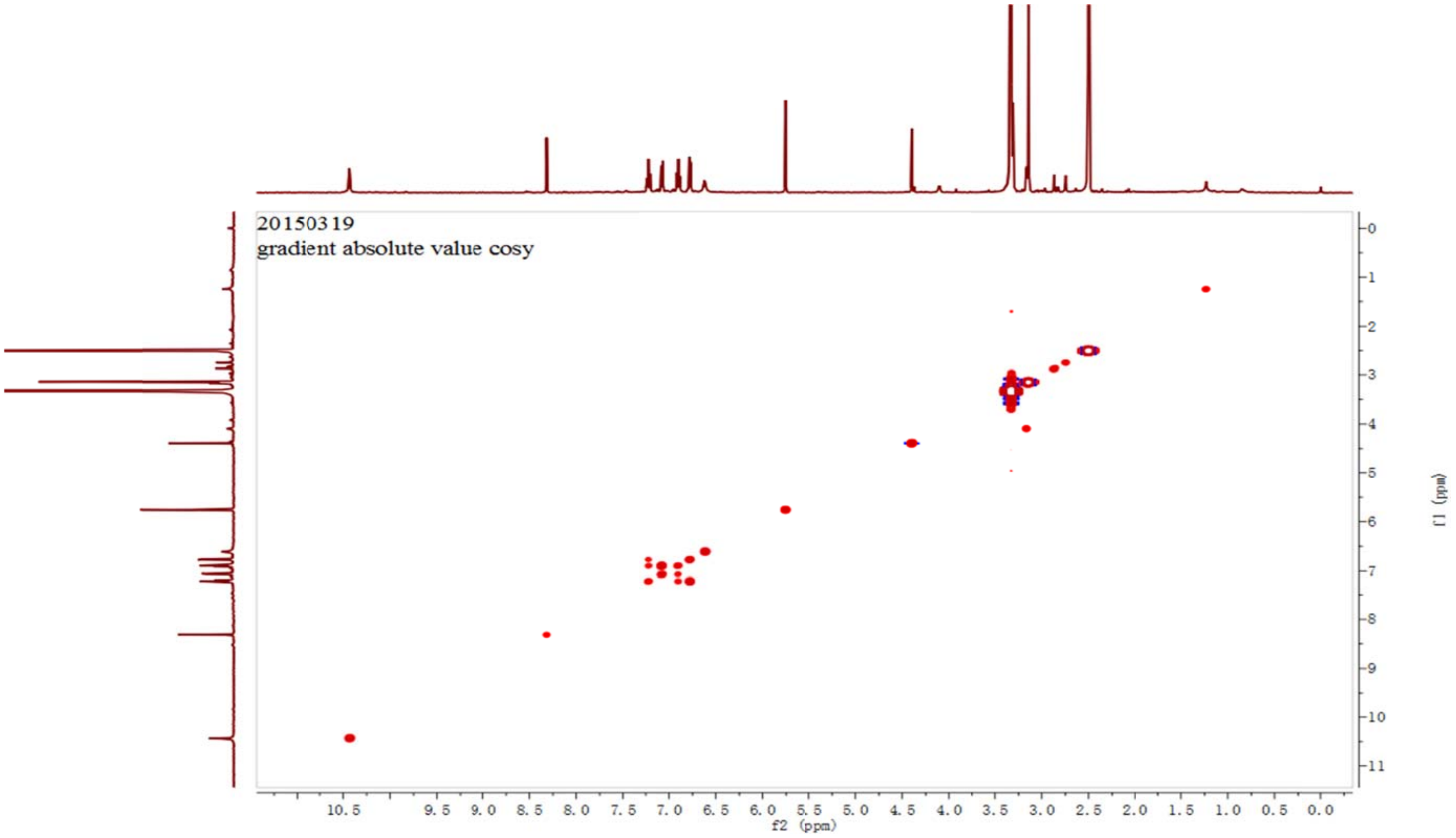

Figure S13. ¹H-¹H COSY spectrum of Compound **2**.

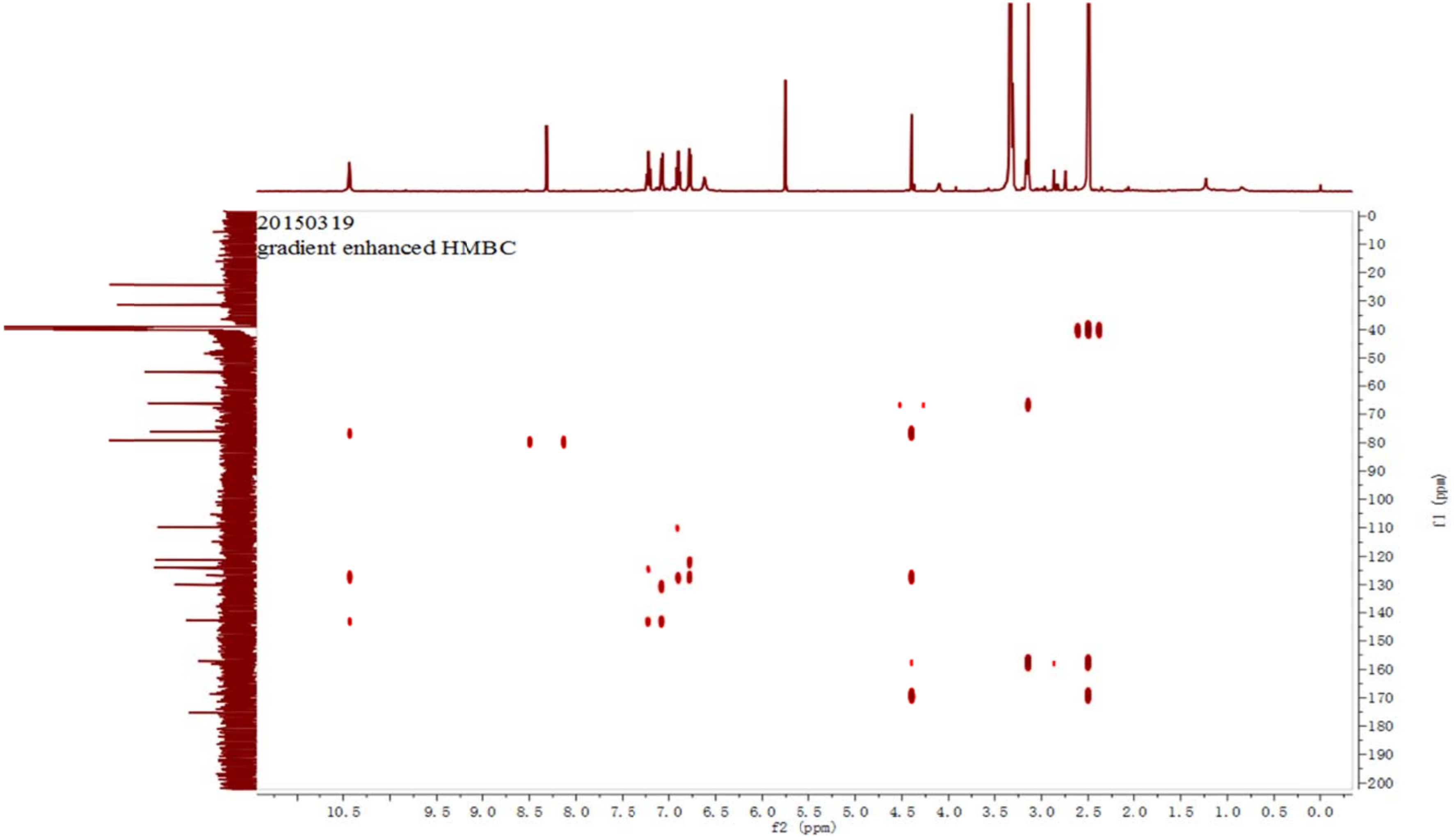

Figure S14. HMBC spectrum of Compound **2**.

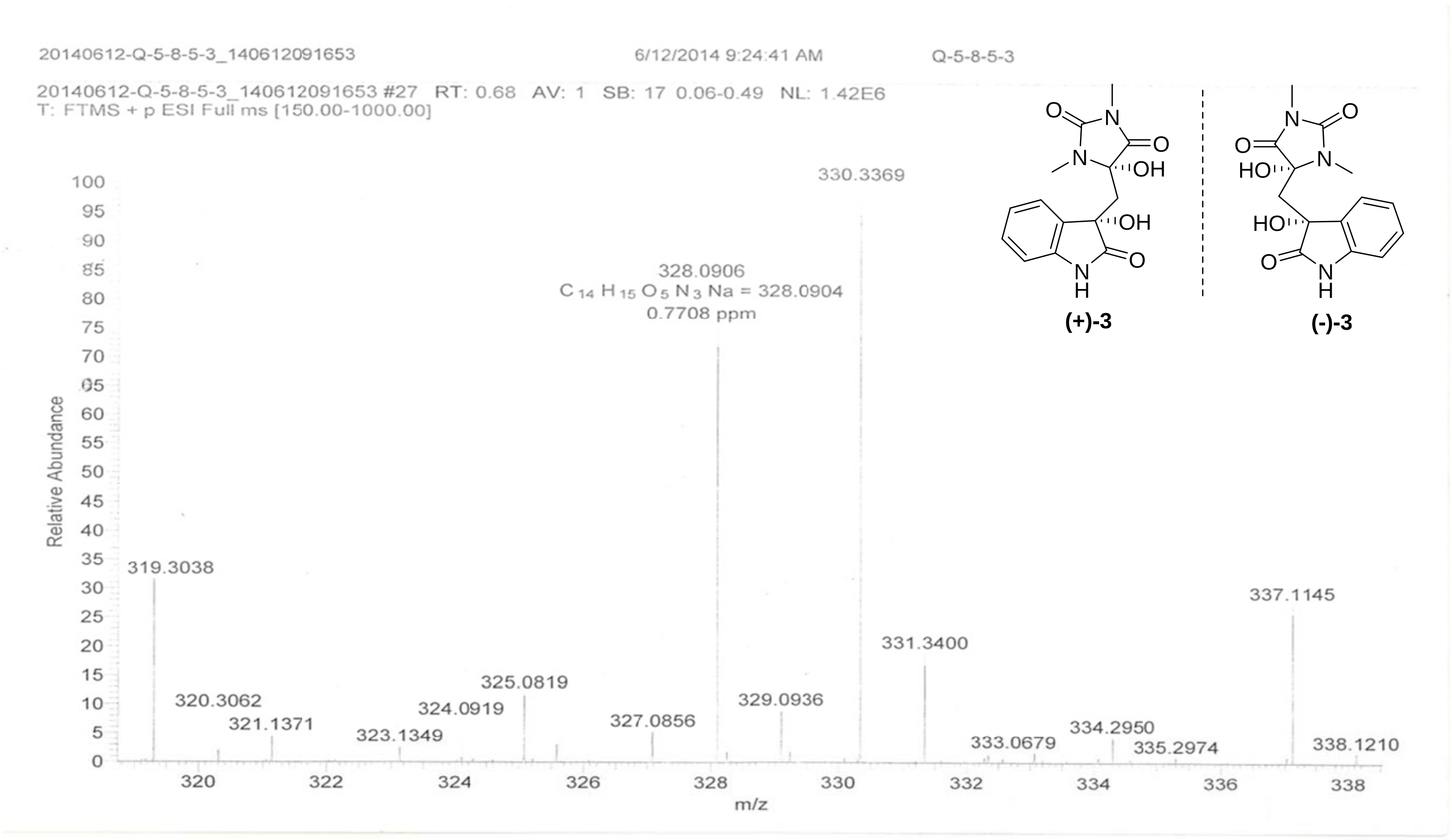

Figure S15. (+)-HRESIMS spectrum of **3**.

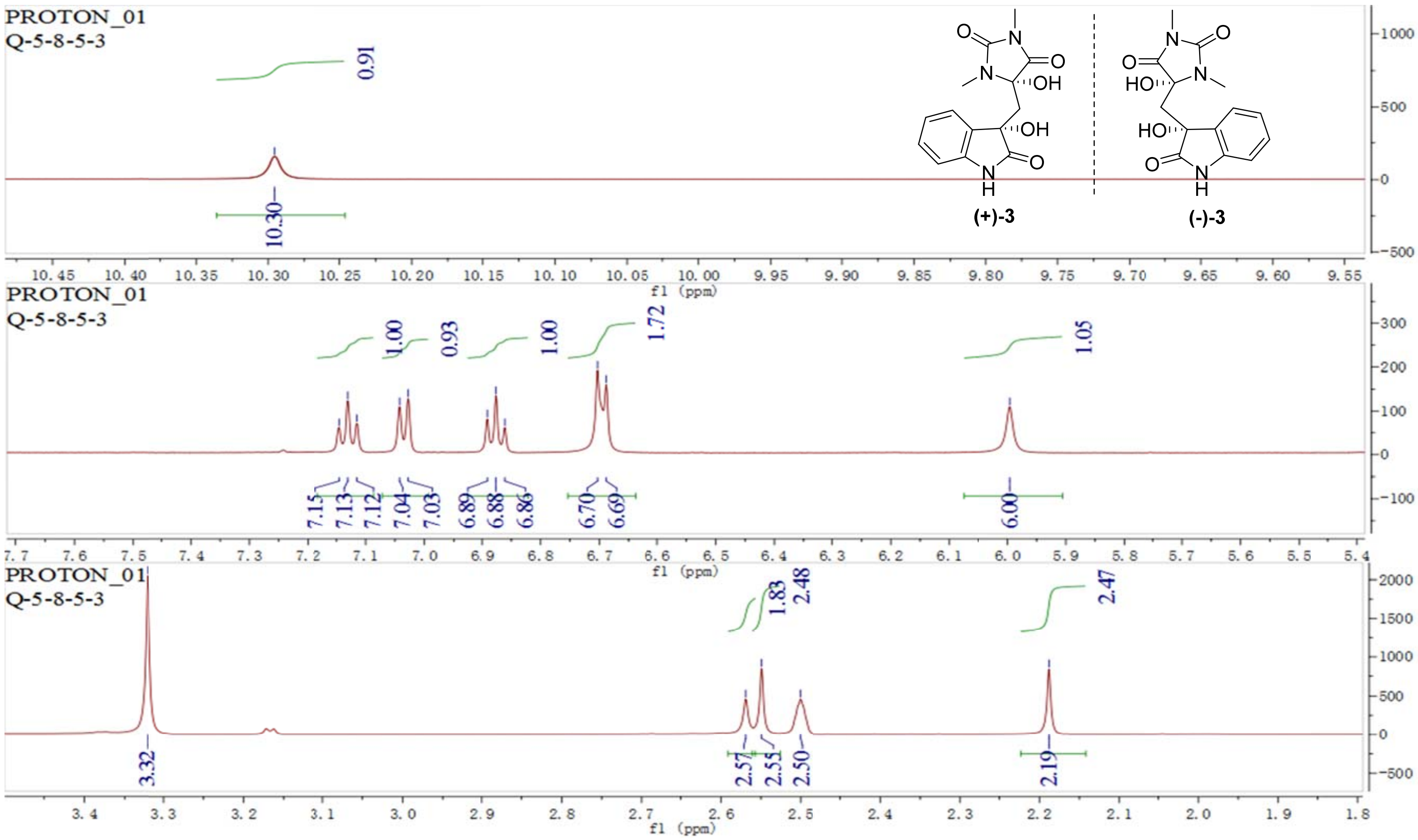

Figure S16. ^{1}H NMR (500 MHz, DMSO-d_6) spectrum of **3**.

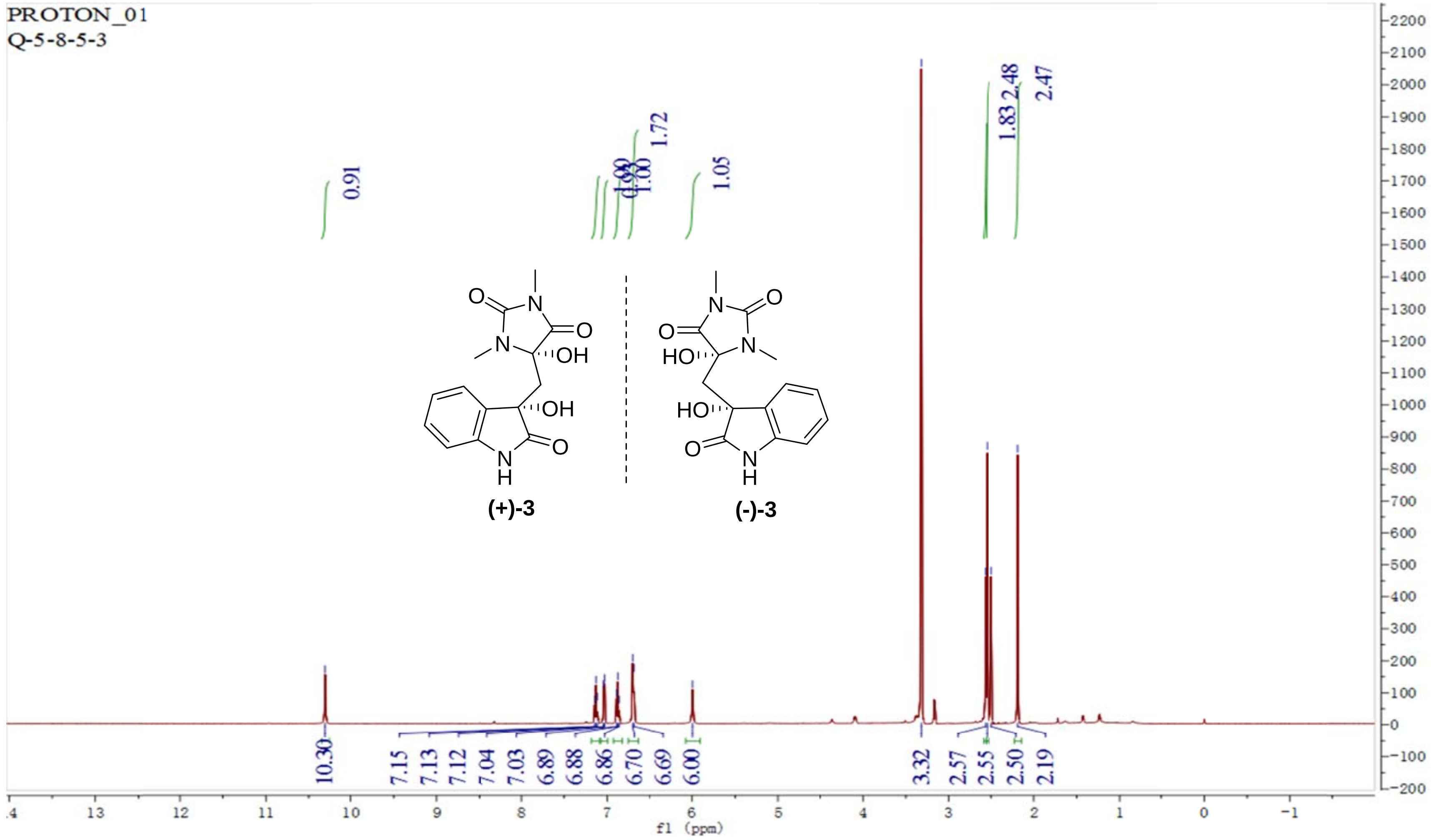

Figure S17. ^{1}H NMR (500 MHz, DMSO-d_6) spectrum of **3**.

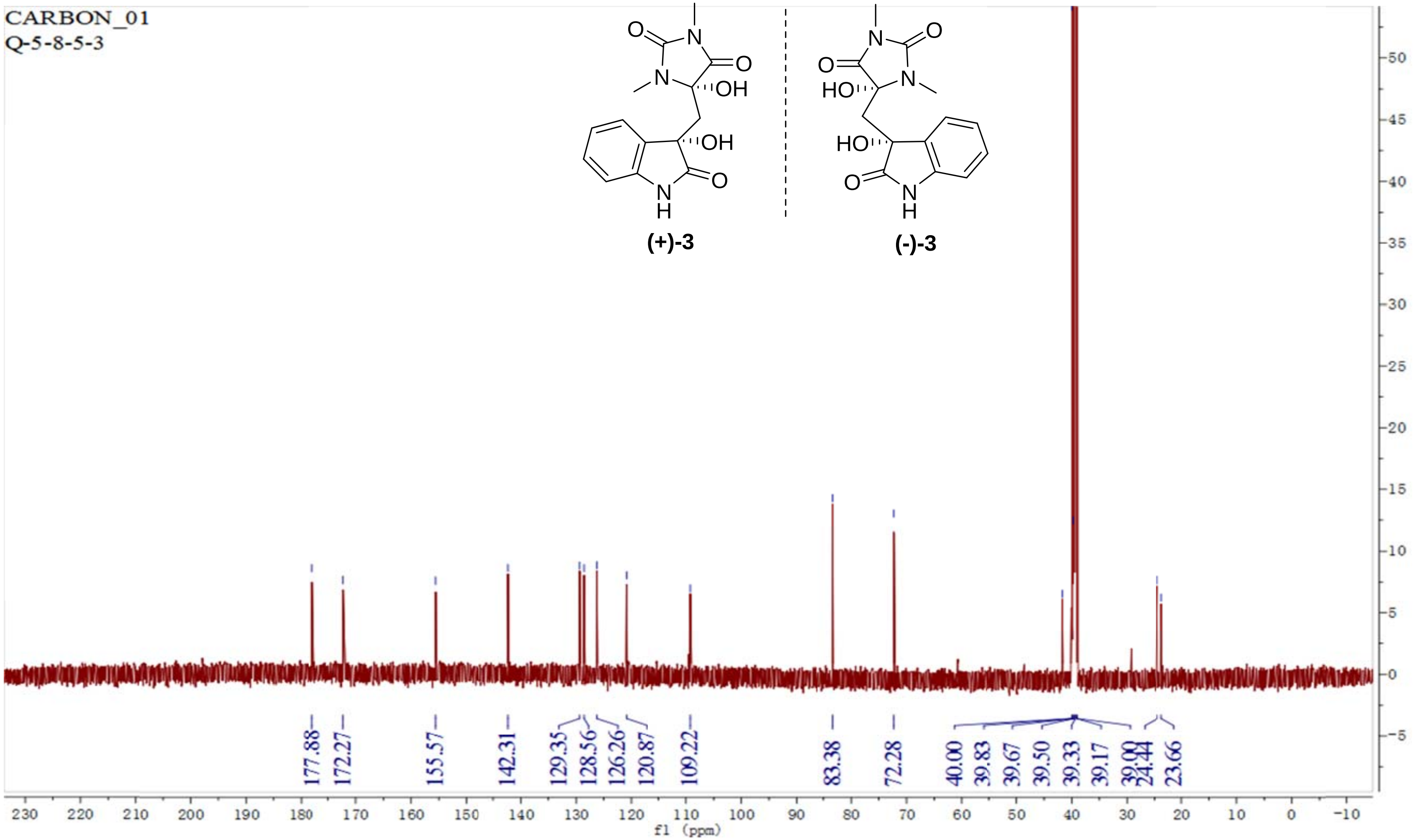

Figure S18. ^{13}C NMR (125 MHz, DMSO-d_6) spectrum of **3**.

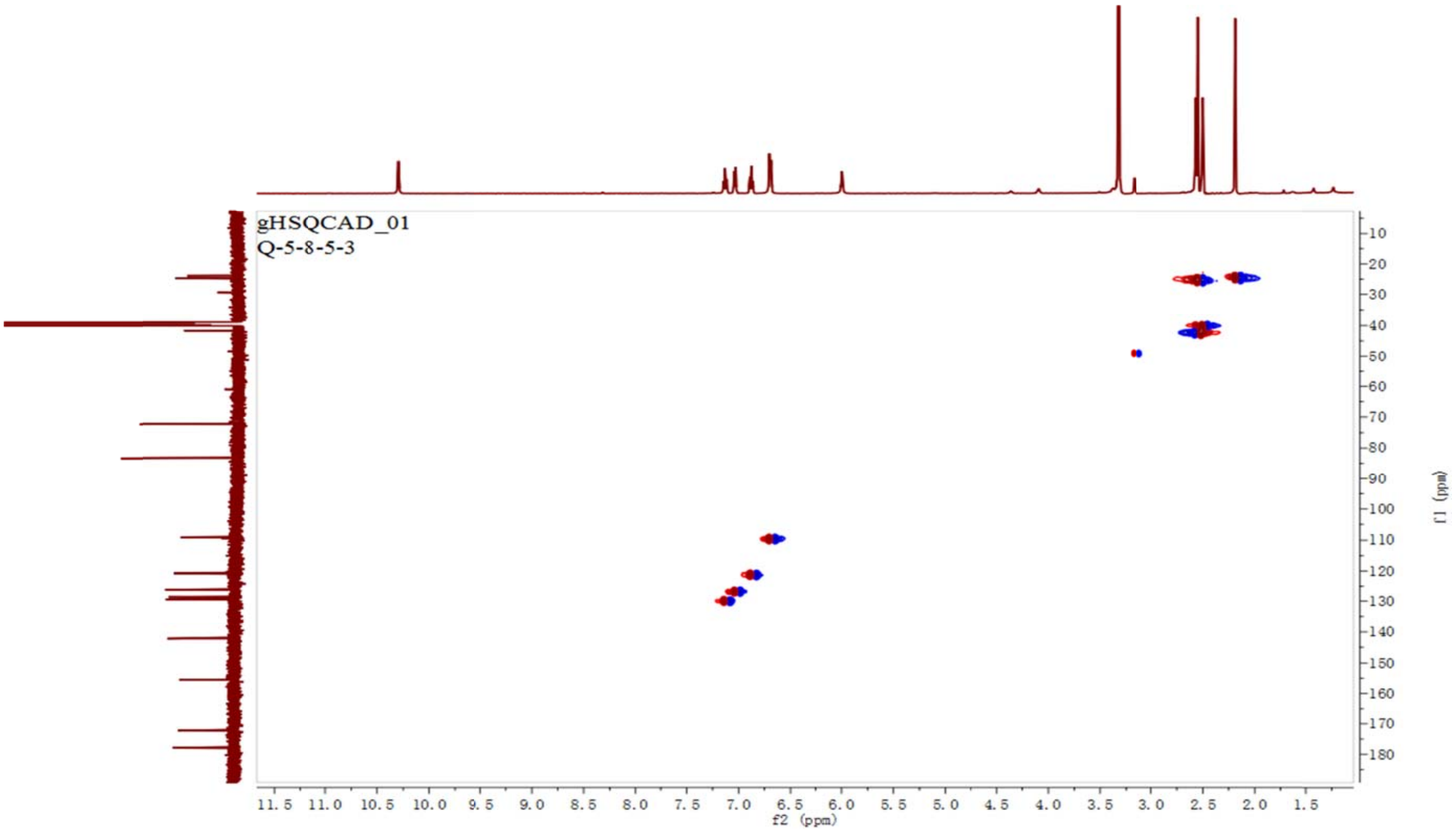

Figure S19. HSQC spectrum of Compound **3**.

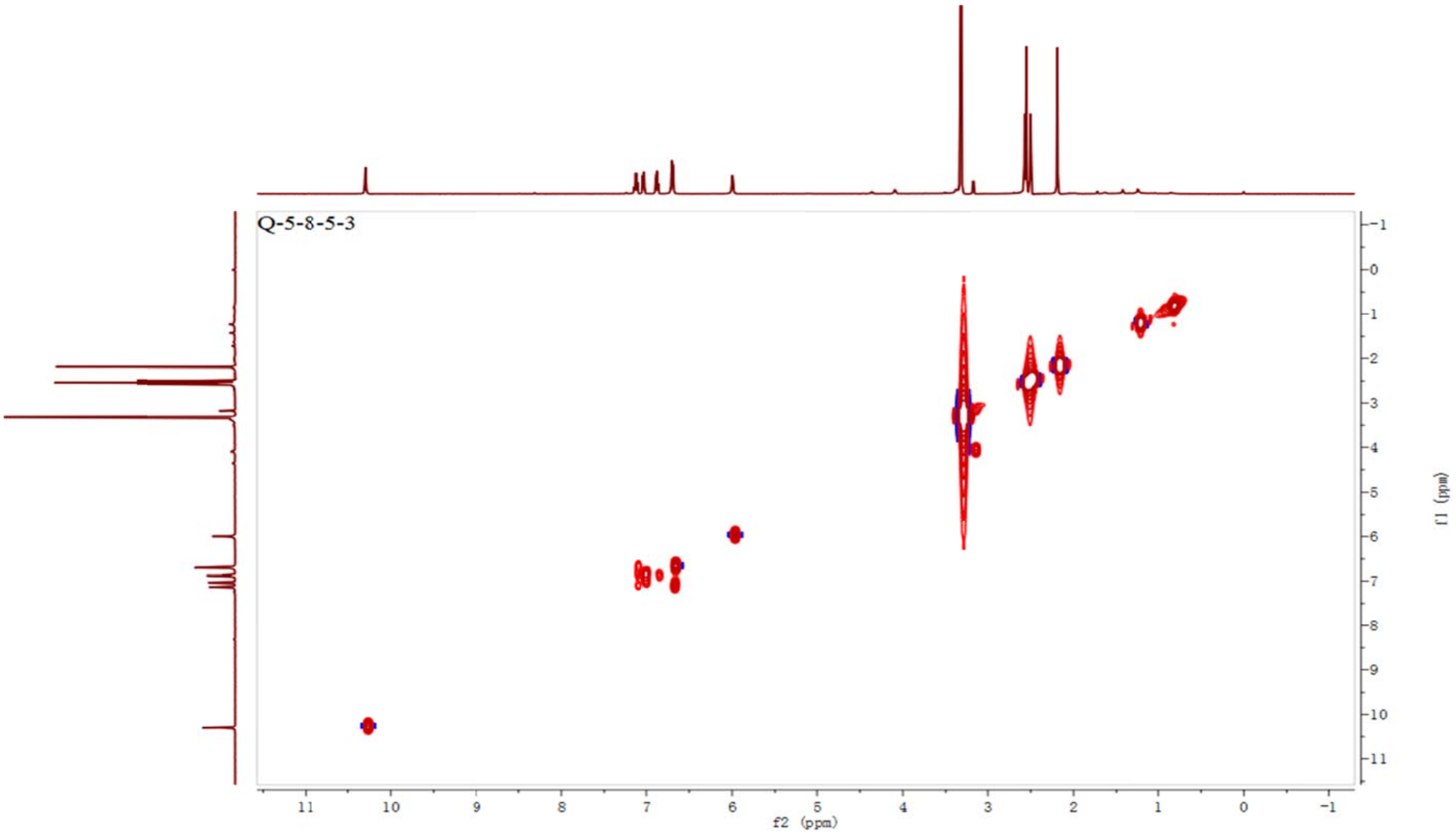

Figure S20. ^{1}H-^{1}H COSY spectrum of Compound **3**.

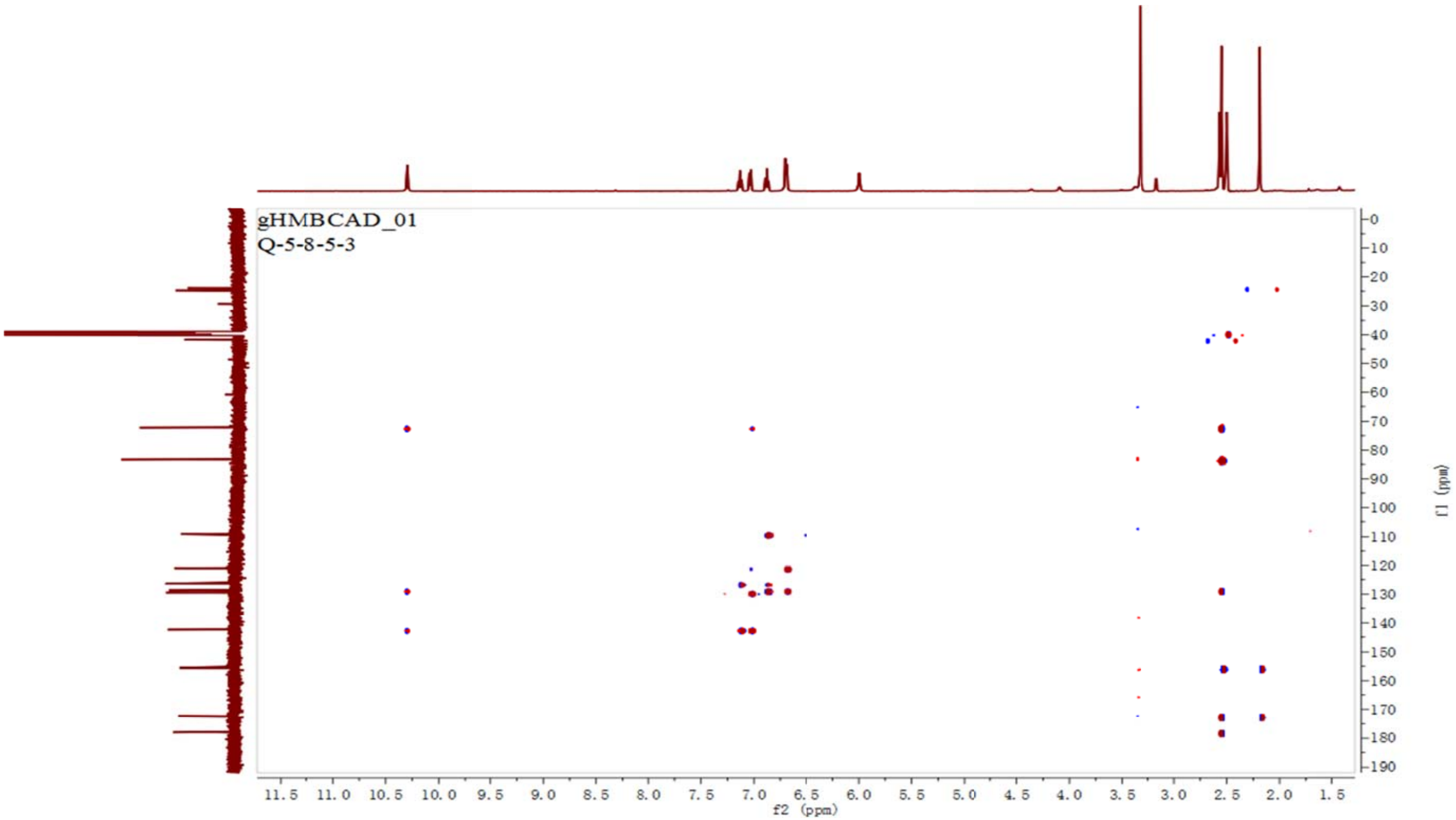

Figure S21. HMBC spectrum of Compound **3**.

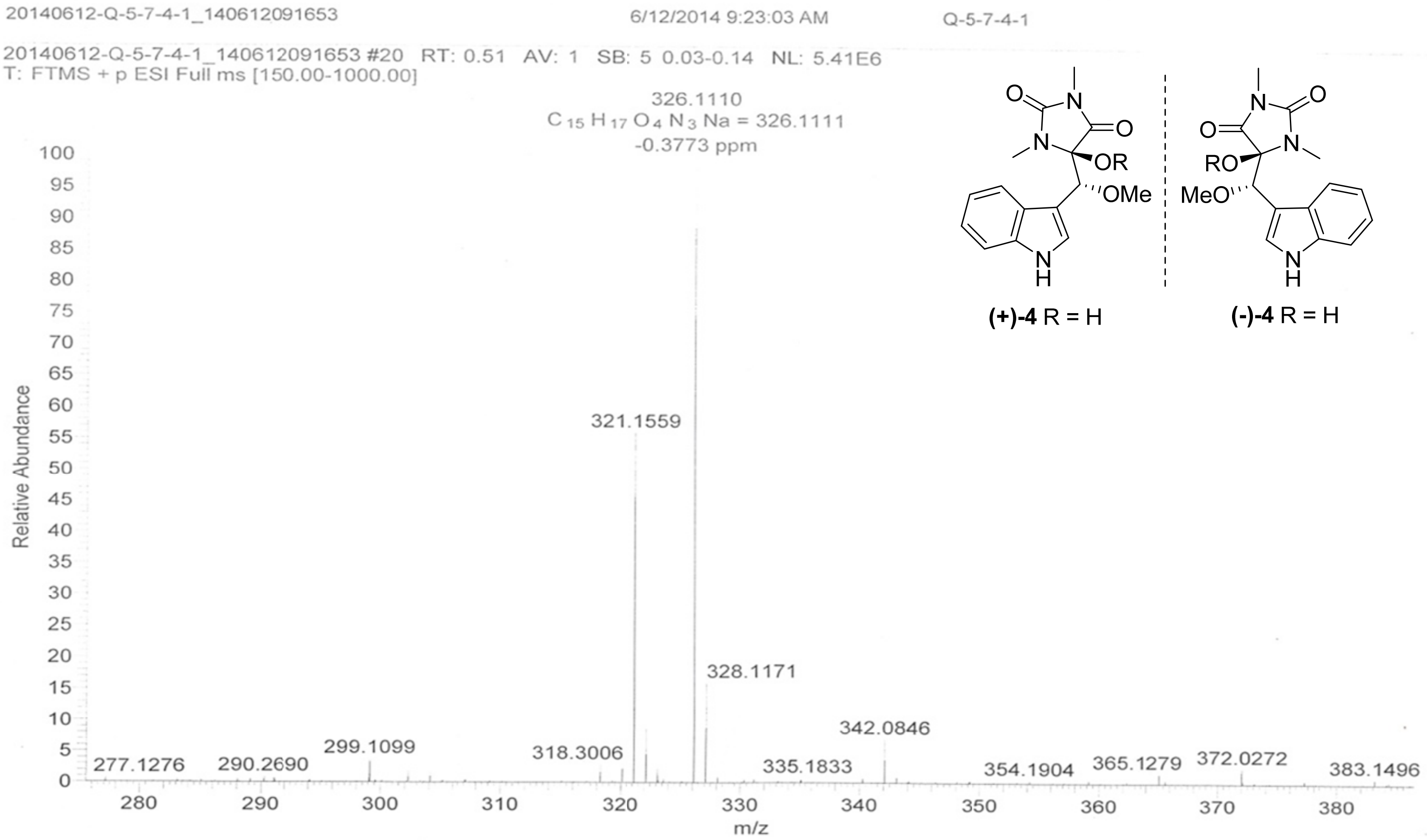

(+)-4 R = H **(-)-4** R = H

Figure S22. (+)-HRESIMS spectrum of **4**.

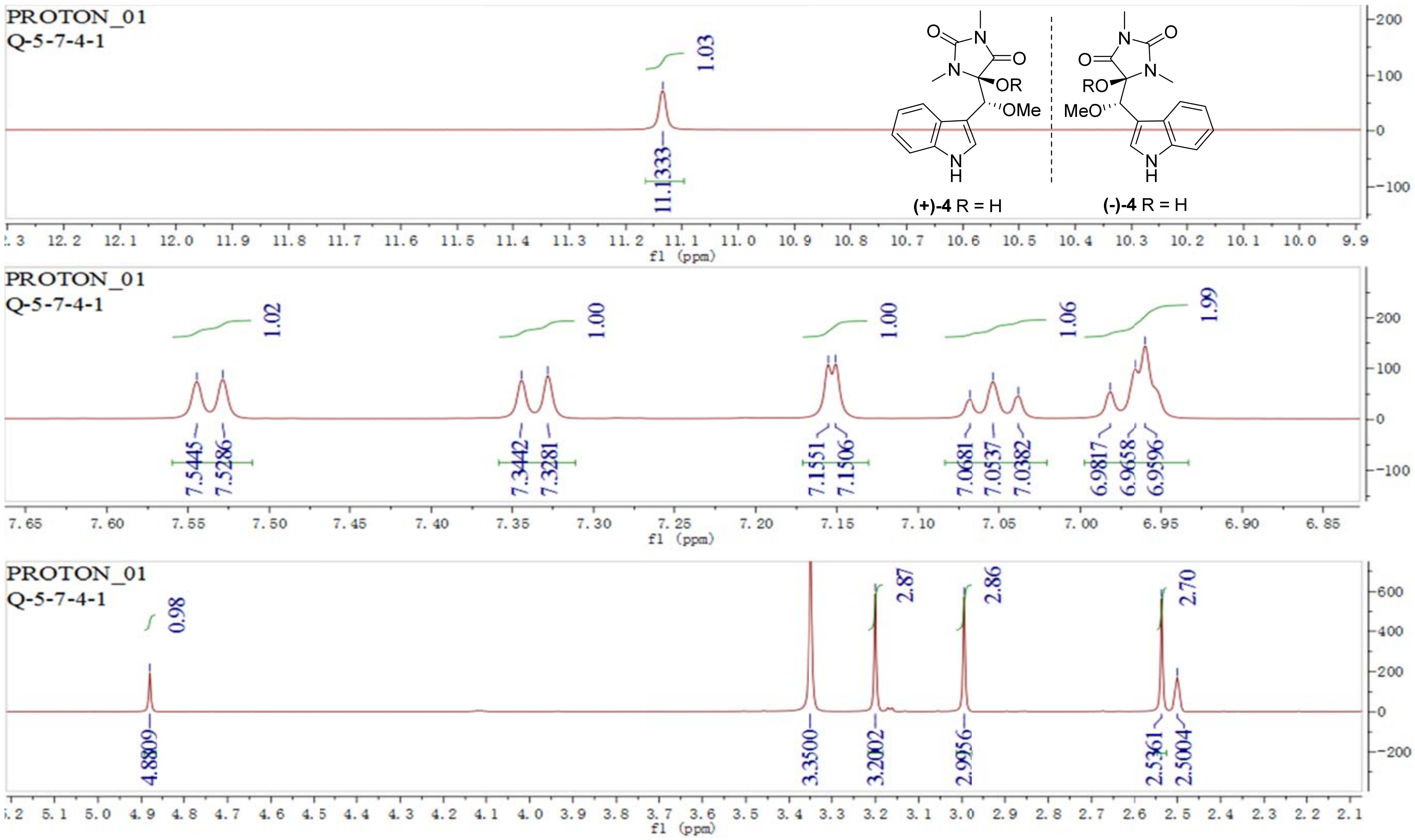

Figure S23. ^{1}H NMR (500 MHz, DMSO-d_6) spectrum of **4**.

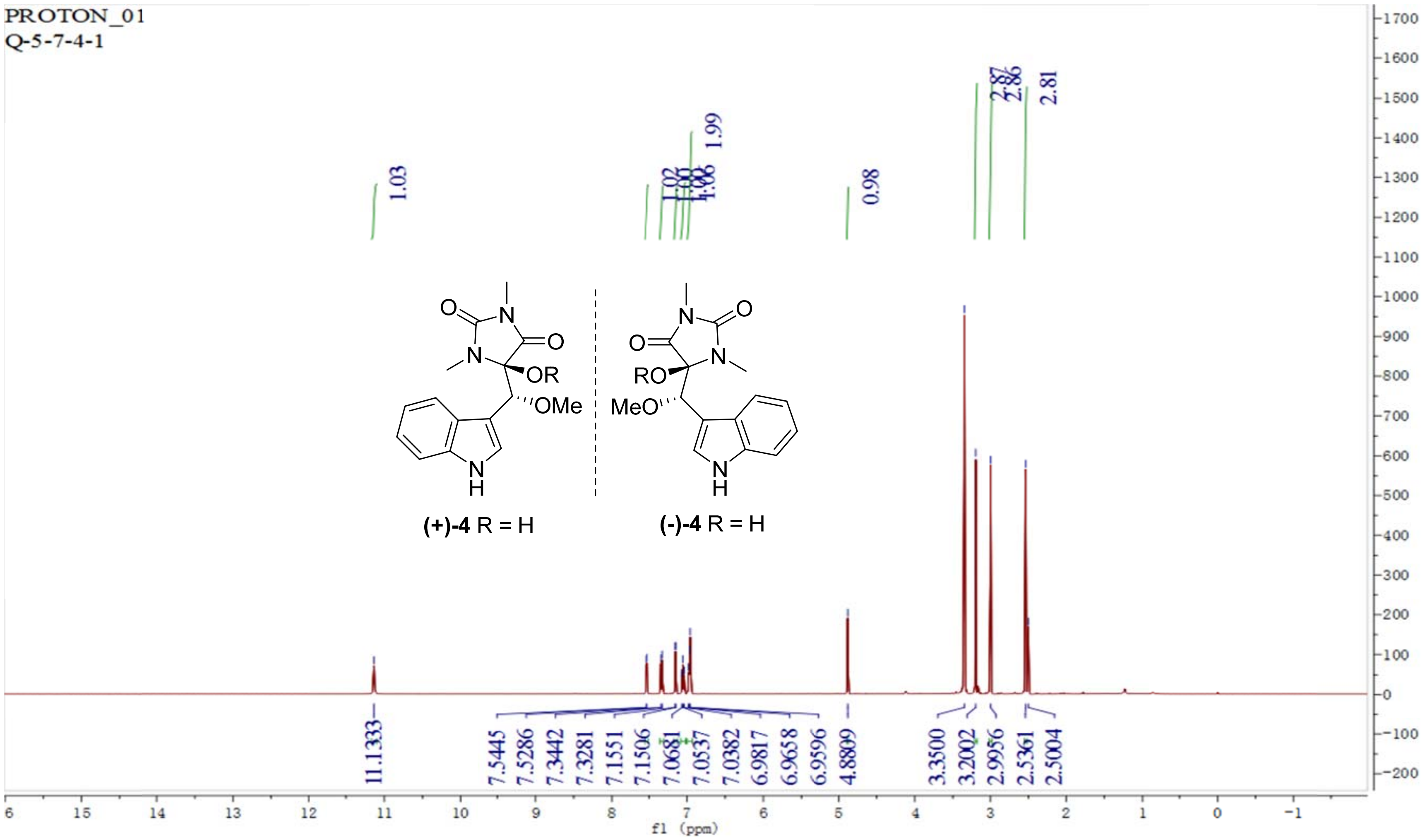

Figure S24. ^{1}H NMR (500 MHz, DMSO-d_6) spectrum of **4**.

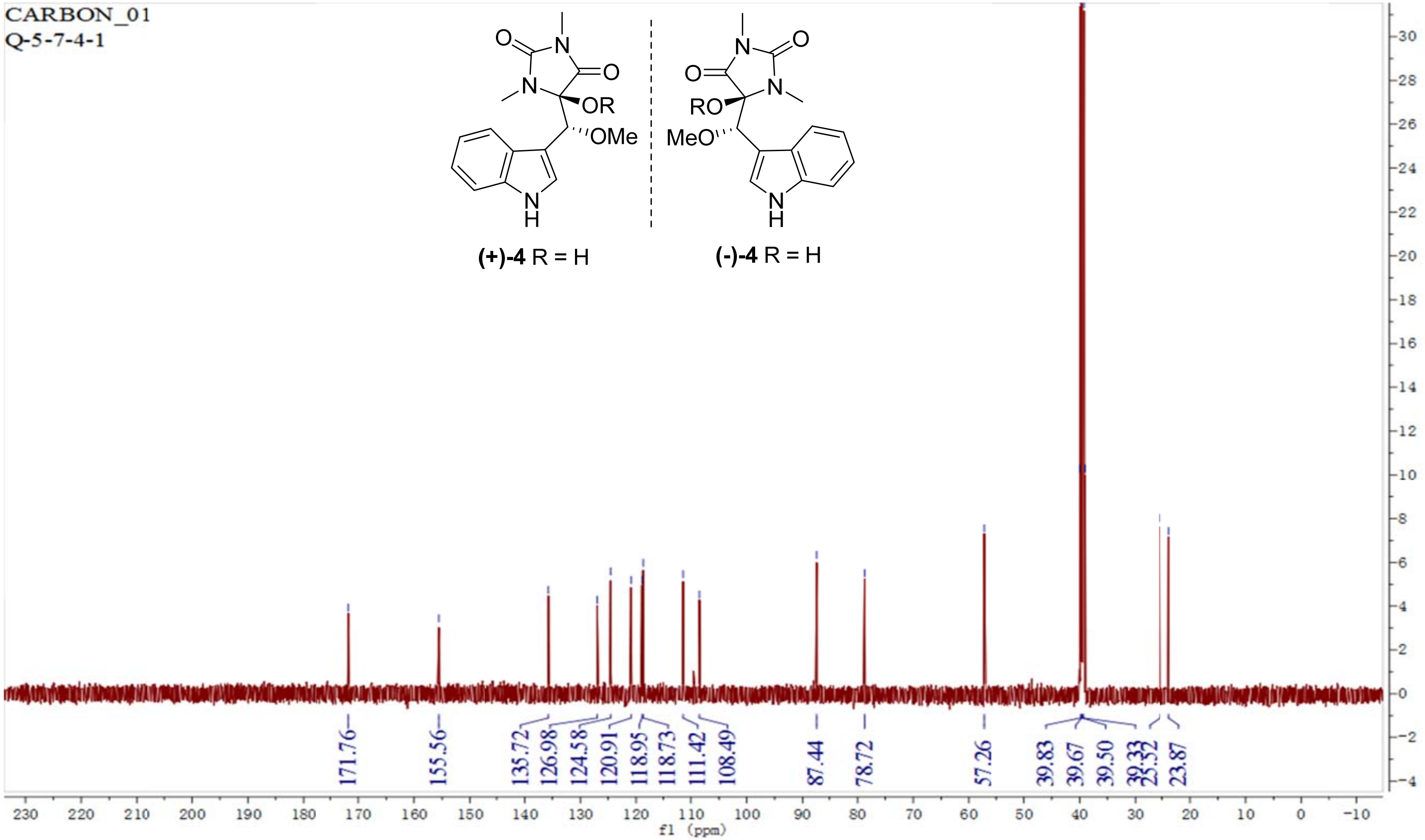

Figure S25. ^{13}C NMR (125 MHz, DMSO-d_6) spectrum of **4**.

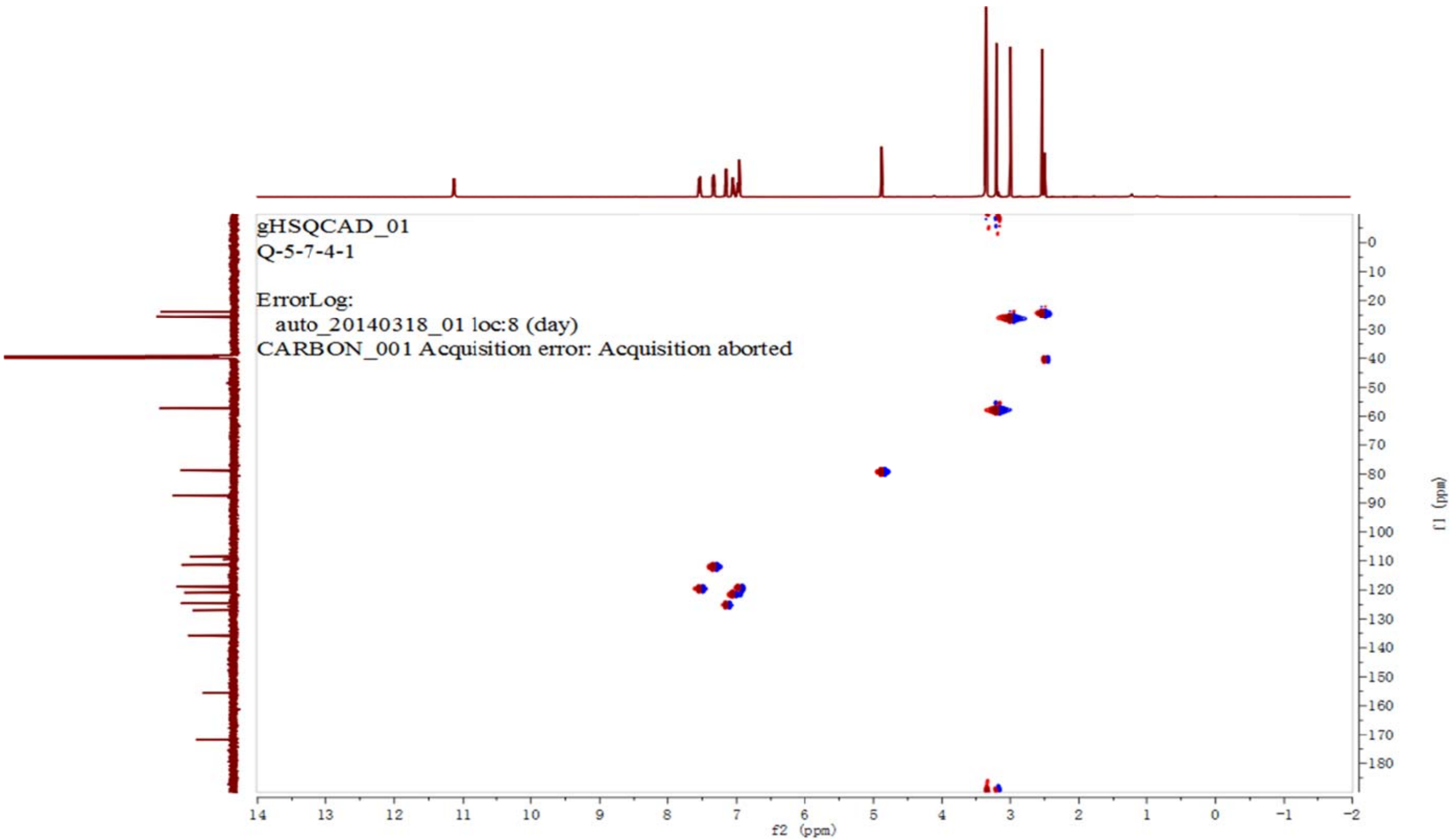

Figure S26. HSQC spectrum of Compound **4**.

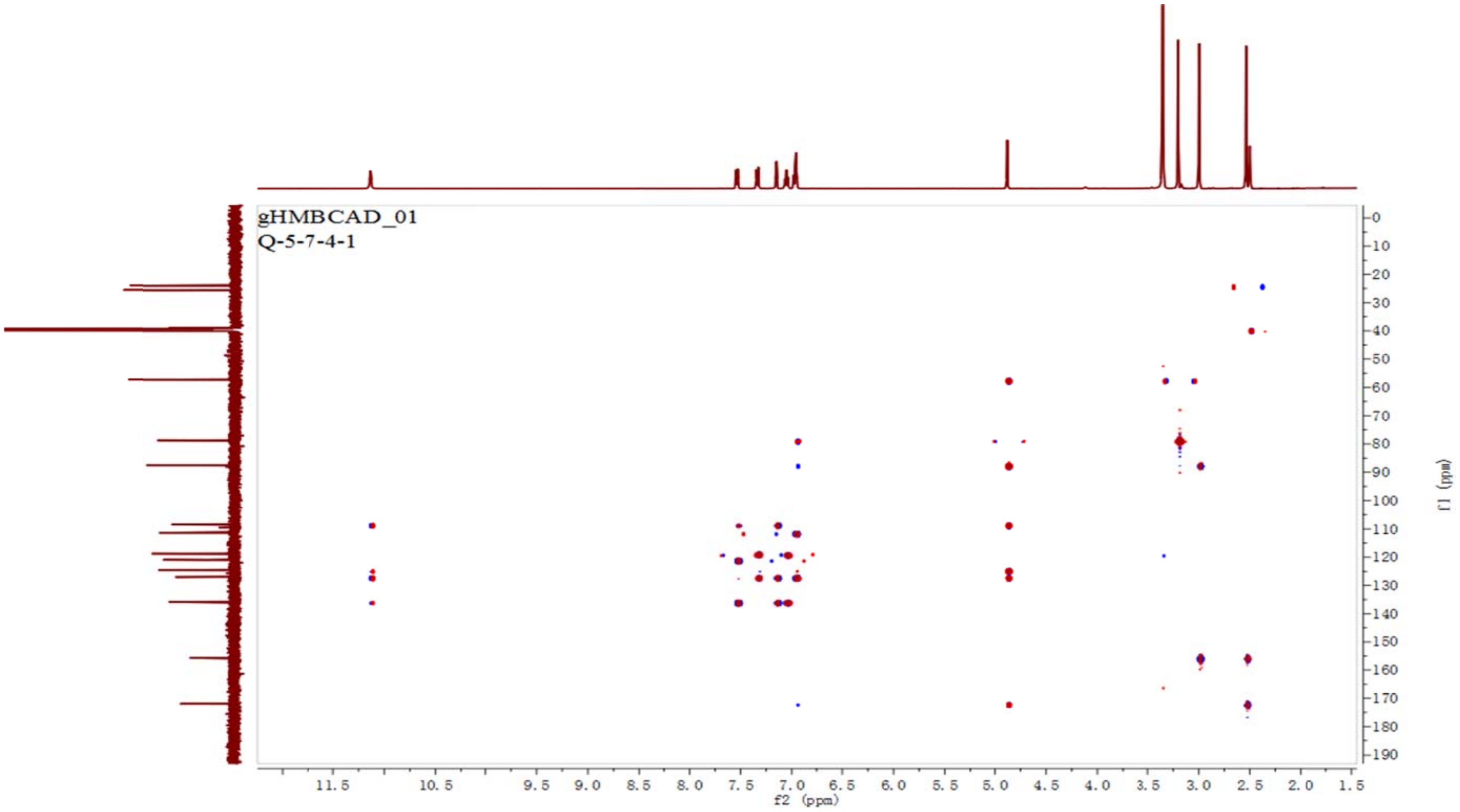

Figure S27. HMBC spectrum of Compound **4**.

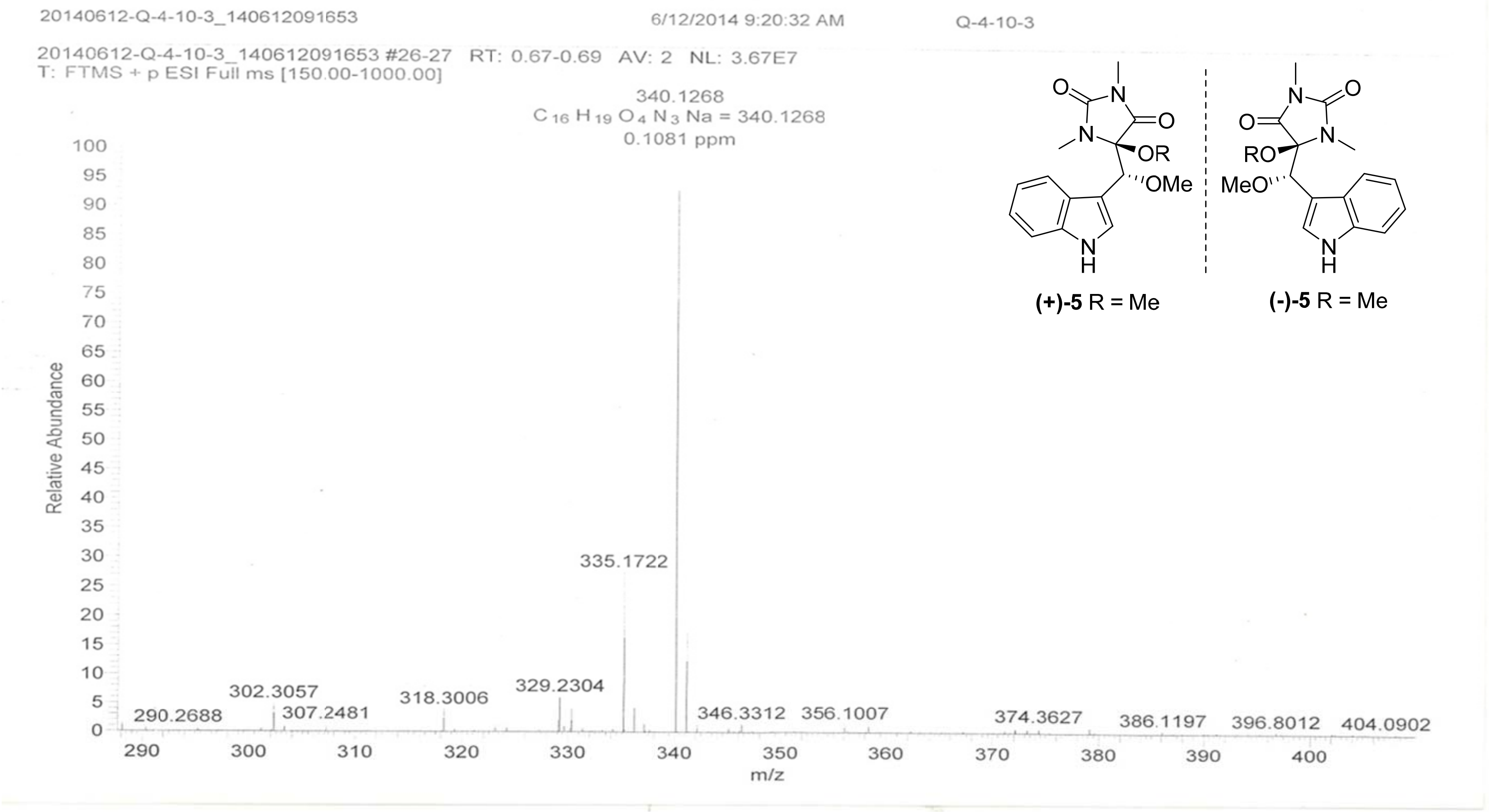

Figure S28. (+)-HRESIMS spectrum of **5**.

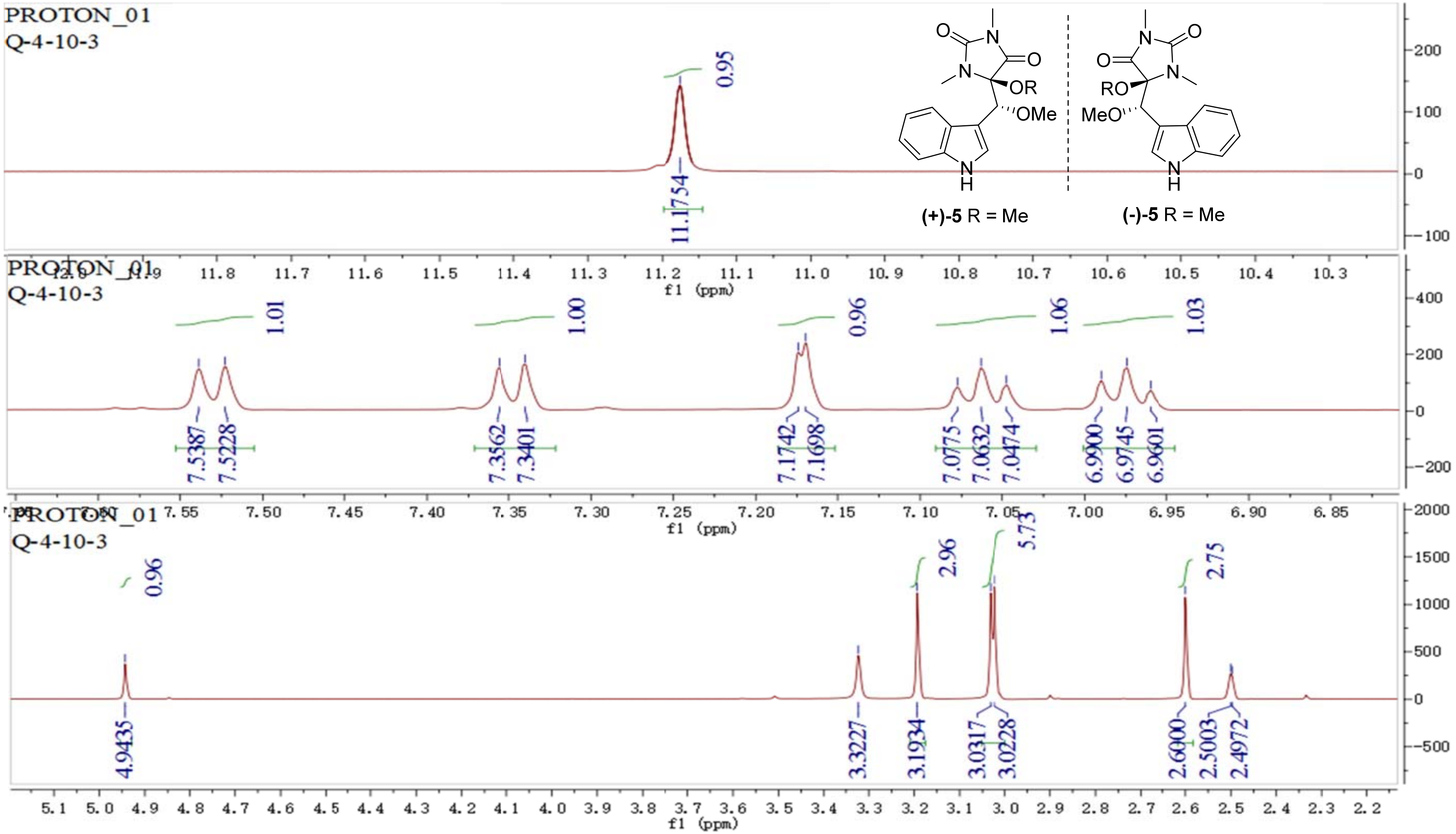

Figure S29. ^{1}H NMR (500 MHz, DMSO-d_6) spectrum of **5**.

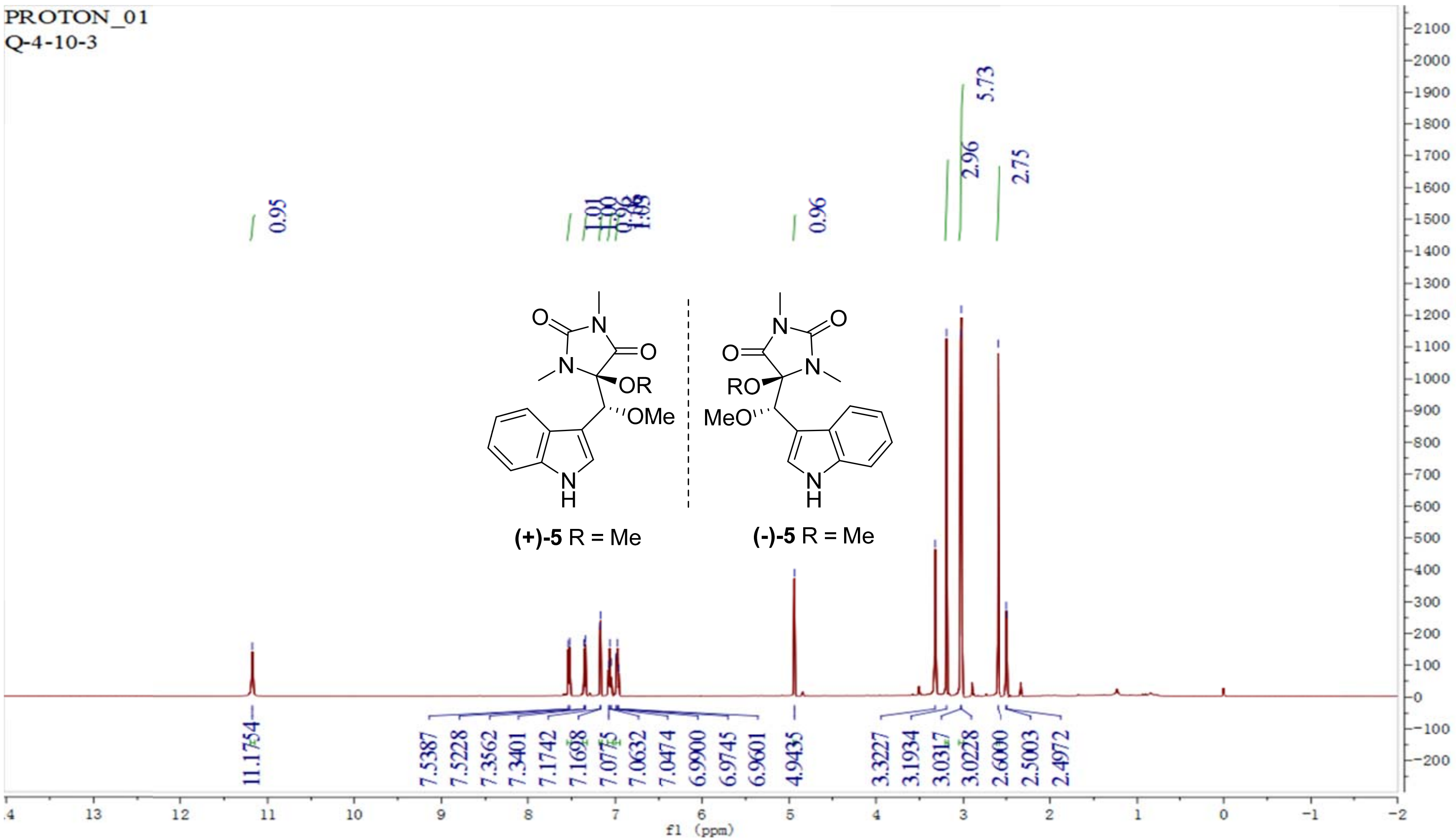

Figure S30. ^{1}H NMR (500 MHz, DMSO-d_6) spectrum of **5**.

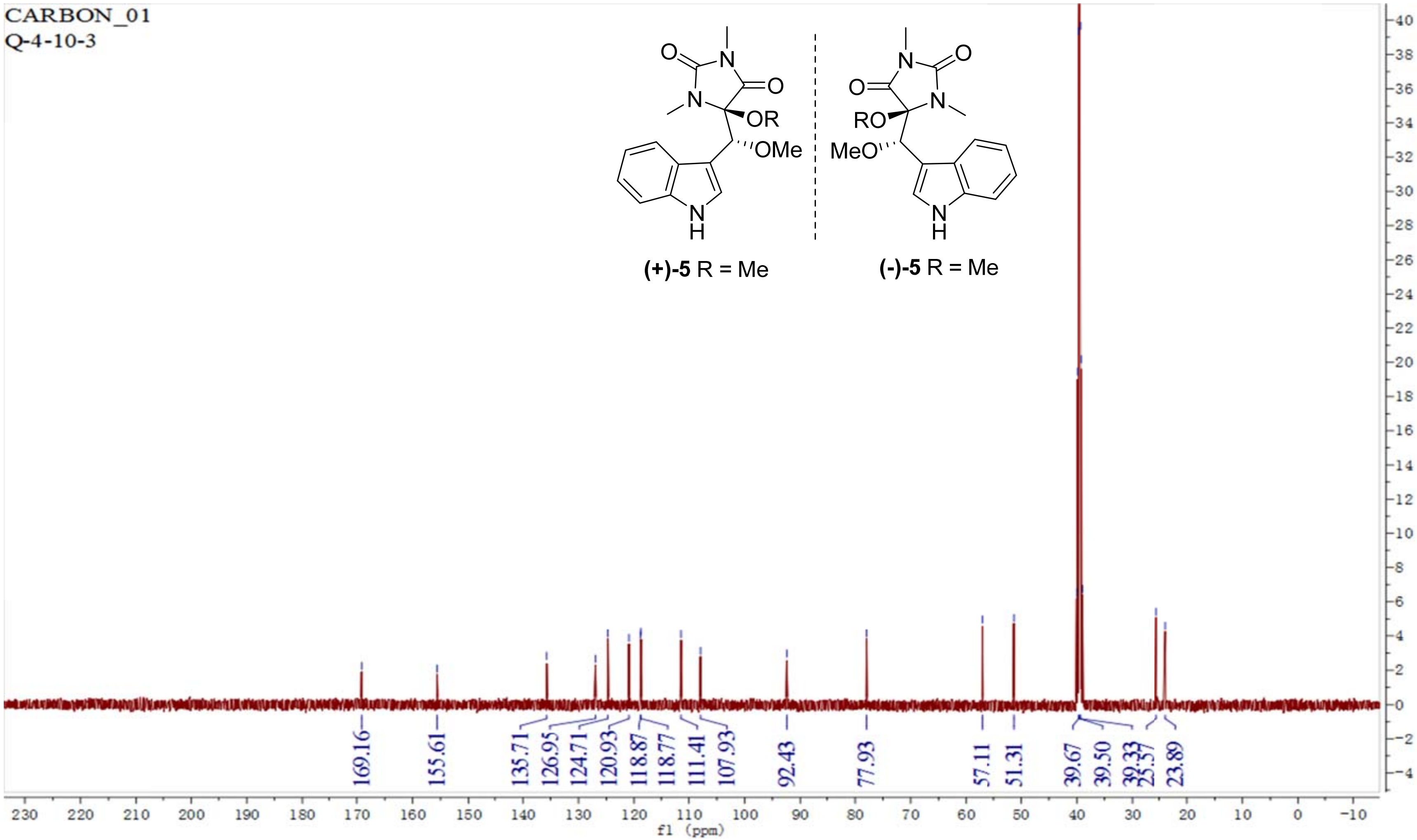

Figure S31. ^{13}C NMR (125 MHz, DMSO-d_6) spectrum of **5**.

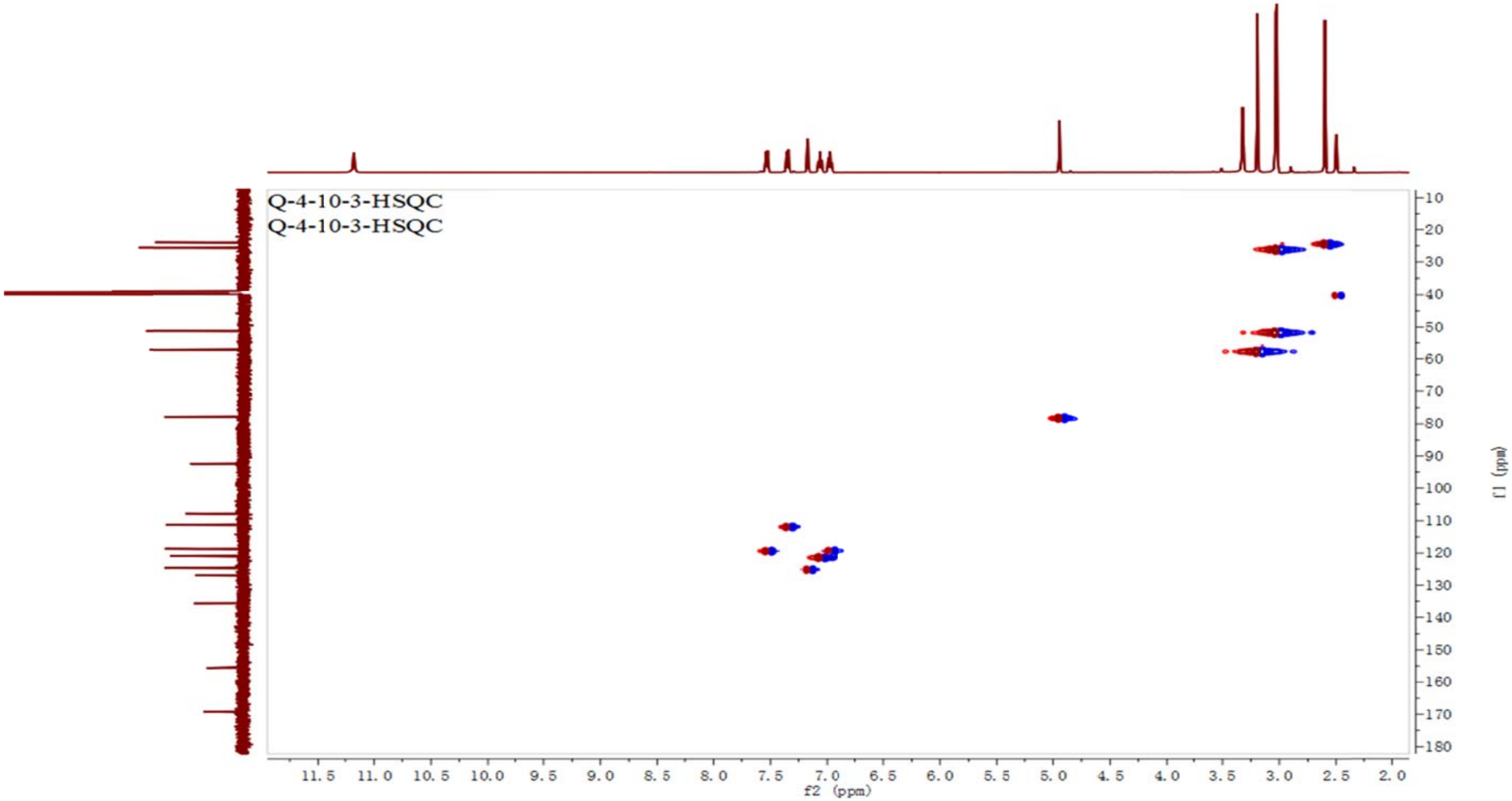

Figure S32. HSQC spectrum of Compound **5**.

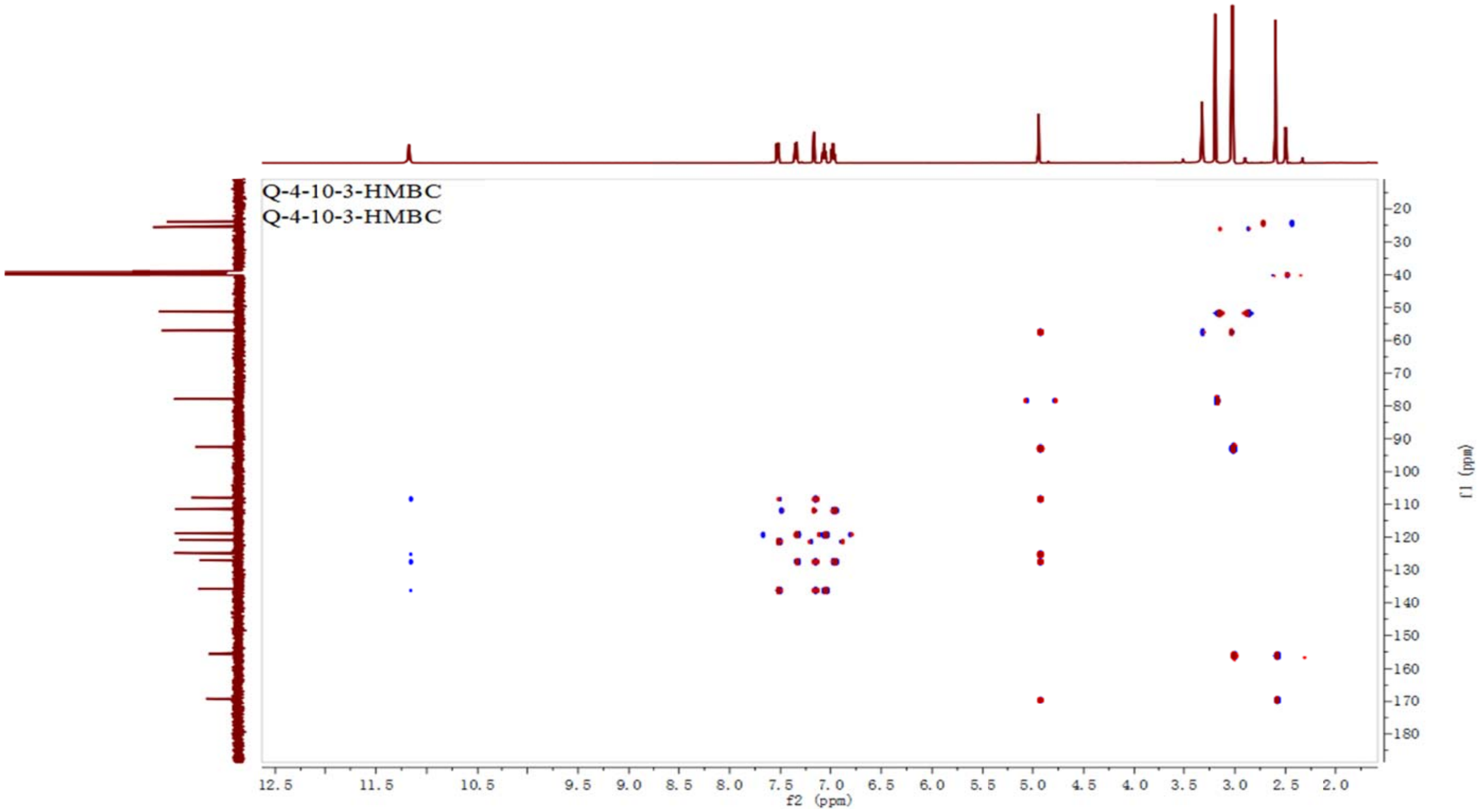

Figure S33. HMBC spectrum of Compound **5**.

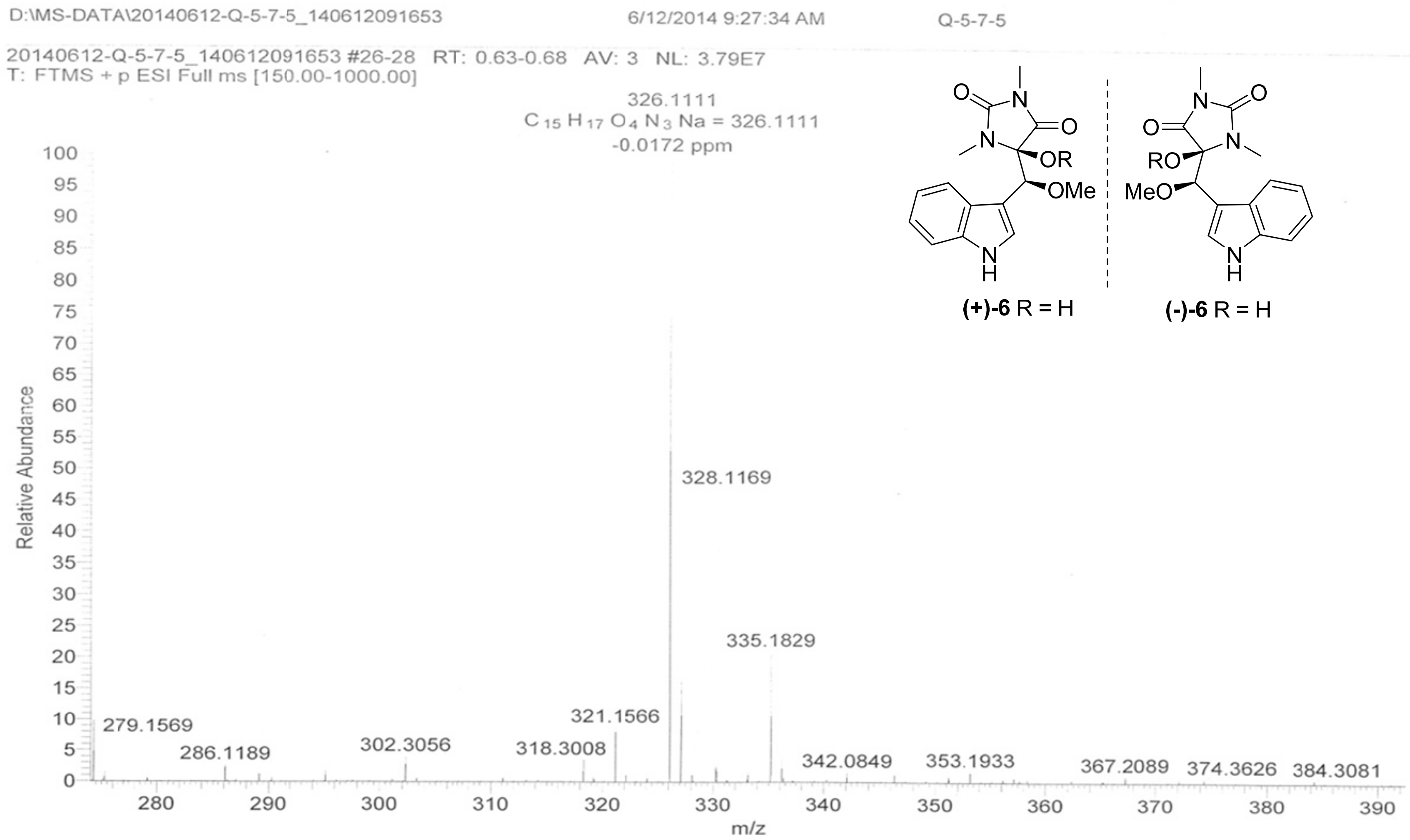

Figure S34. (+)-HRESIMS spectrum of **6**.

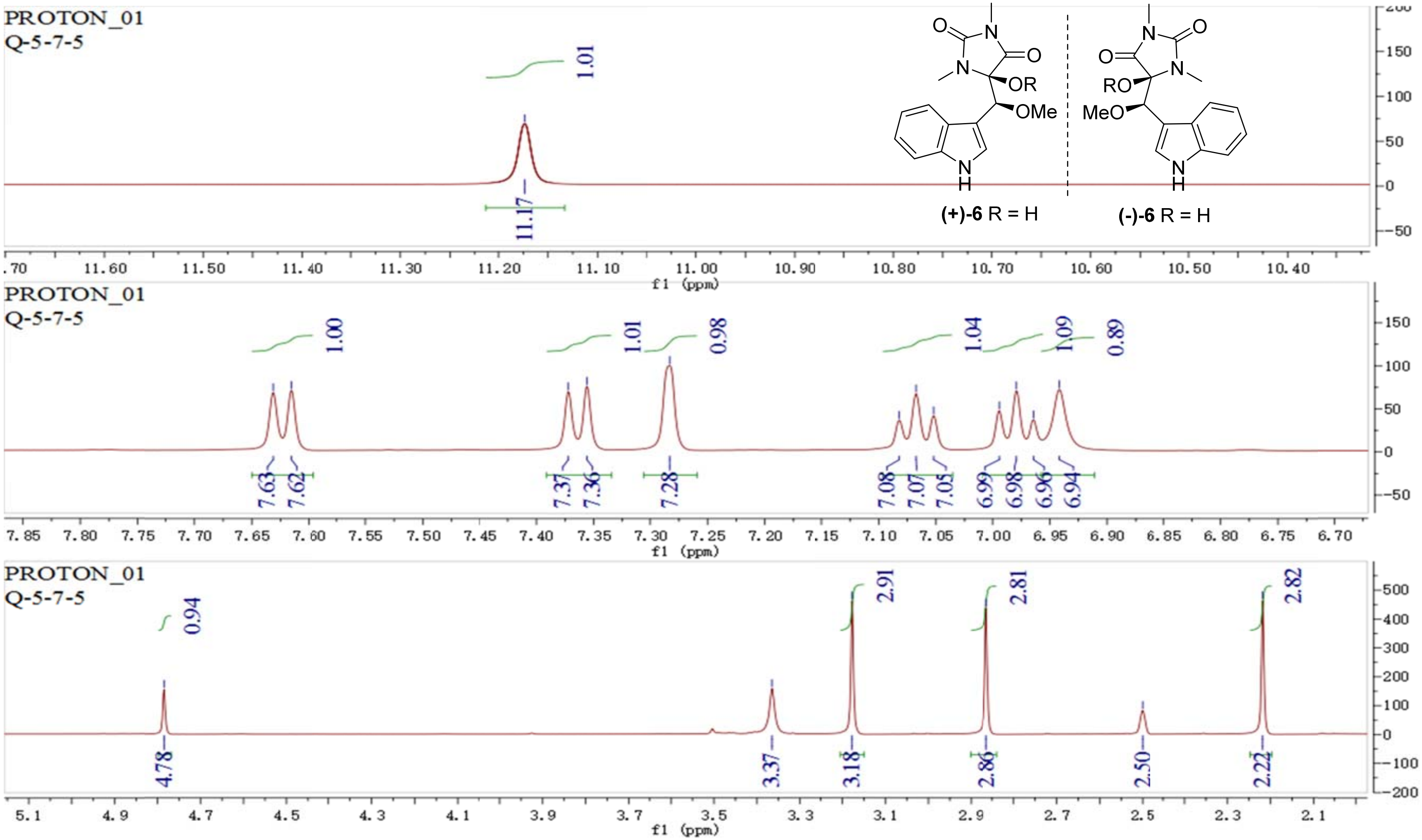

Figure S35. ^{1}H NMR (500 MHz, DMSO-d_6) spectrum of **6**.

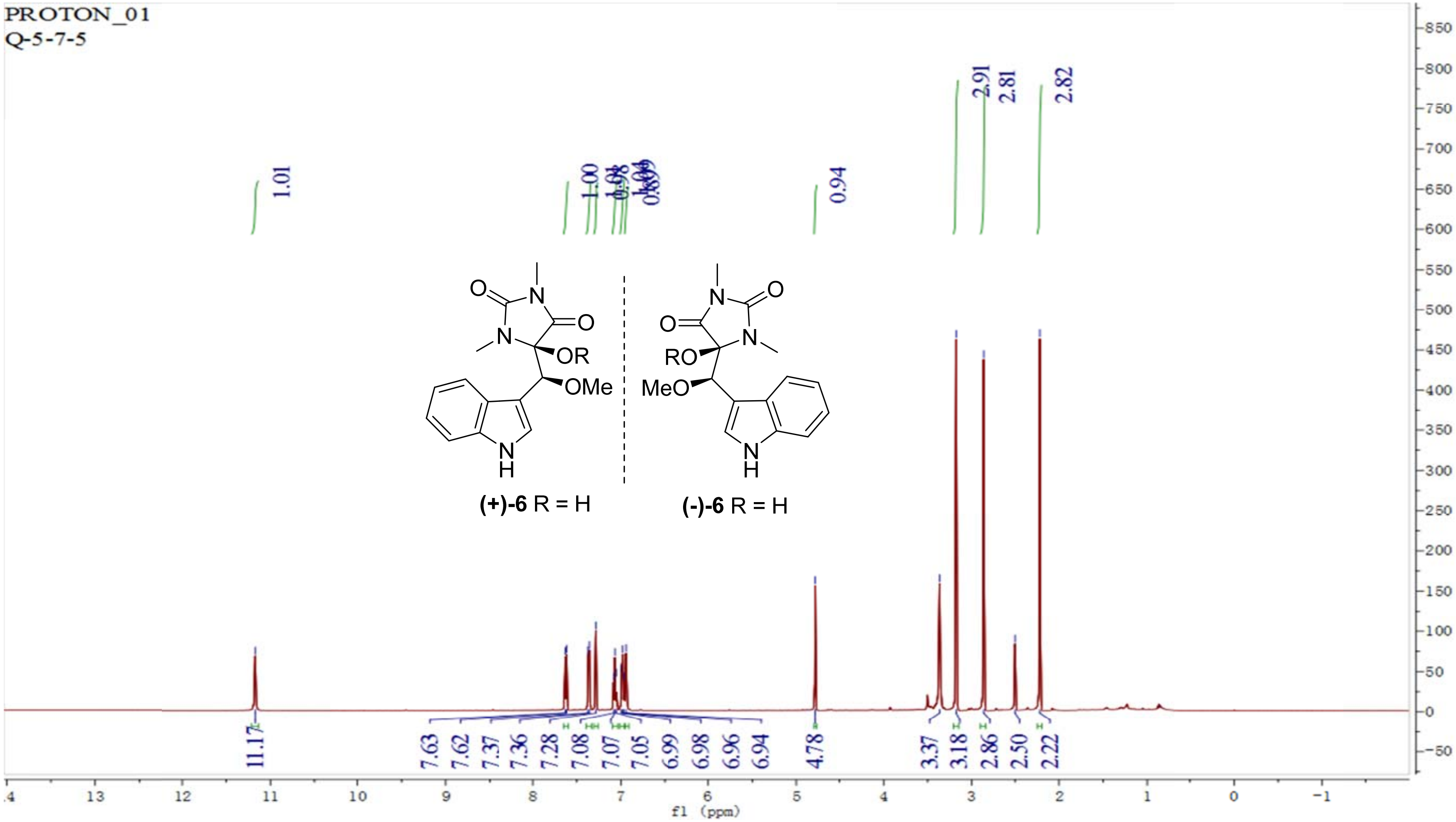

Figure S36. ^{1}H NMR (500 MHz, DMSO-d_6) spectrum of **6**.

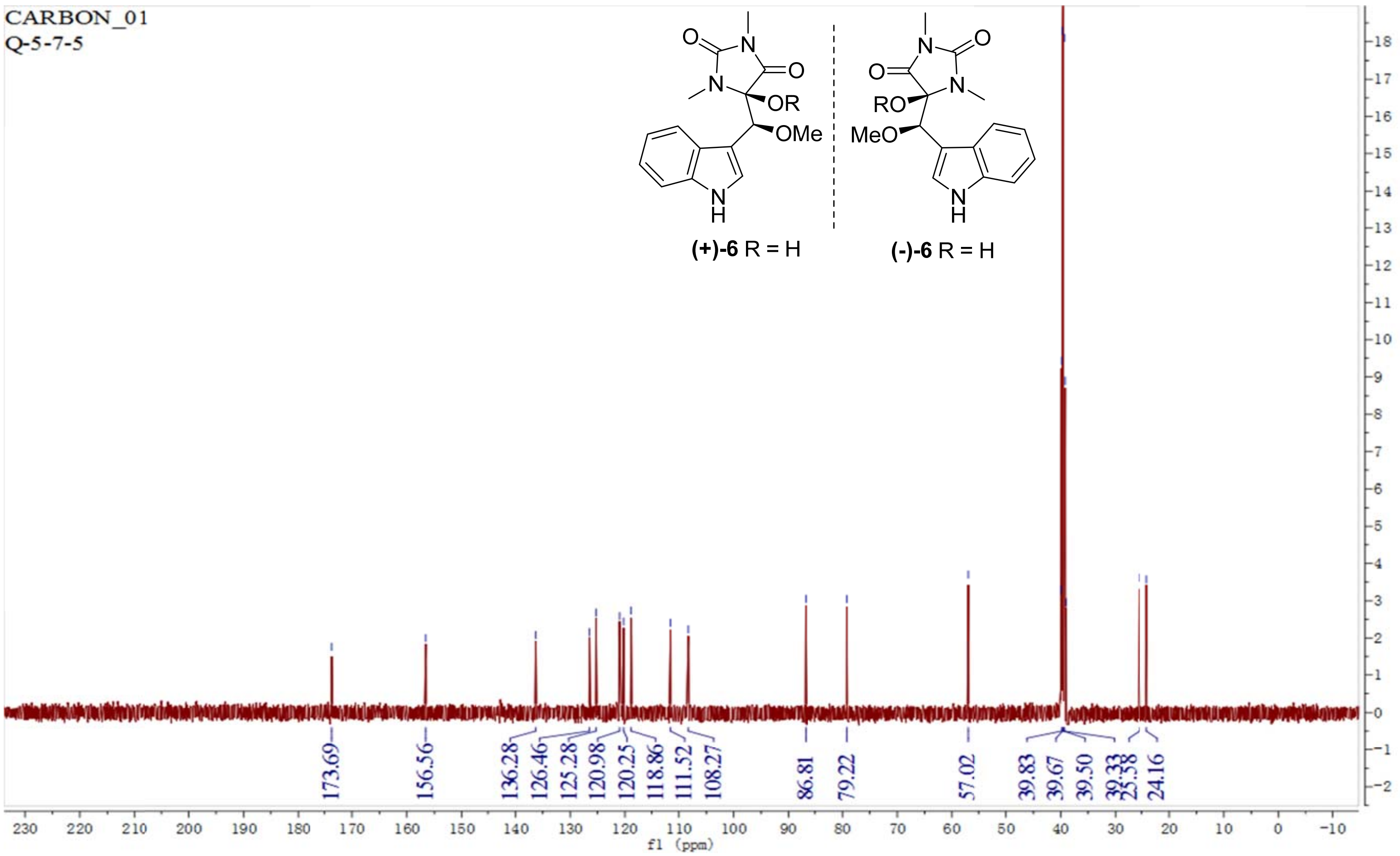

Figure S37. ^{13}C NMR (125 MHz, DMSO-d_6) spectrum of **6**.

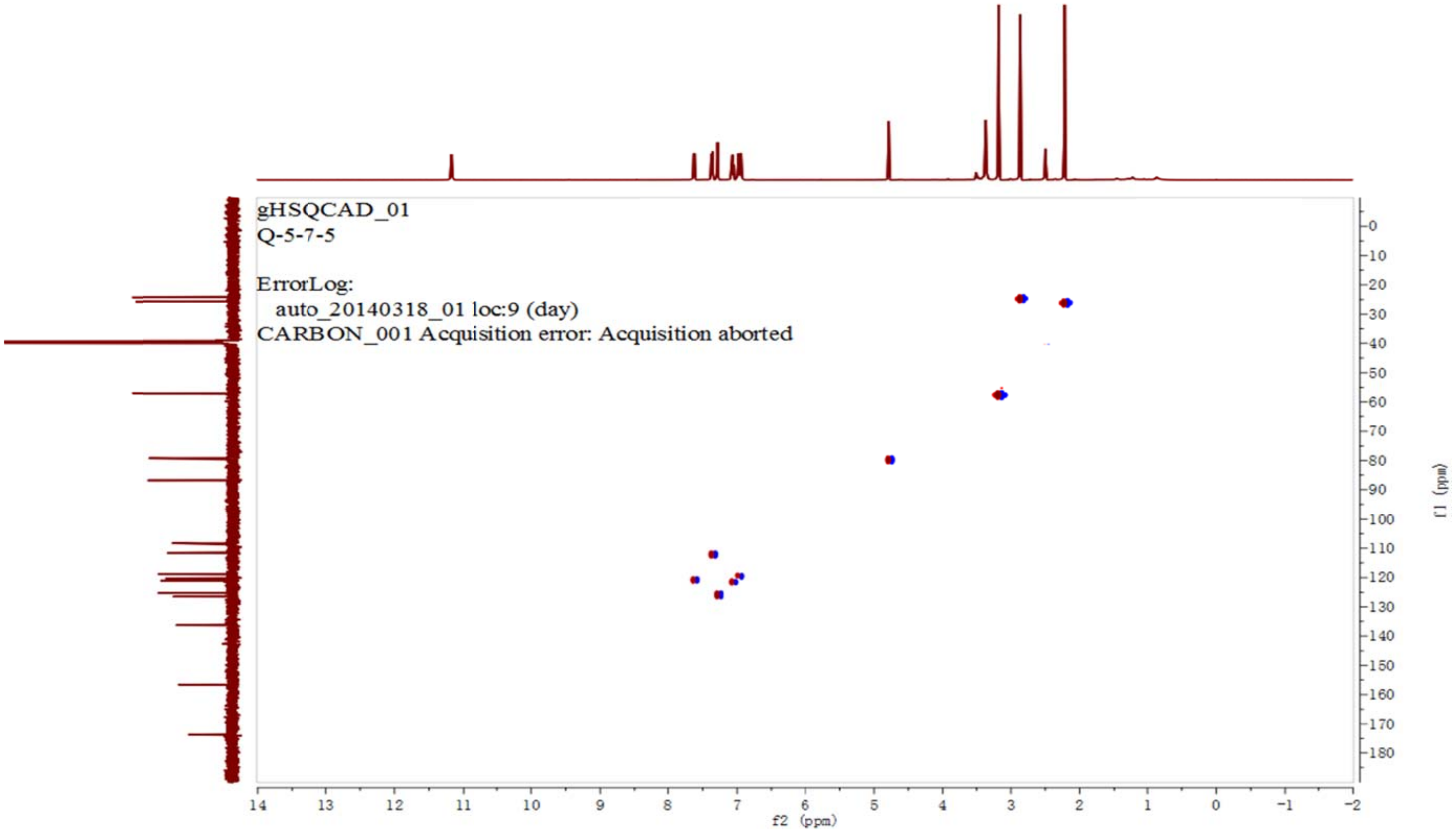

Figure S38. HSQC spectrum of Compound **6**.

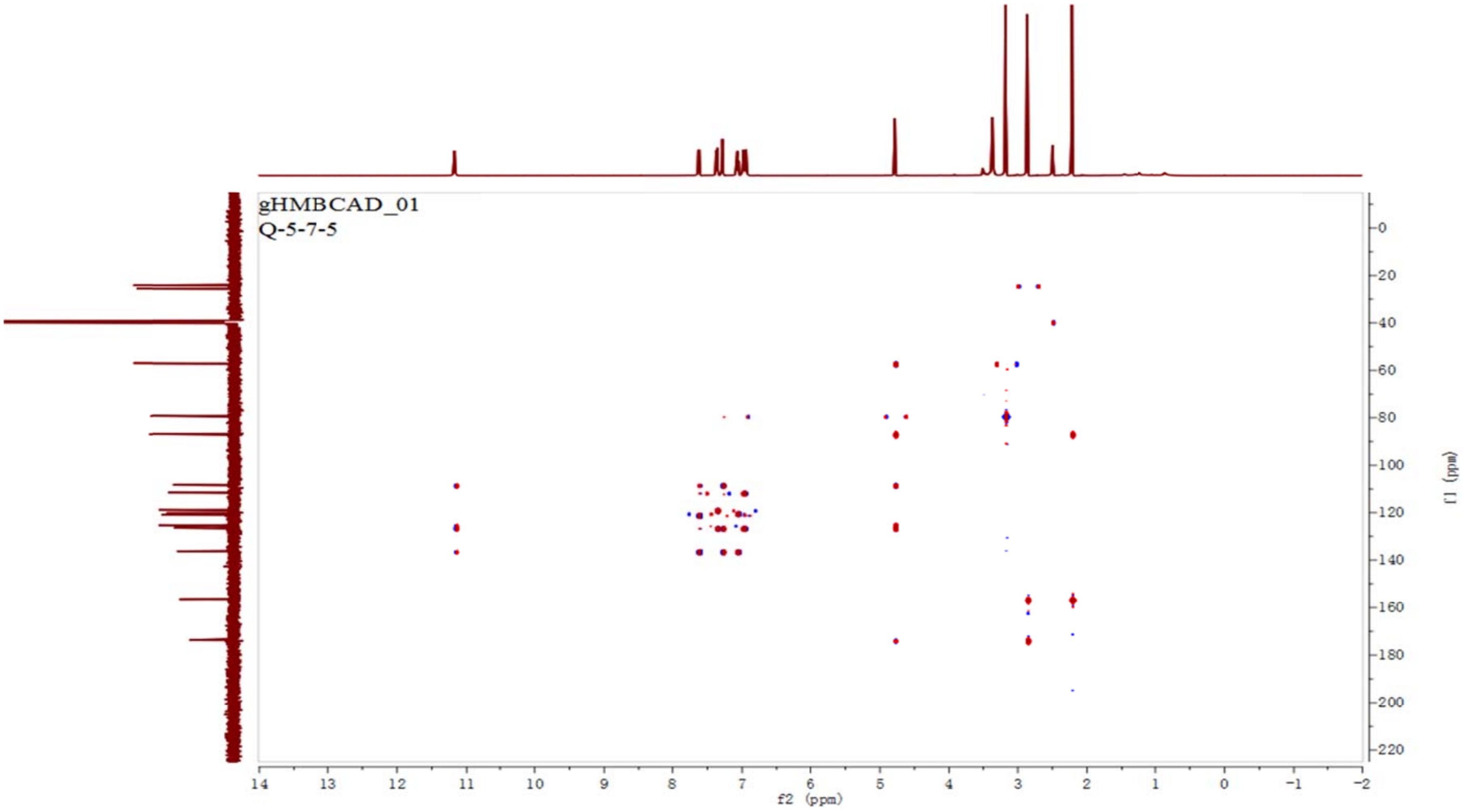

Figure S39. HMBC spectrum of Compound **6**.

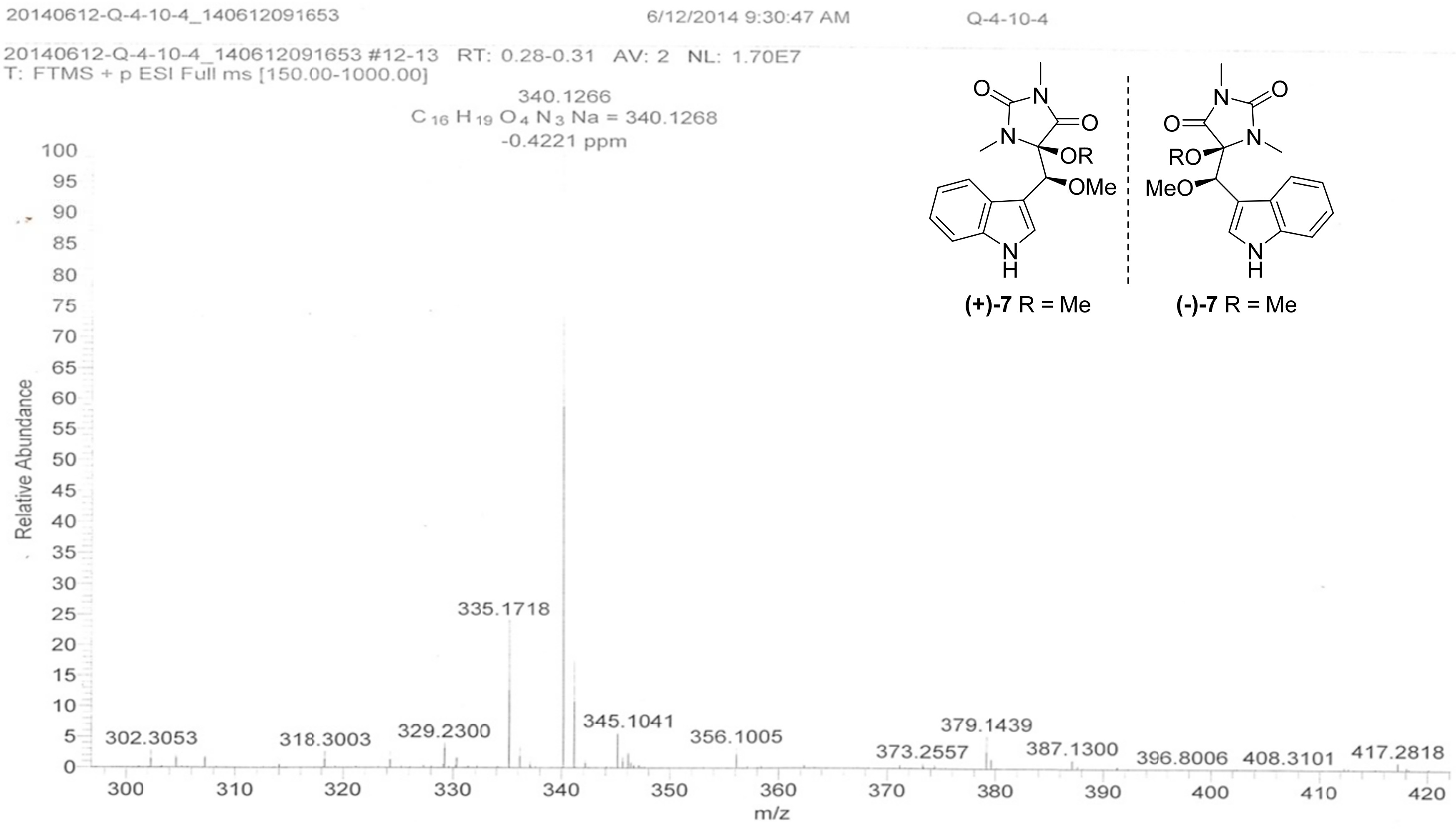

Figure S40. (+)-HRESIMS spectrum of **7**.

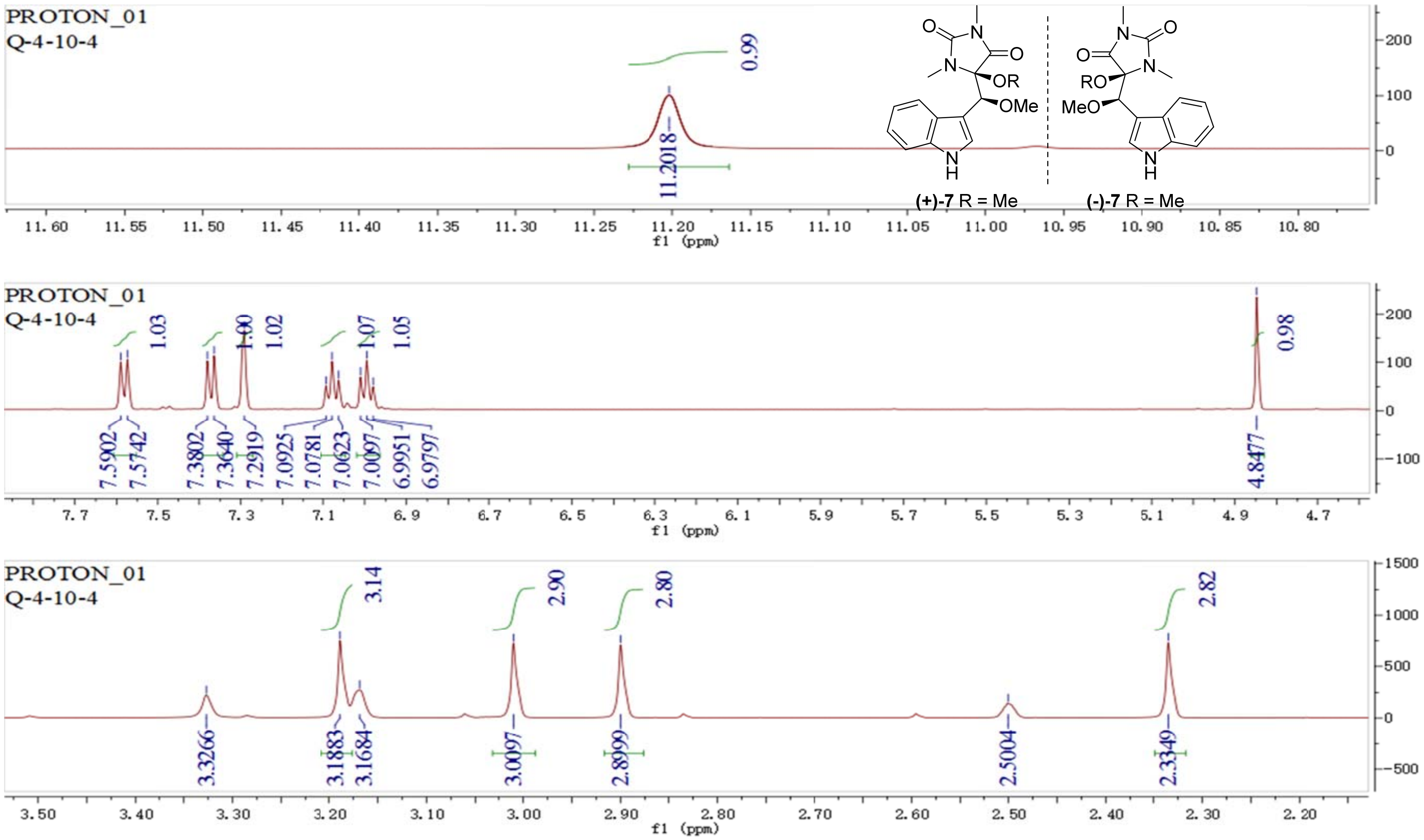

Figure S41. ^{1}H NMR (500 MHz, DMSO-d_6) spectrum of **7**.

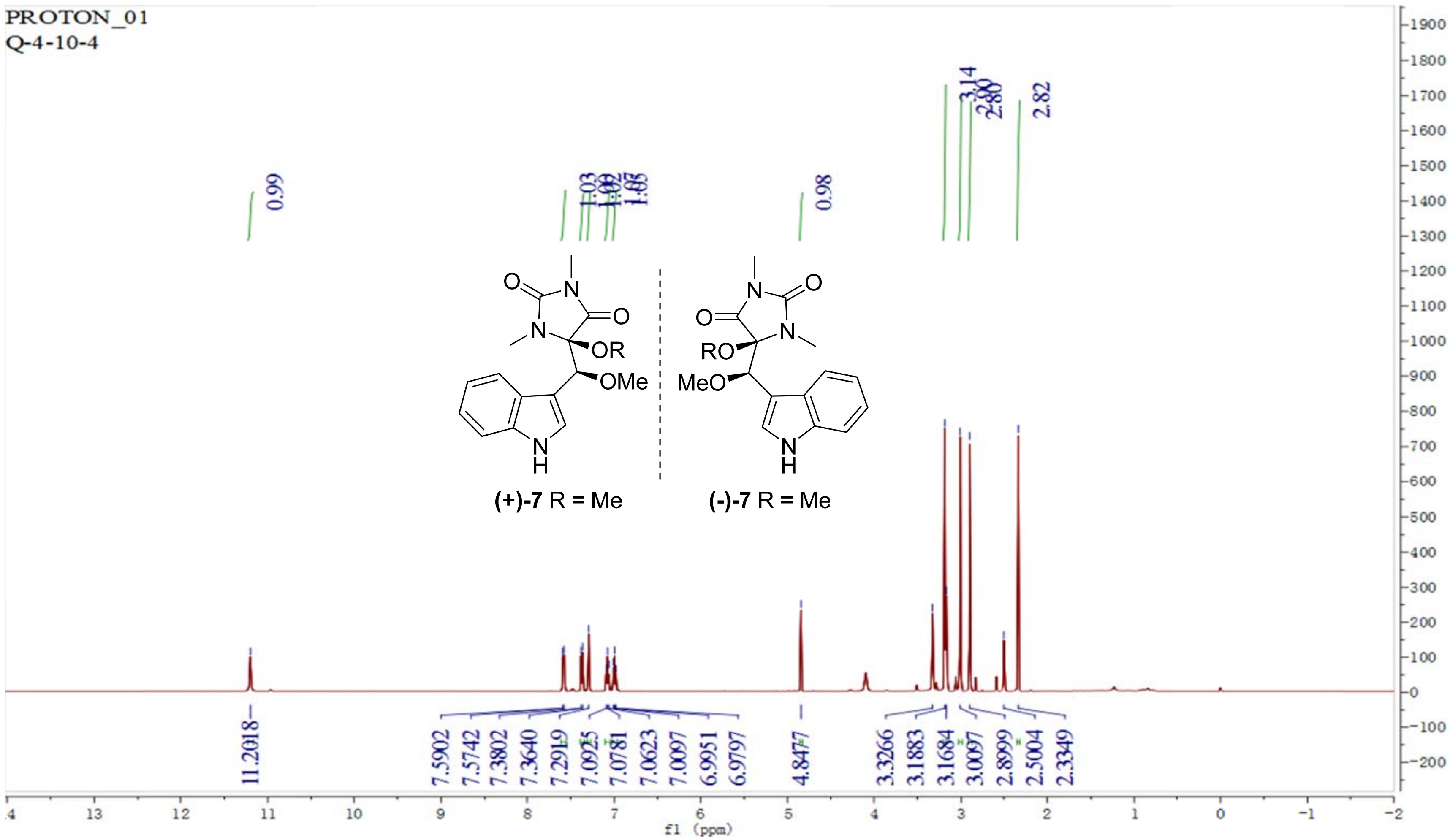

Figure S42. ^{1}H NMR (500 MHz, DMSO-d_6) spectrum of **7**.

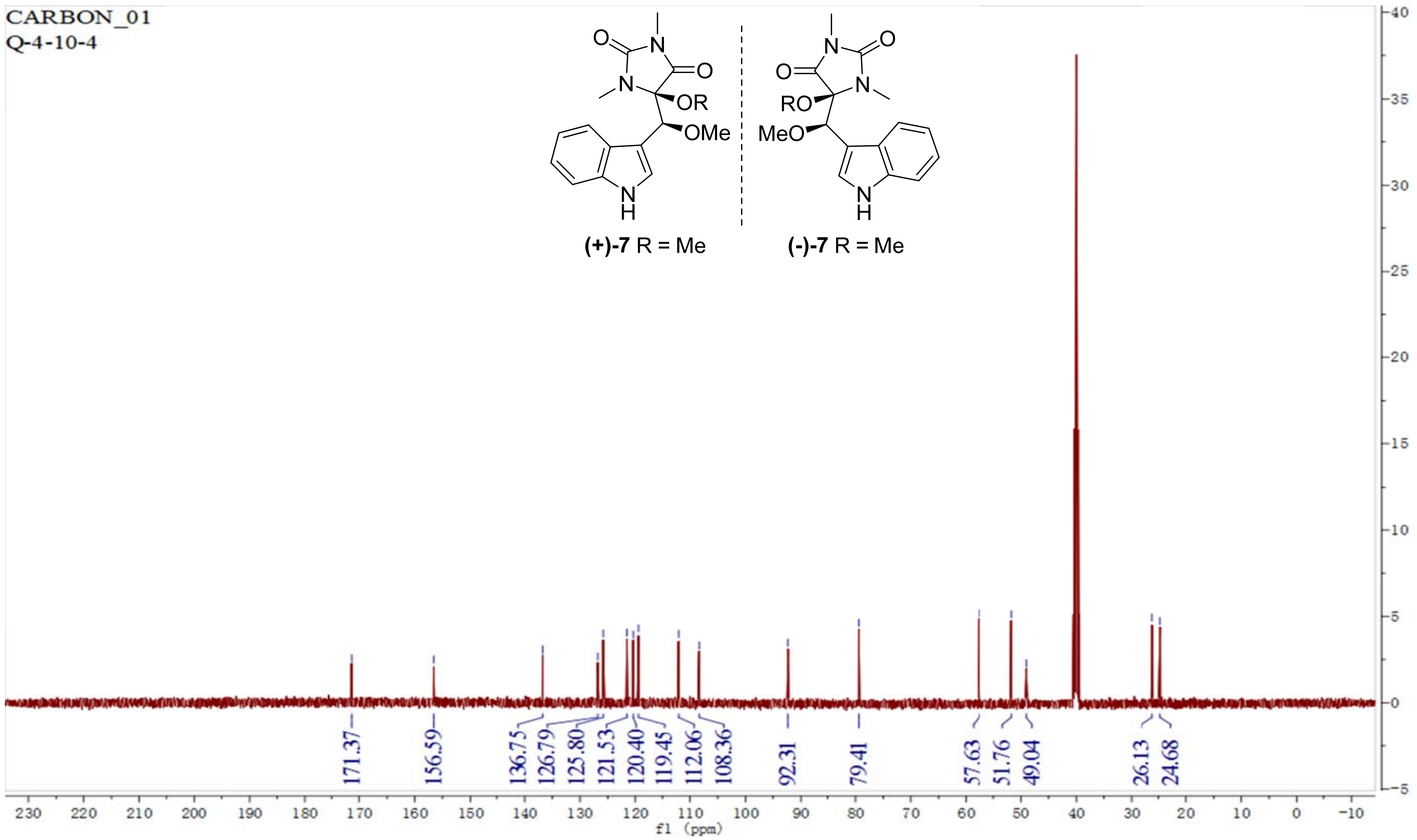

Figure S43. ^{13}C NMR (125 MHz, DMSO-d_6) spectrum of **7**.

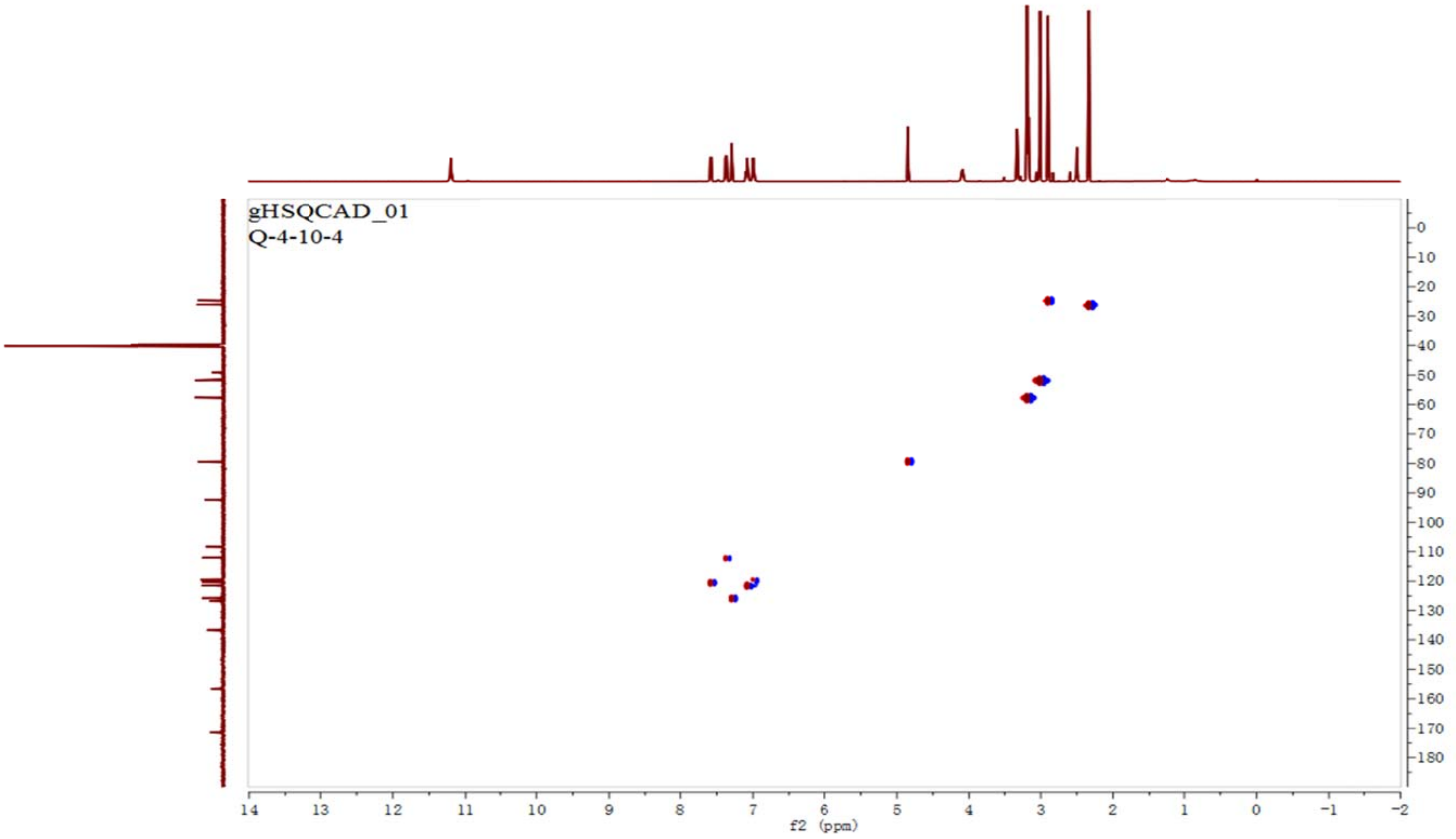

Figure S44. HSQC spectrum of Compound **7**.

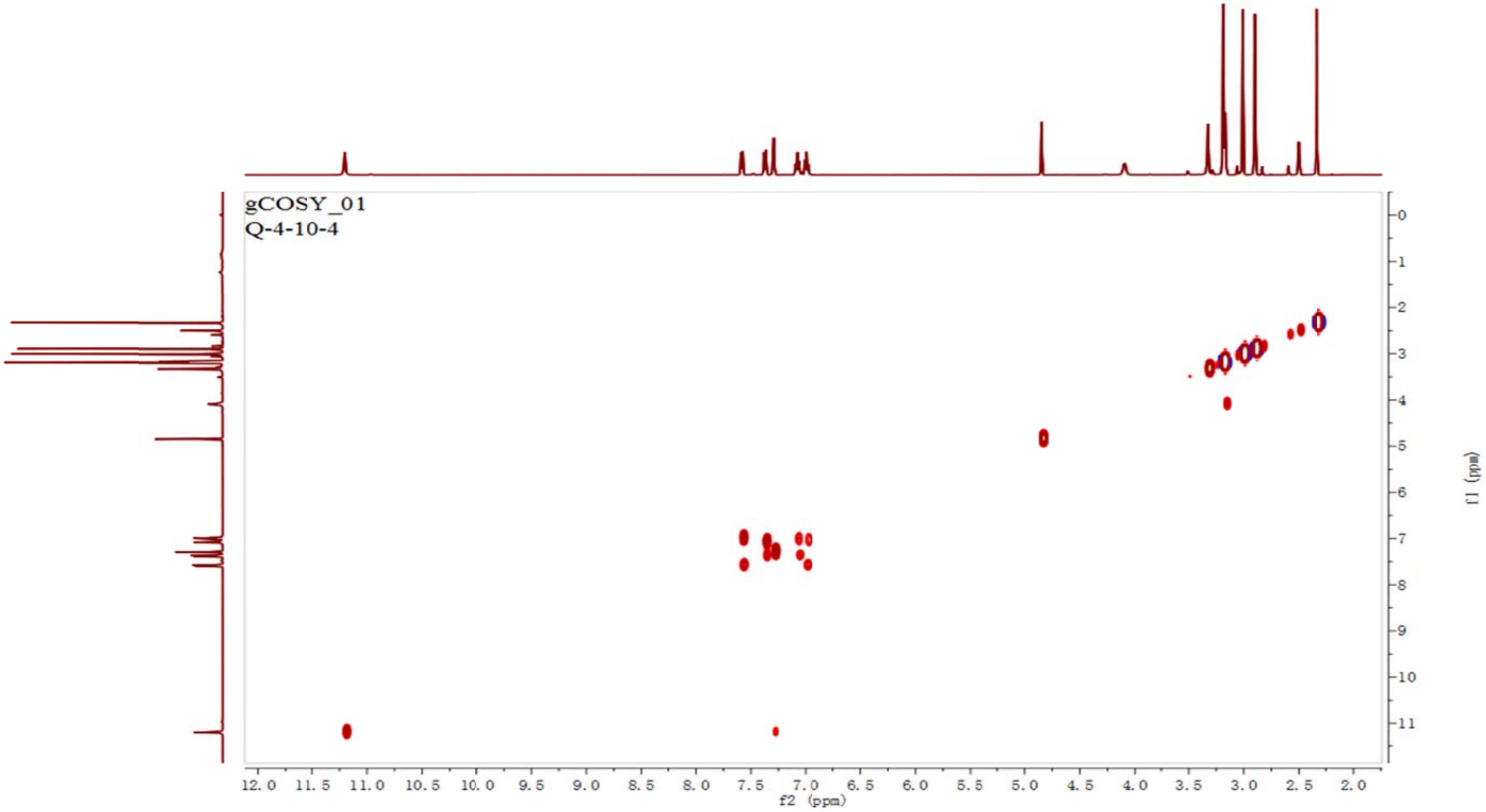

Figure S45. ^{1}H-^{1}H COSY spectrum of Compound **7**.

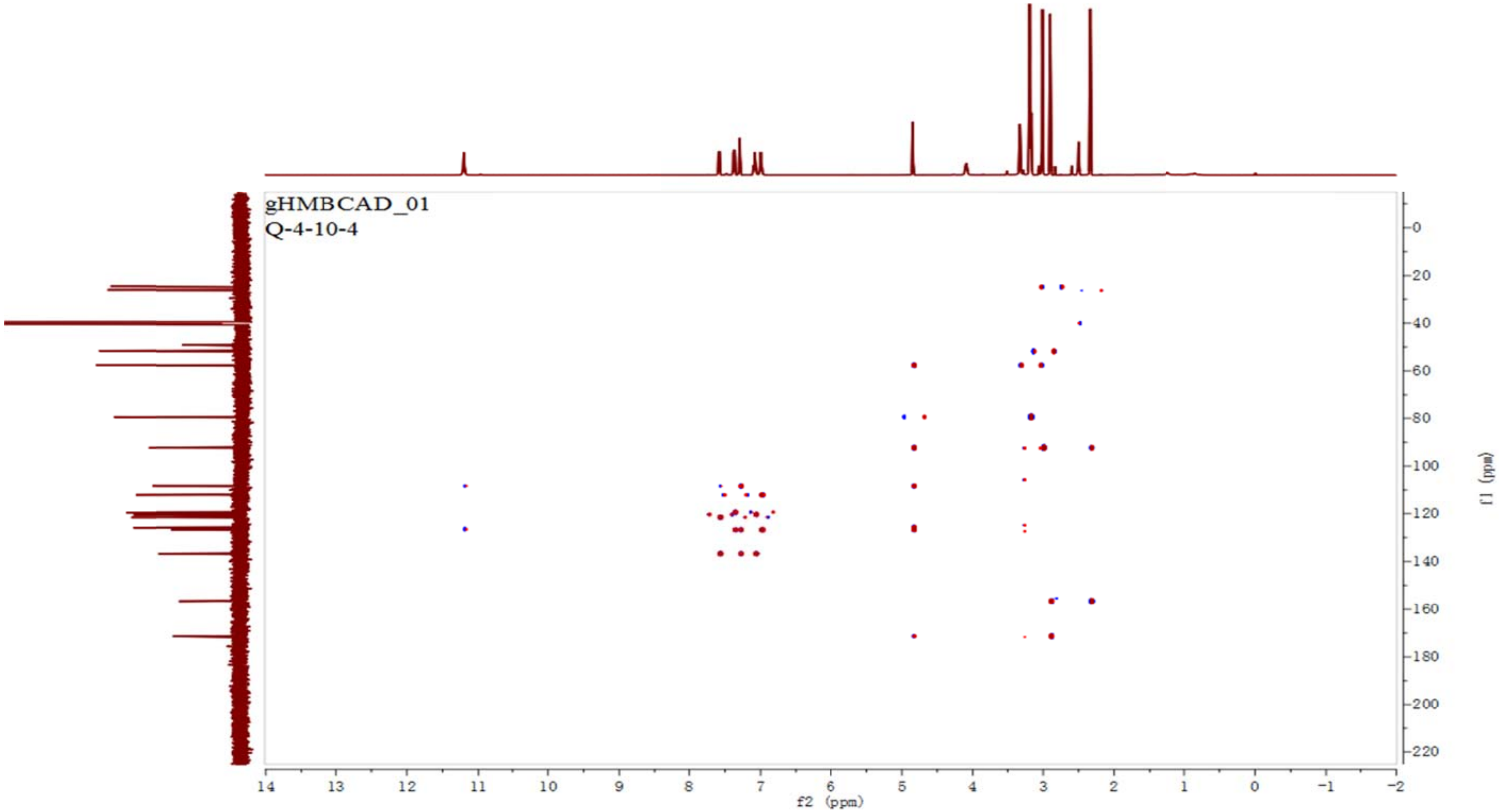

Figure S46. HMBC spectrum of Compound **7**.

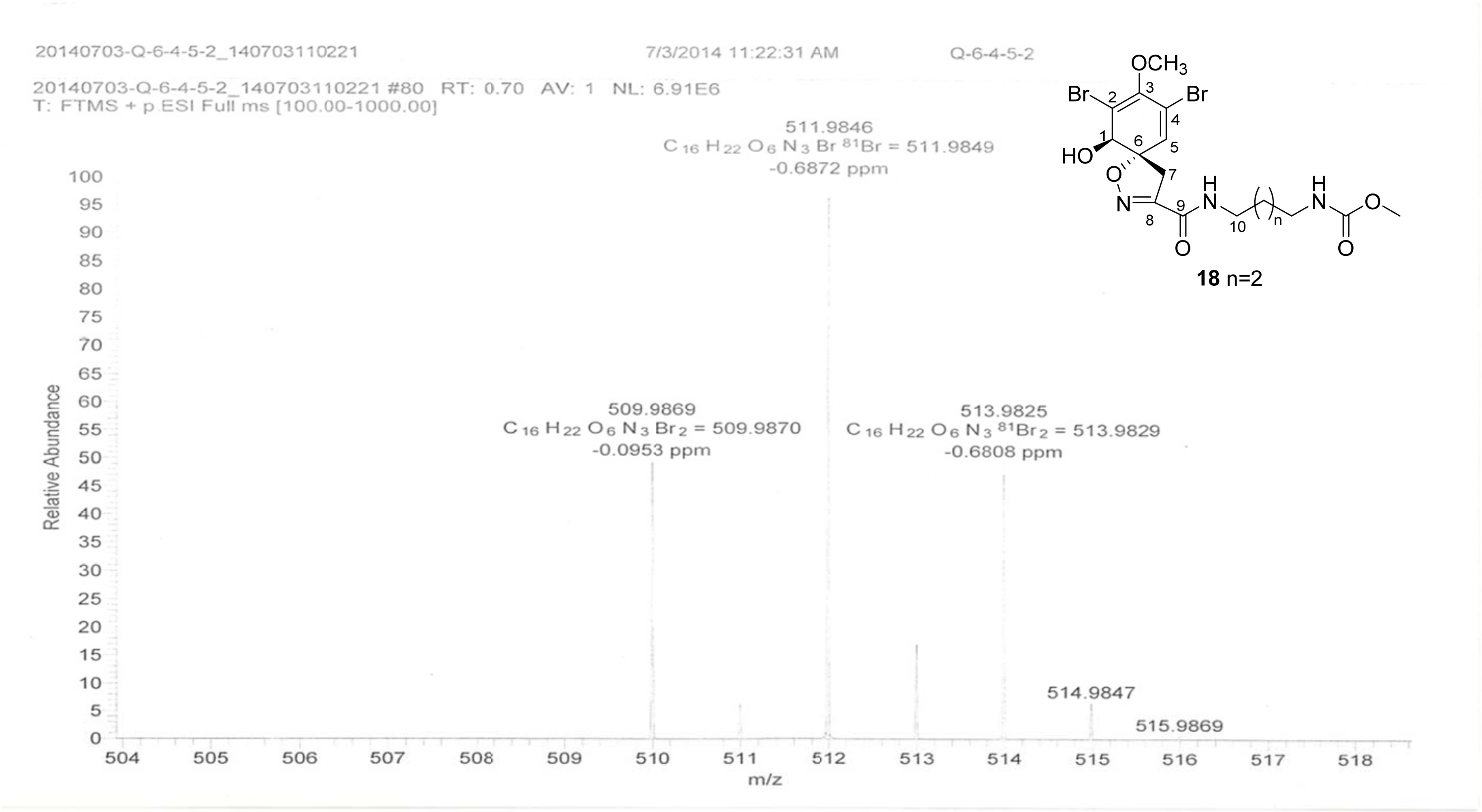

Figure S47. (+)-HRESIMS spectrum of **18**.

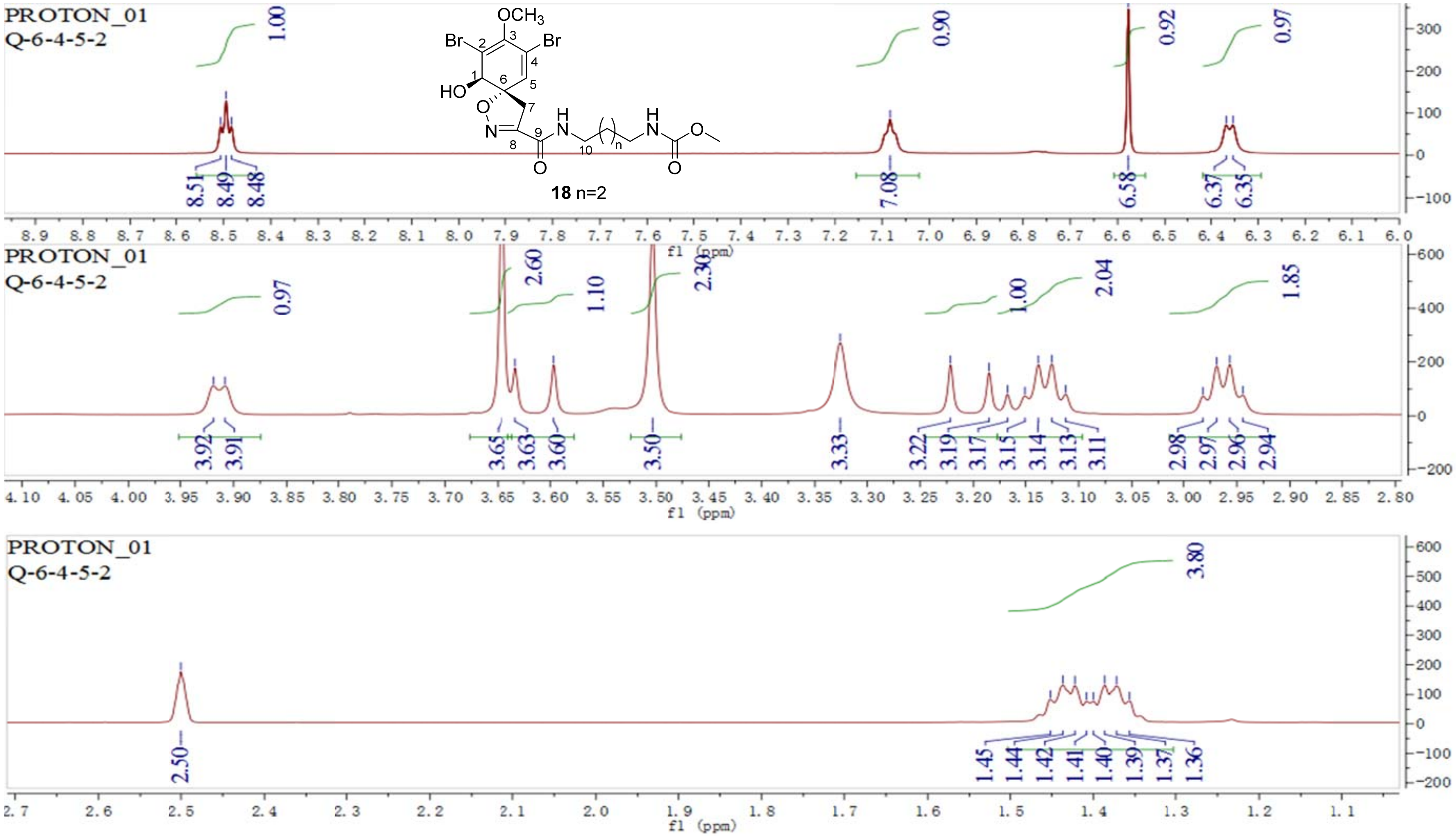

Figure S48. ^{1}H NMR (500 MHz, DMSO-d_6) spectrum of **18**.

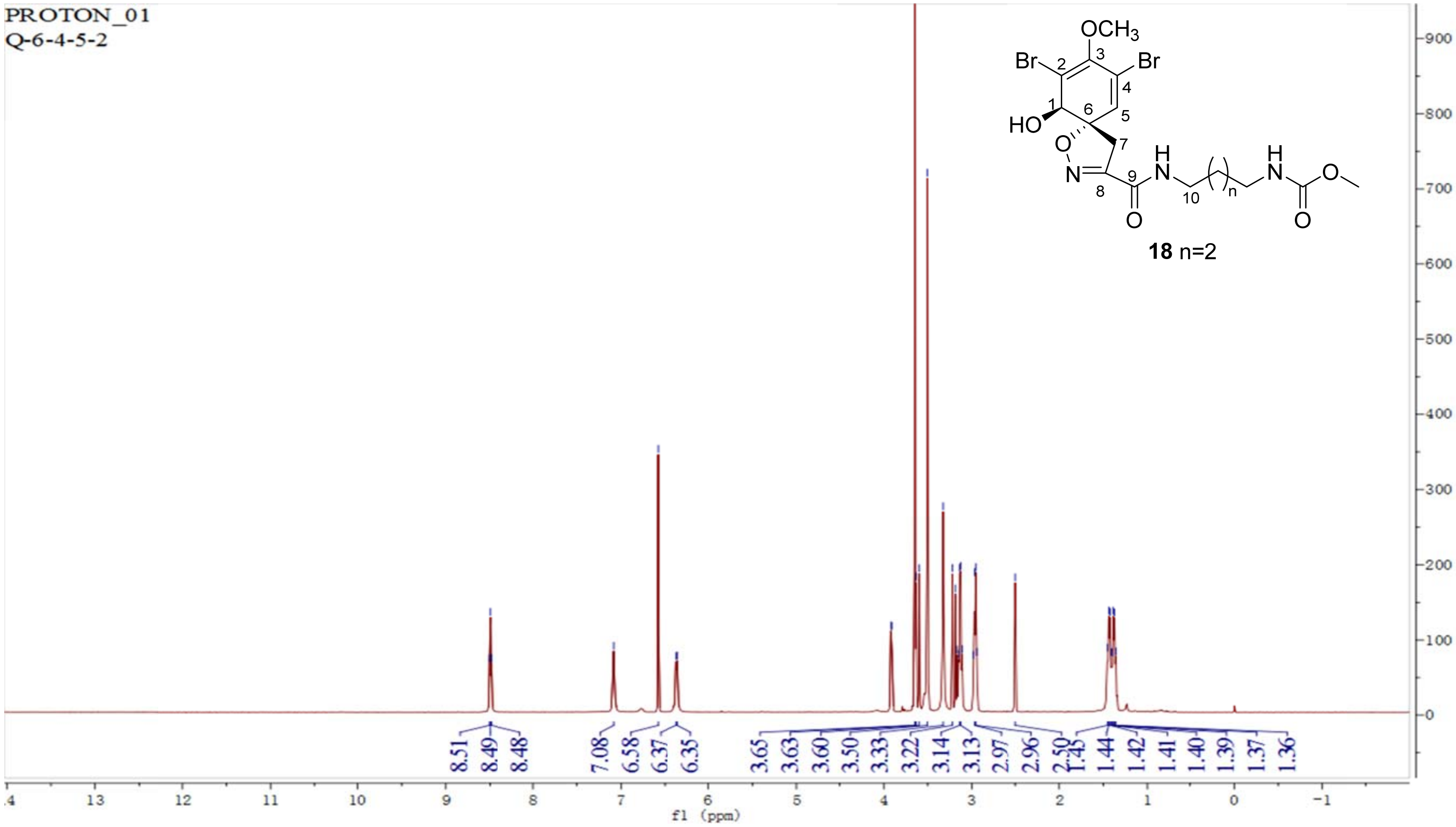

Figure S49. ^{1}H NMR (500 MHz, DMSO-d_6) spectrum of **18**.

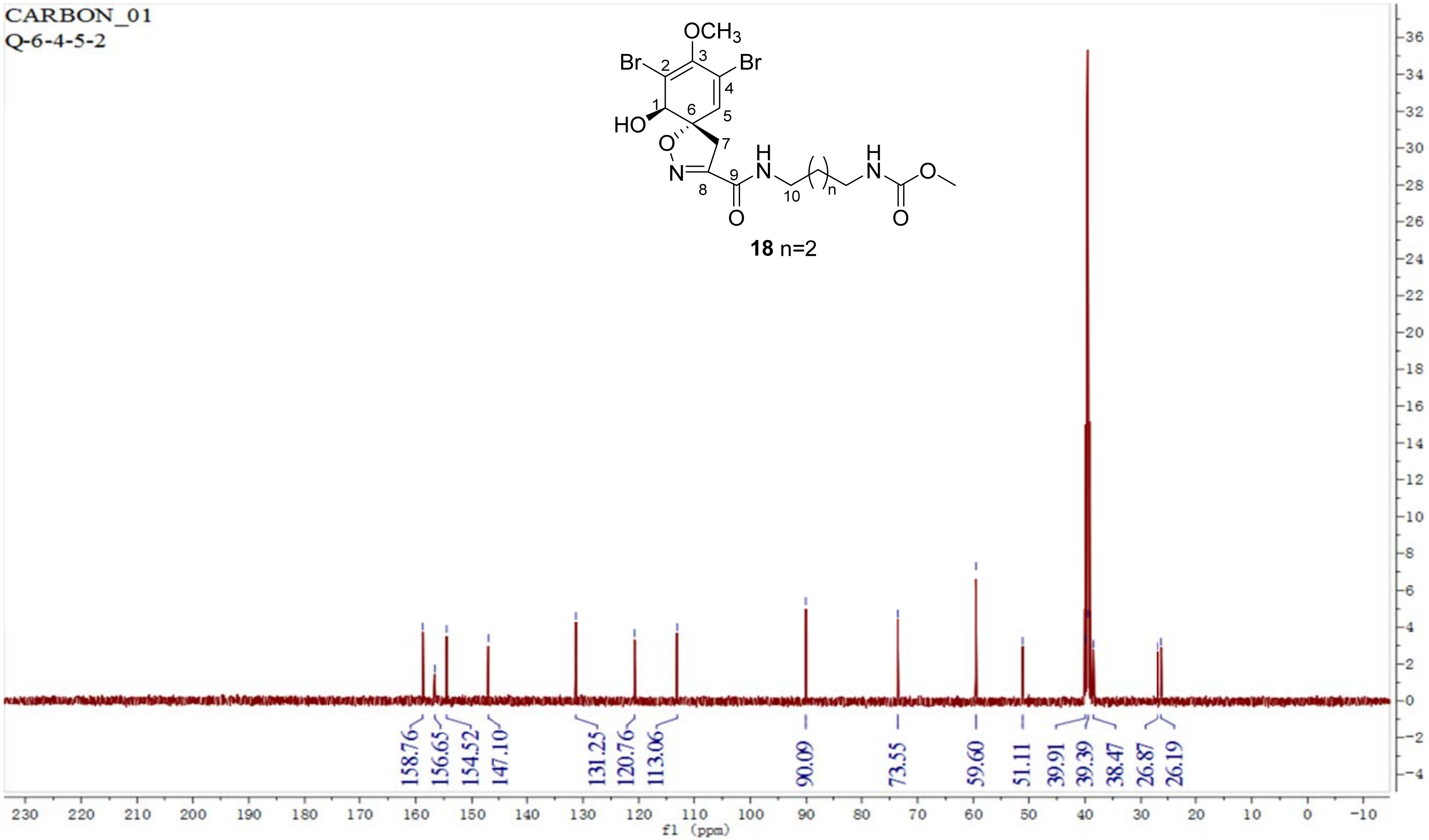

Figure S50. ^{13}C NMR (125 MHz, DMSO-d_6) spectrum of **18**.

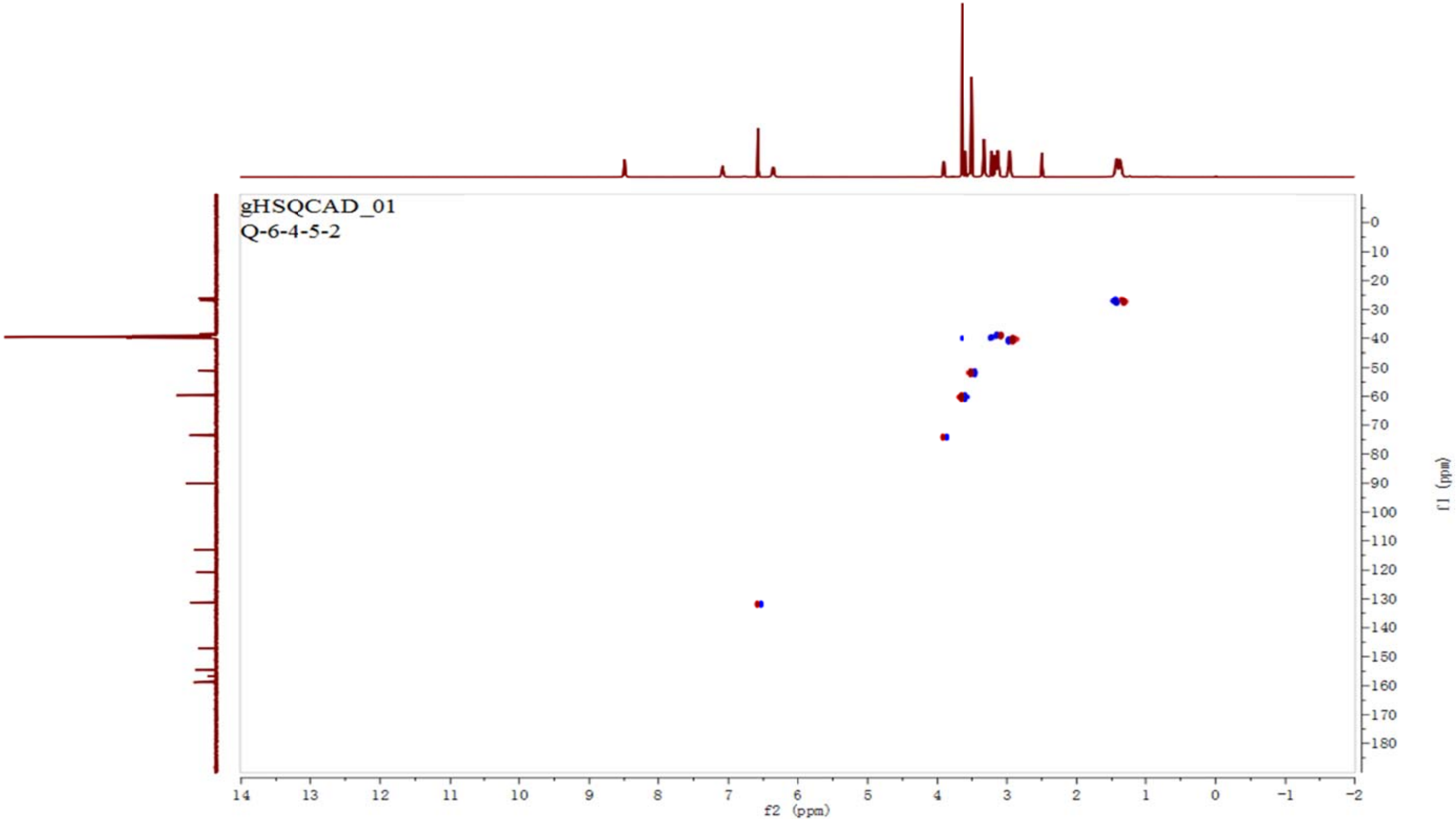

Figure S51. HSQC spectrum of Compound **18**.

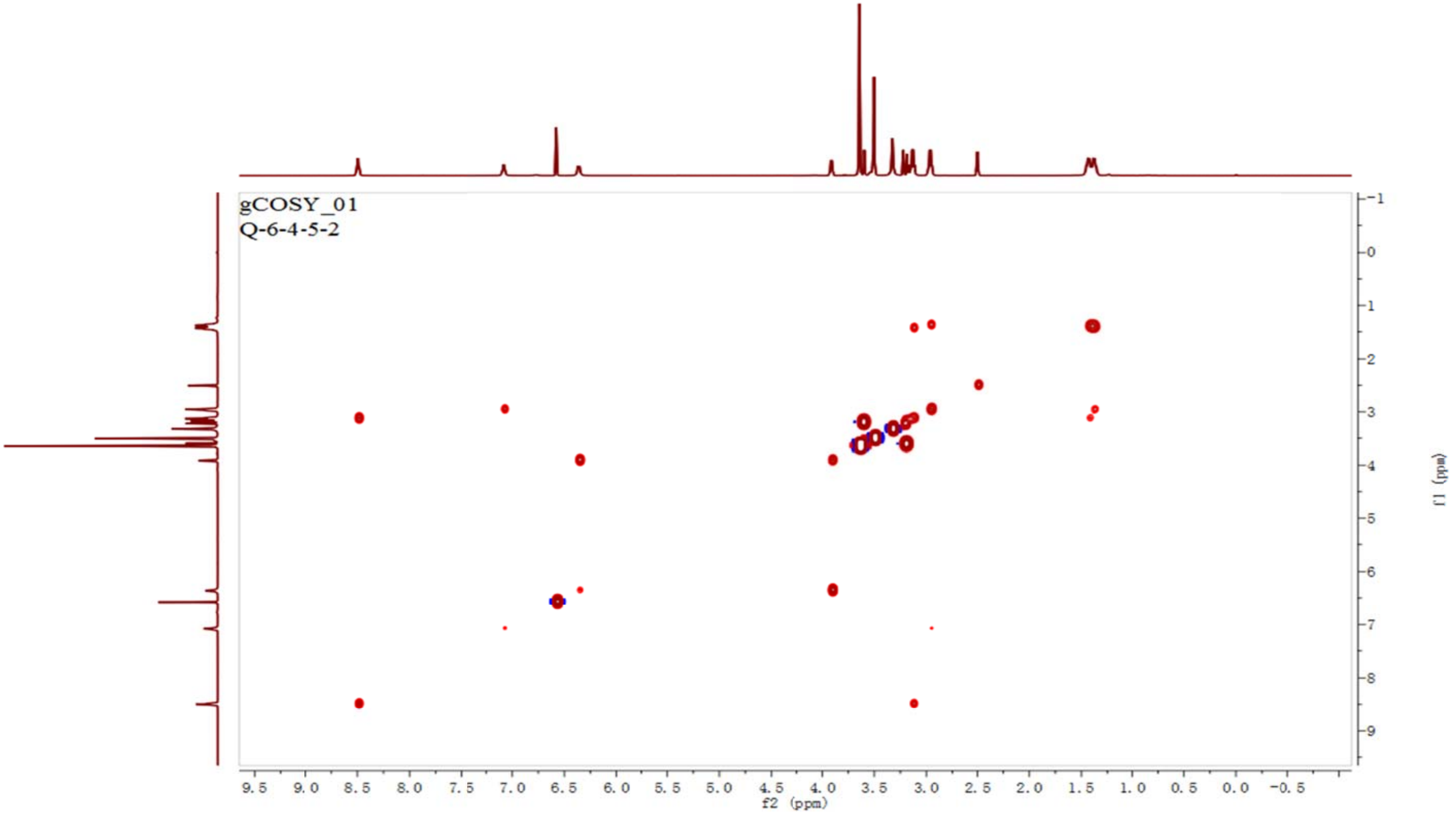

Figure S52. ^{1}H-^{1}H COSY spectrum of Compound **18**.

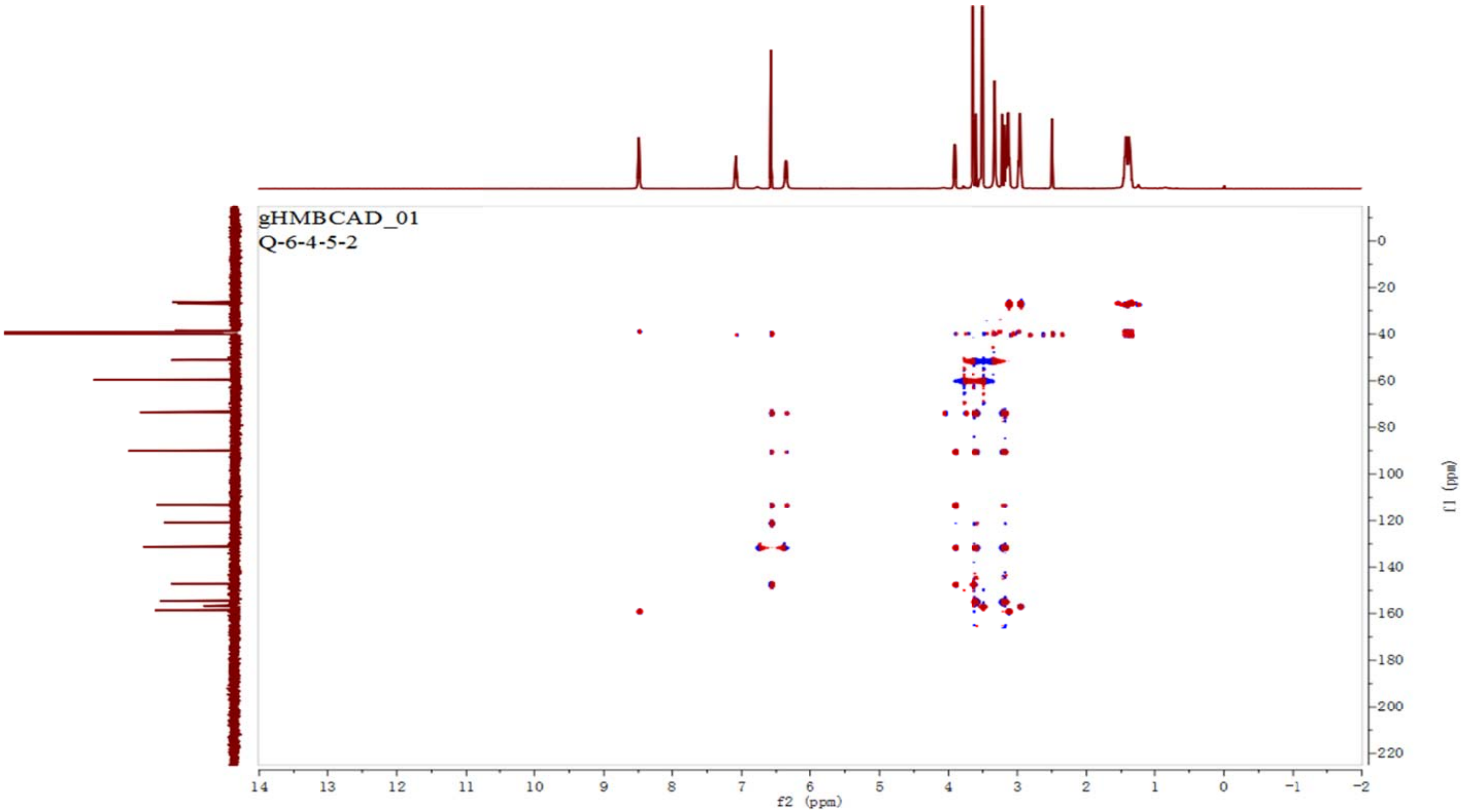

Figure S53. HMBC spectrum of Compound **18**.

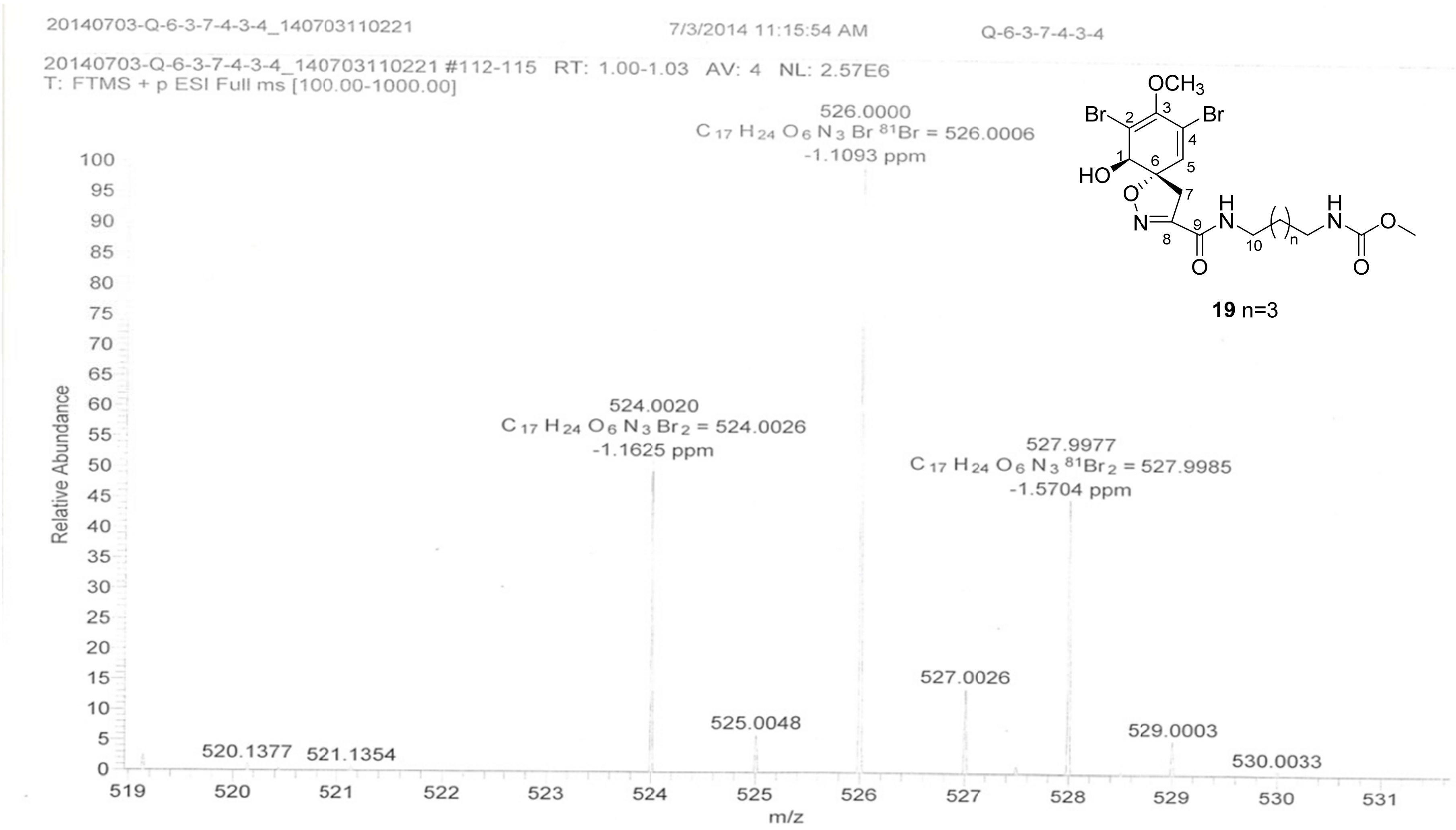

Figure S54. (+)-HRESIMS spectrum of **19**.

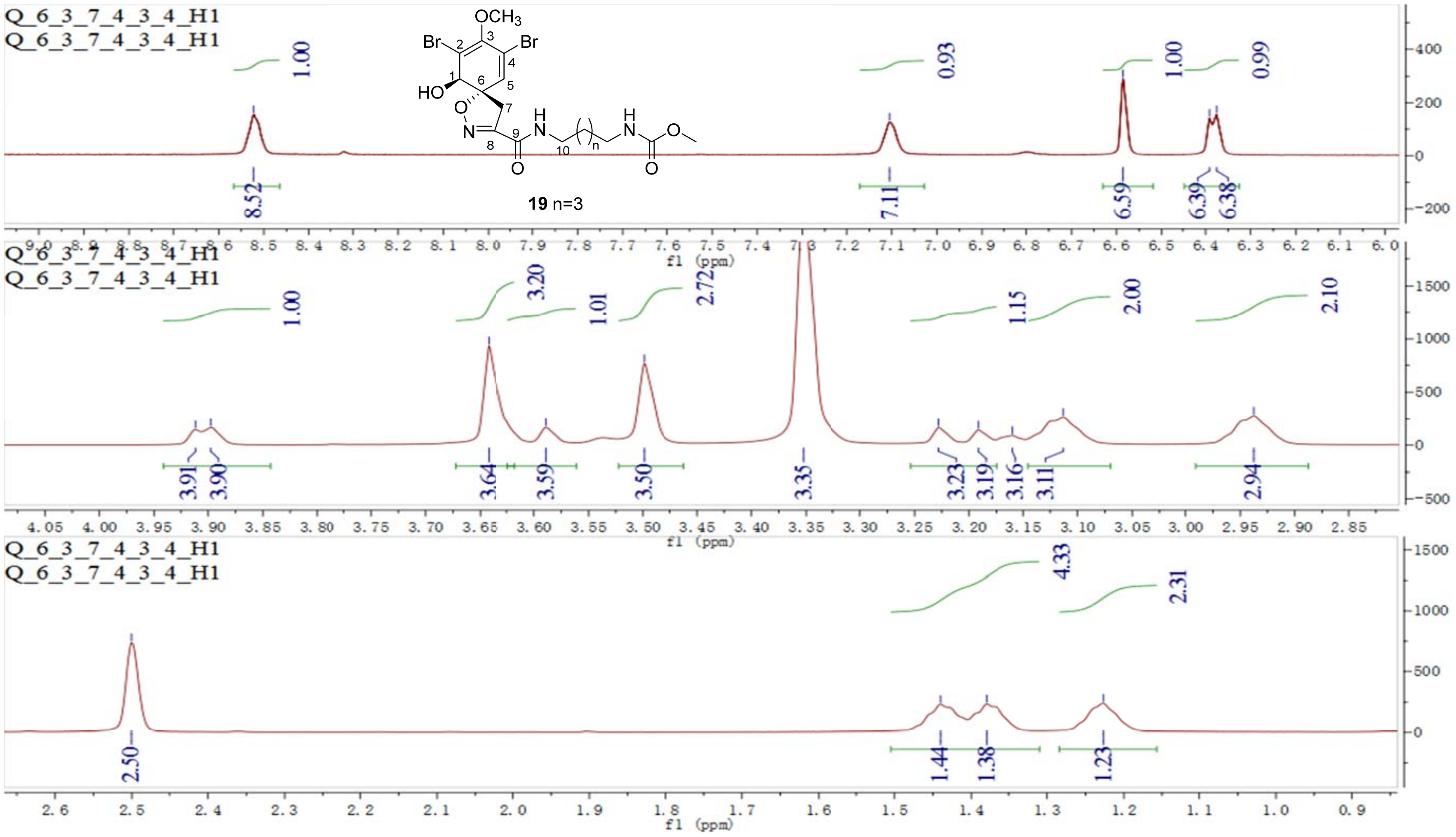

Figure S55. ^{1}H NMR (500 MHz, DMSO-d_6) spectrum of **19**.

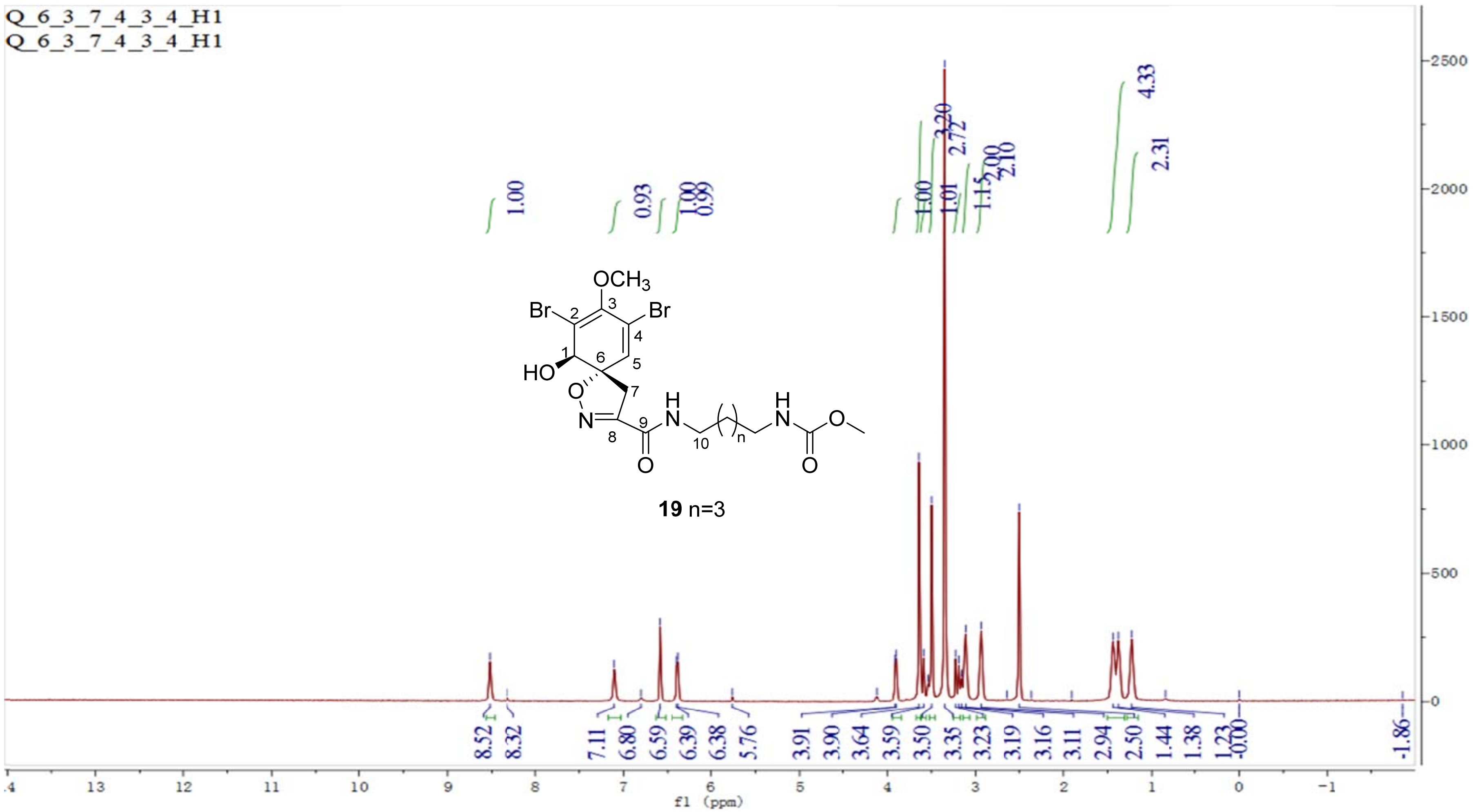

Figure S56. ^{1}H NMR (500 MHz, DMSO-d_6) spectrum of **19**.

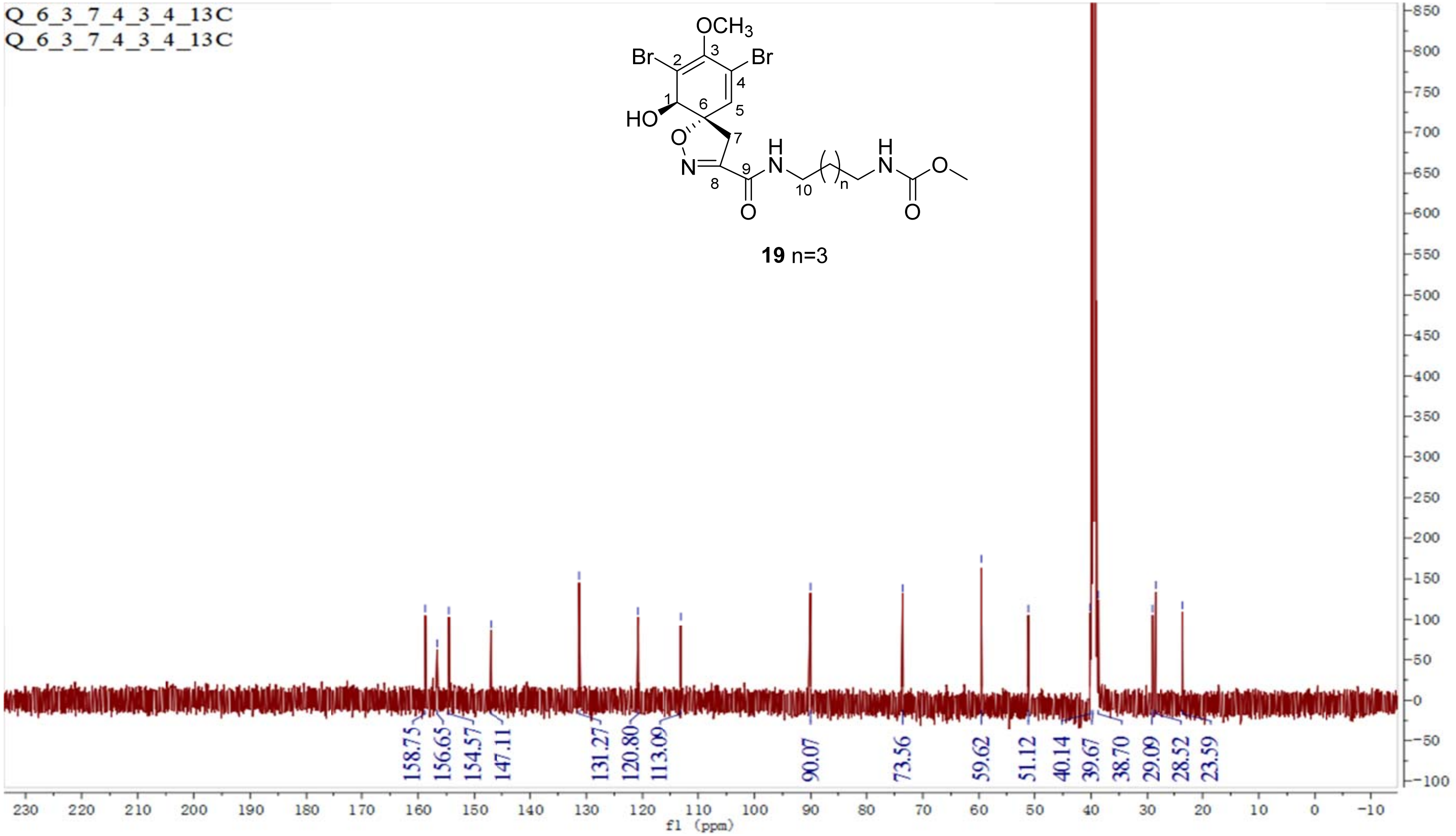

Figure S57. ^{13}C NMR (125 MHz, DMSO-d_6) spectrum of **19**.

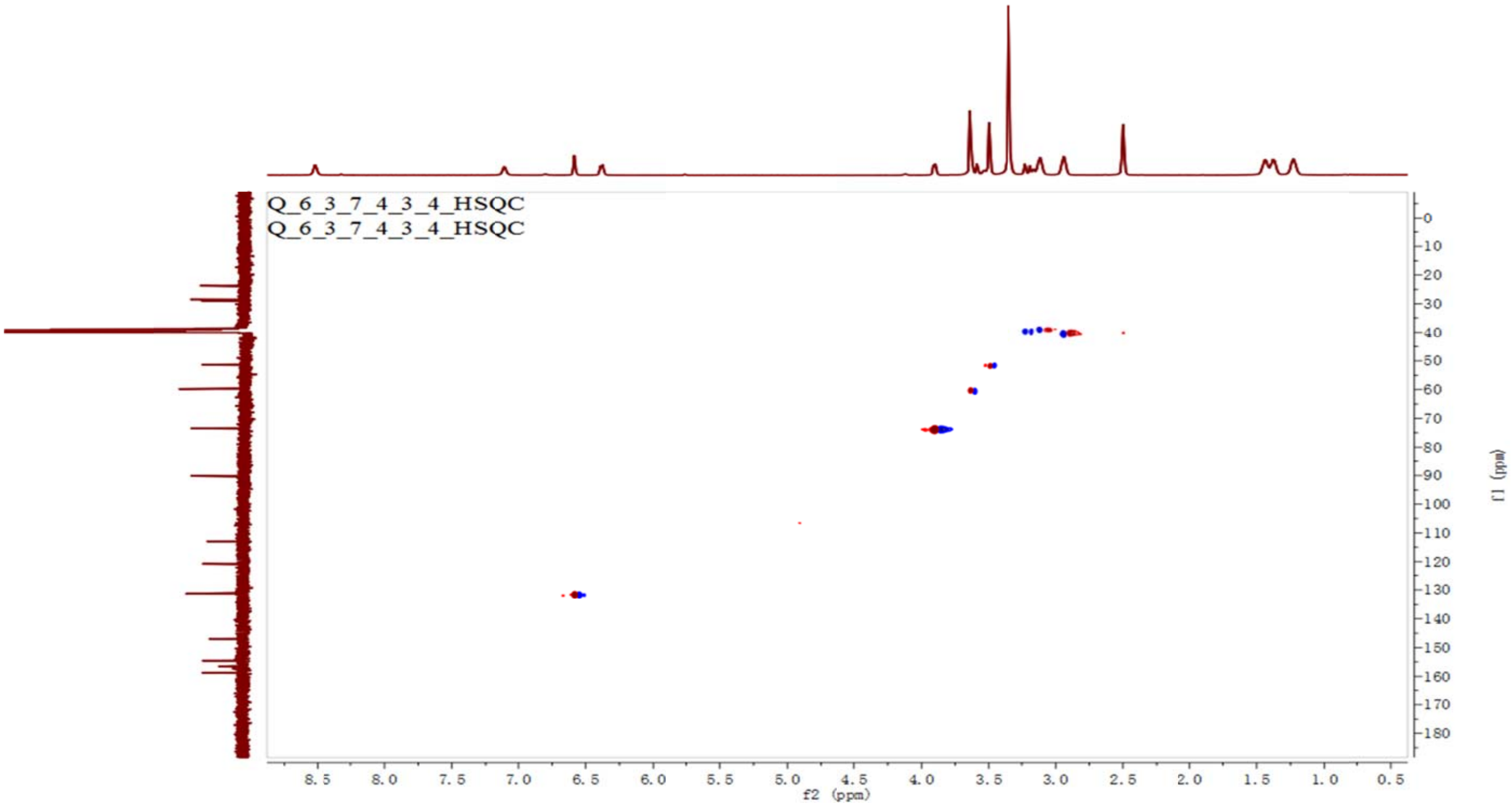

Figure S58. HSQC spectrum of Compound **19**.

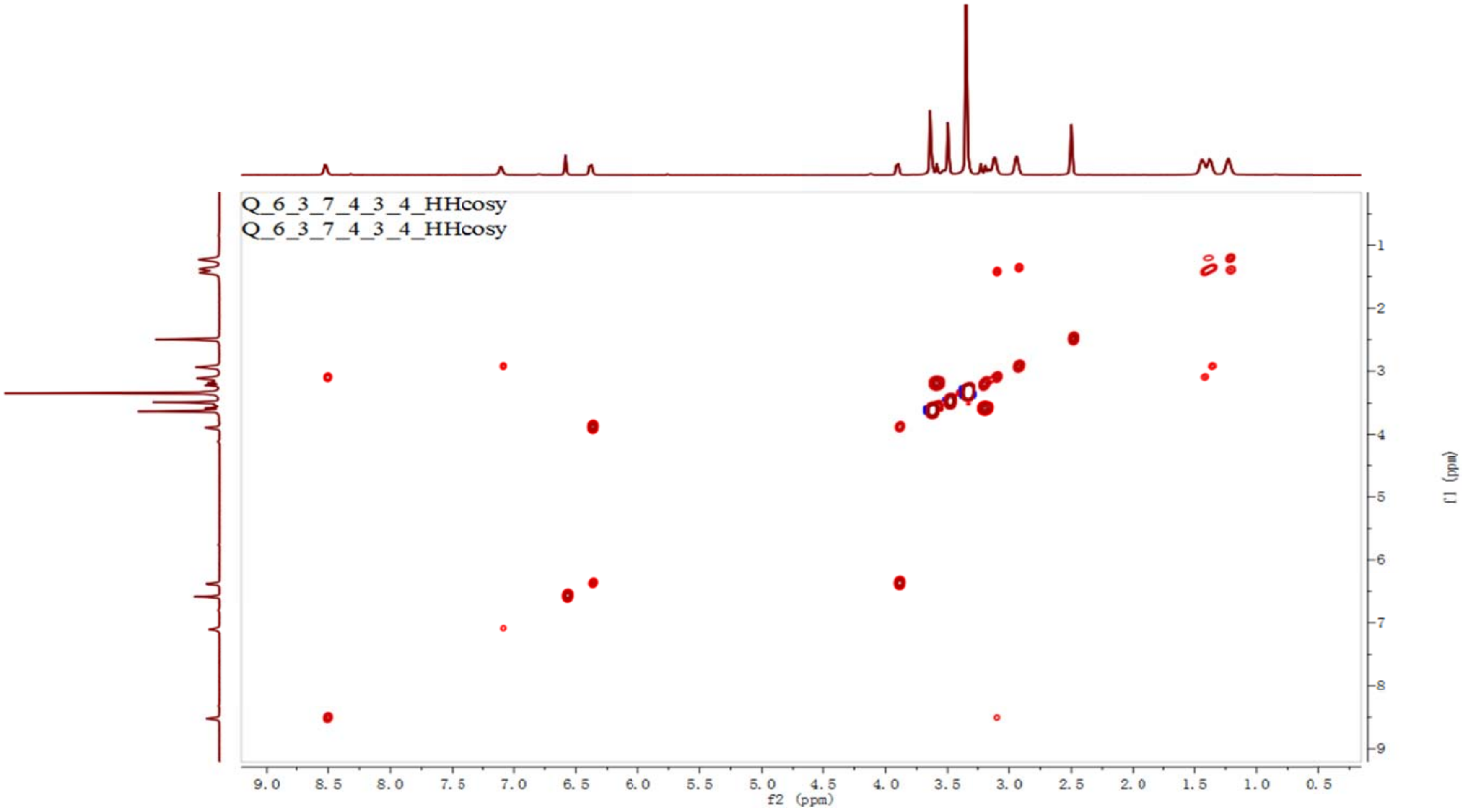

Figure S59. ^{1}H-^{1}H COSY spectrum of Compound **19**.

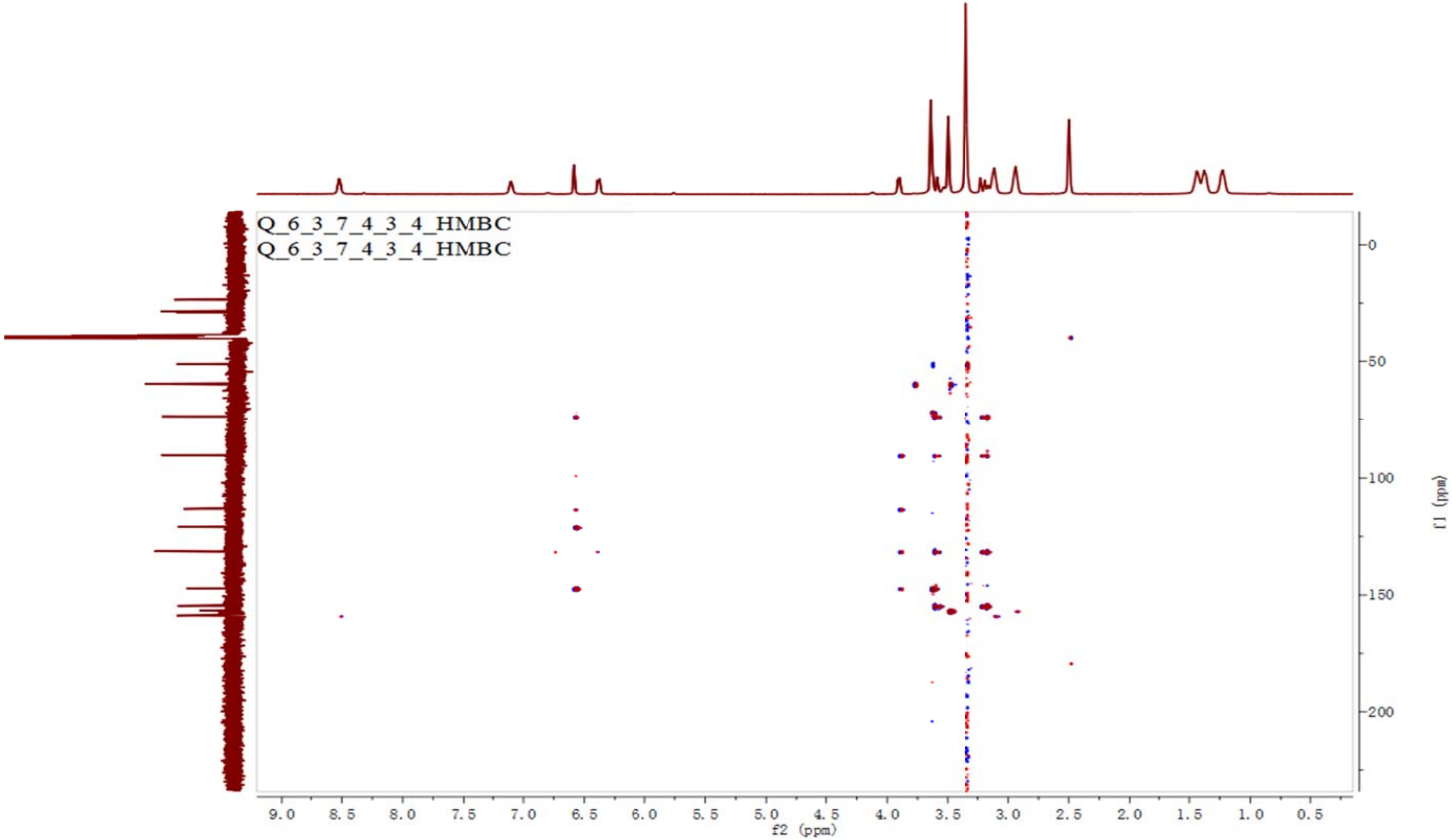

Figure S60. HMBC spectrum of Compound **19**.

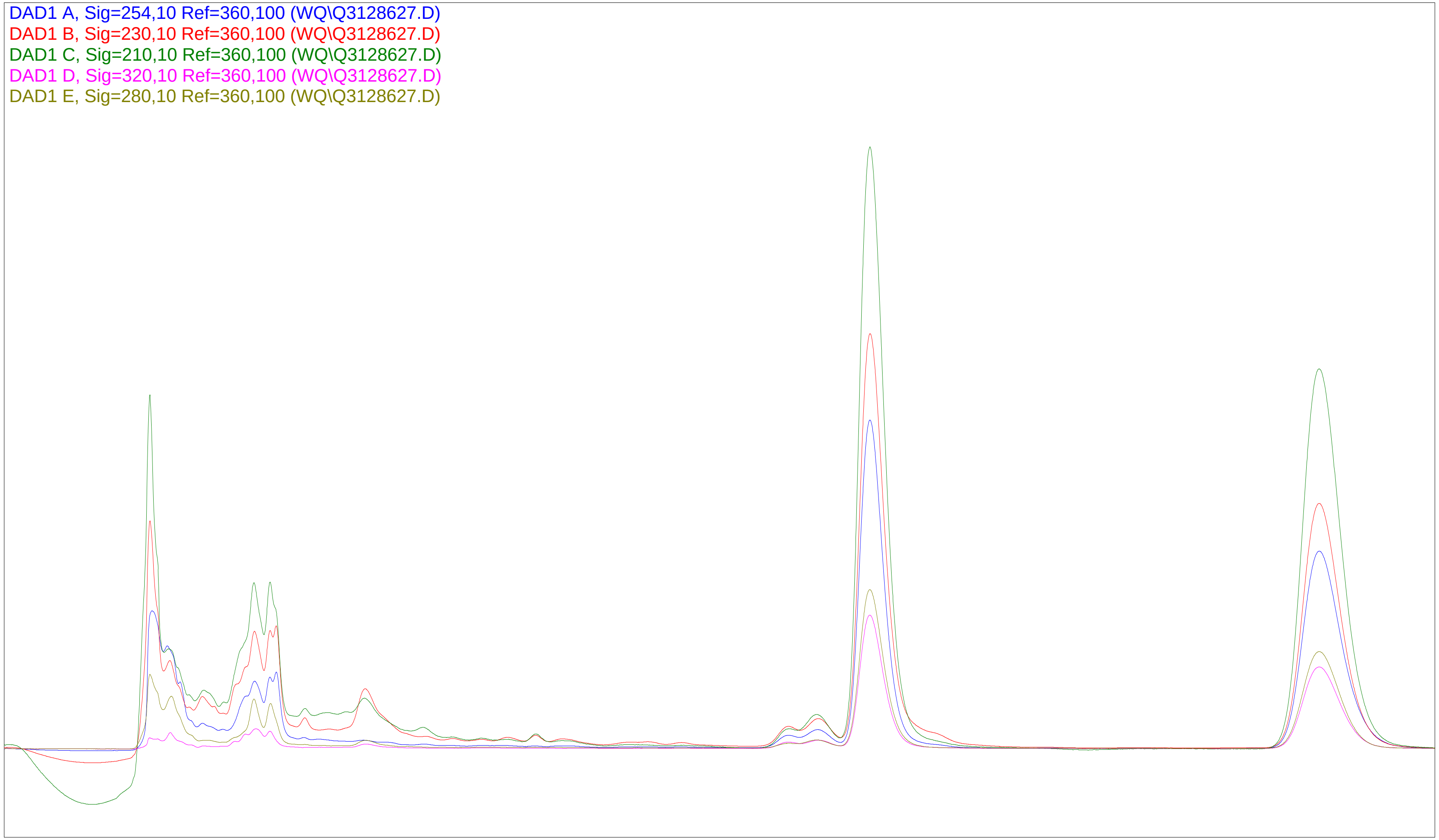

Figure S61. Chiral HPLC of Compound **1**.

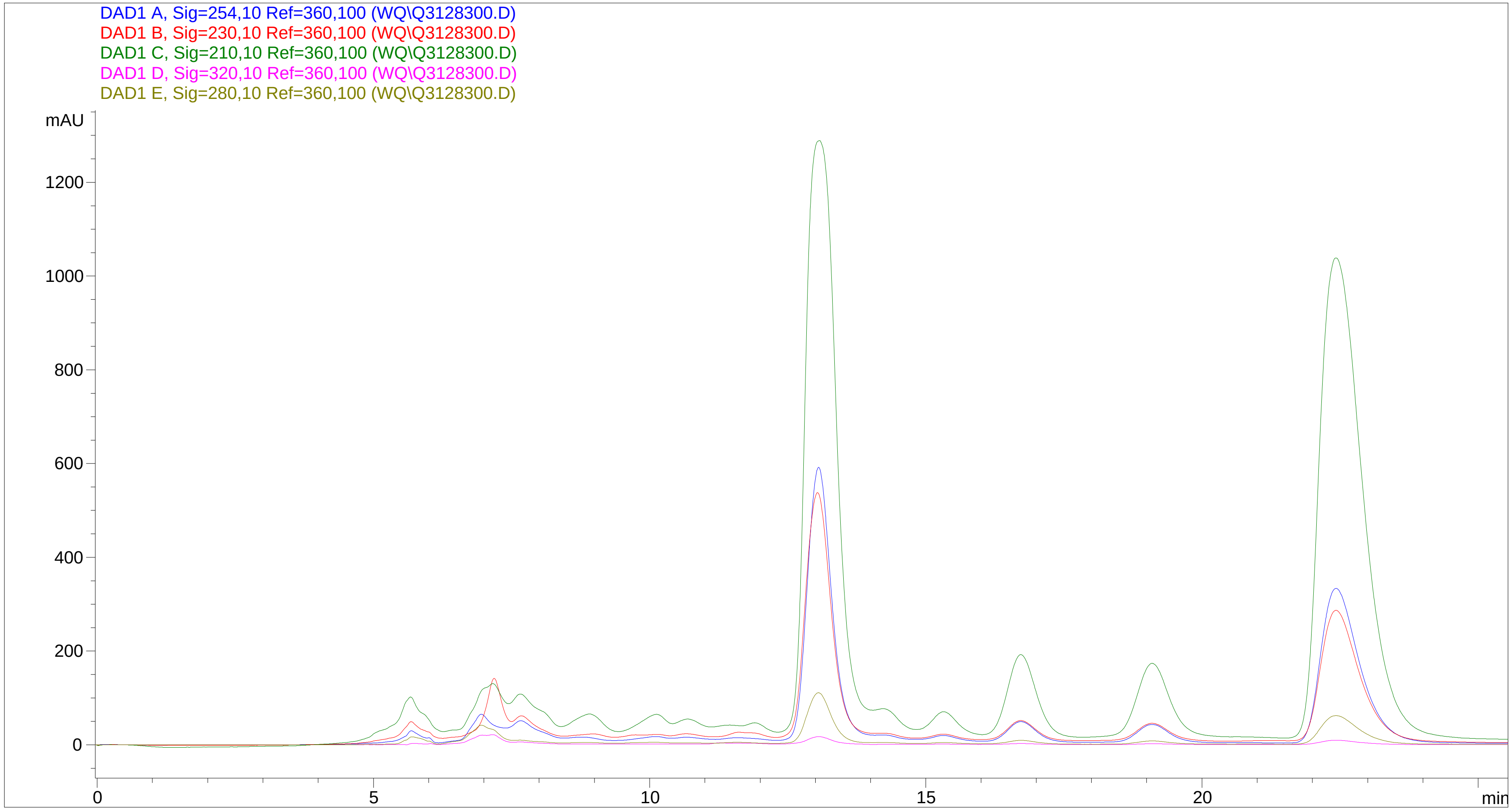

Figure S62. Chiral HPLC of Compound **2**.

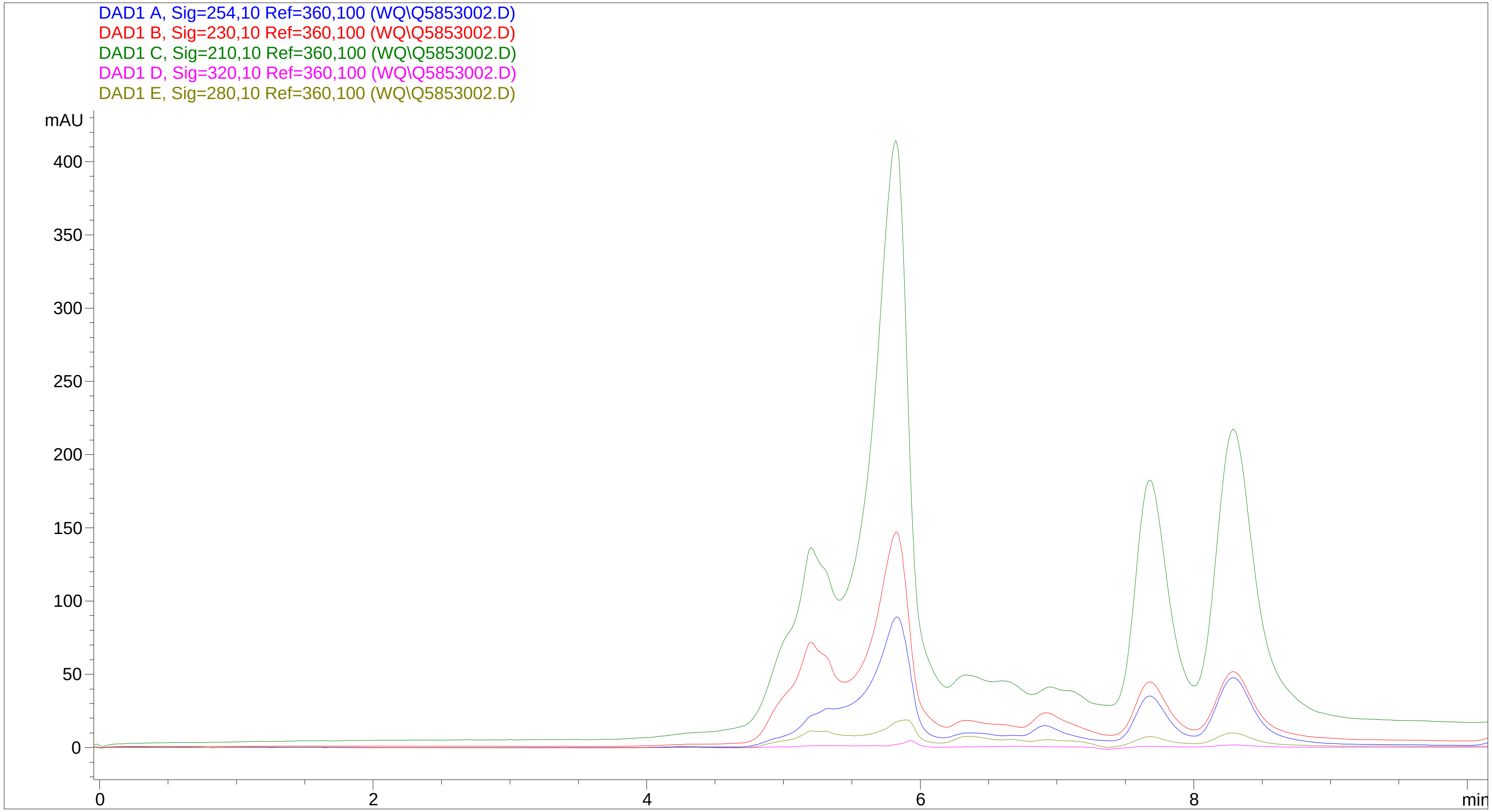

Figure S63. Chiral HPLC of Compound **3**.

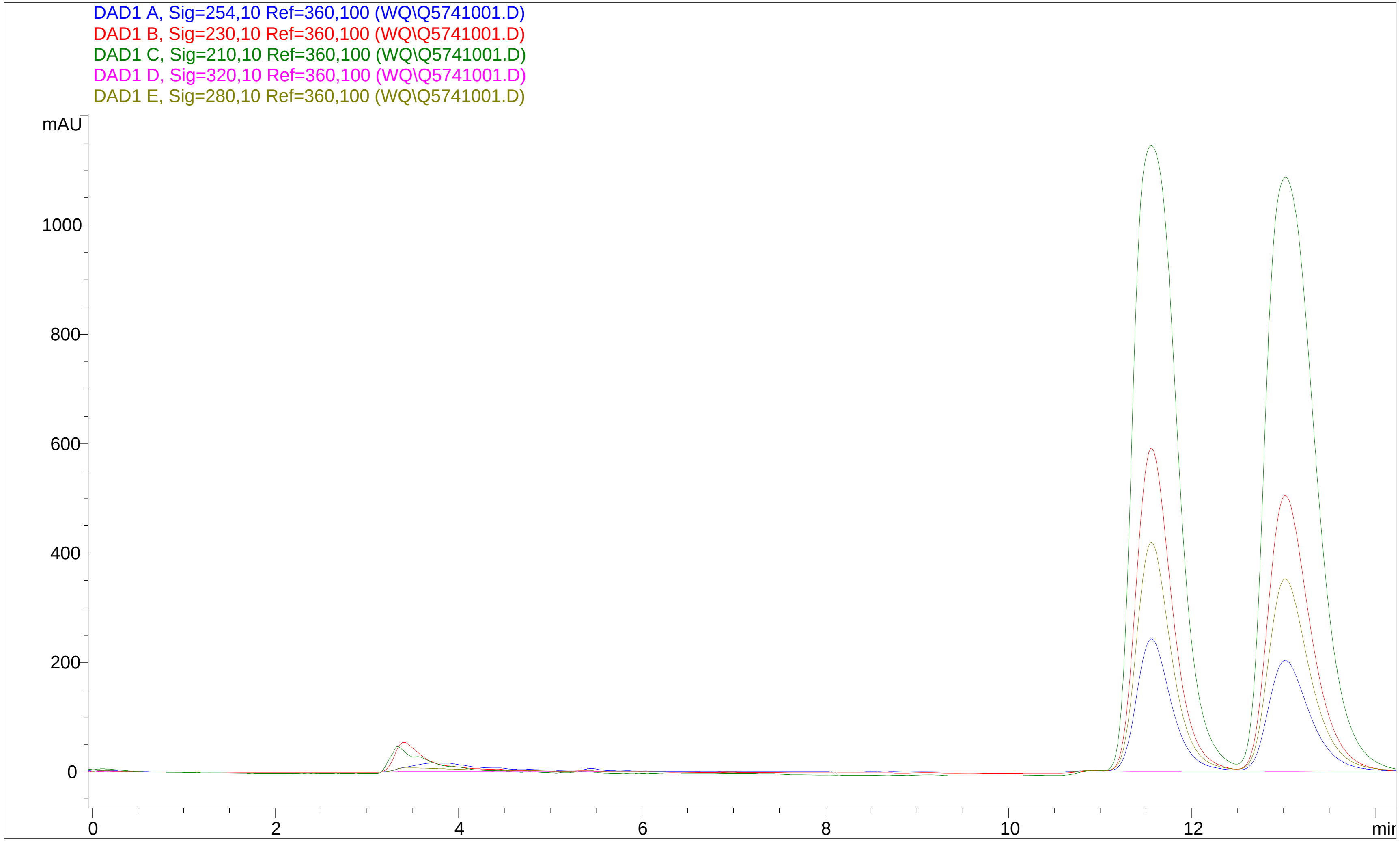

Figure S64. Chiral HPLC of Compound **4**.

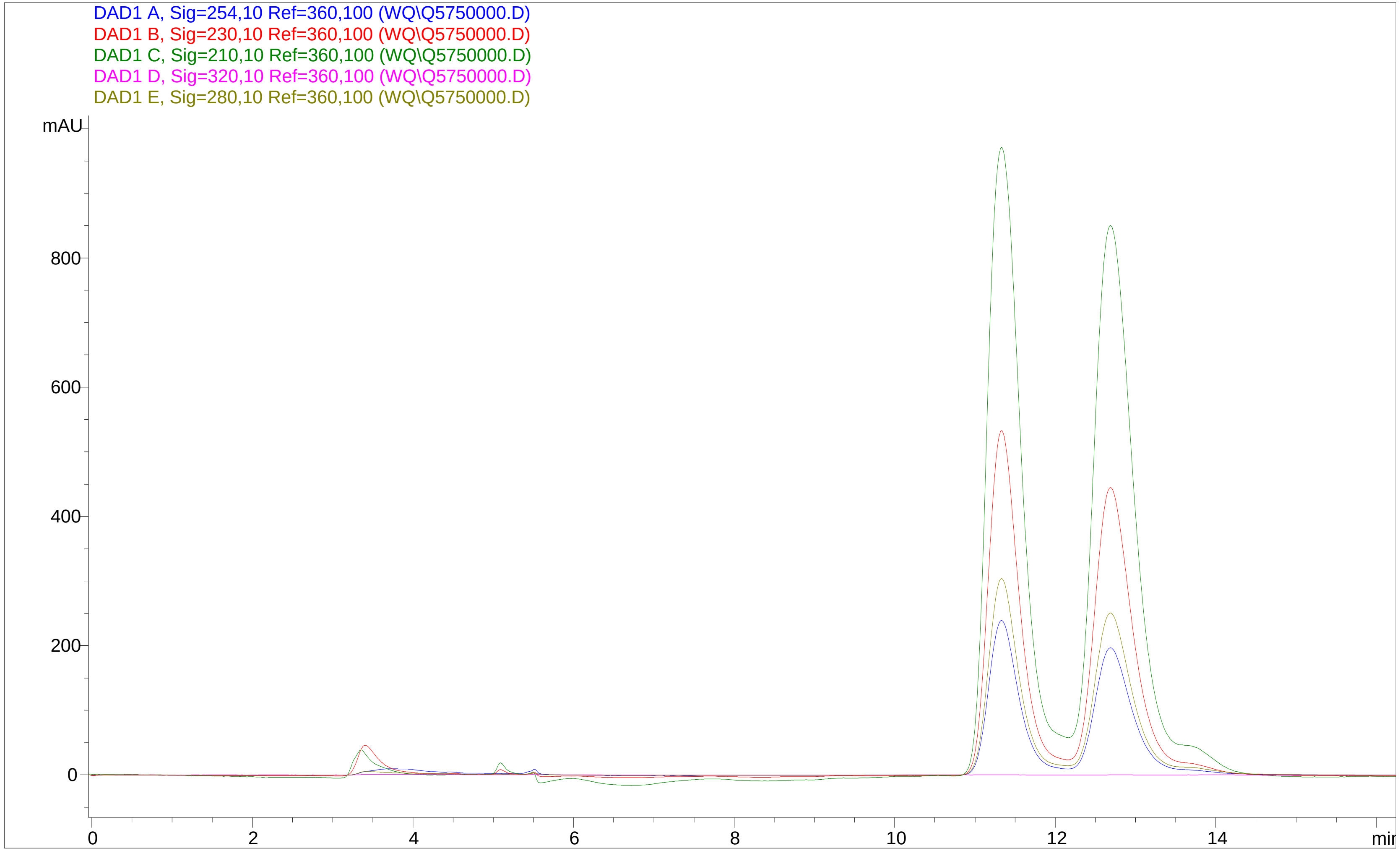

Figure S65. Chiral HPLC of Compound **5**.

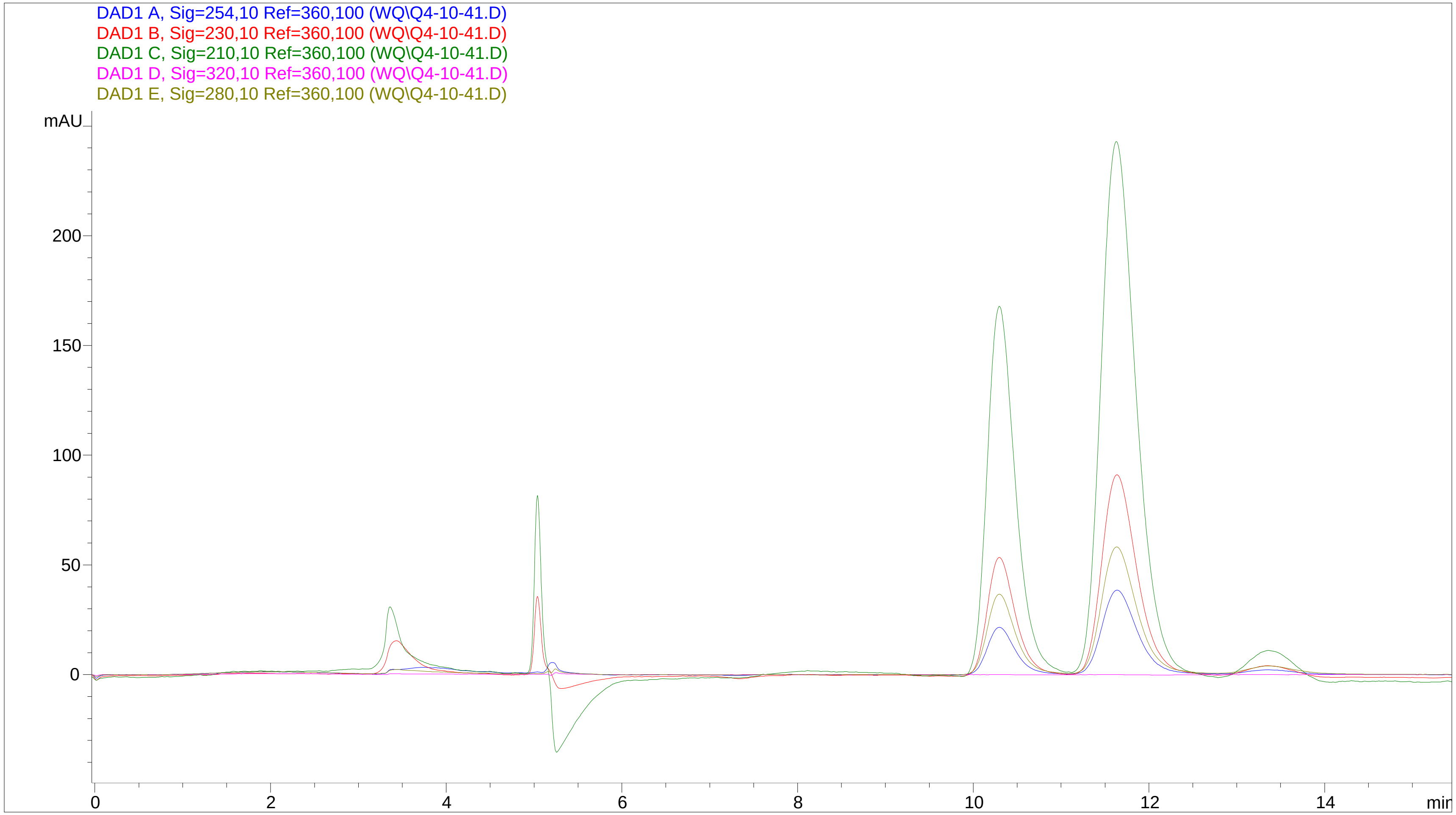

Figure S66. Chiral HPLC of Compound **6**.

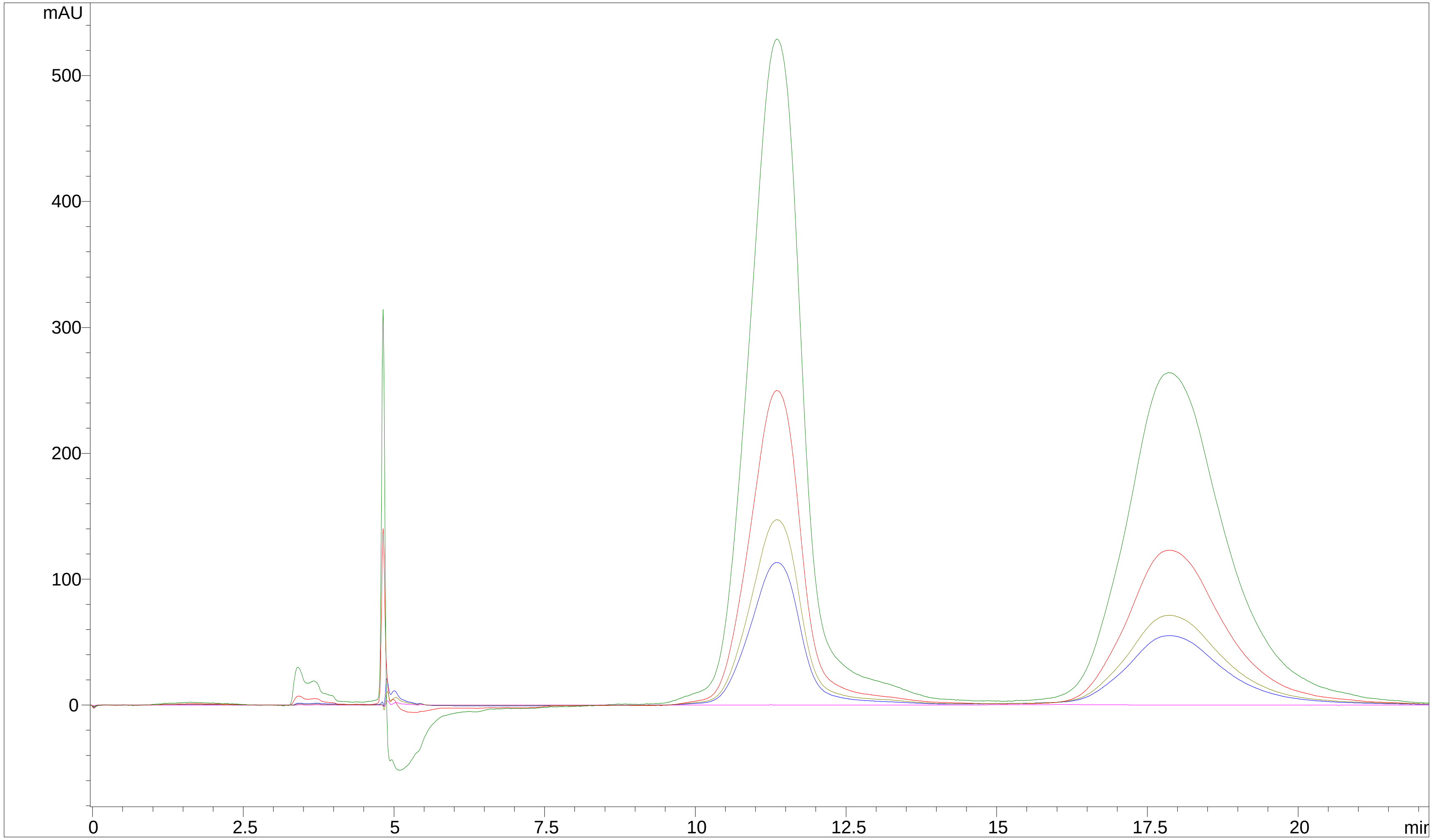

Figure S67. Chiral HPLC of Compound **7**.

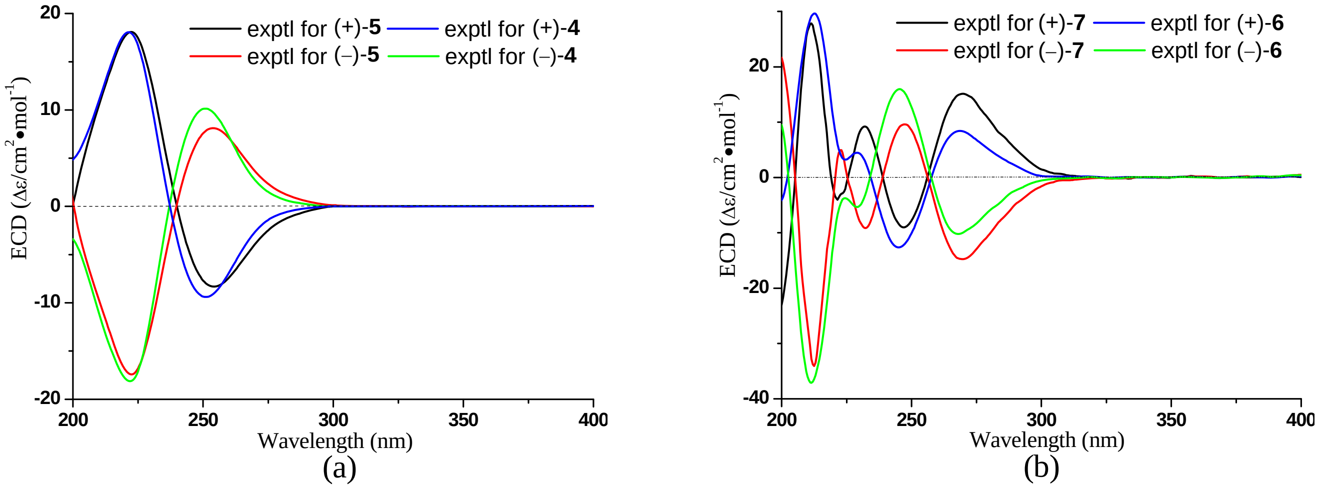

Figure S68. (a) Experimental ECD spectra of (+)- and (-)-**4** in MeOH and experimental ECD spectra of (+)- and (-)-**5** in MeOH. (b) Experimental ECD spectra of (+)- and (-)-**6** in MeOH and experimental ECD spectra of (+)- and (-)-**7** in MeOH.

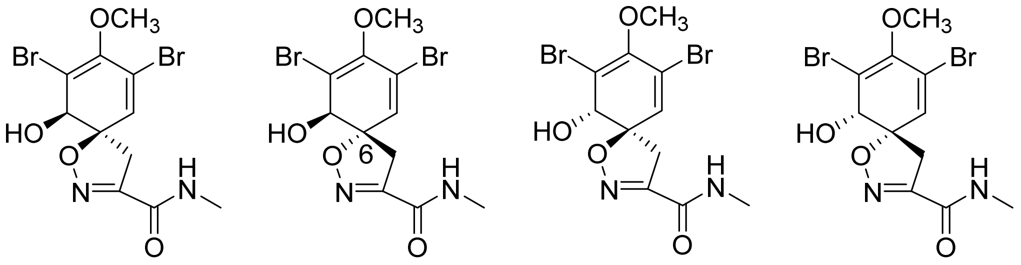

1*R*,6*R* 1*R*,6*S* 1*S*,6*R* 1*S*,6*S*

Figure S69. Calculated fragments of (1R,6R)**-18**, (1R,6S)-18, (1S,6R)-18 and (1S,6S)**-18**.

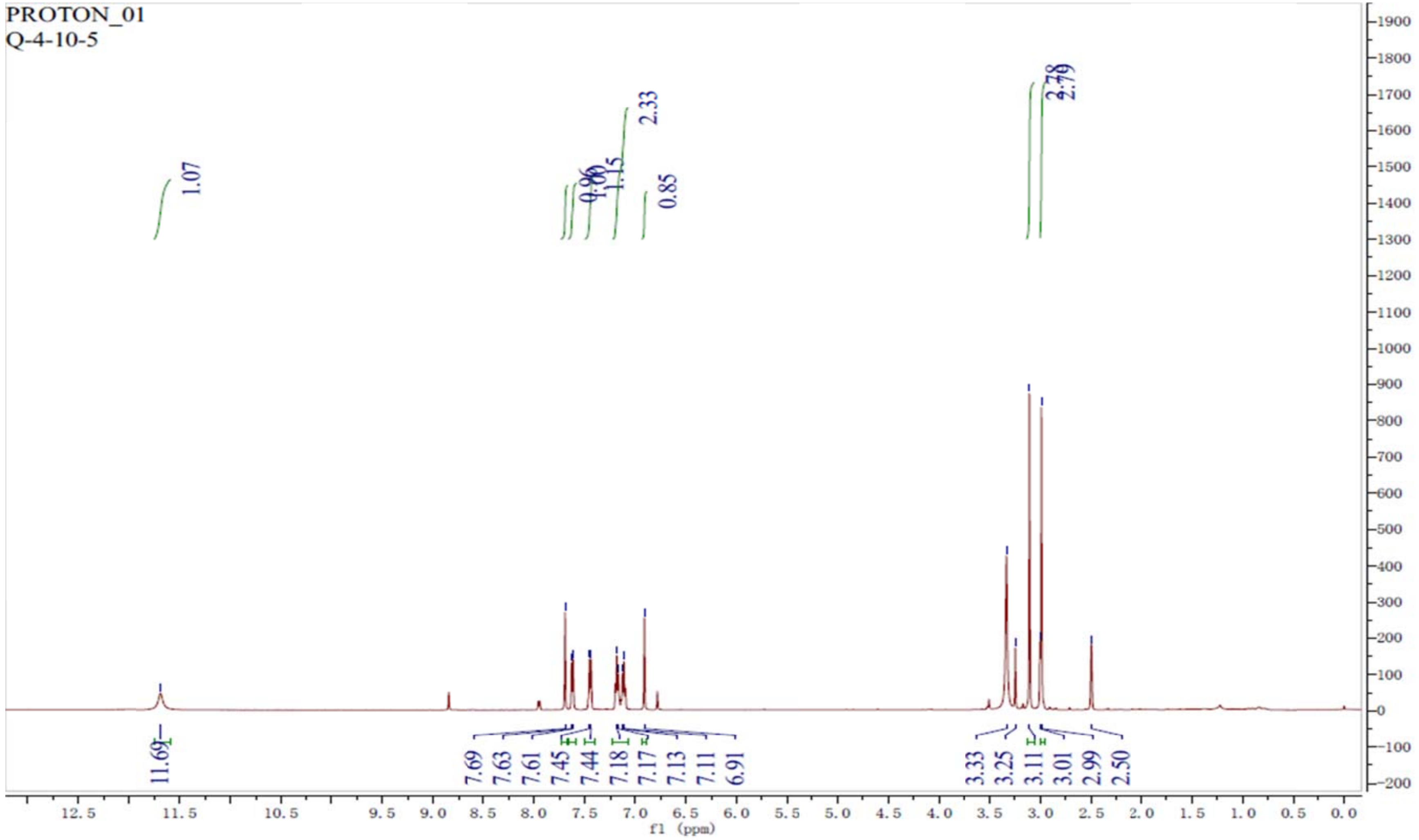

Figure S70. ^{1}H NMR (500 MHz, DMSO-d_6) spectrum of **8**.

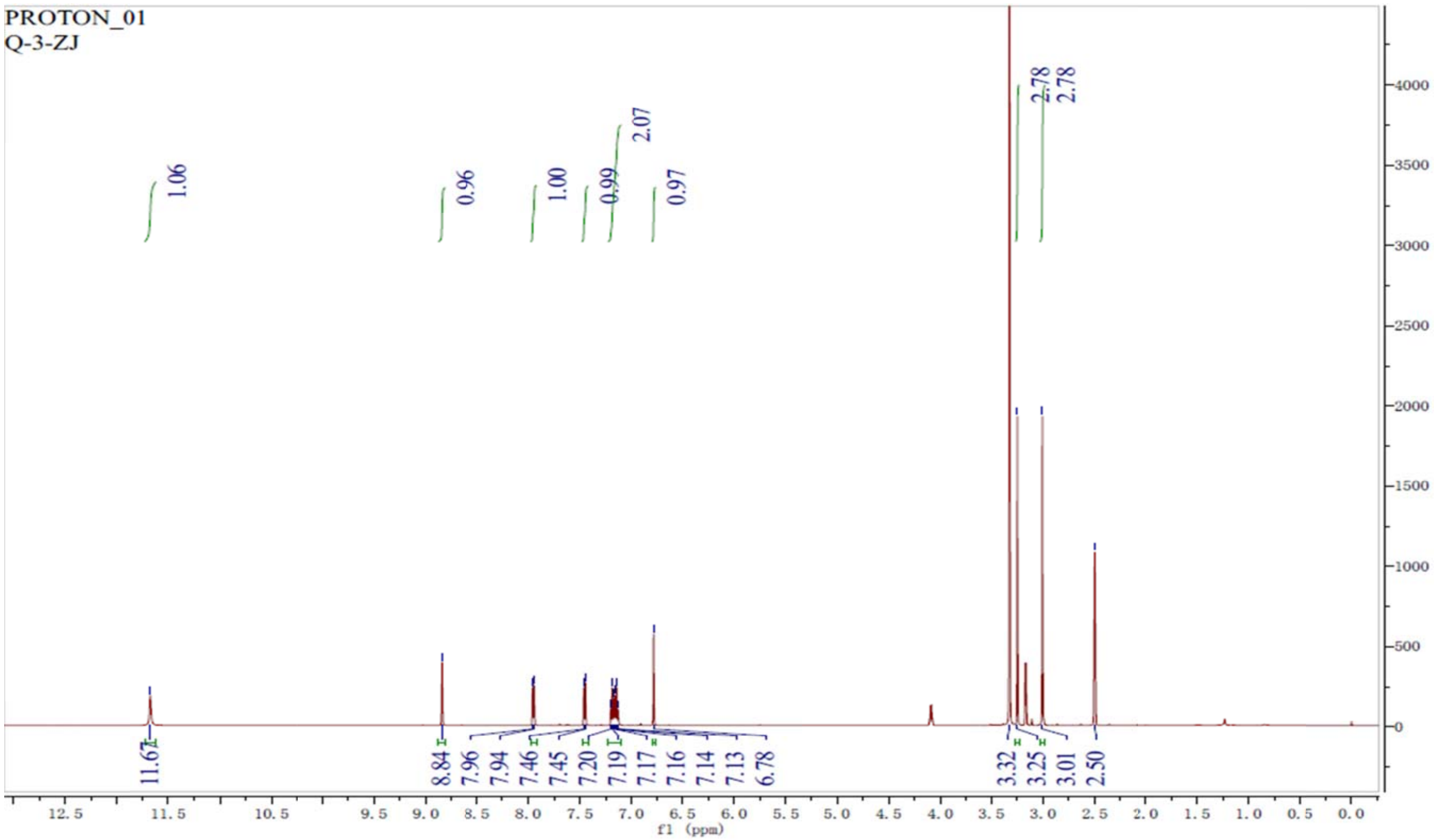

Figure S71. ^{1}H NMR (500 MHz, DMSO-d_6) spectrum of **9**.

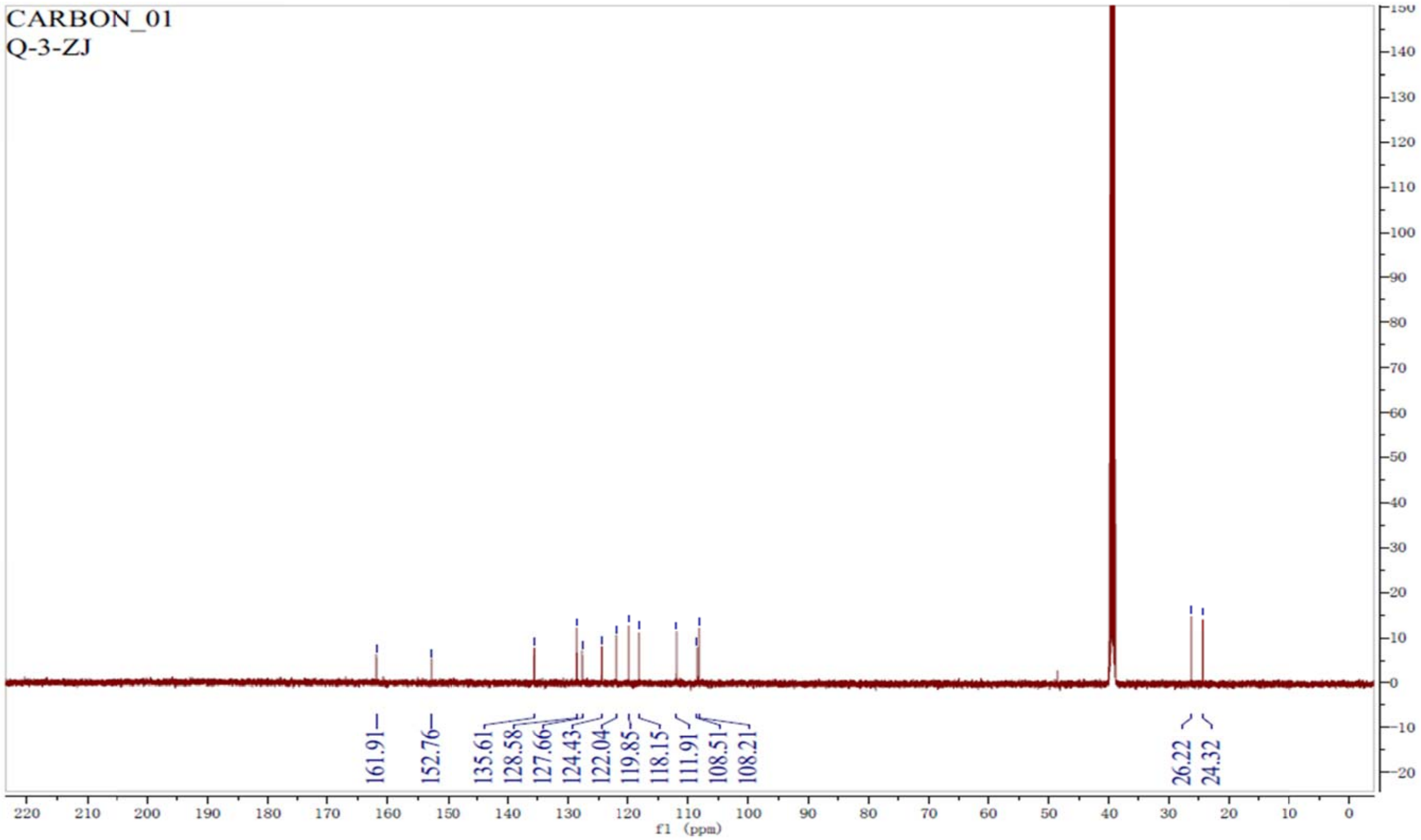

Figure S72. ^{13}C NMR (125 MHz, DMSO-d_6) spectrum of **9**.

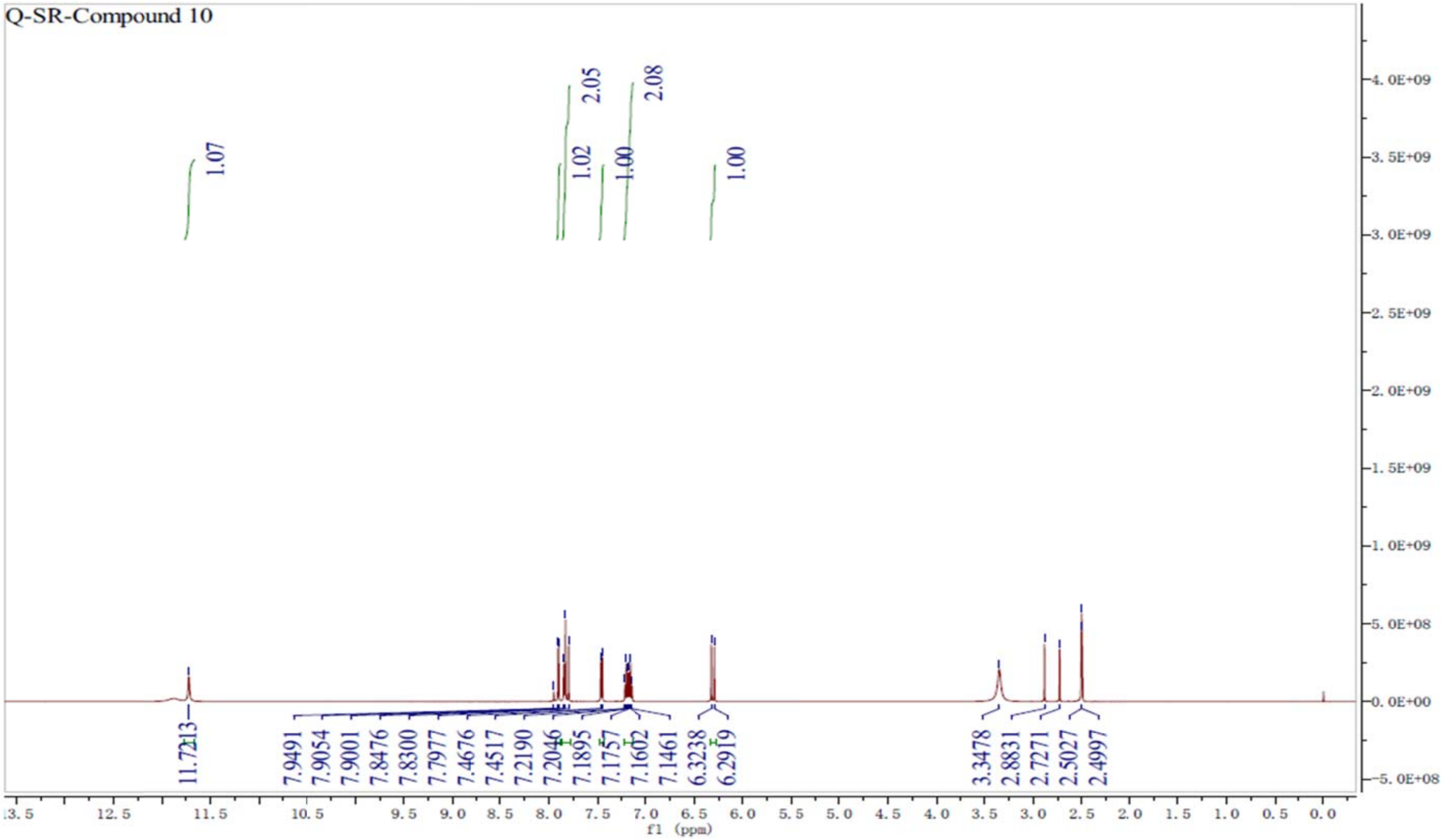

Figure S73. ^{1}H NMR (500 MHz, DMSO-d_6) spectrum of **10**.

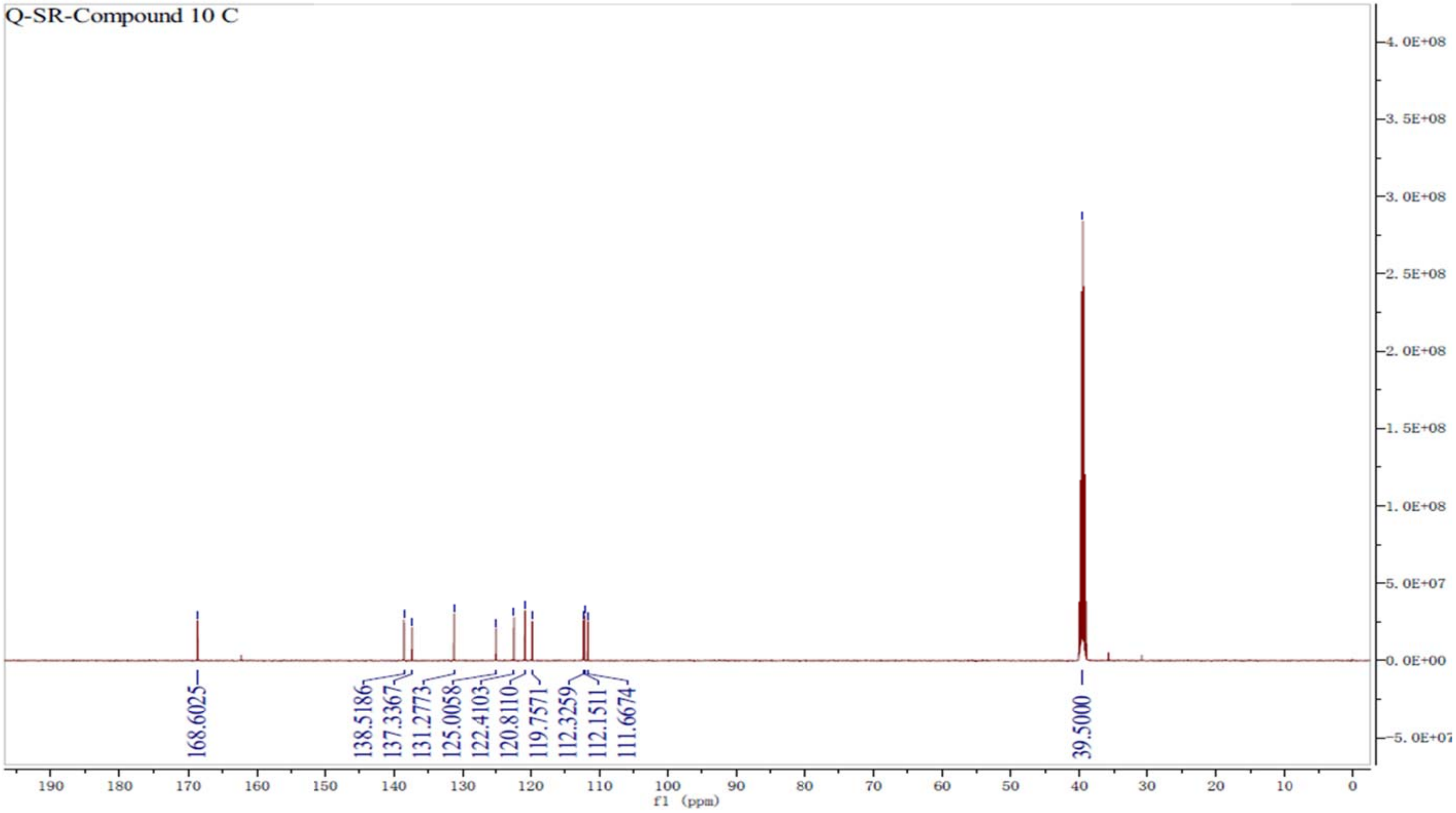

Figure S74. ^{13}C NMR (125 MHz, DMSO-d_6) spectrum of **10**.

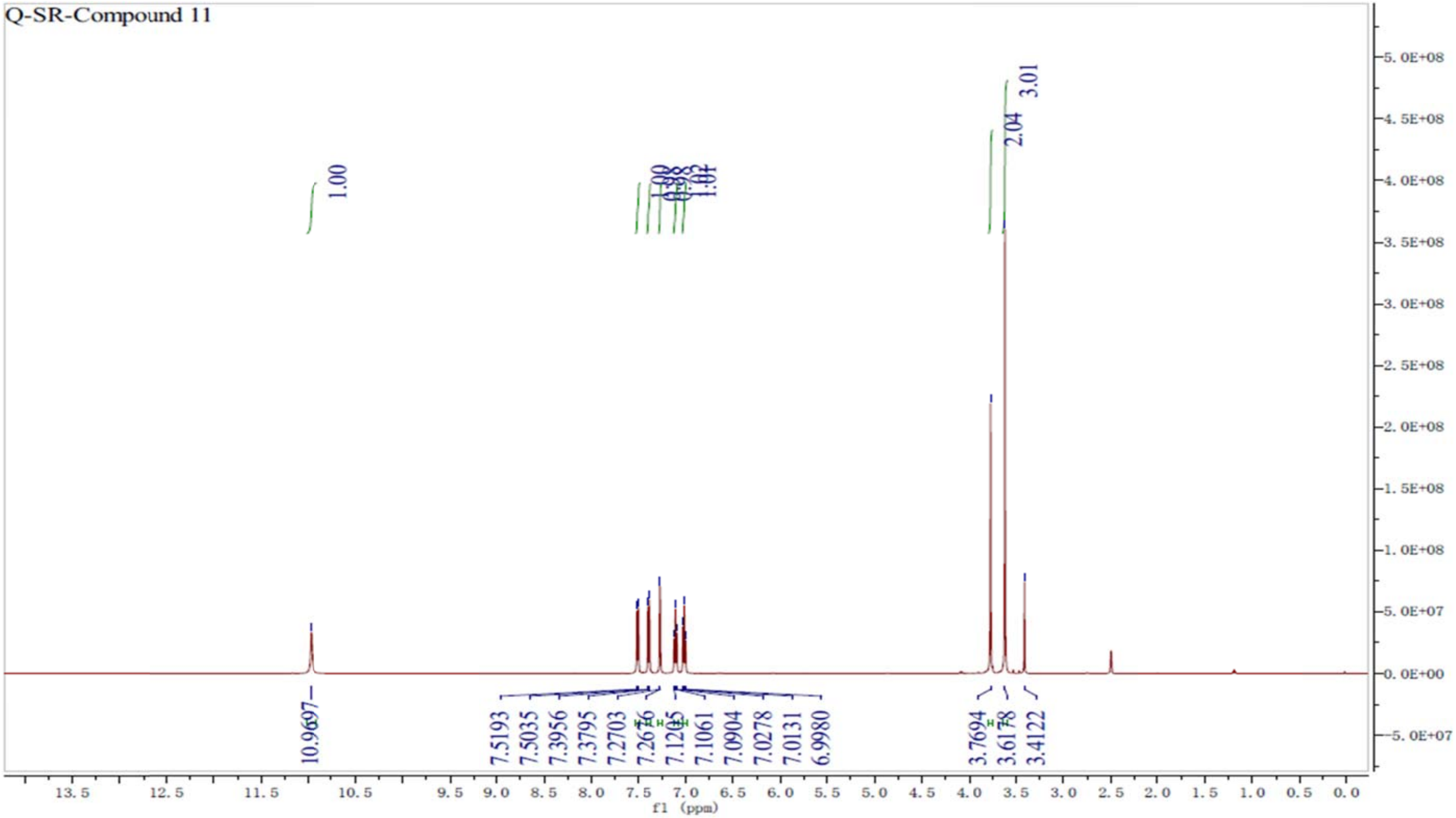

Figure S75. ^{1}H NMR (500 MHz, DMSO-d_6) spectrum of **11**.

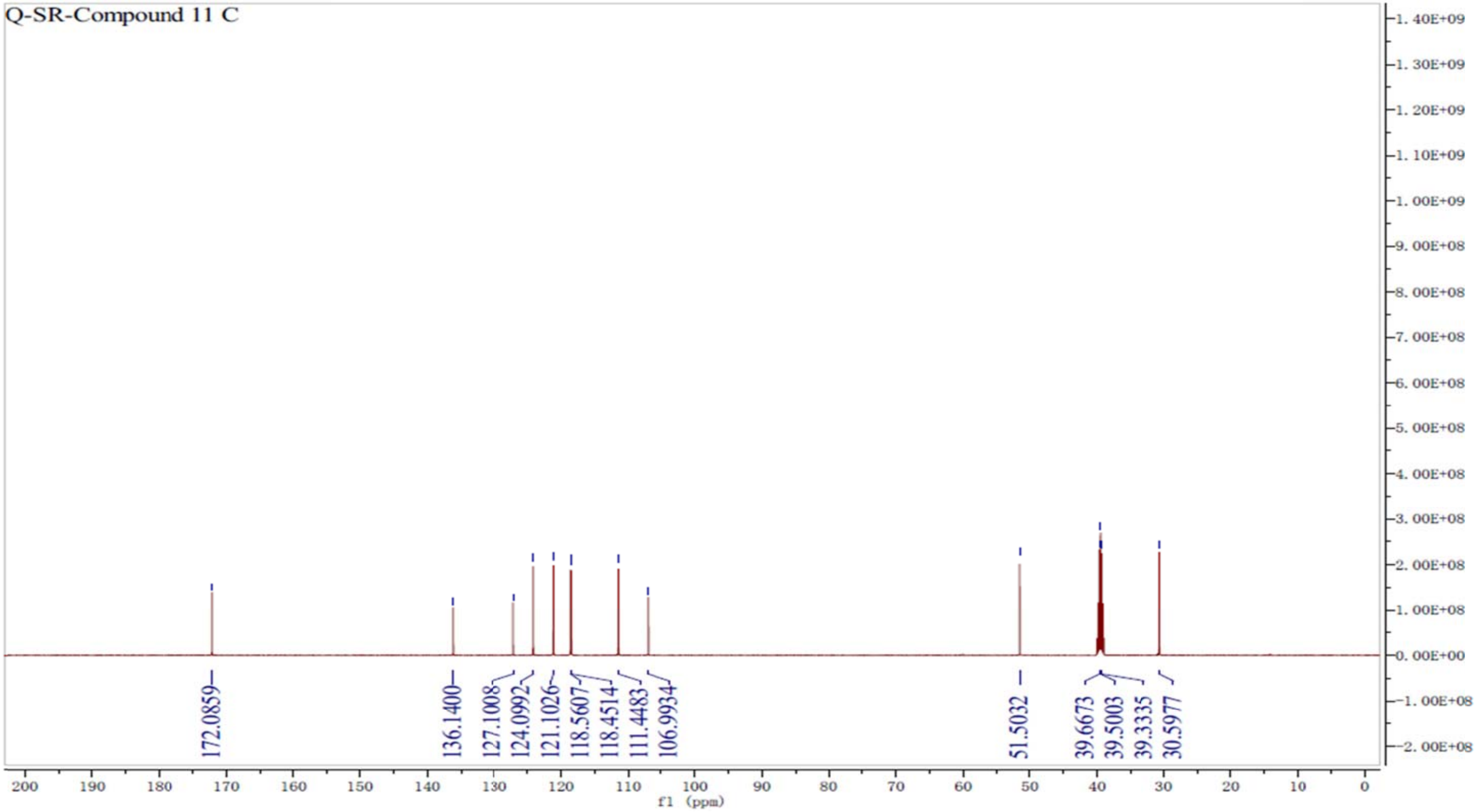

Figure S76. ^{13}C NMR (125 MHz, DMSO-d_6) spectrum of **11**.

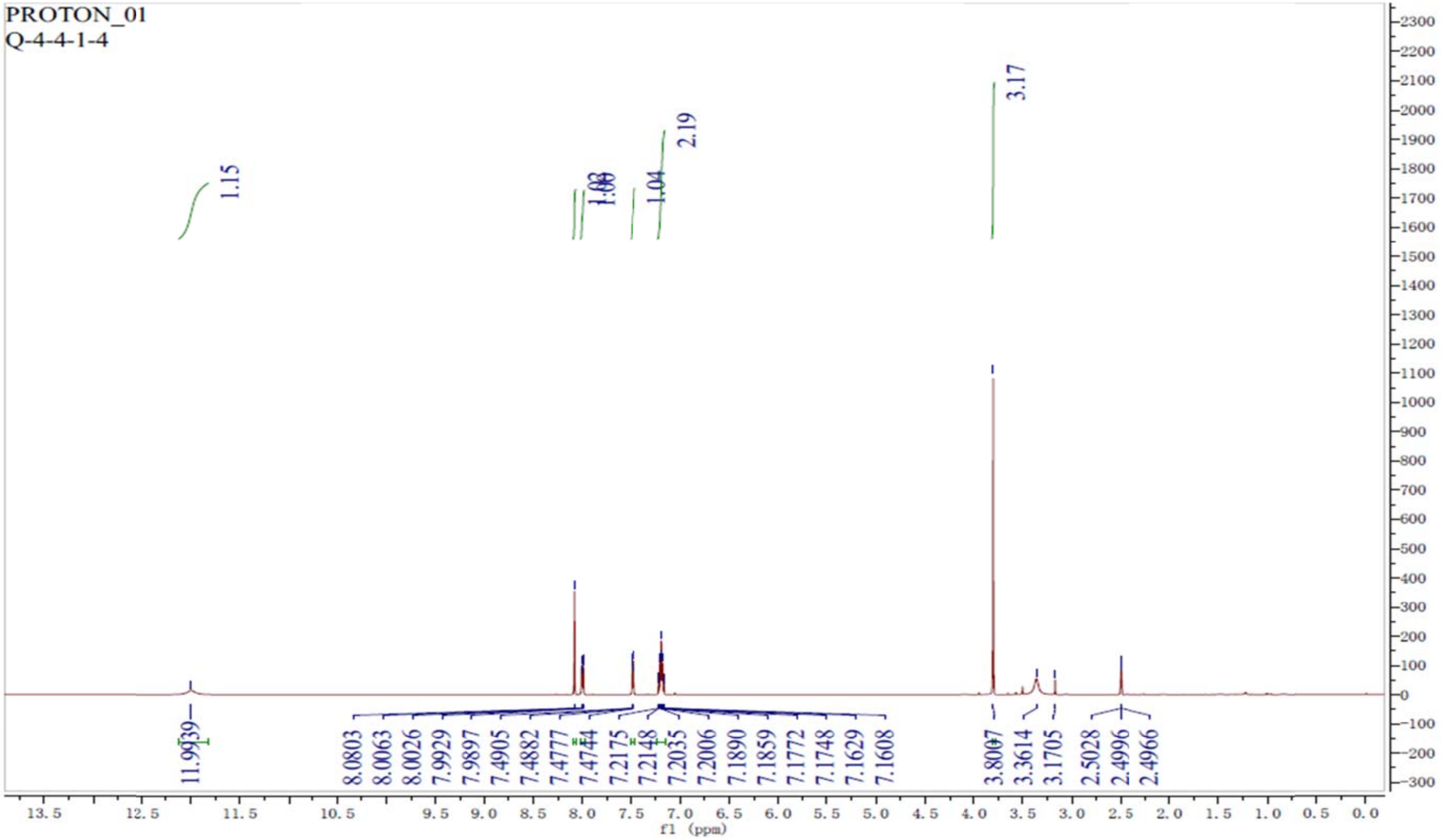

Figure S77. ^{1}H NMR (500 MHz, DMSO-d_6) spectrum of **12**.

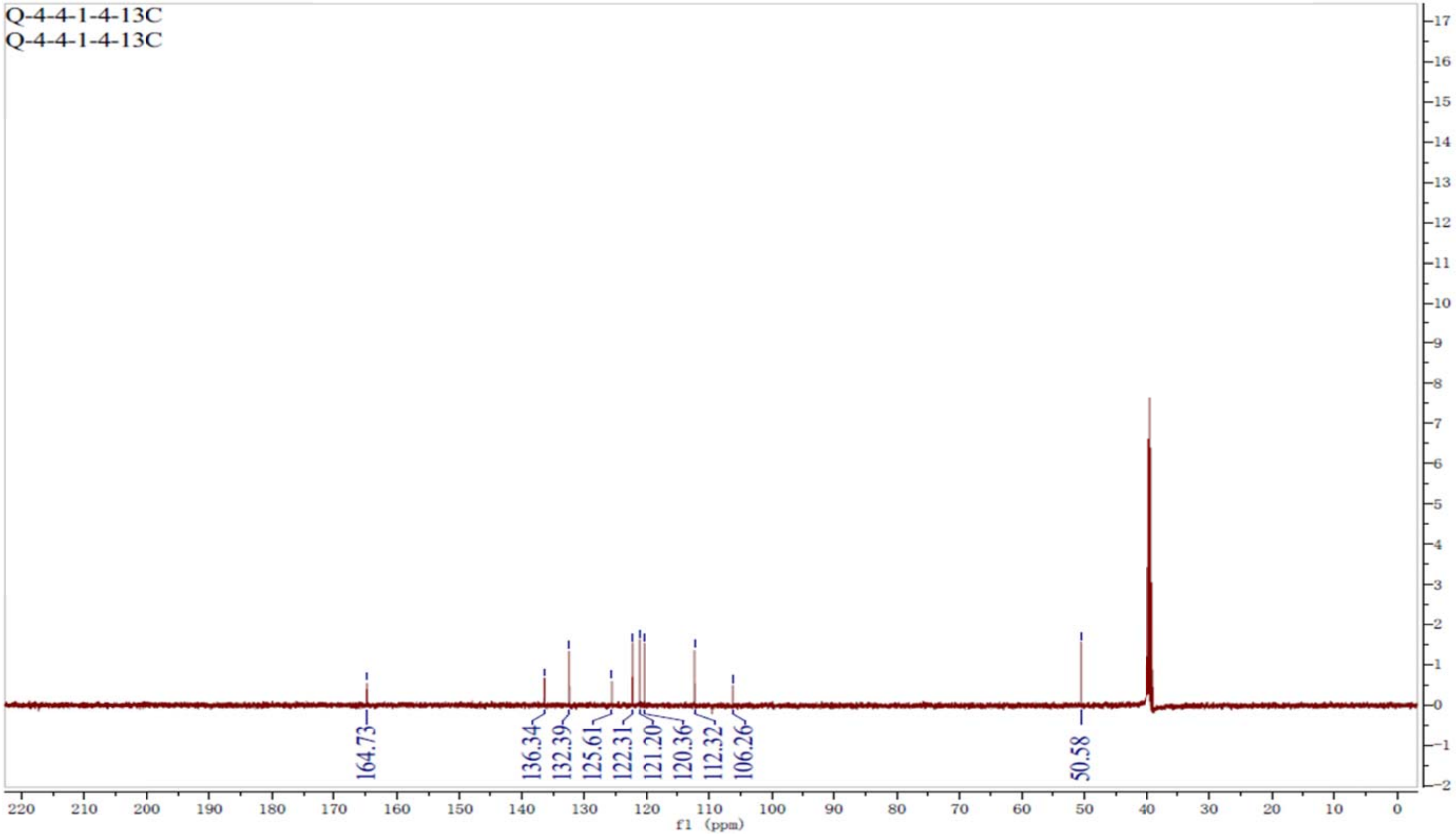

Figure S78. ^{13}C NMR (125 MHz, DMSO-d_6) spectrum of **12**.

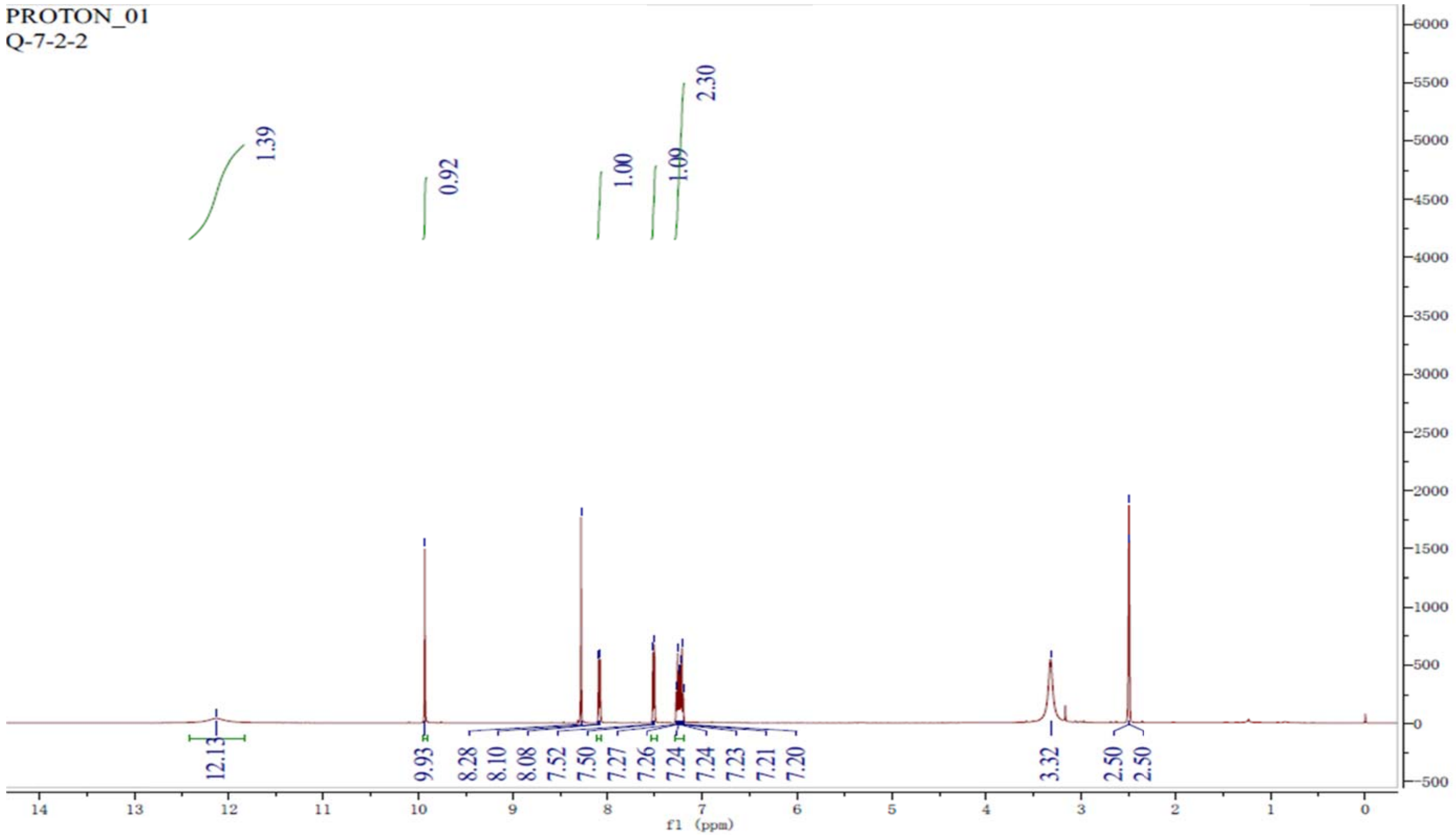

Figure S79. ^{1}H NMR (500 MHz, DMSO-d_6) spectrum of **13**.

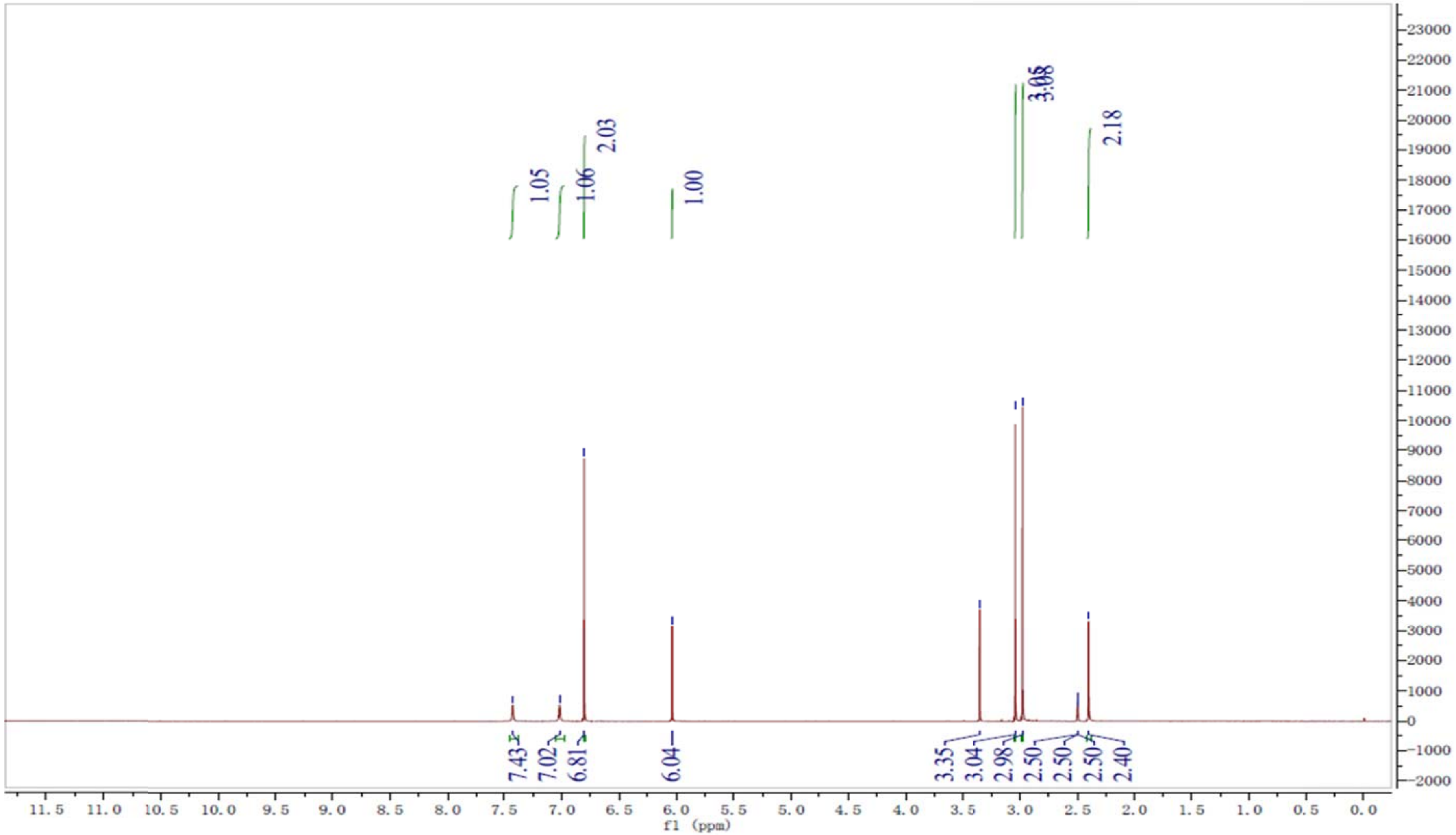

Figure S80. ^{1}H NMR (500 MHz, DMSO-d_6) spectrum of **14**.

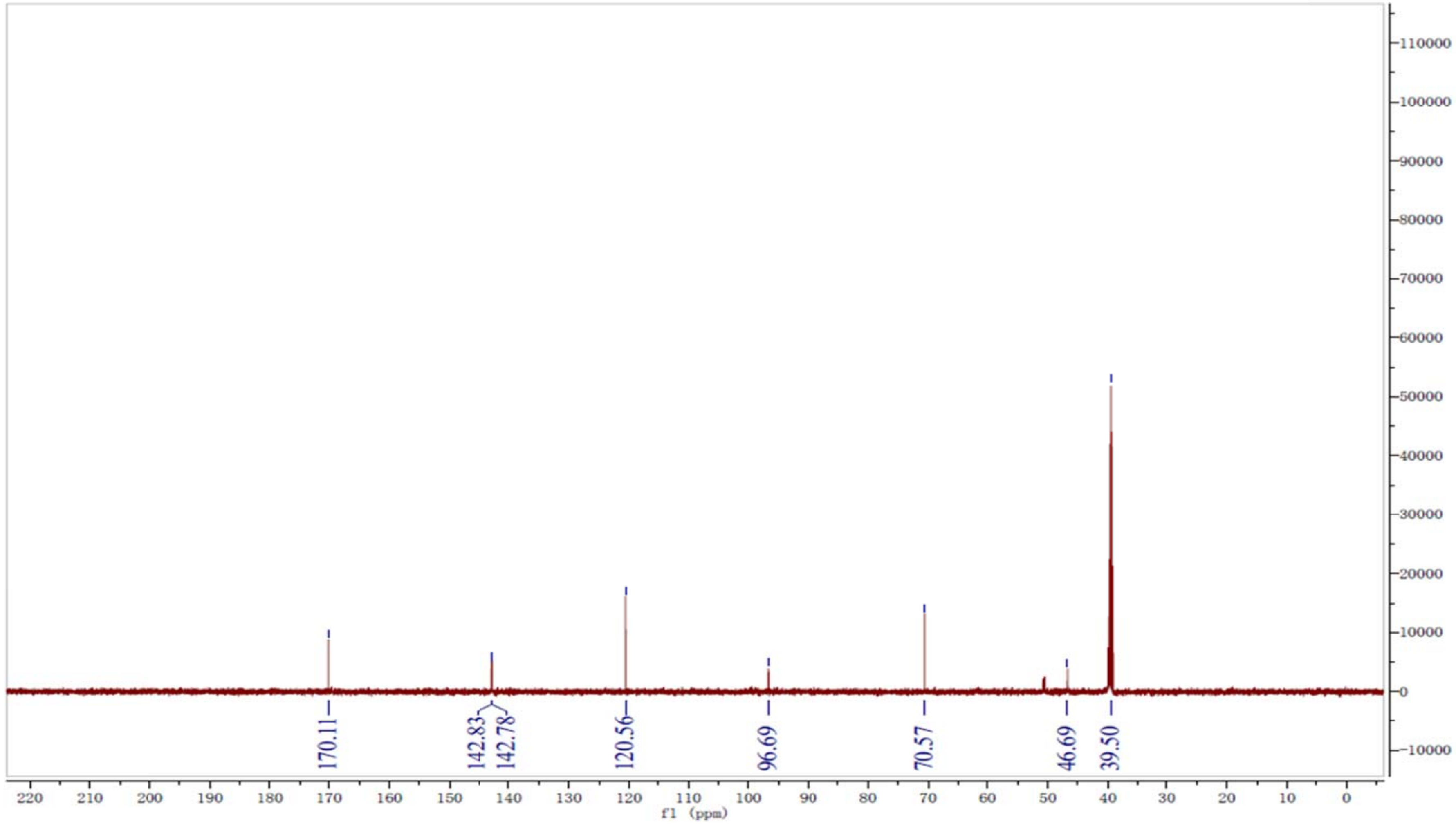

Figure S81. ^{13}C NMR (125 MHz, DMSO-d_6) spectrum of **14**.

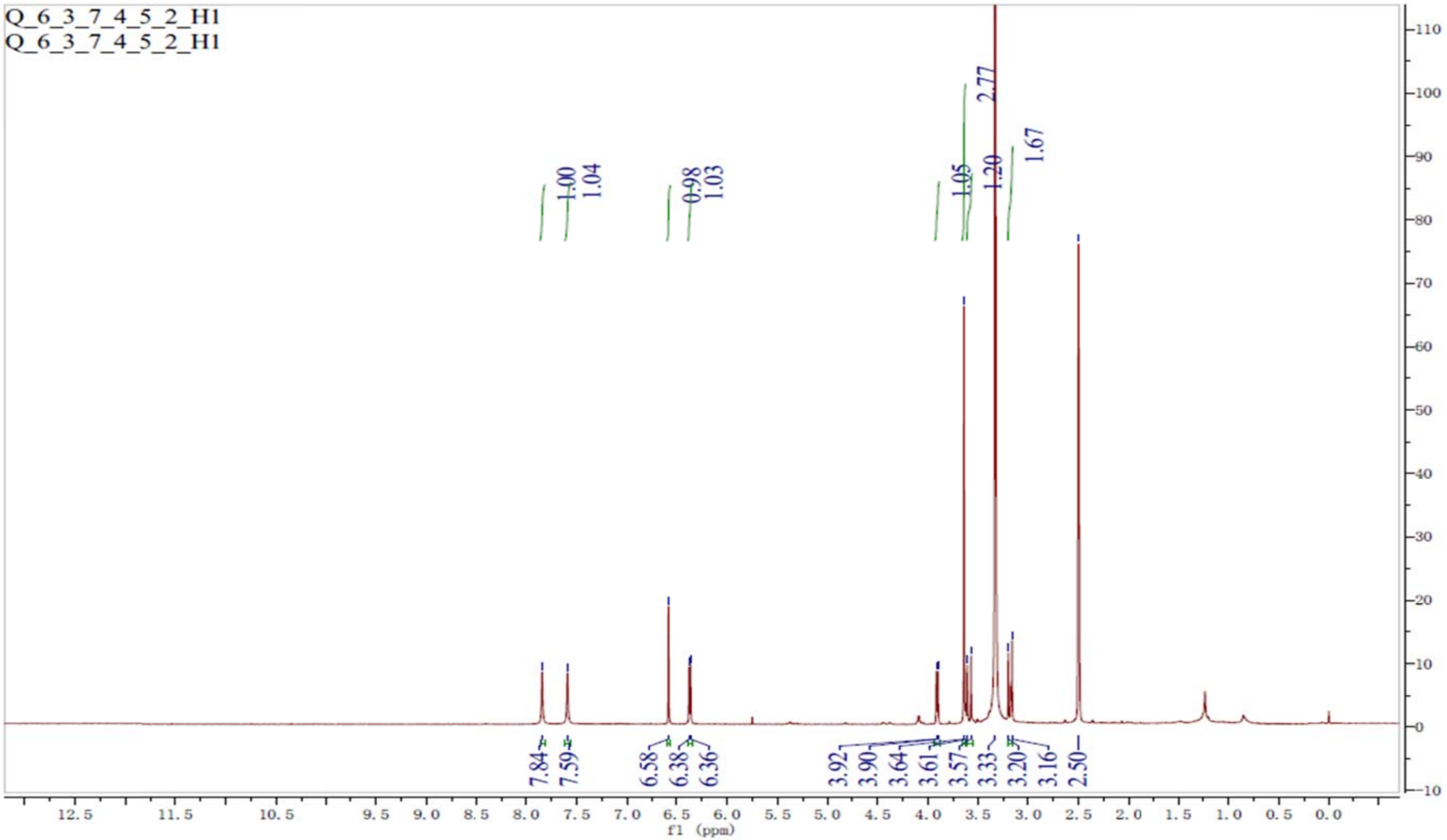

Figure S82. ^{1}H NMR (500 MHz, DMSO-d_6) spectrum of **15**.

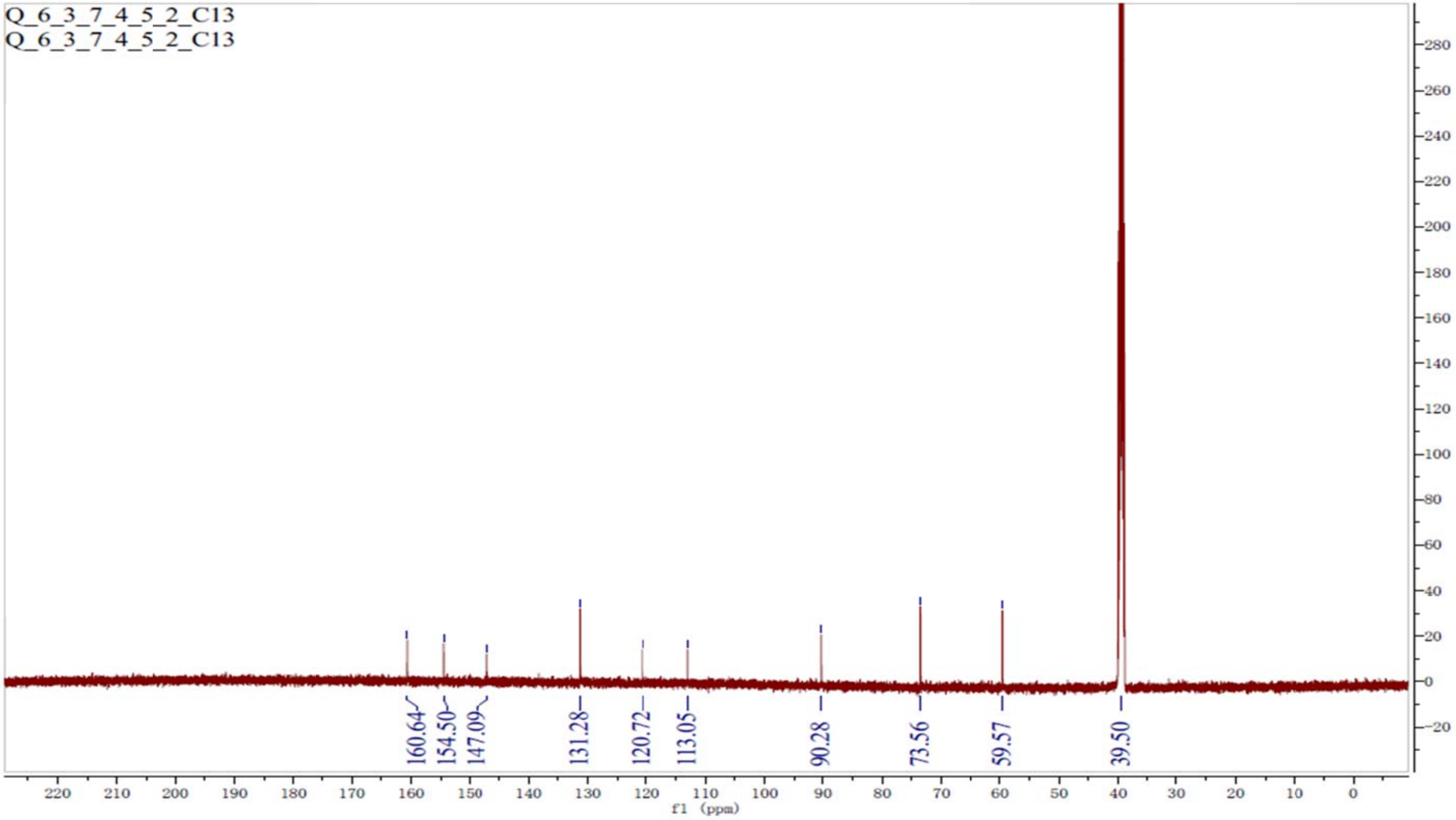

Figure S83. ^{13}C NMR (125 MHz, DMSO-d_6) spectrum of **15**.

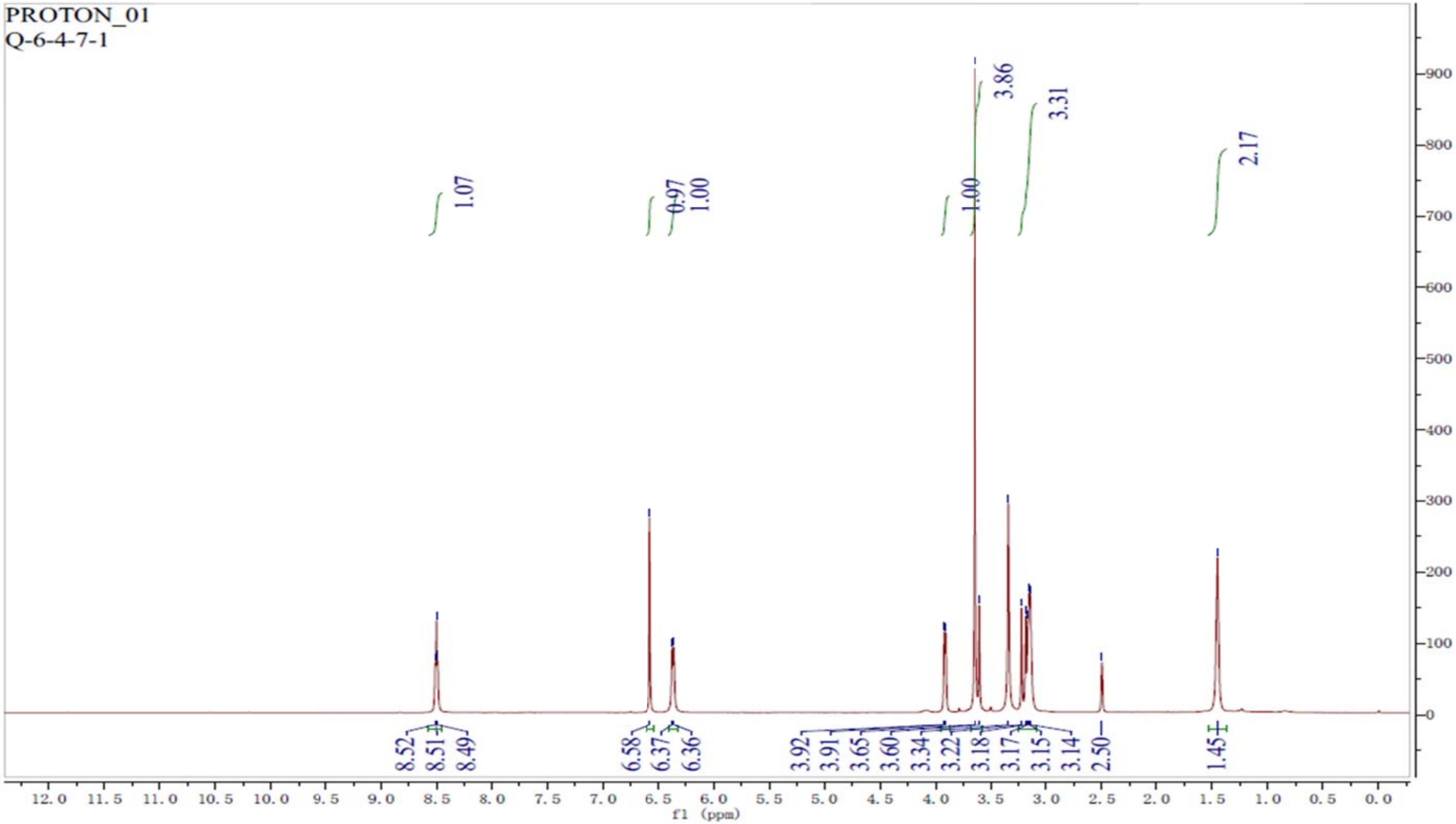

Figure S84. ^{1}H NMR (500 MHz, DMSO-d_6) spectrum of **16**.

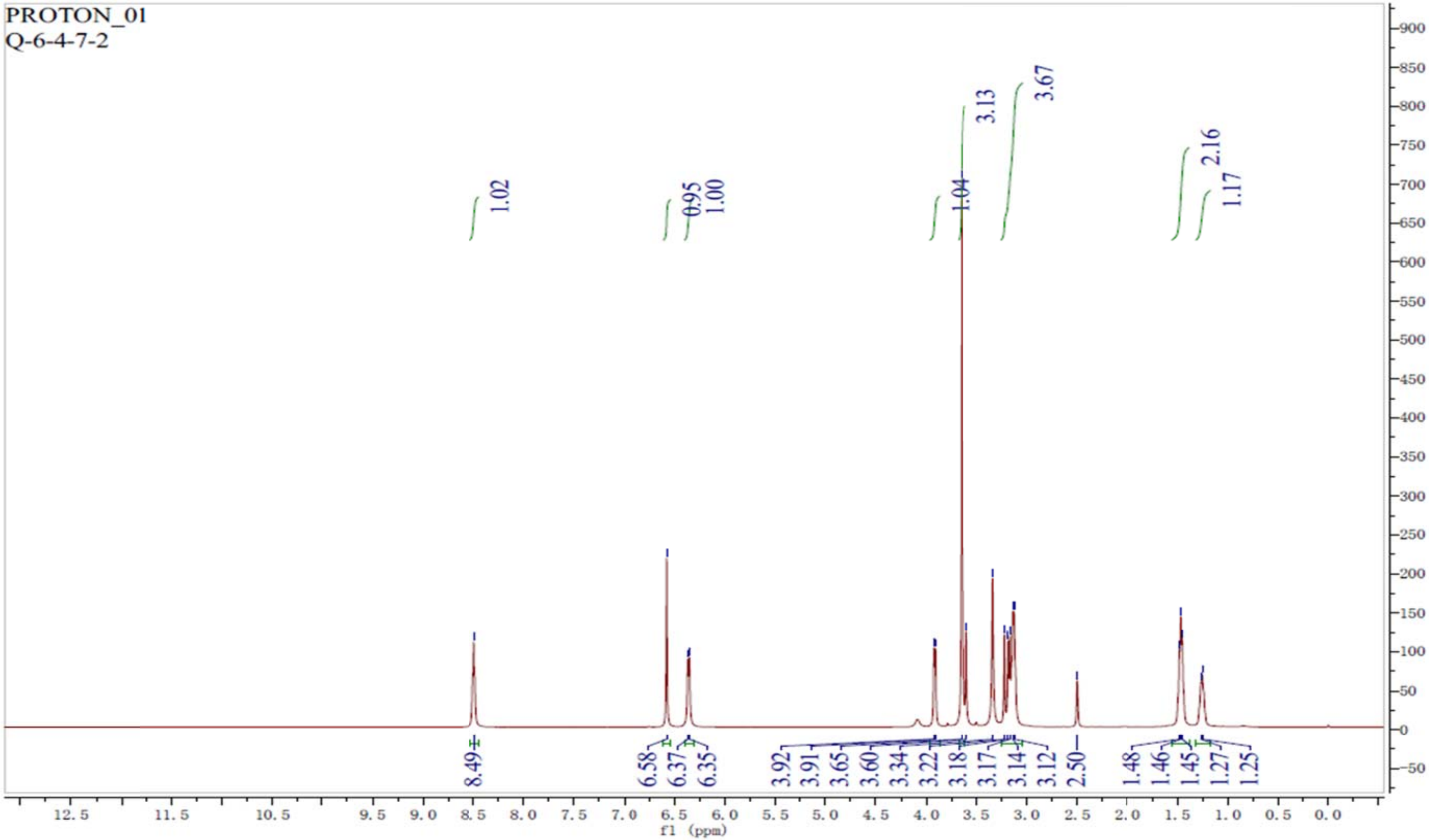

Figure S85. ^{1}H NMR (500 MHz, DMSO-d_6) spectrum of **17**.
